# More is not always better: An experimental individual-level validation of the randomized response technique and the crosswise model

**DOI:** 10.1371/journal.pone.0201770

**Published:** 2018-08-14

**Authors:** Marc Höglinger, Ben Jann

**Affiliations:** 1 Zurich University of Applied Sciences, Winterthur Institute of Health Economics, Winterthur, Switzerland; 2 University of Bern, Institute of Sociology, Bern, Switzerland; Mälardalen University, SWEDEN

## Abstract

Social desirability and the fear of sanctions can deter survey respondents from responding truthfully to sensitive questions. Self-reports on norm breaking behavior such as shoplifting, non-voting, or tax evasion may thus be subject to considerable misreporting. To mitigate such response bias, various indirect question techniques, such as the randomized response technique (RRT), have been proposed. We evaluate the viability of several popular variants of the RRT, including the recently proposed crosswise-model RRT, by comparing respondents’ self-reports on cheating in dice games to actual cheating behavior, thereby distinguishing between false negatives (underreporting) and false positives (overreporting). The study has been implemented as an online survey on Amazon Mechanical Turk (*N* = 6, 505). Our results from two validation designs indicate that the forced-response RRT and the unrelated-question RRT, as implemented in our survey, fail to reduce the level of misreporting compared to conventional direct questioning. For the crosswise-model RRT we do observe a reduction of false negatives. At the same time, however, there is a non-ignorable increase in false positives; a flaw that previous evaluation studies relying on comparative or aggregate-level validation could not detect. Overall, none of the evaluated indirect techniques outperformed conventional direct questioning. Furthermore, our study demonstrates the importance of identifying false negatives as well as false positives to avoid false conclusions about the validity of indirect sensitive question techniques.

## 1 Introduction

Surveying sensitive topics such as deviant behavior, stigmatizing traits, or controversial attitudes poses serious challenges to survey research. First, respondents’ data need to be carefully protected, particularly for sensitive themes like illegal behavior or politically repressed opinions. Second, even with good data protection, respondents might be tempted to misreport on sensitive questions or refuse to answer, for example, due to embarrassment or due to fear of negative sanctions [1]. To avoid biased or incomplete measurement, survey researchers therefore have to find questioning procedures that maximize respondents’ willingness to provide truthful answers.

Various approaches to address this issue have been pursued in previous research, but the results on the success and the failure of the different questioning strategies appear inconsistent and highly dependent on implementation details, the research question, or the studied population [2]. Most promising results can be found with respect to survey mode and, in particular, to whether an interviewer is present or not. For example, Kreuter et al. [3] compared CATI (Computer Assisted Telephone Interviewing), IVR (Interactive Voice Response), and online mode in a study on poor (and potentially embarrassing) academic performance among university alumni, where the respondents’ answers could be validated against the university’s grade records. The level of misreporting (false denial of poor performance) was highest in CATI mode, where an interviewer was present. However, also in the more anonymous IVR and online modes, misreporting remained high.

### 1.1 The randomized response technique

Other approaches try to mitigate misreporting and non-response by employing so-called indirect question techniques, one of which is the randomized response technique (RRT, originated by Warner in 1965 [4]). The basic idea of the RRT is to protect respondents through random misclassification so that a given answer does not reveal the true answer to the sensitive question. Ideally the anonymity induced by the misclassification makes respondents more comfortable providing truthful answers. For example, in the forced-response variant of the RRT [5] a randomizing device such as a coin flip determines whether a respondent is instructed to provide a truthful answer to a sensitive yes/no question or simply respond with “yes” (or “no”), irrespective of the true answer. Therefore, as long as only the respondent knows the outcome of the randomizing device, a given answer does not reveal the true answer to the sensitive question; the given answer could also just be a surrogate response due to the randomizing device.

Despite the theoretical appeal of the RRT, it remains questionable whether all respondents understand the procedure, trust that their anonymity is protected, and are more inclined to provide a truthful answer (when instructed to do so). Existing findings suggest that the level of perceived privacy, the main rationale for implementing indirect techniques, is far from satisfactory [6, 7]. Furthermore, due to lack of understanding, respondents might fail to comply with the RRT instructions even if they are asked to provide an answer that is unrelated to the sensitive question [8–10]. A meta analysis by Lensvelt-Mulders et al. [11], mostly covering face-to-face and paper-and-pencil RRT studies published between 1965 and 2000, concludes that, on average, the RRT yields more valid results than direct questioning, but the variability in results is high. Furthermore, findings from a number of newer studies on the application of the RRT in online mode are not very promising. With few exceptions, RRT estimates of socially undesirable or socially desirable behaviors turned out to be either no different from direct questioning estimates, or different in the “wrong” direction [6, 12–15].

### 1.2 The crosswise-model RRT

Recently, a variant of the RRT, the “crosswise model,” proposed by Yu et al. [16], has received growing attention. Several studies report that the crosswise-model RRT consistently produces higher prevalence estimates of sensitive behaviors than direct questioning [6, 17–24]. The crosswise-model RRT works by presenting two yes/no questions to the respondent, a sensitive question and an unrelated non-sensitive question, and then asking whether the answers to both questions are the same (both “yes” or both “no”) or whether the two answers are different (one “yes,” one “no”). The advantages of the crosswise-model RRT over alternative RRT variants, it is argued, are that the instructions are easy to understand, the response options are obviously ambiguous with respect to the sensitive question (i.e., there is no clear self-protective answering strategy), and no respondents are forced to give “false” answers.

### 1.3 Validation of indirect question techniques

As mentioned above, results from studies evaluating indirect question techniques are often inconclusive. One reason for the variability in the findings is that the studies employ different validation strategies.

By far the most frequent approach is to use the results from direct questioning as a baseline, to which the results from one or several indirect question techniques are compared. We use the term *comparative validation study* to refer to studies employing such an approach. The argument is that if the question is sensitive, respondents will tend to underreport when asked to answer the question directly. An indirect question technique that successfully reduces underreporting should therefore yield higher estimates than direct questioning (likewise, if the problem is over-reporting, such as in questions on voter turnout, a successful indirect technique should yield lower estimates than direct questioning). Hence, comparative validation studies rely on the so-called more-is-better (less-is-better) assumption [11]; an indirect question technique is considered more valid if it produces higher (lower) prevalence estimates than direct questioning. More generally, if comparing multiple indirect techniques, the technique producing the highest (lowest) estimate is judged to be the most valid.

The more-is-better assumption is often legitimate. In many cases it is reasonable to assume that respondents avoid socially undesirable answers and thus underreport on sensitive questions. However, sometimes, social desirability might differ between subpopulations, a well-known example being the number of sexual partners as reported by men and women [25, 26]. Therefore, the more-is-better assumption can sometimes be challenged on the ground that social desirability bias points in different directions depending on the subpopulation.

Furthermore, even if the more-is-better assumption is justified, a higher estimate from an indirect question technique does not necessarily imply that the technique produces more valid measurements than direct questioning. If it is true that direct questioning yields underestimation, then higher estimates by an indirect technique is a necessary condition, but not a sufficient condition. The more-is-better assumption assumes that the increase in estimates is due to more truthful answers. However, given the complexity of the instructions of most RRT implementations it may also be simply due to the respondents’ inability to correctly apply the procedure. That is, the increase in estimates might be due to non-compliance with the RRT instructions (e.g., due to problems with the randomizing device, misunderstanding of instructions, or unwillingness to follow the instructions) rather than more truthful answering. Overall, we conclude that comparative validation studies can only provide weak support for the validity of sensitive question techniques (for similar arguments see [6, 11, 27, 28]).

At least some of the shortcomings of comparative validation studies can be overcome by what we call *aggregate-level validation studies*. In such studies, the true population prevalence of the sensitive trait or behavior is known from an external and reliable source or can be determined based on theoretical reasoning. For example, in studies of voter turnout, true aggregate turnout is known from administrative records (for recent examples see [29] and [30]). If the true value is known, then overestimation and underestimation by different question techniques can be observed directly without having to resort to direct questioning as a baseline, which is a clear improvement over comparative validation studies.

Yet, also such aggregate-level validation studies might be inconclusive. First, true values might differ from the assumed value, perhaps because the study focuses on a special subpopulation or because there is sample selection bias (e.g., due to nonresponse). Second, and more importantly, a close match between the prevalence estimate from a particular sensitive question technique and the true value does not necessarily imply that the technique produces valid measurements at the individual level. As argued above, different mechanisms might affect the prevalence estimate, not all of which are consistent with more truthful answering. In other words, apart from possible sample selection bias, the aggregate-level validation approach rests on the assumption that socially desirable responding is the only misreporting mechanism.

A useful distinction in this context is between false negatives (or true positives) and false positives (or true negatives). The goal of sensitive question techniques is to reduce the number of false negatives, that is, the number of respondents who deny the sensitive question even though it does apply. However, a sensitive question technique might also increase the number of false positives, that is, the number of respondents who agree with the sensitive question even though it does not apply. Comparing overall prevalence estimates from the technique with either direct questioning or a known “true” prevalence, does not allow one to distinguish between a reduction in false negatives and an increase in false positives, both of which will increase the estimated total prevalence. To be able to disentangle the two effects, validation data at the individual level is required. Hence, we argue that *individual-level validation studies*—studies in which true values of the sensitive trait or behavior are known at the individual level—are necessary to be able to evaluate the degree to which a technique does, in fact, produce valid measurements. In the context of indirect questioning techniques one could even go one step further and also observe the outcomes of the randomizing device or the values of the unrelated question (which, however, we did not in this study).

Despite their clear advantage over the comparative approach, individual-level validation studies are very rare. Reviewing RRT studies from over 35 years, Lensvelt-Mulders et al. [11] counted just six published individual-level validation studies dealing with sensitive topics such as convictions, arrests, welfare fraud, or failing university courses. We are aware of five additional studies published since [18, 27, 28, 31, 32]. The available validation studies provide valuable insights, but they do not explicitly focus on disentangling false negatives and false positives. Moreover, some of the studies use a sample that only includes respondents who possess the sensitive trait or engaged in the sensitive activity, so that, by design, only false negatives can be studied. In sum, we believe that additional individual-level validation studies are necessary to disentangle the different response mechanisms and to examine the possibility of false positives in these types of survey techniques. Such studies are the only way to conclusively assess the performance of different sensitive question techniques.

### 1.4 Our study

The goal of our study is to evaluate the validity of some popular variants of the RRT using a validation design that does not rely on the more-is-better assumption and that allows separate analysis of false negatives and false positives. To achieve this we conducted an online survey on Amazon Mechanical Turk (*N* = 6, 505), in which the respondents had the opportunity to play one of two dice games. We used two different dice games to evaluate whether results are robust across different designs. Respondents were given monetary incentives to cheat in these games. After playing the games, respondents were asked about whether they cheated, using direct questioning or an implementation of one of the following three popular RRT variants: forced-response RRT, unrelated-question RRT, and the crosswise-model RRT. For the first game cheating was not directly observable and, hence, respondents could cheat without any risk of disclosure. Nonetheless, the proportion of cheaters, overall as well as within subgroups, can be estimated based on the laws of chance. For the second game cheating was observable and hence, as expected, cheating occurred less frequently. Comparing the cheating behavior in the two games with the answers to the cheating question reveals the degree to which the different questioning techniques are successful in eliciting truthful answers.

## 2 Data and methods

Study participants were US residents recruited via the online platform Amazon Mechanical Turk (AMT). AMT is an online crowdsourcing marketplace where “requesters” can post tasks (called “Human Intelligence Tasks” or HITs) that can then be completed by “workers” in exchange for money. HITs are announced with a short description of the task and the corresponding payment. In our study, participants completed a voluntary and anonymous online survey. Subjects were informed about the issuer, content, and length of the survey before they could choose whether they want to participate. Participation in the study could not have any negative consequences whatsoever for the subjects and anonymity was granted at all times. According to local (Swiss) law, the study does not fall under the Human Research Act, hence does not require authorization from an ethics committee. Therefore, we do not have an approval from a formal Institutional Review Board for this study.

AMT is suitable for any task that can be easily outsourced online to an anonymous workforce and is frequently used to recruit participants for scientific surveys and experiments [33–35]. AMT samples are certainly not representative of the general population. Furthermore, there is evidence of widespread non-naivety (i.e., that many AMT participants are familiar with common experimental tasks from behavioral studies) and more dishonesty compared to other crowdsourcing platforms [36]. However, AMT samples have been shown to be sufficiently heterogeneous and, with respect to results from experimental research, comparable to other samples [37, 38]. Furthermore, non-naivety and elevated dishonesty seem to be particularly prevalent among quick responders [39]. Using a large sample size and an extended recruitment period, as in our study, should lead to a more balanced sample. Moreover, with respect to our study, a somewhat elevated proportion of dishonest behavior could, in fact, be beneficial as it puts the evaluated sensitive question techniques to a stronger test and makes flaws in the techniques better detectable.

On November 5, 2013, we posted a HIT that asked for filling out a scientific survey on “Mood and Personality” for a base payment of $1 and the prospect of winning an additional $2 bonus payment. The HIT was closed on December 5, 2013, when we reached the targeted sample size of 6,500 participants (the target sample size was set ex ante based on approximate considerations of expected statistical power). Workers who accepted our HIT received an access link to the survey. After having completed the survey, they received payment. To identify untrustworthy participants, we employed a screening question from Berinsky et al. [40], which was passed by 97% of the respondents. The median time required to complete the survey was 6.7 minutes. Details on the study and screenshots of the questionnaire are available in S1 Documentation.

A total of 6,505 participants were recruited, of which 6,473 completed the survey at least up to the part containing the sensitive questions. Only the latter are included in our analysis. Furthermore, we exclude 205 participants who did not pass the screening question, 115 participants who did not roll the die in the dice game (or for whom the result of the roll was not recorded due to technical problems, e.g. because of script blockers), and 1 participant who won in the roll-a-six game but did not claim his legitimate bonus payment (we exclude this observation to simplify the analysis; see below). The final sample size for our analysis is *N* = 6, 152.

As displayed in Table 1, the sample has an even gender distribution and the majority of respondents are under 35 (mean age 32). Respondents are relatively well educated, with 88 percent having attended at least some college. About two thirds are employed or self-employed. A large majority of respondents completed the survey at home and most respondents had extensive experience with “scientific studies such as surveys or experiments on MTurk” (wording from the questionnaire; the median number of previous MTurk studies is 50).

**Table 1:** Descriptive statistics of the sample.

Variable	Category	Percent
Gender	male	49.9
	female	50.1
Age	18–24	24.3
	25–29	27.0
	30–34	18.5
	35–39	10.7
	40–49	10.1
	50 or older	9.3
Education	college degree	54.0
	some college	34.2
	high school or other	11.8
Labor market status	employed	54.1
	self-employed	12.7
	unemployed	11.3
	student	13.0
	other	8.9
Prior MTurk studies	0	6.8
	1–9	19.3
	10–99	32.9
	100–999	30.2
	1000 or more	10.8
Current location	at home	85.4
	at work	9.9
	other	4.7

Labor market status recoded from multiple response data (prioritizing categories in the order as listed in the table); *N* = 6, 152.

### 2.1 The dice games

Participants were randomly assigned to one of two dice games in which they could win a $2 bonus payment: the *prediction game* or the *roll-a-six game*. The games were inspired by Greene and Paxton [41] and Fischbacher and Heusi [42] (also see [43, 44]). In both games, participants used a digital online die embedded in the questionnaire that could be “rolled” by clicking on a button. Roll outcomes were randomized and followed a uniform distribution. The die could be rolled several times, but as explained to the respondents, only the first roll counted.

In the *prediction game* participants had to correctly predict the outcome of a die roll to win the $2 bonus payment. On a first screen, the rules of the game and the conditions under which a participant would win the bonus payment were explained. On the second screen, participants were asked to make their prediction (in private) and memorize it. On the third screen they were instructed to roll the die, inspect the result, and then indicate whether their prediction was correct or not. Because the prediction was made in private, it was obvious that one could cheat without any risk of disclosure. However, even though cheating in this game is not directly observable, the proportion of cheating respondents can be estimated, at the overall level and also within subgroups, because the chance of winning is known to be one sixth by design.

In the *roll-a-six game* participants had to roll a six in order to win the $2 bonus payment. Respondents were again presented a first screen on which the game was explained. On the second screen they were instructed to roll the die and then indicate whether the result was a six or not. In contrast to the prediction game also the identification of individual cheaters is possible in this game since the outcomes of the die roll were recorded. Although respondents were not told that the outcomes would be tracked, it was obvious that this was possible, and hence, the proportion of cheaters can be expected to be lower in the roll-a-six game than in the prediction game. Likewise, when asked about whether they cheated, cheating respondents in the roll-a-six game may be expected to provide more truthful answers than cheating respondents in the prediction game.

We used two dice games because both games have their strengths and weaknesses. In the prediction game a high proportion of cheaters can be expected because individual cheaters cannot be singled out. For the same reason, however, analysis of the resulting data and, in particular, the separation of false negatives and false positives requires some extra assumptions (see Data analysis below). In the roll-a-six game, the identification of individual cheaters is possible and, hence, data analysis is straightforward, but the proportion of cheaters is likely to be low and more selective than in the prediction game.

### 2.2 The sensitive question techniques

In the second part of the questionnaire, respondents were asked four “sensitive” questions, the last of which being about whether they gave an honest answer in the dice game (see Table 2).

**Table 2:** Sensitive questions.

Item	Wording
Shoplifting	“Have you ever intentionally taken something from a store without paying for it?”
Tax evasion	“Have you ever provided misleading or incorrect information on your tax return?”
Non-voting*	“Did you vote in the 2012 US presidential election?”
Cheating in dice game*	Prediction game: “In the $2 dice task at the beginning of this survey: Did you honestly report whether your prediction of the dice roll was right?”
	Roll-a-six game: “In the $2 dice game at the beginning of this survey: Did you honestly report whether you actually rolled a 6?”

* Reverse coded for the purpose of analysis.

To evaluate different sensitive question techniques, respondents were randomly assigned to one of four conditions: direct questioning (DQ), the crosswise-model RRT (CM), the unrelated-question RRT (UQ), or the forced-response RRT (FR). Table 3 reports the number of observations per sensitive question technique and dice game variant. Respondents were randomized into the different conditions with a probability of 1/8 for DQ, 3/8 for CM, 2/8 for UG, and for 2/8 FR. These probabilities were employed to achieve comparable statistical precision for all techniques. Item-nonresponse was negligible; below 1% for all sensitive questions in all experimental conditions. We therefore refrain from reporting results on item-nonresponse in the analyses below.

**Table 3:** Number of observations by dice game variant and sensitive question technique.

	Prediction game	Roll-a-six game
Direct questioning (DQ)	387	382
Crosswise-model RRT (CM)	1168	1145
Unrelated-question RRT (UQ)	760	780
Forced-response RRT (FR)	759	771

Direct questioning (DQ) was included as a benchmark for the evaluation of the different sensitive question techniques. The sensitive questions were introduced by a screen announcing some sensitive questions, stating the importance of honest answers for the success of the study, providing privacy assurance, and telling the respondents that their answers to the sensitive questions would not affect their payment or the HIT approval (this introductory screen was identical for all conditions). After that, the four sensitive questions followed on four separate screens.

For the crosswise-model RRT (CM) we used an implementation proposed by Jann et al. [20]; similar implementations have been used in most other studies on the crosswise-model RRT. Respondents were asked two questions: A sensitive question and an unrelated non-sensitive question. Respondents then had to indicate whether their answers to the two questions were the same (both “no” or both “yes”) or different (one “yes,” one “no”) without reporting the individual answers. The unrelated questions, which were randomly paired with the sensitive questions for each respondent, asked about the birthday (in January or February, between the 1^st^ and the 6^th^ of the month) of the respondent’s mother or father. Between the introductory screen and the screen with the first sensitive question, an additional screen was displayed explaining the question technique and how it protects anonymity (similar screens were also displayed for the other indirect question techniques).

For the unrelated-question RRT (UQ) we used an implementation as proposed by Diekmann [45]. Respondents were asked to think of an acquaintance and use the first digit of this person’s house number as their personal random number. If their random digit was 1, 2, 3, 4, or 5, respondents then had to answer the subsequent sensitive questions; otherwise they had to answer the subsequent unrelated non-sensitive questions. Diekmann [45] provides evidence that first digits of house numbers follow “Benford’s Law”. Accordingly, the probability of 1, 2, 3, 4, or 5 (i.e., of having to answer the sensitive questions) is 0.778. To evaluate whether Benford’s Law holds, we included a question on the first digit of an acquaintance’s address for a subsample of respondents in a different experimental condition. The proportion of respondents reporting a 1, 2, 3, 4, or 5 was 0.784 (95% confidence interval: 0.763 to 0.804). Similar tests were included for all unrelated questions used in CM and UQ. Since deviations between the theoretical values (assuming an even distribution of birthdays) and the estimated proportions were only small, we focus on results based on the theoretical values in the analyses below. The unrelated questions were randomly paired with the sensitive questions for each respondent and asked about the birthday of the respondent’s mother (in January–June, in an even-numbered month, in the first half of the month, on an even-numbered day, in an even-numbered year).

Respondents in the forced-response RRT (FR) implementation (adopted from Höglinger et al. [6]) were presented twelve fields on the screen, numbered from one to twelve. They were told to privately choose a field and memorize their choice (without clicking on the field). Then, they were told to click a “Show instructions” button to uncover the instructions hidden within the fields and follow the instruction that appeared in the field of their choice. Possible instructions were “Answer question”, “Directly tick yes”, or “Directly tick no”. The instructions were randomized across fields.

Because participants might have been troubled by the sensitive questions, all respondents were shown a debriefing page at the end of the survey (see page 56 in S1 Documentation). The debriefing page stated the goal of the study and assured the respondents that their answers to the sensitive questions will be kept confidential. The respondents were also assured that they will receive their bonus payments irrespective of these answers.

### 2.3 Data analysis

The RRT leads to data misclassification so that adjusted methods for data analysis are required. Let Y* be the (unobserved) answer to the sensitive question (Y* = 1 if the answer is “yes”, Y* = 0 else) and *Y* be the observed response (*Y* = 1 if the response is “yes” in case of DQ, UQ and FR or “the same” in case of CM; *Y* = 0 else). Throughout this discussion we assume that “yes” is the sensitive answer, although some of the sensitive questions in our study were framed differently (for example, we asked respondents whether they played honestly in the dice game, not whether they cheated). For the purpose of analysis, all data was appropriately recoded.

The RRT procedures introduce misclassification so that *Y* ≠ Y*. In general, in a misclassification setting, the relation between *Y* and Y* can be described as

$$\begin{array}{c} \mathrm{Pr}\left(Y=1\right)=\mathrm{Pr}\left(Y=1|Y^{*}=1\right)\mathrm{Pr}\left(Y^{*}=1\right)+\mathrm{Pr}\left(Y=1|Y^{*}=0\right)\mathrm{Pr}\left(Y^{*}=0\right) \end{array}$$

Solving for Pr(Y* = 1) yields

$$\begin{array}{c} \mathrm{Pr}\left(Y^{*}=1\right)=\frac{\mathrm{Pr}\left(Y=1\right)-p_{1|0}}{p_{1|1}-p_{1|0}} \end{array}$$

where we use shorthand notation *p*_1|1_ = Pr(*Y* = 1|Y* = 1) and *p*_1|0_ = Pr(*Y* = 1|Y* = 0) for sake of brevity. In the RRT, *p*_1|1_ and *p*_1|0_ are known by design. Hence, we can estimate Pr(Y* = 1) by inserting a sample estimate for Pr(*Y* = 1) (i.e., the sample mean $\bar{Y}$) into the above formula. Furthermore, since Pr(Y* = 1) is a linear transformation of Pr(*Y* = 1) and, in general, *V*(*ax* + *b*) = *a*^2^*V*(*x*) [46], the sampling variance of estimator $\hat{\mathrm{Pr}}\left(Y^{*}=1\right)$ is given as

$$\begin{array}{c} V\left(\hat{\mathrm{Pr}}\left(Y^{*}=1\right)\right)=\frac{1}{{\left(p_{1|1}-p_{1|0}\right)}^{2}}V\left(\hat{\mathrm{Pr}}\left(Y=1\right)\right) \end{array}$$

where $V(\hat{\mathrm{Pr}}\left(Y=1\right))$ can be estimated from the data using standard techniques (e.g., as $\bar{Y}\left(1-\bar{Y}\right)/\left(n-1\right)$ where *n* is the sample size).

For direct questioning, there is no misclassification, so that *p*_1|1_ = 1 and *p*_1|0_ = 0 and hence

$$\begin{array}{c} \mathrm{Pr}\left(Y^{*}=1\right)=\mathrm{Pr}\left(Y=1\right) \end{array}$$

For the CM, let *p*_*Z*_ be the known probability that the answer to the non-sensitive question is “yes”. Then *p*_1|1_ = *p*_*Z*_ and *p*_1|0_ = 1 − *p*_*Z*_. Hence,

$$\begin{array}{c} \mathrm{Pr}\left(Y^{*}=1\right)=\frac{\mathrm{Pr}\left(Y=1\right)+p_{Z}-1}{2p_{Z}-1} \end{array}$$

For UQ, again let *p*_*Z*_ be the known probability that the answer to the non-sensitive question is “yes.” Furthermore, let *p*_*U*_ be the probability that the respondent is instructed to answer the non-sensitive question instead of the sensitive question. We then have *p*_1|1_ = 1 − *p*_*U*_(1 − *p*_*Z*_) and *p*_1|0_ = *p*_*U*_*p*_*Z*_, so that

$$\begin{array}{c} \mathrm{Pr}\left(Y^{*}=1\right)=\frac{\mathrm{Pr}\left(Y=1\right)-p_{U}p_{Z}}{1-p_{U}} \end{array}$$

Finally, for FR, let *p*_*yes*_ and *p*_*no*_ be the probabilities of an unconditional “yes” or “no” answer, respectively. Then *p*_1|1_ = 1 − *p*_*no*_ and *p*_1|0_ = *p*_*yes*_, so that

$$\begin{array}{c} \mathrm{Pr}\left(Y^{*}=1\right)=\frac{\mathrm{Pr}\left(Y=1\right)-p_{yes}}{1-p_{yes}-p_{no}} \end{array}$$

The above formulas can be used to obtain prevalence estimates for the sensitive behaviors. Furthermore, suitably modified least-squares [47] or maximum-likelihood techniques [48] [49] could be used to estimate regression models. Employing the more-is-better assumption or comparing the estimates to the aggregate cheating rates in the dice games, we can then decide which of the techniques works best.

The formulas, however, assume that respondents comply with the instructions so that, for example, no false positives occur (apart from false positives induced by design). If this assumption is violated, then the overall estimates can be misleading. To evaluate the degree to which the techniques produce valid results, we therefore perform separate analyses for those who cheated in the dice game and for those who did not cheat. What we are interested in is the *true positive rate* (TPR) that is, the proportion of cheaters who admit having cheated, and the *false positive rate* (FPR), that is, the proportion of non-cheaters who falsely “admit” having cheated. Furthermore, as an overall measure of validity, we are interested in the *correct classification rate* (CCR). Note that false negatives and false positives do not necessarily imply that respondents deliberately lied about their behavior. For example, some respondents might have rolled the die multiple times and overlooked that only the first roll counted. Others might have engaged in self-deception, believing that their answer was correct while it was not. In such cases our measures may be off in the sense that they no longer exactly quantify the degree to which respondents provide honest answers about what they believe to be true. For example, one reason why the true positive rate does not reach 100% might be that some participants think they won the bonus even though they did not. These participants would be classified as false negatives. We cannot rule out that our results are at least partially affected by such problems. However, we see no reason why these biases should differ by sensitive question technique. Hence, comparisons among techniques should still be valid.

For the roll-a-six game, these analyses are straightforward since cheating is observed at the individual level. Let X* = 1 if the respondent rolled a six and X* = 0 else. Furthermore, let *X* = 1 if the respondent *claimed* having rolled a six and *X* = 0 else. A respondent is identified as a cheater (false winner) if *X* = 1 even though X* = 0. Non-cheaters are given if *X* = X*, that is, if *X* = X* = 1 (true winner) or *X* = X* = 0 (true loser). For sake of simplicity, assume that “reverse” cheating (*X* = 0 even though X* = 1) is nonexistent, that is, assume that there are no respondents who did roll a six but then did not claim the bonus payment (false losers; there was only one out of 516 winners in the roll-a-six game who did not claim the bonus payment; we exclude this observation from the analysis below). The *true positive rate* is then given as

$$\begin{array}{c} \text{TPR}=\mathrm{Pr}\left(Y^{*}=1|X\neq X^{*}\right)=\frac{\mathrm{Pr}\left(Y=1|X\neq X^{*}\right)-p_{1|0}}{p_{1|1}-p_{1|0}} \end{array}$$

and the *false positive rate* is give as

$$\begin{array}{c} \text{FPR}=\mathrm{Pr}\left(Y^{*}=1|X=X^{*}\right)=\frac{\mathrm{Pr}\left(Y=1|X=X^{*}\right)-p_{1|0}}{p_{1|1}-p_{1|0}} \end{array}$$

Furthermore, the *correct classification rate* is

$$\begin{array}{c} \text{CCR}=\text{TPR}\cdot \mathrm{Pr}\left(X\neq X^{*}\right)+\left(1-\text{FPR}\right)\mathrm{Pr}\left(X=X^{*}\right) \end{array}$$

Since X* is observed, all of the above quantities can be readily estimated from the data.

In the prediction game, however, where X* denotes whether the respondent’s prediction was correct and *X* denotes whether the respondent *claimed* that the prediction was correct, X* is unobserved. To identify TPR and FPR in the prediction game we make two assumptions.

**Assumption 1 (A1)** All respondents whose predictions were correct do claim the bonus payment (no false losers), that is, X* = 1 *implies X* = 1, *and X* = 0 implies X* = 0.

As mentioned above, only one of 516 winners in the roll-a-six game did not claim the bonus payment. It appears highly plausible to assume that the proportion of false losers is negligible also in the prediction game.

**Assumption 2 (A2)** *The false positive rate of true winners*, Pr(Y* = 1|*X* = X* = 1), *is equal to the false positive rate of true losers*, Pr(Y* = 1|*X* = X* = 0).

Both types of respondents were honest in the prediction game and we do not see much reason why they should differ in their response behavior when asked about whether they were honest or not. Furthermore, results from the roll-a-six game, where the assumption can be evaluated, do not reveal significant differences between the two groups (see S1 Supporting Information). Note, however, that the composition of the two groups is somewhat different. Among the winners there are *potential* cheaters, that is, respondents who would have cheated should they not have won, as well as non-cheaters. The group of true losers only contains non-cheaters. Differential assumptions about the response behavior of potential cheaters and non-cheaters could be made, but would not fundamentally change our results. In S1 Supporting Information we provide additional results assuming the false positive rate of true winners to be zero (Assumption A2’). Even such an extreme assumption does not change our conclusions.

We can now derive estimable expressions for FPR and TPR. A2 implies Pr(Y* = 1|*X* = X*) = Pr(Y* = 1|*X* = X* = 0) and A1 implies Pr(Y* = 1|*X* = X* = 0) = Pr(Y* = 1|*X* = 0). The *false positive rate* is thus identified as

$$\begin{array}{c} \text{FPR}=\mathrm{Pr}\left(Y^{*}=1|X=X^{*}\right)=\frac{\mathrm{Pr}\left(Y=1|X=0\right)-p_{1|0}}{p_{1|1}-p_{1|0}} \end{array}$$

The *true positive rate*, according to the definition of conditional probability, can be written as the joint probability of cheating and admitting having cheated, divided by the cheating probability:

$$\begin{array}{c} \text{TPR}=\mathrm{Pr}\left(Y^{*}=1|X\neq X^{*}\right)=\frac{\mathrm{Pr}(Y^{*}=1\cap X\neq X^{*})}{\mathrm{Pr}(X\neq X^{*})} \end{array}$$

Our strategy is to solve the numerator and the denominator separately. From the design of the game we know that the probability of a correct prediction is $\mathrm{Pr}\left(X^{*}=1\right)=\frac{1}{6}$. Given A1, the denominator (cheating probability) can thus be written as

$$\begin{array}{c} \mathrm{Pr}\left(X\neq X^{*}\right)=\mathrm{Pr}\left(X=1\right)-\mathrm{Pr}\left(X^{*}=1\right)=\mathrm{Pr}\left(X=1\right)-\frac{1}{6} \end{array}$$

For the numerator, note that A1 implies

$$\begin{array}{c} \mathrm{Pr}\left(Y^{*}=1\cap X\neq X^{*}\right)=\mathrm{Pr}\left(Y^{*}=1\cap X=1\right)-\mathrm{Pr}\left(Y^{*}=1\cap X=X^{*}=1\right) \end{array}$$

where the first term is

$$\begin{array}{c} \mathrm{Pr}\left(Y^{*}=1\cap X=1\right)=\mathrm{Pr}\left(X=1\right)\mathrm{Pr}\left(Y^{*}=1|X=1\right) \end{array}$$

with

$$\begin{array}{c} \mathrm{Pr}\left(Y^{*}=1|X=1\right)=\frac{\mathrm{Pr}\left(Y=1|X=1\right)-p_{1|0}}{p_{1|1}-p_{1|0}} \end{array}$$

and the second term is

$$\begin{array}{c} \mathrm{Pr}\left(Y^{*}=1\cap X=X^{*}=1\right)=\mathrm{Pr}\left(X=X^{*}=1\right)\mathrm{Pr}\left(Y^{*}=1|X=X^{*}=1\right) \end{array}$$

with

$$\begin{array}{c} \mathrm{Pr}\left(X=X^{*}=1\right)=\mathrm{Pr}\left(X^{*}=1\right)=\frac{1}{6} \end{array}$$

according to A1, and

$$\begin{array}{c} \mathrm{Pr}\left(Y^{*}=1|X=X^{*}=1\right)=\frac{\mathrm{Pr}\left(Y=1|X=0\right)-p_{1|0}}{p_{1|1}-p_{1|0}} \end{array}$$

according to A1 and A2. Putting all elements together, the *true positive rate* for the prediction game is identified as

$$\begin{array}{c} \text{TPR}=\frac{\mathrm{Pr}\left(X=1\right)\frac{\mathrm{Pr}\left(Y=1|X=1\right)-p_{1|0}}{p_{1|1}-p_{1|0}}-\frac{1}{6}\frac{\mathrm{Pr}\left(Y=1|X=0\right)-p_{1|0}}{p_{1|1}-p_{1|0}}}{\mathrm{Pr}\left(X=1\right)-\frac{1}{6}} \end{array}$$

The *correct classification rate* is

$$\begin{array}{c} \text{CCR}=\text{TPR}\cdot \left(\mathrm{Pr}\left(X=1\right)-\frac{1}{6}\right)+\left(1-\text{FPR}\right)\left(\mathrm{Pr}\left(X=0\right)+\frac{1}{6}\right) \end{array}$$

We estimate the above quantities and their standard errors by applying joint mean estimation to the components of the formulas and then, since some of the equations contain nonlinear transformations, using the delta method [50] to obtain the sampling variances. Nonparametric bootstrap estimation [51] essentially yields the same results (see S1 Supporting Information).

## 3 Results

### 3.1 Comparative validation

**Fig 1:**
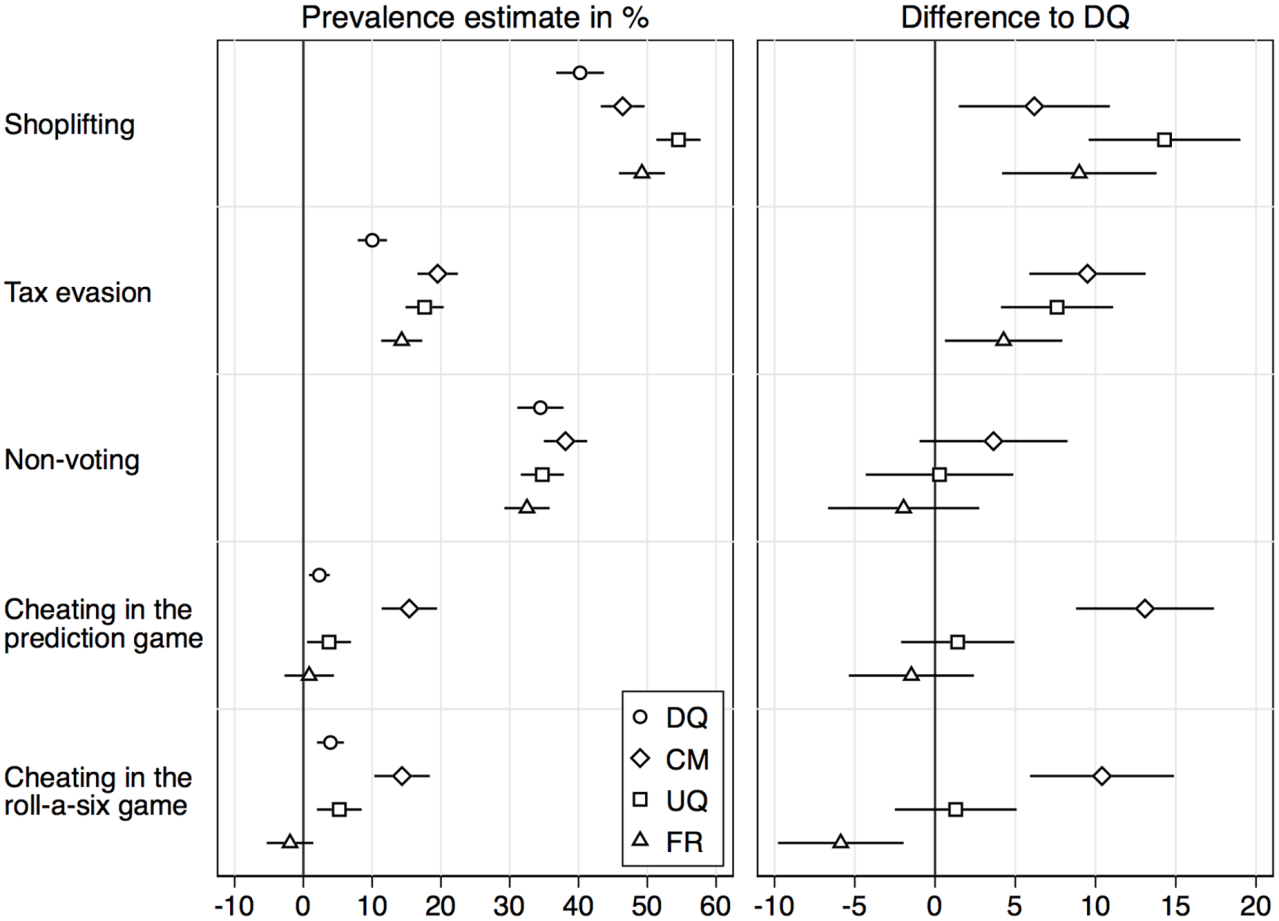
Comparative validation of sensitive question techniques. Point estimates and 95% confidence intervals in percent.

We first report results as in a standard comparative validation study, using the more-is-better assumption. Fig 1 displays the point estimates for the sensitive behaviors from the different sensitive question techniques, as well as the differences in the estimates between direct questioning (DQ) and the indirect techniques (also see S1 Table).

For shoplifting, estimates from all three indirect techniques are significantly higher than the estimate from direct questioning (i.e., the confidence intervals of the differences in the right panel are strictly above zero in all three cases). The highest estimate was obtained by the unrelated-question RRT (UQ). Also for tax evasion, all three techniques significantly outperformed direct questioning, with the crosswise-model RRT (CM) producing the highest estimate. CM also produced the highest estimates for the remaining three items, although the difference to direct questioning is not significant for the non-voting item. The unrelated-question RRT (UQ) and the forced-response RRT (FR) did not produce significantly higher estimates than direct questioning for these three items. Moreover, for cheating in the roll-a-six game, the estimate from FR is significantly lower than the estimate from DQ.

From these results we would conclude that the CM clearly performed best of all techniques; it produced the highest estimates for four of the five items and produced significantly higher estimates than direct questioning for four of the five items. The difference between CM and the other techniques is particularly pronounced for the two cheating items. While cheating rates were 5% or less according to the other techniques, they were about 15% according to CM. The results for UQ and FR are mixed. They outperformed direct questioning for the first two items, but not for the remaining three. For the last item, FR even produced a slightly negative estimate, indicating significant non-compliance with the RRT instructions. A negative estimate is possible if a substantial proportion of respondents deviate from the instructions determined by the randomizing device. This seems to be a common problem with the forced-response RRT (see, e.g., [12]).

### 3.2 Aggregate-level validation

**Fig 2:**
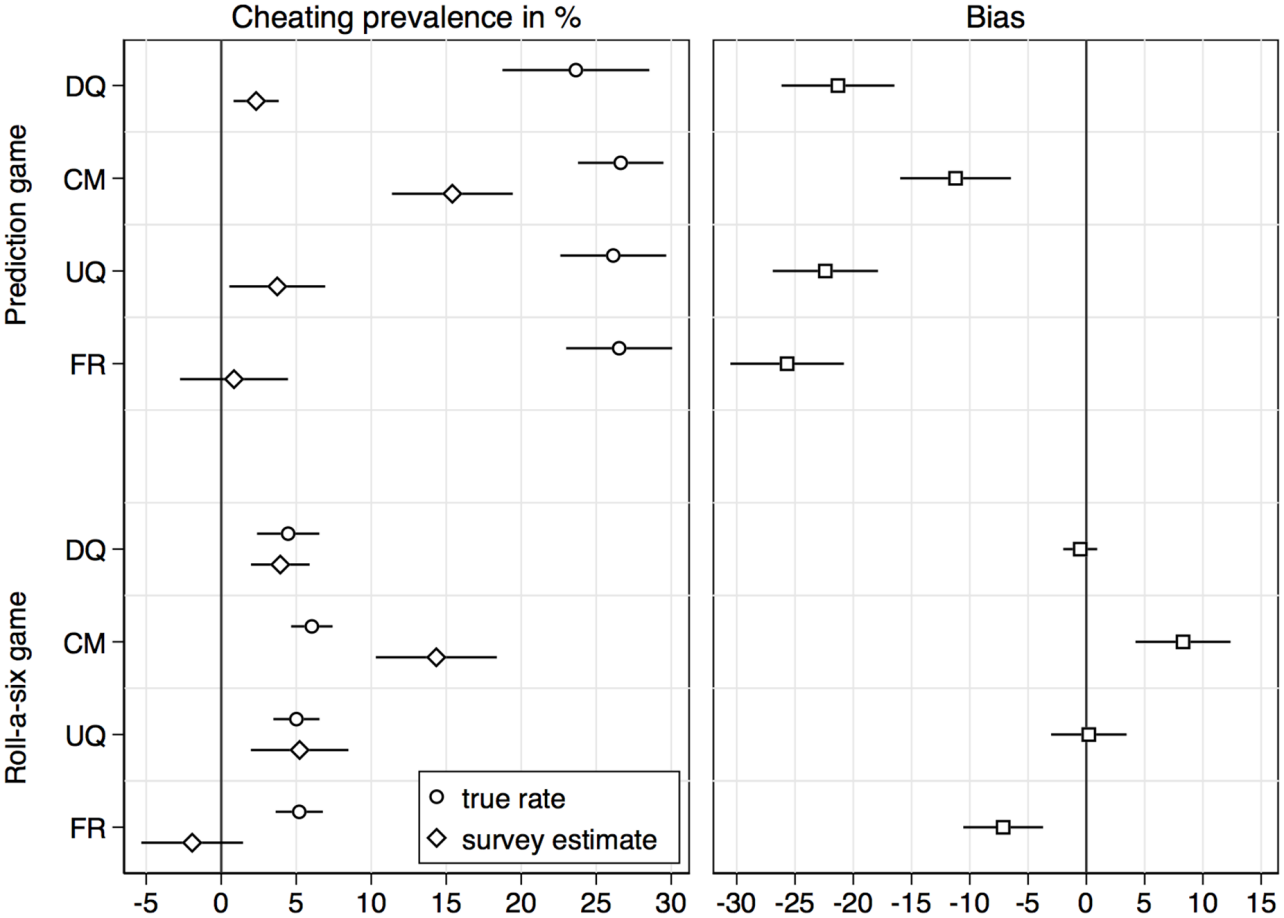
Aggregate-level validation of sensitive question techniques. Point estimates and 95% confidence intervals in percent.

As illustrated above, were we to conduct a comparative validation study based on the more-is-better assumption, we would find that the crosswise-model RRT is the most valid technique, a finding that is consistent with the results from previous comparative validation studies. However, the more-is-better assumption is a strong assumption that might be violated. In the second step, we therefore compare the prevalence estimates from the various techniques to the true prevalence of the sensitive behaviors at the aggregate level. We can conduct such an analysis for the two items on cheating in the dice games. Fig 2 displays the true rates as well as the various estimates including 95% confidence intervals (left panel, also see S2 Table). Confidence intervals are also reported for the true cheating rates even though in the roll-a-six game the sample cheating rate can be determined exactly. The confidence intervals reflect the variability in the cheating rates one could expect were one to repeat the experiment.

In the right panel of the figure, the differences between the true rates and the estimates are shown. For the prediction game, all question techniques performed poorly. DQ, UQ and FR all produced estimates below 5% although the true cheating rate was around 25%. The CM comes closest to the true cheating rate with an estimate of a bit more than 15%, but still underestimates the true rate by about 11 percentage points. For the roll-a-six game, we see that DQ and UQ both produced accurate estimates of a cheating rate of about 5%. As expected, cheating was substantially less prevalent in the roll-a-six game (5%) than in the prediction game (25%), due to the design of the game (the roll-a-six game provided less incentive for cheating than the prediction game because it was obvious that cheating could potentially be detected; see [52] for similar results on the effect of detectability on cheating rates). FR significantly underestimated the cheating rate. For CM, on the other hand, an overestimation by about 8 percentage points occurred.

Hence, while for the prediction game the more-is-better assumption seems to be valid in the sense that the highest estimate comes closest to the true value, the assumption fails for the roll-a-six game. Respondents did not substantially underreport their cheating behavior in the roll-a-six game when asked directly, probably because it was obvious that such misreporting could be detected. One could argue that cheating in the roll-a-six game is therefore not a good test case for evaluating sensitive question techniques; there is no bias that could be improved on by the techniques. On the other hand, we would want a valid sensitive question technique to produce unbiased results also if the question is, in fact, not sensitive. A positive bias such as observed for the CM should not occur.

### 3.3 Individual-level validation

**Fig 3:**
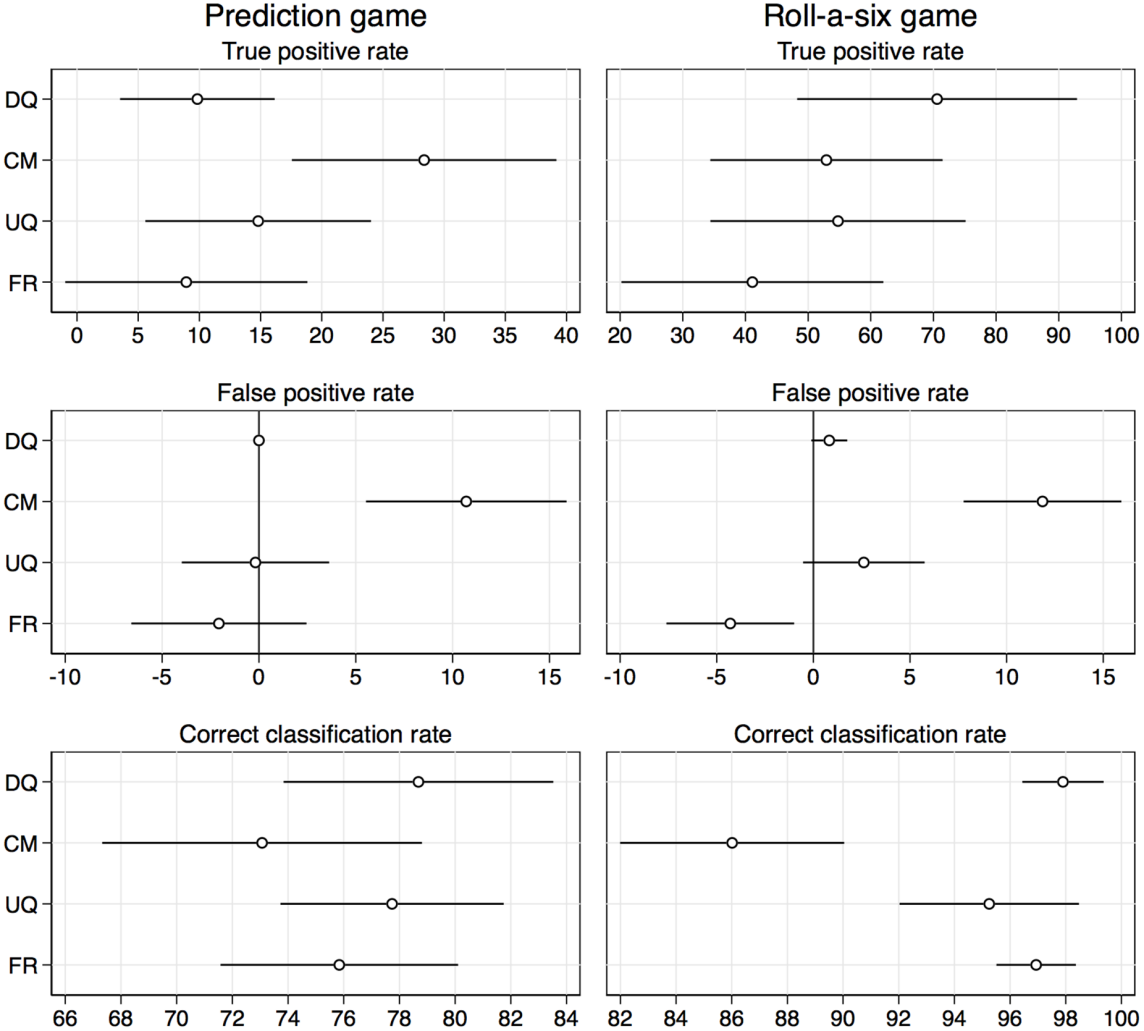
Individual-level validation of sensitive question techniques. Point estimates and 95% confidence intervals in percent. Negative false positive rates were set to zero for the computation of the correct classification rate.

Overall, the results from the aggregate-validation are ambiguous. For the first item, cheating in the prediction game, the crosswise-model RRT (CM) is the clear winner. If we had exclusively looked at the prediction game, we would have again concluded that CM is the most valid technique. However, cheating in the roll-a-six game indicates that there might be a problem with the CM. In the third step of our analysis we therefore evaluate the accuracy of the measurements obtained by the different questioning approaches at the individual level. Fig 3 displays the true and false positive rates of the different techniques for the prediction game and the roll-a-six game (also see S3 Table).

Direct questioning had a true positive rate (TPR) of only 10% in the prediction game, that is, only 10% of respondents who cheated in the prediction game admitted having done so when asked directly. FR did not manage to improve the TPR and UQ slightly increased the TPR to 15% (both differences are not significant; *p* = 0.88 and 0.38). The CM, on the other hand, was considerably more successful in eliciting truthful answers from cheaters than DQ (*p* = 0.004), with a true positive rate of almost 30% (although still being far from 100%). Yet, the CM also had a substantial false positive rate (FPR) of 10.7% (or 8.3% under Assumption A2’). That is to say, about 10% of respondents who did not cheat in the prediction game accidentally admitted having cheated when using the CM. Due to the (relatively) high TPR and the positive FPR the estimate of the cheating rate from the CM came closest to the true cheating rate at the aggregate level (as seen above). However, the correct classification rate (CCR) of the CM was, in fact, worst of all techniques (since about 75% of the respondents did not cheat, the positive FPR has a strong influence on the CCR; *p* = 0.14 compared to DQ; under Assumption A2’ the CCR of CM is a bit higher and comparable the other techniques). The UQ and FR did not have the problem of false positives, but did also not really improve on the TPR compared to DQ, so that these techniques did not reach a better CCR than DQ as well. Overall, for the prediction game, we can therefore conclude that the unrelated-question RRT (UQ) and the forced-response RRT (FR) did not manage to produce more accurate measurements than direct questioning, and that the crosswise-model RRT (CM), although seemingly more valid than direct questioning at the aggregate level, did not yield better measurements either due to the occurrence of false positives.

For the roll-a-six game (right panel in Fig 3) we obtain a similar picture. Also here the CM was affected by a substantial amount of false positives (to a similar degree as in the prediction game; FPR = 11.9%) and, again, although not severely affected by false positives, the UQ and FR did not perform better than direct questioning. For true positives, the ranking of the techniques changed in that direct questioning now performed best, with a true positive rate of about 70% (although the differences to the other techniques are not, or only marginally significant; the *p* values compared to CM, UQ, and FR are 0.23, 0.30, and 0.06, respectively). That the true positive rates for the indirect techniques were lower in this case than for direct questioning might be due to the fact that the RRT, although meant to provide an opportunity to be honest without the risk of disclosure, also provides respondents the possibility to be dishonest without the risk of disclosure. Because it was obvious in the roll-a-six game that a dishonest answer about whether a respondent cheated or not could potentially be identified, some of the respondents who would have felt compelled to answer truthfully in direct questioning might have misused the RRT as a protection mechanism to answer untruthfully without risk of detection. The possibility of such a paradoxical effect of indirect question techniques is also mentioned by Wolter and Preisendörfer [28]. Lelkes et al. [53] found similar adverse effects of complete anonymity on truthful reporting.

To summarize the results for the roll-a-six game: none of the indirect techniques managed to improve the true positive rate compared to direct questioning and the CM was affected by a substantial amount of false positives, so that similar to the prediction game, the correct classification was best for direct questioning and worst for the CM (the difference between DQ and CM now being highly significant, *p* < 0.001).

Our conclusion from the individual-level validation is that none of the tested indirect question techniques yielded an improvement over direct questioning while, at the same time, sacrificing statistical efficiency and hence requiring larger sample sizes than direct questioning. The CM appeared particularly problematic as it was affected by false positives. The occurrence of false positives is the reason why the CM overestimated the cheating rate in the roll-a-six game; it is also the reason why, at the aggregate level, the CM came seemingly closest to the true cheating rate in the prediction game. That the false positive rates of the CM were similar for both games indicates that there was a specific fraction of respondents in our sample who were unable or unwilling to apply the CM procedure correctly. How large this fraction is might depend on the population under study. It is clear, however, that the presence of such noncompliance has strong effects on the estimates obtained by the CM. We suspect that the false positives are the reason for why the CM seemingly performed so well in many previous studies that used a comparative design without the possibility for individual-level validation. False positives inflate the CM estimates and, from a more-is-better perspective, make it look like the CM provides more valid estimates than other techniques.

## 4 Conclusions

In order to evaluate the validity of survey respondents’ self-reports based on various sensitive question techniques we carried out an online experiment in which respondents’ self-reported rates of cheating were compared to true cheating rates. Participants played one of two incentivized dice games in which they could cheat, that is, in which they could illegitimately claim a bonus payment. After the game, participants were asked whether they cheated using either direct questioning or one of several RRT implementations. The resulting self-reports were then validated against the actual rate of cheating in the dice game. Unlike most other evaluation studies of indirect question techniques, our study relies on a true validation criterion and detects misreporting at the individual level.

Results from the two dice games reveal that all tested question techniques suffer sizable misclassification in the direction of the socially desirable answer. Among the different techniques only between 9% and 28% of all cheaters could be correctly classified as cheaters in the first variant of the dice game (prediction game). In the second variant of the dice game (roll-a-six game) between 41% and 71% of cheaters could be correctly classified. The large difference in the true positive rate between the two games suggests that the sensitivity of an item and—possibly, whether answers are potentially verifiable or not—has an important effect on respondents’ decision whether to misreport or not. Although, at least for the prediction game, some of the evaluated indirect question techniques yielded higher true positive rates than direct questioning, none of the techniques produced overall more valid measurements than direct questioning. The reason is that the indirect techniques tend to produce poor results for respondents who do not possess the sensitive trait (i.e. who did not cheat). In particular, a substantial false positive rate was observed for the crosswise-model RRT (CM): for the subsample of non-cheaters, the CM erroneously yielded cheating rates of about 11% or 12%. Furthermore, the forced-response RRT (FR) yielded negative cheating rates in the subsample of non-cheaters, which indicates that some of the respondents did not comply to the RRT instructions and answered “no” even though the procedure instructed them to answer “yes.” The unrelated-question RRT (UQ) had the least problems with respect to misclassification in the subsample of non-cheaters, but it did also not substantially reduce the amount of misclassification in the subsample of cheaters.

The most important insight of our study is that the findings would have been quite different had there not been the possibility for individual-level validation. False positives in the CM inflated the prevalence estimates so that the CM consistently yielded higher prevalence of sensitive behaviors than direct questioning—a result that is in line with previous studies. Hence, employing the more-is-better assumption, the CM seemed superior. As illustrated by the first sensitive item in our study for which validation was possible (cheating in the prediction game), comparing prevalence estimates from indirect question techniques to the true prevalence rate at the aggregate level, although certainly an improvement over the more-is-better assumption, can still be misleading. The CM provided a prevalence estimate that came closest to the true prevalence. Hence, one could again conclude that the CM has superior validity. The analysis at the individual level, however, revealed that this is a false conclusion. The CM came close to the true prevalence primarily because it misclassified some of the non-cheating respondents as cheaters.

That is, our study not only shows that the CM might not be as promising as suggested by previous studies [6, 17–24], it also points to a general weakness in past research on sensitive question techniques. Because complicated misreporting patterns are possible, we must be very cautious when interpreting results from comparative evaluation studies employing the more-is-better assumption, from validation studies that rely on aggregated prevalence validation, or from one-sided validation studies in which the sensitive trait or behavior applies to all or none of the respondents. We argue that an integral evaluation of the performance of a sensitive question technique is only possible if the design is such that false negatives and false positives can be disentangled.

Of course, our study also has limitations. For example, we cannot answer why a substantial share of non-cheaters misreported in the CM. It is noteworthy that such misreporting did not occur with direct questioning. As such, we would speculate the cause might have to do with confusion rather than carelessness. It would be worthwhile to conduct further research on the CM to identify the design feature that causes this type of misreporting and to evaluate possible modifications to address the problem. Possibly, better-designed CM implementations are more robust against the observed problem. Furthermore, although we obtained similar results in both dice games, our study uses a very specific item (cheating for a small amount of money) to evaluate the sensitive question techniques and, in addition, has been conducted in a special setting and in a special population (an online survey on Amazon Mechanical Turk). Whether our results can be generalized to other sensitive questions, and to other populations and settings remains questionable. However, note that our finding of false positives in the CM has recently been replicated with a different design and in a different type of sample in an online survey on organ donation [54]. Finally, we only evaluated three specific variants of the randomized response technique. Although the results of our study are discouraging for all three variants, there might be alternative designs or implementations that are more successful. Future research should focus on evaluating such alternatives. Using a research design that allows individual-level validation of respondents’ answers, however, would be crucial for such research to be meaningful.

## Supporting information

S1 Table Prevalence estimates by sensitive question technique as displayed in Fig 1. (DOCX)

S2 Table Cheating rates in the prediction game and the roll-a-six game as displayed in Fig 2. (DOCX)

S3 Table Individual-level validation results in the prediction game and the roll-a-six game as displayed in Fig 3. (DOCX)

S1 Supporting Information Analysis script and supplementary results. (PDF)

S1 Documentation Documentation and codebook of the survey. (PDF)

S1 Dataset Dataset of the survey (Stata 13 format). (ZIP)
