# Supplementary material for: More is not always better: An experimental individual-level validation of the randomized response technique and the crosswise model
Source: PLoS One. 2018 Aug 14;13(8):e0201770. doi: 10.1371/journal.pone.0201770 (PMC6091935; doi:10.1371/journal.pone.0201770)
Supplement: S1 Table — (DOCX) [file pone.0201770.s001.docx]

**S1 Table. Prevalence estimates by sensitive question technique as displayed in Fig 1.**

|  | Shoplifting (*N* = 6,136) | Tax evasion (*N* = 6,136) | Non-voting (N = 6,131) | Cheating in the prediction game (*N* = 3,065) | Cheating in the roll-a-six game (*N* = 3,070) |
| --- | --- | --- | --- | --- | --- |
| Direct questioning (DQ) | 40.23 (1.77) | 10.03 (1.08) | 34.46 (1.72) | 2.33 (0.77) | 3.94 (1.00) |
| Crosswise-model RRT (CM) | 46.42 (1.62) | 19.52 (1.50) | 38.11 (1.61) | 15.41 (2.05) | 14.34 (2.06) |
| Unrelated-question RRT (UQ) | 54.53 (1.64) | 17.63 (1.42) | 34.74 (1.60) | 3.74 (1.63) | 5.23 (1.66) |
| Forced-response RRT (FR) | 49.22 (1.71) | 14.30 (1.52) | 32.51 (1.68) | 0.85 (1.83) | –1.94 (1.73) |
| Differences: |  |  |  |  |  |
| CM – DQ | 6.18 (2.40) | 9.50 (1.85) | 3.64 (2.35) | 13.08 (2.19) | 10.40 (2.29) |
| UQ – DQ | 14.30 (2.41) | 7.60 (1.78) | 0.28 (2.34) | 1.41 (1.80) | 1.29 (1.94) |
| FR – DQ | 8.99 (2.46) | 4.27 (1.87) | –1.95 (2.40) | –1.47 (1.99) | –5.87 (2.00) |

In percent. Standard errors in parentheses.
