# Supplementary material for: More is not always better: An experimental individual-level validation of the randomized response technique and the crosswise model
Source: PLoS One. 2018 Aug 14;13(8):e0201770. doi: 10.1371/journal.pone.0201770 (PMC6091935; doi:10.1371/journal.pone.0201770)
Supplement: S2 Table — (DOCX) [file pone.0201770.s002.docx]

**S2 Table. Cheating rates in the prediction game and the roll-a-six game as displayed in Fig 2.**

|  | Prediction game (*N* = 3,065) | | | Roll-a-six game (*N* = 3,070) | | |
| --- | --- | --- | --- | --- | --- | --- |
|  | observed | estimated | difference | observed | estimated | difference |
| Direct questioning (DQ) | 23.64 (2.50) | 2.33 (0.77) | –21.32 (2.47) | 4.46 (1.06) | 3.94 (1.00) | –0.52 (0.74) |
| Crosswise-model RRT (CM) | 26.63 (1.45) | 15.41 (2.05) | –11.22 (2.42) | 6.04 (0.71) | 14.34 (2.06) | 8.30 (2.08) |
| Unrelated-question RRT (UQ) | 26.13 (1.80) | 3.74 (1.63) | –22.40 (2.30) | 5.01 (0.78) | 5.23 (1.66) | 0.21 (1.65) |
| Forced-response RRT (FR) | 26.53 (1.80) | 0.85 (1.83) | –25.68 (2.48) | 5.20 (0.80) | –1.94 (1.73) | –7.14 (1.74) |

In percent. Standard errors in parentheses.
