# Supplementary material for: More is not always better: An experimental individual-level validation of the randomized response technique and the crosswise model
Source: PLoS One. 2018 Aug 14;13(8):e0201770. doi: 10.1371/journal.pone.0201770 (PMC6091935; doi:10.1371/journal.pone.0201770)
Supplement: S3 Table — (DOCX) [file pone.0201770.s003.docx]

**S3 Table. Individual-level validation results in the prediction game and the roll-a-six game as displayed in Fig 3.**

|  | Prediction game (*N* = 3,065) | | | Roll-a-six game (*N* = 3,070) | | |
| --- | --- | --- | --- | --- | --- | --- |
|  | TPR | FPR | CCR | TPR | FPR | CCR |
| Direct questioning (DQ) | 9.84 (3.22) | 0.00 (0.00) | 78.68 (2.47) | 70.59 (11.39) | 0.82 (0.47) | 97.90 (0.75) |
| Crosswise-model RRT (CM) | 28.36 (5.52) | 10.71 (2.64) | 73.07 (2.93) | 52.93 (9.46) | 11.86 (2.08) | 86.01 (2.05) |
| Unrelated-question RRT (UQ) | 14.80 (4.70) | –0.18 (1.94) | 77.74 (2.05) | 54.77 (10.39) | 2.61 (1.60) | 95.25 (1.64) |
| Forced-response RRT (FR) | 8.93 (5.05) | –2.07 (2.31) | 75.84 (2.18) | 41.11 (10.66) | –4.30 (1.69) | 96.94 (0.73) |

In percent. Standard errors in parentheses. TPR = true positive rate, FPR = false positive rate, CCR = correct classification rate (negative false positive rates were set to zero for the computation of CCR).
