# Supplementary material for: More is not always better: An experimental individual-level validation of the randomized response technique and the crosswise model
Source: PLoS One. 2018 Aug 14;13(8):e0201770. doi: 10.1371/journal.pone.0201770 (PMC6091935; doi:10.1371/journal.pone.0201770)
Supplement: S1 Supporting Information — (PDF) [file pone.0201770.s004.pdf]

Analysis script and supplementary results for:

Marc Höglinger, Ben Jann. More Is Not Always Better: An Experimental Individual-Level Validation of the Randomized Response Technique and the Crosswise Model.

Software: Stata/MP 15.1 (required user packages: `fre`, `estout`, `grstyle`, `palettes`)

Contents

1 Setup 2

2 Sample selection and descriptive statistics 2

2.1 Response rates . . . 2
2.2 Create dummies for experimental conditions . . . 2
2.3 Sample selection . . . 3
2.4 Response time . . . 5
2.5 Descriptive statistics (Table 1) . . . 6
2.5.1 Sex . . . 6
2.5.2 Age . . . 6
2.5.3 Education . . . 7
2.5.4 Employment status . . . 10
2.5.5 Prior exposure to MTurk studies . . . 11
2.5.6 Location at time of interview . . . 13
2.6 Number of observations per technique, item, and game (Table 3) . . . 14

3 Main analysis 15

3.1 Prepare sensitive items . . . 15
3.2 Comparative validation (Fig 1/S1 Table) . . . 15
3.3 Aggregate-level validation (Fig 2/S2 Table) . . . 19
3.4 Individual-level validation . . . 22
3.4.1 Prediction game . . . 22
3.4.2 Roll-a-six game . . . 25
3.4.3 Overview of results (Fig 3/S3 Table) . . . 29

4 Discussion of assumption A2 30

4.1 False positive rates for true losers and true winners in the roll-a-six game . . . 30
4.2 Results for the prediction game assuming the false positive rate among true winners to be zero 31

Produced by `texdoc`, 14jul2018

1 Setup

```
. // load data
. use ASQ-MTurk-2013.dta
(MTurk Survey on "Mood and Personality" 2013)
. // settings for bootstrap
. set seed 863991
. global reps 1000
. // graph settings
. set scheme s1mono
. grstyle init
. grstyle set plain, box grid
. grstyle set margin zero
. grstyle set color black, p(1/15)
. grstyle set color white, p(1/15): p#markfill
```

2 Sample selection and descriptive statistics

2.1 Response rates

```
. mat R = J(5,2,.)
. mat coln R = "N" "RR"
. mat rown R = "Sample" "Started2" "Completed" "Completed2" "SQ"
. qui count
. mat R[1,1] = r(N), 1
. qui count if submit2!=.b      // submitted first page with questions
. mat R[2,1] = r(N), r(N)/R[1,1]
. qui count if completed==1
. mat R[3,1] = r(N), r(N)/R[1,1]
. qui count if submit21!=.b     // submitted last page with questions (before resp' comments)
. mat R[4,1] = r(N), r(N)/R[1,1]
. qui count if senstec<.        // reached first page after sensitive question intro
. mat R[5,1] = r(N), r(N)/R[1,1]
. // Sample:     Gross sample = Accepted HIT & started survey
. // Started2:   Submitted at least the first page containing questions
. // Completed:  Completed questionnaire to very end
. // Completed2: Completed the questionnaire to last page containing questions
. // SQ:         Submitted at least sensitive questions introduction page
. mat list R
R[5,2]
                   N         RR
    Sample      6505          1
  Started2      6495  .99846272
 Completed      6461  .99323597
Completed2      6461  .99323597
        SQ      6473  .99508071
```

2.2 Create dummies for experimental conditions

```
. fre senstec
senstec — Sensitive question technique assignment
```

	Freq.	Percent	Valid	Cum.

Valid 1 DQ	810	12.45	12.51	12.51
2 CMquest	2438	37.48	37.66	50.18
3 UQbenf	1618	24.87	25.00	75.17
4 FRnumb	1607	24.70	24.83	100.00
Total	6473	99.51	100.00	
Missing .b break-off	32	0.49		
Total	6505	100.00		

```
. gen byte DQ = senstec==1 if senstec<.
(32 missing values generated)
. gen byte CM = senstec==2 if senstec<.
(32 missing values generated)
. gen byte UQ = senstec==3 if senstec<.
(32 missing values generated)
. gen byte FR = senstec==4 if senstec<.
(32 missing values generated)
. fre DQ CM UQ FR
```

DQ

	Freq.	Percent	Valid	Cum.
Valid 0	5663	87.06	87.49	87.49
1	810	12.45	12.51	100.00
Total	6473	99.51	100.00	
Missing .	32	0.49		
Total	6505	100.00		

CM

	Freq.	Percent	Valid	Cum.
Valid 0	4035	62.03	62.34	62.34
1	2438	37.48	37.66	100.00
Total	6473	99.51	100.00	
Missing .	32	0.49		
Total	6505	100.00		

UQ

	Freq.	Percent	Valid	Cum.
Valid 0	4855	74.63	75.00	75.00
1	1618	24.87	25.00	100.00
Total	6473	99.51	100.00	
Missing .	32	0.49		
Total	6505	100.00		

FR

	Freq.	Percent	Valid	Cum.
Valid 0	4866	74.80	75.17	75.17
1	1607	24.70	24.83	100.00
Total	6473	99.51	100.00	
Missing .	32	0.49		
Total	6505	100.00		

2.3 Sample selection

```
. // exclude respondents who did not reach first page of assigned sensitive question technique
. keep if senstec<.
(32 observations deleted)
```

```
. // screening question
. gen str tmp = lower(stritrim(strtrim(q2txt)))
(201 missing values generated)

. replace tmp = subinstr(tmp, `"""', "", .)
(12 real changes made)

. replace tmp = subinstr(tmp, `"."', "", .)
(9 real changes made)

. replace tmp = subinstr(tmp, `"!"', "", .)
(8 real changes made)

. replace tmp = subinstr(tmp, `"'"', "", .)
(72 real changes made)

. replace tmp = subinstr(tmp, `"’"', "", .)
(11 real changes made)

. replace tmp = subinstr(tmp, `"‘"', "", .)
(10 real changes made)

. gen byte passed = tmp=="got it"

. foreach s in  "get it" "git it" "go it" "god it" "gor it" "got" "got lt" "got ot" "got t" {
  2.     replace passed = 1 if tmp==`"`s'"'
  3. }
(1 real change made)
(4 real changes made)
(8 real changes made)
(1 real change made)
(1 real change made)
(3 real changes made)
(1 real change made)
(2 real changes made)
(1 real change made)

. fre tmp if passed==0
```

tmp

		Freq.	Percent	Valid	Cum.
Valid	mood	1	0.49	25.00	25.00
	mood and personality	1	0.49	25.00	50.00
	paying attention	1	0.49	25.00	75.00
	personality	1	0.49	25.00	100.00
	Total	4	1.95	100.00	
Missing		201	98.05		
Total		205	100.00		

```
. fre tmp if passed==1
```

tmp

		Freq.	Percent	Valid	Cum.
Valid	get it	1	0.02	0.02	0.02
	git it	4	0.06	0.06	0.08
	go it	8	0.13	0.13	0.21
	god it	1	0.02	0.02	0.22
	gor it	1	0.02	0.02	0.24
	got	3	0.05	0.05	0.29
	got it	6246	99.65	99.65	99.94
	got lt	1	0.02	0.02	99.95
	got ot	2	0.03	0.03	99.98
	got t	1	0.02	0.02	100.00
	Total	6268	100.00	100.00	

```
. fre passed
```

passed

		Freq.	Percent	Valid	Cum.
Valid	0	205	3.17	3.17	3.17

```
           1 |       6268      96.83      96.83     100.00
       Total |       6473     100.00     100.00
-----------------------------------------------------------

. drop tmp

. keep if passed
(205 observations deleted)

. drop passed

. // exclude respondents for whom no dice roll was recorded (could be because the
. // die was not/could not be rolled or because there was a recording error)
. gen byte rollrec = q6_rollcount!=0 if q6_rollcount<.

. fre rollrec

rollrec
-----------------------------------------------------------
             |      Freq.    Percent      Valid       Cum.
-------------+---------------------------------------------
Valid   0    |        115       1.83       1.83       1.83
        1    |       6153      98.17      98.17     100.00
        Total|       6268     100.00     100.00
-----------------------------------------------------------

. keep if rollrec
(115 observations deleted)

. drop rollrec

. // exclude one roll-a-six participant with predefined six not claiming his legitimate bonus
. fre q6_roll1 if dicegame==2

q6_roll1 — Predefined outcome roll 1
-----------------------------------------------------------
             |      Freq.    Percent      Valid       Cum.
-------------+---------------------------------------------
Valid   1    |        515      16.73      16.73      16.73
        2    |        547      17.77      17.77      34.49
        3    |        516      16.76      16.76      51.25
        4    |        498      16.17      16.17      67.42
        5    |        487      15.82      15.82      83.24
        6    |        516      16.76      16.76     100.00
        Total|       3079     100.00     100.00
-----------------------------------------------------------

. drop if q6==0 & q6_roll1==6 & dicegame==2
(1 observation deleted)

. // number of remaining observations
. count
  6,152
```

2.4 Response time

```
. gen duration = seconds(enddate - startdate)

. su duration, detail

                          duration
-------------------------------------------------------------
      Percentiles      Smallest
 1%          181            118
 5%          227            119
10%          261            124       Obs               6,152
25%          321            130       Sum of Wgt.       6,152

50%          401                      Mean           448.1977
                        Largest       Std. Dev.      236.0526
75%          515           3164
90%          662           4556       Variance       55720.83
95%          791           5128       Skewness       6.201485
99%         1256           5500       Kurtosis        91.2505

. di r(p50)/60
6.6833333
```

```
. drop duration
```

2.5 Descriptive statistics (Table 1)

2.5.1 Sex

```
. fre q9_3
```

q9_3 — Gender

		Freq.	Percent	Valid	Cum.
Valid	1 Male	3066	49.84	49.91	49.91
	2 Female	3077	50.02	50.09	100.00
	Total	6143	99.85	100.00	
Missing	.a no answer	9	0.15		
Total		6152	100.00		

2.5.2 Age

```
. fre q9_3
```

q9_3 — Gender

		Freq.	Percent	Valid	Cum.
Valid	1 Male	3066	49.84	49.91	49.91
	2 Female	3077	50.02	50.09	100.00
	Total	6143	99.85	100.00	
Missing	.a no answer	9	0.15		
Total		6152	100.00		

```
. gen     age = q9_2 if q9_2<100
(6,133 missing values generated)

. replace age = 2013 - q9_2 + 1 if q9_2>1900
(6,130 real changes made)

. replace age = 2013 - 1972 + 1 if q9_2==172
(1 real change made)

. replace age = 2013 - 1994 + 1 if q9_2==194
(1 real change made)

. replace age = 2013 - 1960 + 1 if q9_2==1060
(1 real change made)

. compress age
  variable age was double now byte
  (43,064 bytes saved)

. fre age
```

age

		Freq.	Percent	Valid	Cum.
Valid	18	4	0.07	0.07	0.07
	19	81	1.32	1.32	1.38
	20	171	2.78	2.78	4.16
	21	223	3.62	3.62	7.79
	22	296	4.81	4.81	12.60
	23	326	5.30	5.30	17.90
	24	394	6.40	6.40	24.30
	25	364	5.92	5.92	30.22
	26	343	5.58	5.58	35.79
	27	343	5.58	5.58	41.37
	28	314	5.10	5.10	46.47
	29	298	4.84	4.84	51.32

```
       30 |        263       4.28       4.28      55.59
       31 |        240       3.90       3.90      59.49
       32 |        223       3.62       3.62      63.12
       33 |        203       3.30       3.30      66.42
       34 |        209       3.40       3.40      69.81
       35 |        170       2.76       2.76      72.58
       36 |        153       2.49       2.49      75.07
       37 |        130       2.11       2.11      77.18
       :  |          :          :          :          :
       59 |         31       0.50       0.50      97.06
       60 |         31       0.50       0.50      97.56
       61 |         20       0.33       0.33      97.89
       62 |         27       0.44       0.44      98.33
       63 |         13       0.21       0.21      98.54
       64 |         12       0.20       0.20      98.73
       65 |         10       0.16       0.16      98.89
       66 |         14       0.23       0.23      99.12
       67 |         15       0.24       0.24      99.37
       68 |         10       0.16       0.16      99.53
       69 |          7       0.11       0.11      99.64
       70 |          5       0.08       0.08      99.72
       71 |          4       0.07       0.07      99.79
       72 |          1       0.02       0.02      99.80
       73 |          5       0.08       0.08      99.89
       74 |          2       0.03       0.03      99.92
       75 |          1       0.02       0.02      99.93
       76 |          1       0.02       0.02      99.95
       77 |          2       0.03       0.03      99.98
       82 |          1       0.02       0.02     100.00
       Total |     6152     100.00     100.00
------------------------------------------------------------

. su age, detail
                              age
-------------------------------------------------------------
      Percentiles      Smallest
 1%           19             18
 5%           21             18
10%           22             18       Obs               6,152
25%           25             18       Sum of Wgt.       6,152
50%           29                      Mean            32.2934
                        Largest       Std. Dev.       10.6223
75%           36             76
90%           49             77       Variance       112.8333
95%           56             77       Skewness       1.346843
99%           66             82       Kurtosis       4.496818
. egen agecat = cut(age), at(18, 25, 30, 35, 40, 50, 100)
. fre agecat
agecat
------------------------------------------------------------
              |      Freq.    Percent      Valid       Cum.
--------------+---------------------------------------------
Valid   18    |       1495      24.30      24.30      24.30
        25    |       1662      27.02      27.02      51.32
        30    |       1138      18.50      18.50      69.81
        35    |        660      10.73      10.73      80.54
        40    |        624      10.14      10.14      90.69
        50    |        573       9.31       9.31     100.00
        Total |       6152     100.00     100.00
------------------------------------------------------------

. drop agecat
```

2.5.3 Education

```
. replace q9_1=2 if q9_1txt=="GED" // General educational development test, similar to highschool
```

```
(9 real changes made)
. replace q9_1=6 if q9_1txt=="JD" // Juris Doctor, professional doctorate
(3 real changes made)
. replace q9_1=2 if q9_1txt=="Trade school"
(1 real change made)
. replace q9_1=3 if q9_1txt=="college diploma"
(1 real change made)
. replace q9_1=1 if q9_1txt=="Currently in High School"
(1 real change made)
. replace q9_1=6 if q9_1txt=="M.D" // Medicinae Doctor
(1 real change made)
. replace q9_1=7 if q9_1txt=="culinary school graduate"
(0 real changes made)
. replace q9_1=3 if q9_1txt=="currently studying for bachelor's"
(1 real change made)
. replace q9_1=5 if q9_1txt=="BS, RCP,(Respiratory Care Practitioner)"
(1 real change made)
. replace q9_1=2 if q9_1txt=="Trade School"
(1 real change made)
. replace q9_1=5 if q9_1txt=="Master's degree in progress"
(1 real change made)
. replace q9_1=6 if q9_1txt=="jd"
(1 real change made)
. replace q9_1=7 if q9_1txt=="37 Certificates in Information Technologies"
(0 real changes made)
. replace q9_1=6 if q9_1txt=="MD"
(2 real changes made)
. replace q9_1=6 if q9_1txt=="J.D."
(2 real changes made)
. replace q9_1=2 if q9_1txt=="trade school"
(2 real changes made)
. replace q9_1=5 if q9_1txt=="Undergraduate/Student"
(1 real change made)
. replace q9_1=7 if q9_1txt=="Ed.S"
(0 real changes made)
. replace q9_1=3 if q9_1txt=="Still attending college-Junior"
(1 real change made)
. replace q9_1=7 if q9_1txt=="Tech School"
(0 real changes made)
. replace q9_1=7 if q9_1txt=="Current student"
(0 real changes made)
. replace q9_1=2 if q9_1txt=="Vocational school"
(0 real changes made)
. replace q9_1=3 if q9_1txt=="Student in college"
(1 real change made)
. replace q9_1=5 if q9_1txt=="Current Graduate Student"
(1 real change made)
. replace q9_1=2 if q9_1txt=="medical assistant certification"
(1 real change made)
. replace q9_1=5 if q9_1txt=="Some graduate school"
(2 real changes made)
. replace q9_1=5 if q9_1txt=="Some Master's-Speech Pathology"
(1 real change made)
. replace q9_1=5 if q9_1txt=="Some graduate school"
(0 real changes made)
. replace q9_1=7 if q9_1txt=="Student"
(0 real changes made)
. replace q9_1=2 if q9_1txt=="Vocational Certificate after high school diploma"
(0 real changes made)
```

```
. replace q9_1=7 if q9_1txt=="State Board Cosmetology license"
(0 real changes made)

. replace q9_1=2 if q9_1txt=="some high school , GED"
(1 real change made)

. replace q9_1=6 if q9_1txt=="professional degree J.D."
(1 real change made)

. replace q9_1=7 if q9_1txt=="tech school"
(0 real changes made)

. replace q9_1=3 if q9_1txt=="Completing bachelor's"
(1 real change made)

. replace q9_1=2 if q9_1txt=="AT-CTI program for Air Traffic Control"
(1 real change made)

. replace q9_1=2 if q9_1txt=="Certificate of Completion (trade)"
(1 real change made)

. replace q9_1=3 if q9_1txt=="Currently in College"
(1 real change made)

. replace q9_1=3 if q9_1txt=="In college- BA History"
(1 real change made)

. replace q9_1=3 if q9_1txt=="Current undergrad (senior) completing BS and BA"
(1 real change made)

. replace q9_1=6 if q9_1txt=="Pharm.D"
(1 real change made)

. replace q9_1=6 if q9_1txt=="MD"
(0 real changes made)

. replace q9_1=7 if q9_1txt=="Technical training, HS deploma"
(0 real changes made)

. replace q9_1=5 if q9_1txt=="enrolled in master's program"
(1 real change made)

. replace q9_1=3 if q9_1txt=="In college now for my Bachelor's"
(1 real change made)

. replace q9_1=2 if q9_1txt=="Vocational degree - LPN"
(1 real change made)

. replace q9_1=7 if q9_1txt=="Certificate from cosmetology"
(0 real changes made)

. replace q9_1=3 if q9_1txt=="currently working on my Associate"
(1 real change made)

. replace q9_1=2 if q9_1txt=="trade school"
(0 real changes made)

. replace q9_1=7 if q9_1txt=="tech school - completed"
(0 real changes made)

. replace q9_1=3 if q9_1txt=="Still in college"
(1 real change made)

. replace q9_1=3 if q9_1txt=="trade school certificate and some college"
(1 real change made)

. replace q9_1=7 if q9_1txt=="technical school"
(0 real changes made)

. replace q9_1=3 if q9_1txt=="College"
(0 real changes made)

. replace q9_1=2 if q9_1txt=="Vocational Degree"
(1 real change made)

. replace q9_1=3 if q9_1txt=="currently in college"
(1 real change made)

. replace q9_1=7 if q9_1txt=="EMT-B Licensed"
(0 real changes made)

. replace q9_1=2 if q9_1txt=="Vocational/Certification"
(1 real change made)

. replace q9_1=2 if q9_1txt=="G.E.D."
(1 real change made)
```

```
. replace q9_1=2 if q9_1txt=="GED, overall average 656"
(0 real changes made)

. replace q9_1=3 if q9_1txt=="College, on going"
(1 real change made)

. gen byte educ = 1 if inlist(q9_1, 4, 5, 6)
(2,835 missing values generated)

. replace  educ = 2 if inlist(q9_1, 3)
(2,103 real changes made)

. replace  educ = 3 if inlist(q9_1, 1, 2, 7)
(728 real changes made)

. lab def  educ 1 "college degree" 2 "some college" 3 "high school or other"

. lab val  educ educ

. fre educ

educ
```

		Freq.	Percent	Valid	Cum.
Valid	1 college degree	3317	53.92	53.95	53.95
	2 some college	2103	34.18	34.21	88.16
	3 high school or other	728	11.83	11.84	100.00
	Total	6148	99.93	100.00	
Missing	.	4	0.07		
Total		6152	100.00		

2.5.4 Employment status

```
. d q10_2*

              storage   display    value
variable name   type    format     label      variable label
------------------------------------------------------------------------------------------------
q10_2_1         byte    %12.0g     q10_2_1    Currently employed
q10_2_2         byte    %12.0g     q10_2_2    Currently self-employed
q10_2_3         byte    %12.0g     q10_2_3    Currently out of work and looking for work
q10_2_4         byte    %12.0g     q10_2_4    Currently homemaker
q10_2_5         byte    %12.0g     q10_2_5    Currently student
q10_2_6         byte    %12.0g     q10_2_6    Currently retired
q10_2_7         byte    %12.0g     q10_2_7    Currently other
q10_2_7txt      str108  %9s                   Currently other (text)

. gen byte lfp = .
(6,152 missing values generated)

. replace lfp = 1 if q10_2_1==1 & lfp==.      // employed
(3,321 real changes made)

. replace lfp = 2 if q10_2_2==1 & lfp==.      // selfemployed
(777 real changes made)

. replace lfp = 3 if q10_2_3==1 & lfp==.      // unemployed
(695 real changes made)

. replace lfp = 4 if q10_2_5==1 & lfp==.      // student
(800 real changes made)

. replace lfp = 5 if (q10_2_4==1 | q10_2_6==1 | q10_2_7==1) & lfp==.   // other
(546 real changes made)

. lab def lfp 1 "employed" 2 "self-employed" 3 "unemployed" 4 "student" 5 "other"

. lab val lfp lfp

. fre lfp

lfp
```

		Freq.	Percent	Valid	Cum.
Valid	1 employed	3321	53.98	54.10	54.10
	2 self-employed	777	12.63	12.66	66.75

3 unemployed	695	11.30	11.32	78.07
4 student	800	13.00	13.03	91.11
5 other	546	8.88	8.89	100.00
Total	6139	99.79	100.00	
Missing .	13	0.21		
Total	6152	100.00		

2.5.5 Prior exposure to MTurk studies

```
. replace q10_1 = lower(q10_1)
(15 real changes made)
. replace q10_1 = subinstr(q10_1, ",", "", 1)
(12 real changes made)
. replace q10_1 = subinstr(q10_1, "~", "", 1)
(23 real changes made)
. replace q10_1 = subinstr(q10_1, ">", "", 1)
(12 real changes made)
. replace q10_1 = subinstr(q10_1, "<", "", 1)
(3 real changes made)
. replace q10_1 = subinstr(q10_1, "?", "", 1)
(15 real changes made)
. replace q10_1 = subinstr(q10_1, "+", "", 1)
(90 real changes made)
. replace q10_1 = subinstr(q10_1, "over ", "", 1)
(4 real changes made)
. replace q10_1 = subinstr(q10_1, " times", "", 1)
(6 real changes made)
. replace q10_1 = subinstr(q10_1, "about ", "", 1)
(4 real changes made)
. replace q10_1 = subinstr(q10_1, "maybe around ", "", 1)
(1 real change made)
. replace q10_1 = subinstr(q10_1, "around ", "", 1)
(1 real change made)
. replace q10_1 = subinstr(q10_1, "approx ", "", 1)
(1 real change made)
. replace q10_1 = subinstr(q10_1, "approx. ", "", 1)
(1 real change made)
. replace q10_1 = subinstr(q10_1, "approximately ", "", 1)
(1 real change made)
. replace q10_1 = subinstr(q10_1, " i think", "", 1)
(1 real change made)
. replace q10_1 = subinstr(q10_1, " or more", "", 1)
(2 real changes made)
. replace q10_1 ="10"     if q10_1=="10-11"
(1 real change made)
. replace q10_1 ="12"     if q10_1=="10-15"
(1 real change made)
. replace q10_1 ="15"     if q10_1=="10-20"
(1 real change made)
. replace q10_1 ="100"    if q10_1==`"100"s"'
(1 real change made)
. replace q10_1 ="100"    if q10_1=="100s"
(2 real changes made)
. replace q10_1 ="17"     if q10_1=="15-20"
(2 real changes made)
. replace q10_1 ="22"     if q10_1=="15-30"
(1 real change made)
```

```
. replace q10_1 ="3"      if q10_1=="2-5"
(1 real change made)

. replace q10_1 ="27"     if q10_1=="25-30"
(1 real change made)

. replace q10_1 ="3"      if q10_1=="3-4"
(2 real changes made)

. replace q10_1 ="4"      if q10_1=="3-5"
(2 real changes made)

. replace q10_1 ="40"     if q10_1=="30-50"
(1 real change made)

. replace q10_1 ="40"     if q10_1=="40 or so"
(1 real change made)

. replace q10_1 ="47"     if q10_1=="45-50"
(1 real change made)

. replace q10_1 ="7"      if q10_1=="5-10"
(2 real changes made)

. replace q10_1 ="75"     if q10_1=="50-100"
(1 real change made)

. replace q10_1 ="50"     if q10_1=="50x"
(1 real change made)

. replace q10_1 ="80"     if q10_1=="80ish"
(1 real change made)

. replace q10_1 ="978"    if q10_1=="978 approved hits"
(1 real change made)

. replace q10_1 =""       if q10_1=="a lot"
(1 real change made)

. replace q10_1 =""       if q10_1=="alot"
(1 real change made)

. replace q10_1 =""       if q10_1=="couple"
(1 real change made)

. replace q10_1 =""       if q10_1=="don't know"
(2 real changes made)

. replace q10_1 =""       if q10_1=="dont know"
(1 real change made)

. replace q10_1 ="100"    if q10_1=="hundreds"
(1 real change made)

. replace q10_1 =""       if q10_1=="i don't know.  this isn't the first one though."
(1 real change made)

. replace q10_1 =""       if q10_1=="lots"
(2 real changes made)

. replace q10_1 =""       if q10_1=="lots -- cannot count"
(1 real change made)

. replace q10_1 =""       if q10_1=="lots!"
(1 real change made)

. replace q10_1 =""       if q10_1=="many"
(4 real changes made)

. replace q10_1 ="0"      if q10_1=="no"
(2 real changes made)

. replace q10_1 ="0"      if q10_1=="none"
(1 real change made)

. replace q10_1 =""       if q10_1=="not sure"
(6 real changes made)

. replace q10_1 ="20"     if q10_1=="not sure. 20 maybe"
(1 real change made)

. replace q10_1 =""       if q10_1=="numerous"
(1 real change made)

. replace q10_1 =""       if q10_1=="often"
(1 real change made)

. replace q10_1 ="1"      if q10_1=="one other study"
```

```
(1 real change made)
. replace q10_1 =""       if q10_1=="several"
(1 real change made)
. replace q10_1 =""       if q10_1=="started this week"
(1 real change made)
. replace q10_1 ="1000"   if q10_1=="thousands"
(1 real change made)
. destring q10_1, replace
q10_1: all characters numeric; replaced as long
(45 missing values generated)
. su q10_1, detail
              Number of MTurk studies attended
-------------------------------------------------------------
      Percentiles      Smallest
 1%            0              0
 5%            0              0
10%            1              0       Obs               6,107
25%            7              0       Sum of Wgt.       6,107

50%           50                      Mean           547.4488
                        Largest       Std. Dev.      4349.993
75%          250          60000
90%         1000          90000       Variance       1.89e+07
95%         2000         150557       Skewness       38.34398
99%         7100         244001       Kurtosis       1882.163
. egen exposure = cut(q10_1), at(0, 1, 10, 100, 1000, 250000)
(45 missing values generated)
. compress exposure
  variable exposure was double now int
  (36,912 bytes saved)
. fre exposure
```

exposure

		Freq.	Percent	Valid	Cum.
Valid	0	413	6.71	6.76	6.76
	1	1181	19.20	19.34	26.10
	10	2008	32.64	32.88	58.98
	100	1847	30.02	30.24	89.23
	1000	658	10.70	10.77	100.00
	Total	6107	99.27	100.00	
Missing	.	45	0.73		
Total		6152	100.00		

2.5.6 Location at time of interview

```
. fre q10_3
```

q10_3 — Actual location: categorized

		Freq.	Percent	Valid	Cum.
Valid	1 at home	5251	85.35	85.38	85.38
	2 at workplace/office	607	9.87	9.87	95.25
	3 in a cafe/restaurant	41	0.67	0.67	95.92
	4 at school/university	147	2.39	2.39	98.31
	5 travelling	10	0.16	0.16	98.47
	6 other (please specify)	94	1.53	1.53	100.00
	Total	6150	99.97	100.00	
Missing	.a no answer	2	0.03		
Total		6152	100.00		

```
. gen byte location = 1 if q10_3==1
```

```
(901 missing values generated)
. replace  location = 2 if q10_3==2
(607 real changes made)
. replace  location = 3 if inlist(q10_3,3,4,5,6)
(292 real changes made)
. lab def  location 1 "at home" 2 "at workplace" 3 "other"
. lab val  location location
. fre location
```

location

		Freq.	Percent	Valid	Cum.
Valid	1 at home	5251	85.35	85.38	85.38
	2 at workplace	607	9.87	9.87	95.25
	3 other	292	4.75	4.75	100.00
	Total	6150	99.97	100.00	
Missing	.	2	0.03		
Total		6152	100.00		

2.6 Number of observations per technique, item, and game (Table 3)

```
. // overview
. lab def senstec 2 "CM" 3 "UQ" 4 "FR", modify
. table dicegame senstec, c(freq) row col
```

Dice game assignment	Sensitive question technique assignment				
	DQ	CM	UQ	FR	Total
prediction	387	1,168	760	759	3,074
roll-a-six	382	1,145	780	771	3,078
Total	769	2,313	1,540	1,530	6,152

```
. // by sensitive question
. qui estpost tabstat q14 q15 q16 q17, s(count) by(senstec) columns(statistics)
. esttab, cell(count) unstack collab(none) nonumb nomti noobs compress ///
>     title("N by sensitive item and technique")
```

N by sensitive item and technique

	DQ	CM	UQ	FR	Total
q14	768	2313	1534	1521	6136
q15	768	2310	1532	1526	6136
q16	766	2306	1534	1525	6131
q17	768	2306	1535	1526	6135

```
. // item nonresponse
. foreach v in q14 q15 q16 q17 {
  2.     gen _mis_`v' = (`v'>=.)*100
  3. }
. qui estpost tabstat _mis_*, by(senstec) columns(statistics)
. estout, cell(mean(f(%9.2f))) unstack collab(none) mlab(none) ///
>     title("Item nonresponse (percent)")
```

Item nonresponse (percent)

	DQ	CM	UQ	FR	Total
_mis_q14	0.13	0.00	0.39	0.59	0.26
_mis_q15	0.13	0.13	0.52	0.26	0.26

```
_mis_q16             0.39          0.30          0.39          0.33          0.34
_mis_q17             0.13          0.30          0.32          0.26          0.28
----------------------------------------------------------------------------------

. drop _mis_*
```

3 Main analysis

3.1 Prepare sensitive items

```
. d q14 q15 q16 q17
              storage   display    value
variable name   type    format     label      variable label
----------------------------------------------------------------------------------------------------------
q14             byte    %13.0g     q14        Have you ever intentionally taken something from a
                                                store without paying for it?
q15             byte    %13.0g     q15        Have you ever provided misleading or incorrect
                                                information on your tax return?
q16             byte    %13.0g     q16        Did you vote in the 2012 US presidential election?
q17             byte    %13.0g     q17        Did you honestly report whether your prediction was
                                                right?/you rolled a 6?

. gen byte shoplift = q14
(16 missing values generated)
. gen shoplift_p1   = (1-q14_pno) * q14_pcm
(1 missing value generated)
. gen shoplift_p0   = q14_pyes + (1-q14_pcm)
(1 missing value generated)
. gen byte taxeva   = q15
(16 missing values generated)
. gen taxeva_p1     = (1-q15_pno) * q15_pcm
(5 missing values generated)
. gen taxeva_p0     = q15_pyes + (1-q15_pcm)
(5 missing values generated)
. gen byte nonvote  = 1 - q16
(21 missing values generated)
. gen nonvote_p1    = (1-q16_pyes) * q16_pcm
(7 missing values generated)
. gen nonvote_p0    = q16_pno + (1-q16_pcm)
(7 missing values generated)
. gen byte cheat    = 1 - q17
(17 missing values generated)
. gen cheat_p1      = (1-q17_pyes) * q17_pcm
(8 missing values generated)
. gen cheat_p0      = q17_pno + (1-q17_pcm)
(8 missing values generated)
```

3.2 Comparative validation (Fig 1/S1 Table)

Helper program:

```
capt prog drop rrt
program rrt, eclass
    syntax varname [if] [in] [aw fw iw pw], p1(varname) p0(varname) [ * ]
    tempvar depvar
    qui gen `depvar' = (`varlist' - (`p0')) / ((`p1') - (`p0'))
    qui mean `depvar' `if' `in' [`weight'`exp'], `options'
    tempname b
    mat `b' = e(b)
    local eqs
    local N_over = e(N_over)
```

```
    mat coleq `b' = `varlist'
    eret repost b=`b', rename
    mean
end
```

Estimation:

```
. foreach v in shoplift taxeva nonvote {
  2.     di _n as res `"==> `v'"'
  3.     rrt `v', over(senstec) p1(`v'_p1) p0(`v'_p0)
  4.     est sto `v'
  5.     nlcom (CM: _b[CM]-_b[DQ]) (UQ: _b[UQ]-_b[DQ]) (FR: _b[FR]-_b[DQ]), post
  6.     est sto d`v'
  7. }

==> shoplift

Mean estimation                   Number of obs   =      6,136

           DQ: senstec = DQ
           CM: senstec = CM
           UQ: senstec = UQ
           FR: senstec = FR

--------------------------------------------------------------
        Over |       Mean   Std. Err.     [95% Conf. Interval]
-------------+------------------------------------------------
shoplift     |
          DQ |   .4023438   .0177063      .3676333    .4370542
          CM |   .4641868   .0162203      .4323894    .4959842
          UQ |   .5453174   .0163735      .5132195    .5774152
          FR |     .49222   .0170557      .4587849    .5256551
--------------------------------------------------------------

          CM:  _b[CM]-_b[DQ]
          UQ:  _b[UQ]-_b[DQ]
          FR:  _b[FR]-_b[DQ]

------------------------------------------------------------------------------
        Mean |      Coef.   Std. Err.      z    P>|z|     [95% Conf. Interval]
-------------+----------------------------------------------------------------
          CM |    .061843   .0240127     2.58   0.010     .0147791     .108907
          UQ |   .1429736   .0241165     5.93   0.000     .0957062     .190241
          FR |   .0898763   .0245847     3.66   0.000     .0416912    .1380614
------------------------------------------------------------------------------

==> taxeva

Mean estimation                   Number of obs   =      6,136

           DQ: senstec = DQ
           CM: senstec = CM
           UQ: senstec = UQ
           FR: senstec = FR

--------------------------------------------------------------
        Over |       Mean   Std. Err.     [95% Conf. Interval]
-------------+------------------------------------------------
taxeva       |
          DQ |   .1002604   .0108449      .0790006    .1215202
          CM |   .1952329   .0149807      .1658655    .2246004
          UQ |    .176287    .014159      .1485305    .2040436
          FR |   .1430028   .0152268       .113153    .1728525
--------------------------------------------------------------

          CM:  _b[CM]-_b[DQ]
          UQ:  _b[UQ]-_b[DQ]
          FR:  _b[FR]-_b[DQ]

------------------------------------------------------------------------------
        Mean |      Coef.   Std. Err.      z    P>|z|     [95% Conf. Interval]
-------------+----------------------------------------------------------------
          CM |   .0949725   .0184941     5.14   0.000     .0587247    .1312204
          UQ |   .0760266    .017835     4.26   0.000     .0410706    .1109826
          FR |   .0427424    .018694     2.29   0.022     .0061028    .0793819
------------------------------------------------------------------------------
```

```
==> nonvote
Mean estimation                   Number of obs   =      6,131

          DQ: senstec = DQ
          CM: senstec = CM
          UQ: senstec = UQ
          FR: senstec = FR

--------------------------------------------------------------
        Over |       Mean   Std. Err.     [95% Conf. Interval]
-------------+------------------------------------------------
nonvote      |
          DQ |   .3446475   .0171828      .3109632    .3783319
          CM |   .3810852   .0160718      .3495788    .4125916
          UQ |   .3474351   .0159523      .3161631    .3787071
          FR |   .3251002   .0168013      .2921637    .3580367
--------------------------------------------------------------

         CM:  _b[CM]-_b[DQ]
         UQ:  _b[UQ]-_b[DQ]
         FR:  _b[FR]-_b[DQ]

------------------------------------------------------------------------------
        Mean |      Coef.   Std. Err.      z    P>|z|     [95% Conf. Interval]
-------------+----------------------------------------------------------------
          CM |   .0364377   .0235277     1.55   0.121    -.0096758    .0825511
          UQ |   .0027876   .0234462     0.12   0.905    -.0431661    .0487413
          FR |  -.0195473   .0240319    -0.81   0.416     -.066649    .0275544
------------------------------------------------------------------------------

. local i 0

. foreach g in predict roll6 {
  2.     local ++i
  3.     di _n as res `"==> dicegame = `:lab dicegame `i''"'
  4.     rrt cheat if dicegame==`i', over(senstec) p1(cheat_p1) p0(cheat_p0)
  5.     est sto `g'
  6.     nlcom (CM: _b[CM]-_b[DQ]) (UQ: _b[UQ]-_b[DQ]) (FR: _b[FR]-_b[DQ]), post
  7.     est sto d`g'
  8. }

==> dicegame = prediction
Mean estimation                   Number of obs   =      3,065

          DQ: senstec = DQ
          CM: senstec = CM
          UQ: senstec = UQ
          FR: senstec = FR

--------------------------------------------------------------
        Over |       Mean   Std. Err.     [95% Conf. Interval]
-------------+------------------------------------------------
cheat        |
          DQ |   .0232558   .0076712      .0082146     .038297
          CM |   .1540808   .0205389      .1138093    .1943523
          UQ |   .0373548   .0162843      .0054256     .069284
          FR |   .0085131   .0183445     -.0274555    .0444818
--------------------------------------------------------------

         CM:  _b[CM]-_b[DQ]
         UQ:  _b[UQ]-_b[DQ]
         FR:  _b[FR]-_b[DQ]

------------------------------------------------------------------------------
        Mean |      Coef.   Std. Err.      z    P>|z|     [95% Conf. Interval]
-------------+----------------------------------------------------------------
          CM |   .1308249   .0219248     5.97   0.000     .0878532    .1737967
          UQ |    .014099   .0180007     0.78   0.433    -.0211817    .0493796
          FR |  -.0147427   .0198838    -0.74   0.458    -.0537142    .0242289
------------------------------------------------------------------------------

==> dicegame = roll-a-six
Mean estimation                   Number of obs   =      3,070

          DQ: senstec = DQ
          CM: senstec = CM
```

```
        UQ: senstec = UQ
        FR: senstec = FR

--------------------------------------------------------------
        Over |       Mean   Std. Err.     [95% Conf. Interval]
-------------+------------------------------------------------
cheat        |
          DQ |   .0393701   .0099763      .0198092     .058931
          CM |   .1433933   .0205794      .1030424    .1837442
          UQ |   .0522663   .0165942      .0197295    .0848032
          FR |  -.0193614   .0172802     -.0532433    .0145205
--------------------------------------------------------------

         CM:  _b[CM]-_b[DQ]
         UQ:  _b[UQ]-_b[DQ]
         FR:  _b[FR]-_b[DQ]

------------------------------------------------------------------------------
        Mean |      Coef.   Std. Err.      z    P>|z|     [95% Conf. Interval]
-------------+----------------------------------------------------------------
          CM |   .1040232   .0228701     4.55   0.000     .0591987    .1488477
          UQ |   .0128962   .0193622     0.67   0.505    -.0250529    .0508454
          FR |  -.0587314   .0199532    -2.94   0.003    -.0978391   -.0196238
------------------------------------------------------------------------------
```

Table:

```
. esttab shoplift taxeva nonvote predict roll6, ///
>     nostar mti nonumb eqlab(none) compress transform(@*100 100) b(2) se(2)

------------------------------------------------------------
             shoplift    taxeva   nonvote   predict     roll6
------------------------------------------------------------
DQ              40.23     10.03     34.46      2.33      3.94
               (1.77)    (1.08)    (1.72)    (0.77)    (1.00)
CM              46.42     19.52     38.11     15.41     14.34
               (1.62)    (1.50)    (1.61)    (2.05)    (2.06)
UQ              54.53     17.63     34.74      3.74      5.23
               (1.64)    (1.42)    (1.60)    (1.63)    (1.66)
FR              49.22     14.30     32.51      0.85     -1.94
               (1.71)    (1.52)    (1.68)    (1.83)    (1.73)
------------------------------------------------------------
N                6136      6136      6131      3065      3070
------------------------------------------------------------
Standard errors in parentheses
. esttab dshoplift dtaxeva dnonvote dpredict droll6, ///
>     mti nonumb eqlab(none) compress transform(@*100 100) b(2) se(2)

----------------------------------------------------------------------------
            dshoplift       dtaxeva      dnonvote      dpredict        droll6
----------------------------------------------------------------------------
CM               6.18*         9.50***       3.64         13.08***      10.40***
               (2.40)        (1.85)        (2.35)        (2.19)        (2.29)
UQ              14.30***       7.60***       0.28          1.41          1.29
               (2.41)        (1.78)        (2.34)        (1.80)        (1.94)
FR               8.99***       4.27*        -1.95         -1.47         -5.87**
               (2.46)        (1.87)        (2.40)        (1.99)        (2.00)
----------------------------------------------------------------------------
N                6136          6136          6131          3065          3070
----------------------------------------------------------------------------
Standard errors in parentheses
* p<0.05, ** p<0.01, *** p<0.001
```

Graph:

```
. local list  shoplift \ taxeva \ nonvote \ predict \ roll6
. local dlist dshoplift, aseq(shoplift) \     ///
>             dtaxeva  , aseq(taxeva)   \     ///
```

```
>             dnonvote , aseq(nonvote)  \     ///
>             dpredict , aseq(predict)  \     ///
>             droll6   , aseq(roll6)    \
. coefplot (`list', keep(DQ)) (`list', keep(CM)) (`list', keep(UQ)) ///
>          (`list', keep(FR)), aseq ///
>     || _skip (`dlist', keep(CM)) (`dlist', keep(UQ)) (`dlist', keep(FR)) ///
>     || , rescale(100) swap xline(0) xlab(#10, grid) yscale(alt noline)   ///
>     plotlabels(DQ CM UQ FR) bylabels("Prevalence estimate in %" "Difference to DQ") ///
>     coeflabels(shoplift = "Shoplifting"                               ///
>                taxeva   = "Tax evasion"                               ///
>                nonvote  = "Non-voting"                                ///
>                predict  = `""Cheating in the" "prediction game""'     ///
>                roll6    = `""Cheating in the" "roll-a-six game""'     ///
>                ,  tlc(none))                                          ///
>     byopts(legend(off) xrescale) legend(pos(0))
. gr_edit .plotregion1.move yaxis1[2] leftof 8 6
. addplot 1: , legend(order(2 "DQ" 4 "CM" 6 "UQ" 8 "FR") on bplace(se) cols(1)) norescaling
. estimates clear
```

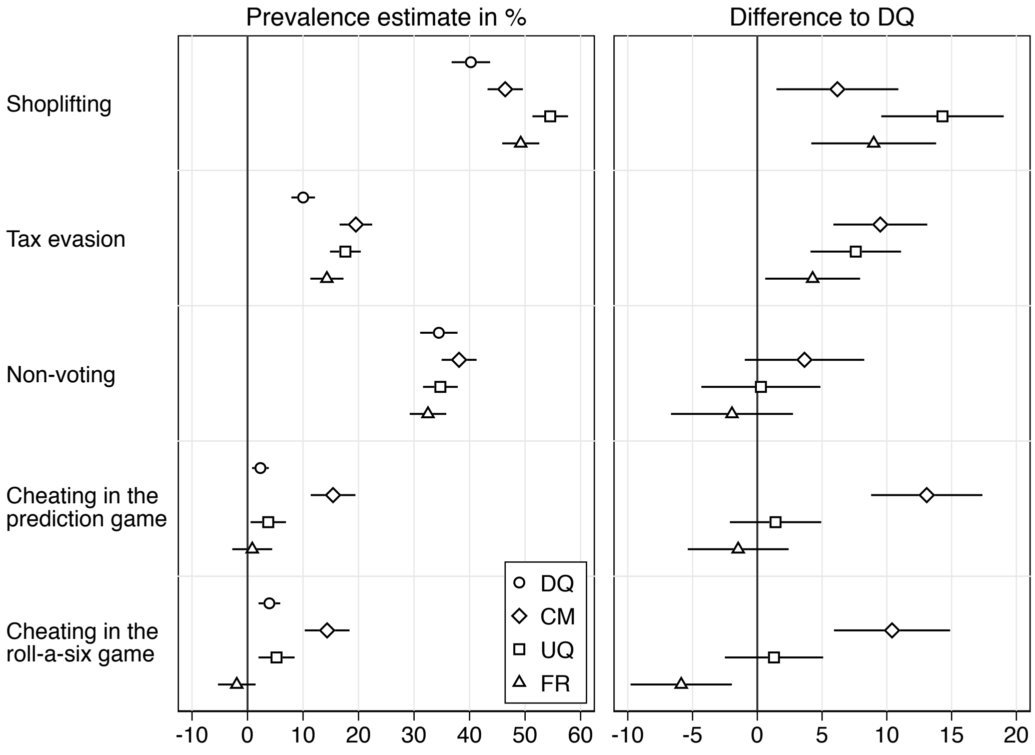

3.3 Aggregate-level validation (Fig 2/S2 Table)

Helper program:

```
capt prog drop rrt
program rrt, eclass
    syntax varlist [if] [in] [aw fw iw pw], p1(varname) p0(varname) [ roll(varname) * ]
    local claim: word 1 of `varlist'
    local sq: word 2 of `varlist'
    tempvar tmp1 tmp2 tmp3
    if "`roll'"=="" {   // => prediction game
        qui gen `tmp1' = `claim' - 1/6
    }
```

```
    else {              // => roll-a-six game
        qui gen byte `tmp1' = (`roll'!=6) & (`claim'==1) if `roll'<. & `claim'<.
    }
    qui gen `tmp2' = (`sq' - (`p0')) / ((`p1') - (`p0'))
    qui gen `tmp3' =  `tmp2' - `tmp1'
    qui mean `tmp1' `tmp2' `tmp3' `if' `in' [`weight'`exp'], `options'
    tempname b
    mat `b' = e(b)
    local eqs
    local N_over = e(N_over)
    foreach eq in cheated estimate difference {
        forv i=1/`N_over' {
            local eqs `eqs' `eq'
        }
    }
    mat coleq `b' = `eqs'
    eret repost b=`b', rename
    mean
end
```

Estimation:

```
. rrt q6 cheat if dicegame==1, over(senstec) p1(cheat_p1) p0(cheat_p0)

Mean estimation                   Number of obs   =      3,065

                DQ: senstec = DQ
                CM: senstec = CM
                UQ: senstec = UQ
                FR: senstec = FR
```

Over	Mean	Std. Err.	[95% Conf.	Interval]
cheated				
DQ	.2364341	.0249668	.1874807	.2853876
CM	.266323	.0145293	.2378349	.2948112
UQ	.2613386	.0179953	.2260545	.2966227
FR	.2653016	.0180157	.2299775	.3006257
estimate				
DQ	.0232558	.0076712	.0082146	.038297
CM	.1540808	.0205389	.1138093	.1943523
UQ	.0373548	.0162843	.0054256	.069284
FR	.0085131	.0183445	-.0274555	.0444818
difference				
DQ	-.2131783	.0247036	-.2616155	-.1647411
CM	-.1122423	.02423	-.159751	-.0647336
UQ	-.2239838	.0230163	-.2691128	-.1788549
FR	-.2567885	.0248441	-.3055012	-.2080758

```
. eststo pred

. rrt q6 cheat if dicegame==2, roll(q6_roll1) over(senstec) p1(cheat_p1) p0(cheat_p0)

Mean estimation                   Number of obs   =      3,070

                DQ: senstec = DQ
                CM: senstec = CM
                UQ: senstec = UQ
                FR: senstec = FR
```

Over	Mean	Std. Err.	[95% Conf.	Interval]
cheated				
DQ	.0446194	.0105915	.0238522	.0653866
CM	.0604203	.0070537	.0465899	.0742507
UQ	.0501285	.0078282	.0347794	.0654777
FR	.0520156	.0080128	.0363045	.0677267
estimate				

```
          DQ |   .0393701   .0099763     .0198092     .058931
          CM |   .1433933   .0205794     .1030424    .1837442
          UQ |   .0522663   .0165942     .0197295    .0848032
          FR |  -.0193614   .0172802    -.0532433    .0145205
-------------+----------------------------------------------
difference   |
          DQ |  -.0052493   .0074286    -.0198148    .0093161
          CM |    .082973   .0207942      .042201     .123745
          UQ |   .0021378   .0165142    -.0302422    .0345178
          FR |   -.071377   .0174499    -.1055917   -.0371622
------------------------------------------------------------

. eststo roll6
```

Table:

```
. esttab pred roll6, unstack nogap nostar mti nonumb compress ///
>     transform(@*100 100) b(2) se(2)
```

	pred cheated	estimate	differe~e	roll6 cheated	estimate	differe~e
DQ	23.64	2.33	-21.32	4.46	3.94	-0.52
	(2.50)	(0.77)	(2.47)	(1.06)	(1.00)	(0.74)
CM	26.63	15.41	-11.22	6.04	14.34	8.30
	(1.45)	(2.05)	(2.42)	(0.71)	(2.06)	(2.08)
UQ	26.13	3.74	-22.40	5.01	5.23	0.21
	(1.80)	(1.63)	(2.30)	(0.78)	(1.66)	(1.65)
FR	26.53	0.85	-25.68	5.20	-1.94	-7.14
	(1.80)	(1.83)	(2.48)	(0.80)	(1.73)	(1.74)
N	3065			3070		

Standard errors in parentheses

Graph:

```
. coefplot (pred, keep(cheated:)    \ roll6, keep(cheated:))          ///
>          (pred, keep(estimate:)   \ roll6, keep(estimate:))         ///
>          , bylabel("Cheating prevalence in %")                      ///
>     ||   (pred, keep(difference:) \ roll6, keep(difference:))       ///
>          , bylabel("Bias")                                          ///
>     || , aseq norecycle rescale(100) xline(0) xlab(#10, grid)       ///
>     eqlab("Prediction game" "Roll-a-six game")                      ///
>     byopts(legend(off) xrescale) legend(pos(0))
. addplot 1: , legend(order(2 "true rate" 4 "survey estimate") on bplace(se) cols(1)) norescaling
. estimates clear
```

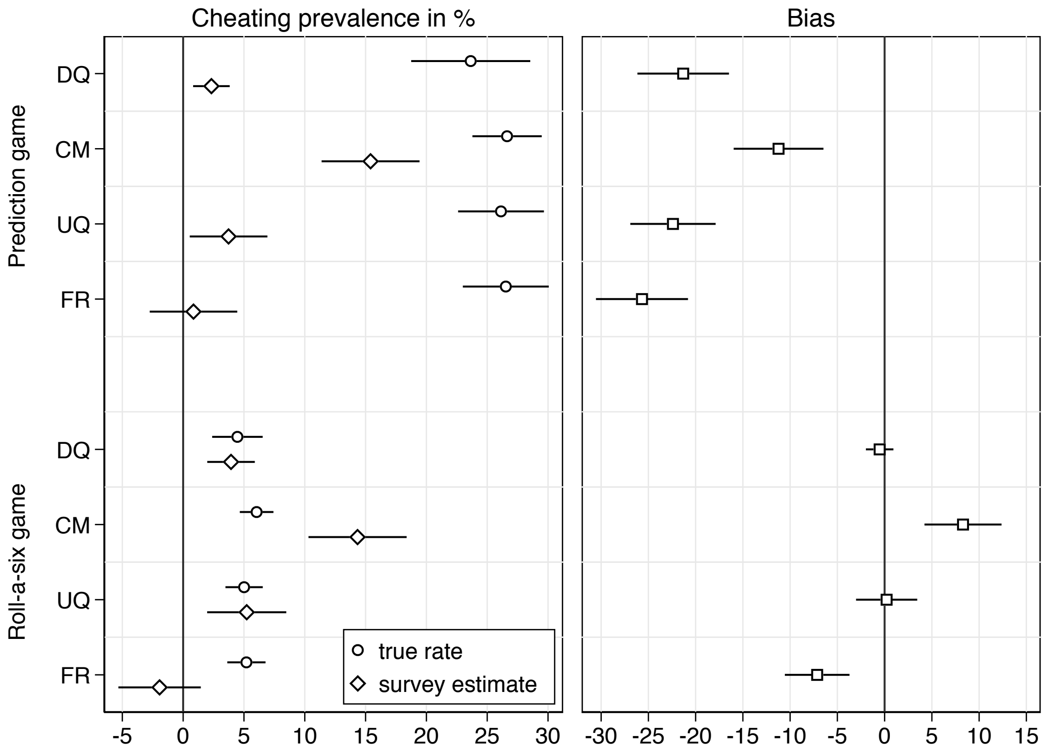

3.4 Individual-level validation

3.4.1 Prediction game

Helper programs:

```
// program using joint mean estimation and nlcom for standard errors
capt prog drop rrt
program rrt, eclass
    syntax varlist [if] [in] [aw fw iw pw], over(varname) p1(varname) p0(varname) [ * ]
    marksample touse
    markout `touse' `over' `p1' `p0'
    local claim: word 1 of `varlist'
    local sq: word 2 of `varlist'
    tempvar tmp
    qui gen `tmp' = (`sq' - (`p0')) / ((`p1') - (`p0')) if `touse'
    assert (`tmp'<.) if `touse'
    qui count if `touse'
    local N = r(N)
    tempname TPR vTPR FPR vFPR P vP DPR vDPR CCR vCCR
    qui mean `tmp' if `touse' & `claim'==1 [`weight'`exp'], over(`over') `options'
    mat `TPR' = e(b)
    mat coleq `TPR' = "TPR"
    mat `vTPR' = e(V)
    mat coleq `vTPR' = "TPR"
    mat roweq `vTPR' = "TPR"
    qui mean `tmp' if `touse' & `claim'==0 [`weight'`exp'], over(`over') `options'
    mat `FPR' = e(b)
    mat coleq `FPR' = "FPR"
    mat `vFPR' = e(V)
    mat coleq `vFPR' = "FPR"
    mat roweq `vFPR' = "FPR"
    qui mean `claim' if `touse' [`weight'`exp'], over(`over') `options'
    mat `P' = e(b)
```

```
    mat coleq `P' = "P"
    mat `vP' = e(V)
    mat coleq `vP' = "P"
    mat roweq `vP' = "P"
    local N_over = colsof(`P')
    tempname b V
    mat `b' = `TPR', `FPR', `P'
    mat `V' = (`vTPR', `vFPR'*0, `vP'*0)    ///
           \ (`vFPR'*0, `vFPR', `vFPR'*0)  ///
           \ (`vP'*0, `vP'*0, `vP')
    eret post `b' `V'
    local levels: coln `P'
    local expTPR
    local expP
    local expCCR
    foreach l of local levels {
        local expTPR `expTPR' ///
            (`l': ([P]_b[`l']*[TPR]_b[`l']-[FPR]_b[`l']/6)/([P]_b[`l']-1/6))
        local expP   `expP'   ///
            (`l': [P]_b[`l']-1/6)
        local expCCR `expCCR' ///
            (`l': (([P]_b[`l']*[TPR]_b[`l']-[FPR]_b[`l']/6)/([P]_b[`l']-1/6))*([P]_b[`l']-1/6) ///
            + (1 - max(0, [FPR]_b[`l']))*(1-([P]_b[`l']-1/6)))
    }
    foreach l in TPR P CCR {
        qui nlcom `exp`l''
        mat ``l'' = r(b)
        mat coleq ``l'' = "`l'"
        mat `v`l'' = r(V)
        mat coleq `v`l'' = "`l'"
        mat roweq `v`l'' = "`l'"
    }
    mat `b' = `TPR', `FPR', `P', `CCR'
    mat `V' = (`vTPR', `vFPR'*0, `vP'*0, `vCCR'*0)        ///
           \ (`vFPR'*0, `vFPR', `vFPR'*0, `vFPR'*0)      ///
           \ (`vP'*0, `vP'*0, `vP', `vP'*0)              ///
           \ (`vCCR'*0, `vCCR'*0, `vCCR'*0, `vCCR')
    eret post `b' `V', obs(`N') esample(`touse')
    eret scalar N_over = `N_over'
    eret local cmd "rrt"
    _coef_table_header
    eret di
end
// fast program for bootstrap estimation
capt prog drop rrtbs
program rrtbs, eclass
    syntax varlist [if] [in], over(varname) p1(varname) p0(varname)
    marksample touse
    markout `touse' `over' `p1' `p0'
    local claim: word 1 of `varlist'
    local sq: word 2 of `varlist'
    tempvar tmp
    qui gen `tmp' = (`sq' - (`p0')) / ((`p1') - (`p0')) if `touse'
    assert (`tmp'<.) if `touse'
    qui count if `touse'
    local N = r(N)
    qui levelsof `over' if `touse', local(levels)
    local N_over: list sizeof levels
    local coln
    foreach l of local levels {
        local coln `coln' `: label (`over') `l''
    }
    foreach m in TPR FPR P CCR {
        tempname `m'
        mat ``m'' = J(1, `N_over', .)
        mat coln ``m'' = `coln'
        mat coleq ``m'' = `m'
    }
```

```
    local i 0
    foreach l of local levels {
        local ++i
        su `claim' if `touse' & `over'==`l', meanonly
        local pclaim = r(mean)
        mat `P'[1, `i'] = r(mean) - 1/6
        su `tmp' if `touse' & `claim'==0 & `over'==`l', meanonly
        mat `FPR'[1, `i'] = r(mean)
        su `tmp' if `touse' & `claim'==1 & `over'==`l', meanonly
        mat `TPR'[1, `i'] = (`pclaim'*r(mean) - `FPR'[1, `i']/6)/(`pclaim'-1/6)
        mat `CCR'[1, `i'] = min(1,max(0,`TPR'[1, `i']))*(`pclaim'-1/6) ///
                            + (1-min(1,max(0,`FPR'[1, `i'])))*(1-(`pclaim'-1/6))
    }
    tempname b
    mat `b' = `TPR', `FPR', `P', `CCR'
    eret post `b', obs(`N') esample(`touse')
    eret scalar N_over = `N_over'
    _coef_table_header
    eret di
end
```

Estimation:

```
. rrt q6 cheat if dicegame==1, over(senstec) p1(cheat_p1) p0(cheat_p0)
                                                  Number of obs     =      3,065

------------------------------------------------------------------------------
             |      Coef.   Std. Err.      z    P>|z|     [95% Conf. Interval]
-------------+----------------------------------------------------------------
TPR          |
          DQ |   .0983607   .0322171     3.05   0.002     .0352164     .161505
          CM |    .283634   .0551521     5.14   0.000     .1755378    .3917302
          UQ |   .1480406   .0470357     3.15   0.002     .0558524    .2402288
          FR |   .0893349   .0504695     1.77   0.077    -.0095835    .1882534
-------------+----------------------------------------------------------------
FPR          |
          DQ |          0  (omitted)
          CM |   .1070532   .0264156     4.05   0.000     .0552796    .1588269
          UQ |  -.0018059   .0194135    -0.09   0.926    -.0398555    .0362438
          FR |  -.0206718   .0230592    -0.90   0.370    -.0658671    .0245234
-------------+----------------------------------------------------------------
P            |
          DQ |   .2364341   .0249668     9.47   0.000        .1875    .2853682
          CM |    .266323   .0145293    18.33   0.000     .2378461    .2947999
          UQ |   .2613386   .0179953    14.52   0.000     .2260684    .2966088
          FR |   .2653016   .0180157    14.73   0.000     .2299915    .3006118
-------------+----------------------------------------------------------------
CCR          |
          DQ |   .7868217    .024708    31.84   0.000     .7383949    .8352485
          CM |   .7306727   .0292892    24.95   0.000     .6732669    .7880785
          UQ |   .7773501   .0204535    38.01   0.000      .737262    .8174382
          FR |   .7583991    .021761    34.85   0.000     .7157484    .8010498
------------------------------------------------------------------------------

. est sto pred

. // difference in TPR compared to direct questioning
. nlcom (dCM:_b[TPR:CM]-_b[TPR:DQ]) (dUQ:_b[TPR:UQ]-_b[TPR:DQ]) ///
>     (dFR:_b[TPR:FR ]-_b[TPR:DQ]), noheader

------------------------------------------------------------------------------
             |      Coef.   Std. Err.      z    P>|z|     [95% Conf. Interval]
-------------+----------------------------------------------------------------
         dCM |   .1852733   .0638725     2.90   0.004     .0600855    .3104611
         dUQ |   .0496799   .0570113     0.87   0.384    -.0620602    .1614201
         dFR |  -.0090257   .0598758    -0.15   0.880    -.1263802    .1083287
------------------------------------------------------------------------------

. // difference in CCR compared to direct questioning
. nlcom (dCM:_b[CCR:CM]-_b[CCR:DQ]) (dUQ:_b[CCR:UQ]-_b[CCR:DQ]) ///
>     (dFR:_b[CCR:FR ]-_b[CCR:DQ]), noheader
```

```
------------------------------------------------------------------------------
             |      Coef.   Std. Err.      z    P>|z|     [95% Conf. Interval]
-------------+----------------------------------------------------------------
         dCM |   -.056149    .038319    -1.47   0.143    -.1312528    .0189548
         dUQ |  -.0094716   .0320754    -0.30   0.768    -.0723382     .053395
         dFR |  -.0284226   .0329245    -0.86   0.388    -.0929535    .0361082
------------------------------------------------------------------------------

. // bootstrap estimates
. bootstrap, reps($reps) nodots: ///
>     rrtbs q6 cheat if dicegame==1, over(senstec) p1(cheat_p1) p0(cheat_p0)

Bootstrap results                               Number of obs     =      3,065
                                                Replications      =      1,000

------------------------------------------------------------------------------
             |   Observed   Bootstrap                         Normal-based
             |      Coef.   Std. Err.      z    P>|z|     [95% Conf. Interval]
-------------+----------------------------------------------------------------
TPR          |
          DQ |   .0983607   .0334468     2.94   0.003      .032806    .1639153
          CM |    .283634   .0554837     5.11   0.000      .174888      .39238
          UQ |   .1480406   .0472129     3.14   0.002      .055505    .2405761
          FR |   .0893349   .0531078     1.68   0.093    -.0147545    .1934243
-------------+----------------------------------------------------------------
FPR          |
          DQ |          0  (omitted)
          CM |   .1070532   .0265468     4.03   0.000     .0550226    .1590839
          UQ |  -.0018059   .0197516    -0.09   0.927    -.0405183    .0369065
          FR |  -.0206718    .022422    -0.92   0.357    -.0646181    .0232745
-------------+----------------------------------------------------------------
P            |
          DQ |   .2364341   .0259162     9.12   0.000     .1856393    .2872289
          CM |    .266323   .0141939    18.76   0.000     .2385035    .2941425
          UQ |   .2613386   .0177502    14.72   0.000     .2265488    .2961284
          FR |   .2653016   .0183111    14.49   0.000     .2294125    .3011908
-------------+----------------------------------------------------------------
CCR          |
          DQ |   .7868217   .0257888    30.51   0.000     .7362765    .8373669
          CM |   .7306727   .0300559    24.31   0.000     .6717643    .7895812
          UQ |   .7773501   .0232287    33.47   0.000     .7318228    .8228774
          FR |   .7583991   .0232575    32.61   0.000     .7128151     .803983
------------------------------------------------------------------------------

. // difference in TPR compared to direct questioning
. nlcom (dCM:_b[TPR:CM]-_b[TPR:DQ]) (dUQ:_b[TPR:UQ]-_b[TPR:DQ]) ///
>     (dFR:_b[TPR:FR ]-_b[TPR:DQ]), noheader

------------------------------------------------------------------------------
             |      Coef.   Std. Err.      z    P>|z|     [95% Conf. Interval]
-------------+----------------------------------------------------------------
         dCM |   .1852733   .0639846     2.90   0.004     .0598658    .3106808
         dUQ |   .0496799   .0572166     0.87   0.385    -.0624625    .1618223
         dFR |  -.0090257   .0631311    -0.14   0.886    -.1327605     .114709
------------------------------------------------------------------------------

. // difference in CCR compared to direct questioning
. nlcom (dCM:_b[CCR:CM]-_b[CCR:DQ]) (dUQ:_b[CCR:UQ]-_b[CCR:DQ]) ///
>     (dFR:_b[CCR:FR ]-_b[CCR:DQ]), noheader

------------------------------------------------------------------------------
             |      Coef.   Std. Err.      z    P>|z|     [95% Conf. Interval]
-------------+----------------------------------------------------------------
         dCM |   -.056149   .0398172    -1.41   0.158    -.1341892    .0218913
         dUQ |  -.0094716   .0340919    -0.28   0.781    -.0762905    .0573473
         dFR |  -.0284226   .0355422    -0.80   0.424     -.098084    .0412387
------------------------------------------------------------------------------
```

3.4.2 Roll-a-six game

Helper programs:

```
// program using joint mean estimation and nlcom for standard errors
capt prog drop rrt
program rrt, eclass
    syntax varlist [if] [in] [aw fw iw pw], over(varname) p1(varname) p0(varname) [ * ]
    marksample touse
    markout `touse' `over' `p1' `p0'
    local roll: word 1 of `varlist'
    local claim: word 2 of `varlist'
    local sq: word 3 of `varlist'
    tempvar tmp1 tmp2
    qui gen byte `tmp1' = (`roll'!=6) & (`claim'==1) if `touse'
    qui gen `tmp2' = (`sq' - (`p0')) / ((`p1') - (`p0')) if `touse'
    assert (`tmp1'<. & `tmp2'<.) if `touse'
    qui count if `touse'
    local N = r(N)
    tempname TPR vTPR FPR vFPR P vP DPR vDPR CCR vCCR
    qui mean `tmp2' if `touse' & `tmp1'==1 [`weight'`exp'], over(`over') `options'
    mat `TPR' = e(b)
    mat coleq `TPR' = "TPR"
    mat `vTPR' = e(V)
    mat coleq `vTPR' = "TPR"
    mat roweq `vTPR' = "TPR"
    qui mean `tmp2' if `touse' & `tmp1'==0 [`weight'`exp'], over(`over') `options'
    mat `FPR' = e(b)
    mat coleq `FPR' = "FPR"
    mat `vFPR' = e(V)
    mat coleq `vFPR' = "FPR"
    mat roweq `vFPR' = "FPR"
    qui mean `tmp1' if `touse' [`weight'`exp'], over(`over') `options'
    mat `P' = e(b)
    mat coleq `P' = "P"
    mat `vP' = e(V)
    mat coleq `vP' = "P"
    mat roweq `vP' = "P"
    local N_over = colsof(`P')
    tempname b V
    mat `b' = `TPR', `FPR', `P'
    mat `V' = (`vTPR', `vFPR'*0, `vP'*0)    ///
            \ (`vFPR'*0, `vFPR', `vFPR'*0)  ///
            \ (`vP'*0, `vP'*0, `vP')
    eret post `b' `V'
    local levels: coln `P'
    local expCCR
    foreach l of local levels {
        local expCCR `expCCR' ///
            (`l': [TPR]_b[`l']*[P]_b[`l'] + (1 - max(0, [FPR]_b[`l']))*(1-[P]_b[`l']))
    }
    qui nlcom `expCCR'
    mat `CCR' = r(b)
    mat coleq `CCR' = "CCR"
    mat `vCCR' = r(V)
    mat coleq `vCCR' = "CCR"
    mat roweq `vCCR' = "CCR"
    mat `b' = `TPR', `FPR', `P', `CCR'
    mat `V' = (`vTPR', `vFPR'*0, `vP'*0, `vCCR'*0)        ///
            \ (`vFPR'*0, `vFPR', `vFPR'*0, `vFPR'*0)      ///
            \ (`vP'*0, `vP'*0, `vP', `vP'*0)              ///
            \ (`vCCR'*0, `vCCR'*0, `vCCR'*0, `vCCR')
    eret post `b' `V', obs(`N') esample(`touse')
    eret scalar N_over = `N_over'
    eret local cmd "rrt"
    _coef_table_header
    eret di
end
// fast program for bootstrap estimation
capt prog drop rrtbs
program rrtbs, eclass
    syntax varlist [if] [in], over(varname) p1(varname) p0(varname)
```

```
    marksample touse
    markout `touse' `over' `p1' `p0'
    local roll: word 1 of `varlist'
    local claim: word 2 of `varlist'
    local sq: word 3 of `varlist'
    tempvar tmp1 tmp2
    qui gen byte `tmp1' = (`roll'!=6) & (`claim'==1) if `touse'
    qui gen `tmp2' = (`sq' - (`p0')) / ((`p1') - (`p0')) if `touse'
    assert (`tmp1'<. & `tmp2'<.) if `touse'
    qui count if `touse'
    local N = r(N)
    qui levelsof `over' if `touse', local(levels)
    local N_over: list sizeof levels
    local coln
    foreach l of local levels {
        local coln `coln' `: label (`over') `l''
    }
    foreach m in TPR FPR P CCR {
        tempname `m'
        mat ``m'' = J(1, `N_over', .)
        mat coln ``m'' = `coln'
        mat coleq ``m'' = `m'
    }
    local i 0
    foreach l of local levels {
        local ++i
        su `tmp1' if `touse' & `over'==`l', meanonly
        local pcheat = r(mean)
        mat `P'[1, `i'] = r(mean)
        su `tmp2' if `touse' & `tmp1'==1 & `over'==`l', meanonly
        mat `TPR'[1, `i'] = r(mean)
        su `tmp2' if `touse' & `tmp1'==0 & `over'==`l', meanonly
        mat `FPR'[1, `i'] = r(mean)
        mat `CCR'[1, `i'] = min(1,max(0,`TPR'[1, `i']))*`pcheat' ///
                            + (1-min(1,max(0,`FPR'[1, `i'])))*(1-`pcheat')
    }
    tempname b
    mat `b' = `TPR', `FPR', `P', `CCR'
    eret post `b', obs(`N') esample(`touse')
    eret scalar N_over = `N_over'
    _coef_table_header
    eret di
end
```

Estimation:

```
. rrt q6_roll1 q6 cheat if dicegame==2, over(senstec) p1(cheat_p1) p0(cheat_p0)
                                                   Number of obs     =      3,070
```

	Coef.	Std. Err.	z	P>|z|	[95% Conf.	Interval]
TPR						
DQ	.7058824	.1139113	6.20	0.000	.4826204	.9291443
CM	.5292724	.0945779	5.60	0.000	.3439032	.7146417
UQ	.5477228	.1039083	5.27	0.000	.3440664	.7513793
FR	.4111111	.1066186	3.86	0.000	.2021425	.6200797
FPR						
DQ	.0082418	.0047453	1.74	0.082	-.0010588	.0175423
CM	.1185791	.0208271	5.69	0.000	.0777588	.1593993
UQ	.0261191	.0160437	1.63	0.104	-.0053259	.0575641
FR	-.0429813	.0168567	-2.55	0.011	-.0760198	-.0099427
P						
DQ	.0446194	.0105915	4.21	0.000	.0238604	.0653784
CM	.0604203	.0070537	8.57	0.000	.0465954	.0742453
UQ	.0501285	.0078282	6.40	0.000	.0347855	.0654716
FR	.0520156	.0080128	6.49	0.000	.0363107	.0677205

CCR						
DQ	.9790026	.0074535	131.35	0.000	.9643941	.9936112
CM	.860144	.0205367	41.88	0.000	.8198927	.9003953
UQ	.9525182	.0164469	57.91	0.000	.9202829	.9847536
FR	.9693686	.0072816	133.13	0.000	.9550969	.9836403

```
. est sto roll6

. // difference in TPR compared to direct questioning
. nlcom (dCM:_b[TPR:CM]-_b[TPR:DQ]) (dUQ:_b[TPR:UQ]-_b[TPR:DQ]) ///
>     (dFR:_b[TPR:FR ]-_b[TPR:DQ]), noheader
```

	Coef.	Std. Err.	z	P>|z|	[95% Conf.	Interval]
dCM	-.1766099	.1480566	-1.19	0.233	-.4667955	.1135757
dUQ	-.1581595	.154184	-1.03	0.305	-.4603546	.1440356
dFR	-.2947712	.1560234	-1.89	0.059	-.6005715	.011029

```
. // difference in CCR compared to direct questioning
. nlcom (dCM:_b[CCR:CM]-_b[CCR:DQ]) (dUQ:_b[CCR:UQ]-_b[CCR:DQ]) ///
>     (dFR:_b[CCR:FR ]-_b[CCR:DQ]), noheader
```

	Coef.	Std. Err.	z	P>|z|	[95% Conf.	Interval]
dCM	-.1188586	.0218475	-5.44	0.000	-.1616789	-.0760384
dUQ	-.0264844	.018057	-1.47	0.142	-.0618755	.0089067
dFR	-.009634	.01042	-0.92	0.355	-.0300569	.0107888

```
. // bootstrap estimates
. bootstrap, reps($reps) nodots: ///
>     rrtbs q6_roll1 q6 cheat if dicegame==2, over(senstec) p1(cheat_p1) p0(cheat_p0)

Bootstrap results                               Number of obs     =      3,070
                                                Replications      =      1,000
```

	Observed Coef.	Bootstrap Std. Err.	z	P>|z|	Normal-based [95% Conf.	Interval]
TPR						
DQ	.7058824	.1132603	6.23	0.000	.4838962	.9278685
CM	.5292724	.0999247	5.30	0.000	.3334236	.7251213
UQ	.5477228	.1035036	5.29	0.000	.3448596	.7505861
FR	.4111111	.1100371	3.74	0.000	.1954423	.6267799
FPR						
DQ	.0082418	.0047397	1.74	0.082	-.001048	.0175315
CM	.1185791	.0213805	5.55	0.000	.0766741	.160484
UQ	.0261191	.0160732	1.63	0.104	-.0053838	.057622
FR	-.0429813	.0171604	-2.50	0.012	-.076615	-.0093475
P						
DQ	.0446194	.0105167	4.24	0.000	.0240071	.0652317
CM	.0604203	.0069969	8.64	0.000	.0467066	.0741341
UQ	.0501285	.007954	6.30	0.000	.034539	.0657181
FR	.0520156	.00788	6.60	0.000	.0365712	.06746
CCR						
DQ	.9790026	.0073079	133.96	0.000	.9646794	.9933259
CM	.860144	.0210378	40.89	0.000	.8189108	.9013772
UQ	.9525182	.0161834	58.86	0.000	.9207994	.9842371
FR	.9693686	.0072603	133.52	0.000	.9551386	.9835986

```
. // difference in TPR compared to direct questioning
. nlcom (dCM:_b[TPR:CM]-_b[TPR:DQ]) (dUQ:_b[TPR:UQ]-_b[TPR:DQ]) ///
>     (dFR:_b[TPR:FR ]-_b[TPR:DQ]), noheader
```

```
             |      Coef.   Std. Err.      z    P>|z|     [95% Conf. Interval]
-------------+----------------------------------------------------------------
         dCM |  -.1766099   .1547516    -1.14   0.254    -.4799175    .1266977
         dUQ |  -.1581595   .1526368    -1.04   0.300    -.4573221    .1410031
         dFR |  -.2947712   .1552221    -1.90   0.058     -.599001    .0094585
------------------------------------------------------------------------------

. // difference in CCR compared to direct questioning
. nlcom (dCM:_b[CCR:CM]-_b[CCR:DQ]) (dUQ:_b[CCR:UQ]-_b[CCR:DQ]) ///
>     (dFR:_b[CCR:FR ]-_b[CCR:DQ]), noheader

------------------------------------------------------------------------------
             |      Coef.   Std. Err.      z    P>|z|     [95% Conf. Interval]
-------------+----------------------------------------------------------------
         dCM |  -.1188586   .0223727    -5.31   0.000    -.1627083   -.0750089
         dUQ |  -.0264844   .0174756    -1.52   0.130     -.060736    .0077672
         dFR |   -.009634     .00994    -0.97   0.332    -.0291161     .009848
------------------------------------------------------------------------------
```

3.4.3 Overview of results (Fig 3/S3 Table)

Table:

```
. esttab pred roll6, unstack drop(P:) nogap nostar mti nonumb compress ///
>     transform(@*100 100) b(2) se(2)

----------------------------------------------------------------------
                pred                             roll6
                 TPR        FPR        CCR        TPR        FPR        CCR
----------------------------------------------------------------------
DQ              9.84       0.00      78.68      70.59       0.82      97.90
              (3.22)        (.)     (2.47)    (11.39)     (0.47)     (0.75)
CM             28.36      10.71      73.07      52.93      11.86      86.01
              (5.52)     (2.64)     (2.93)     (9.46)     (2.08)     (2.05)
UQ             14.80      -0.18      77.74      54.77       2.61      95.25
              (4.70)     (1.94)     (2.05)    (10.39)     (1.60)     (1.64)
FR              8.93      -2.07      75.84      41.11      -4.30      96.94
              (5.05)     (2.31)     (2.18)    (10.66)     (1.69)     (0.73)
----------------------------------------------------------------------
N               3065                             3070
----------------------------------------------------------------------
Standard errors in parentheses
```

Graph:

```
. coefplot (pred, keep(TPR:)) || (roll6, keep(TPR:)) ///
>     ||   (pred, keep(FPR:)) || (roll6, keep(FPR:)) ///
>     ||   (pred, keep(CCR:)) || (roll6, keep(CCR:)) ///
>     || , bylabels("True positive rate" "True positive rate" ///
>                   "False positive rate" "False positive rate" ///
>                   "Correct classification rate" "Correct classification rate") xlab(#10, grid) ///
>         rescale(100) byopts(xrescale cols(2) scale(0.75)) ysize(5)

. addplot 3 4: , xline(0) norescaling

. addplot 1: , title(Prediction game) norescaling

. addplot 2: , title(Roll-a-six game) norescaling
```

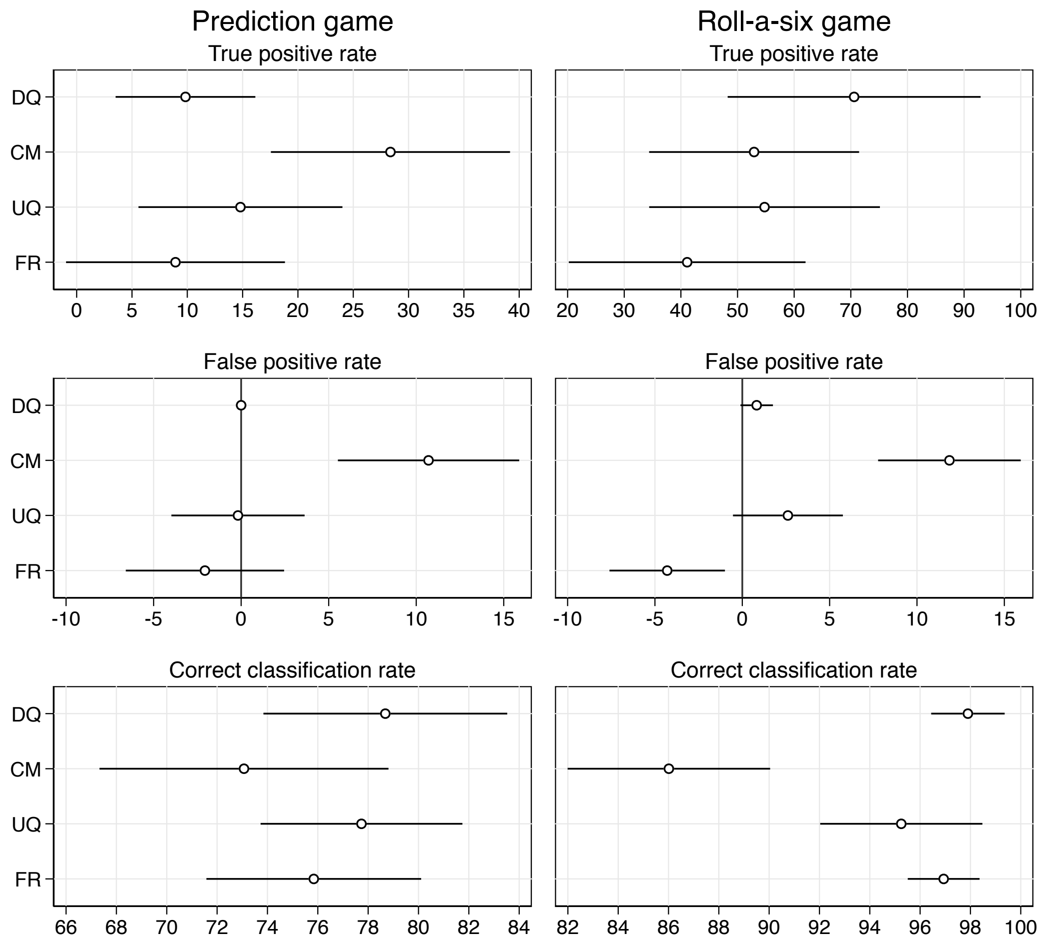

4 Discussion of assumption A2

4.1 False positive rates for true losers and true winners in the roll-a-six game

For the prediction game the assumption is made that the false positive rate for true winners is the same as for true losers. We can use the roll-a-six game to test this assumption.

Helper program:

```
capt prog drop rrt
program rrt, eclass
    syntax varlist [if] [in] [aw fw iw pw], over(varname) p1(varname) p0(varname) [ * ]
    marksample touse
    markout `touse' `over' `p1' `p0'
    local roll: word 1 of `varlist'
    local claim: word 2 of `varlist'
    local sq: word 3 of `varlist'
    assert (`roll'==6 & `claim'==0)==0 if `touse' // no false loosers
    qui replace `touse' = 0 if `roll'!=6 & `claim'==1 // exclude false winners
    tempvar tmp1 tmp2
    qui gen byte `tmp1' = 1 if (`roll'==6) & (`claim'==1) & `touse' // true winners
    qui replace  `tmp1' = 0 if (`roll'!=6) & (`claim'==0) & `touse' // true losers
    qui gen `tmp2' = (`sq' - (`p0')) / ((`p1') - (`p0')) if `touse'
    assert (`tmp1'<. & `tmp2'<.) if `touse'
```

```
    qui count if `touse'
    local N = r(N)
    qui mean `tmp2' if `touse' [`weight'`exp'], over(`over' `tmp1') `options'
    local lose 1
    local win  2
    local expr
    local coln
    foreach s in DQ CM UQ FR {
        local expr `expr' (_b[_subpop_`lose']) (_b[_subpop_`win']) ///
                          (_b[_subpop_`win'] - _b[_subpop_`lose'])
        local coln `"`coln'"`s':FPR true losers" "`s':FPR true winners" "'
        local coln `"`coln'"`s':difference" "'
        local lose = `lose' + 2
        local win  = `win'  + 2
    }
    qui nlcom `expr'
    tempname b V
    mat `b' = r(b)
    mat `V' = r(V)
    mat coln `b' = `coln'
    mat coln `V' = `coln'
    mat rown `V' = `coln'
    eret post `b' `V', obs(`N')
    eret local cmd "rrt"
    _coef_table_header
    eret di
end
```

Estimation:

```
. rrt q6_roll1 q6 cheat if dicegame==2, over(senstec) p1(cheat_p1) p0(cheat_p0)
                                               Number of obs     =      2,905
```

	Coef.	Std. Err.	z	P>|z|	[95% Conf.	Interval]
DQ						
FPR true losers	.0101351	.0058317	1.74	0.082	-.0012947	.021565
FPR true winners	0	(omitted)				
difference	-.0101351	.0058317	-1.74	0.082	-.021565	.0012947
CM						
FPR true losers	.1104792	.0227485	4.86	0.000	.0658929	.1550655
FPR true winners	.1572059	.0516836	3.04	0.002	.0559079	.2585039
difference	.0467267	.0564685	0.83	0.408	-.0639495	.1574029
UQ						
FPR true losers	.0329724	.017971	1.83	0.067	-.0022501	.0681948
FPR true winners	-.005986	.0350798	-0.17	0.865	-.0747411	.0627692
difference	-.0389583	.0394151	-0.99	0.323	-.1162105	.0382938
FR						
FPR true losers	-.0349313	.0189615	-1.84	0.065	-.072095	.0022325
FPR true winners	-.0797286	.0361287	-2.21	0.027	-.1505396	-.0089176
difference	-.0447973	.0408022	-1.10	0.272	-.1247682	.0351736

Interpretation: There is no evidence for a systematic difference in the false positive rate of true losers and true winners.

4.2 Results for the prediction game assuming the false positive rate among true winners to be zero

Instead of assuming the false positive rate of true winners and true losers to be equal, we now evaluate how the results change if we make an assumption that is most in favor of the sensitive questions techniques: that

true winners fully comply with the procedure and hence have a false positive rate of zero. Notation in the following exposition is the same as in the paper.

Assumption 2' (A2') *The false positive rate among true winners is equal to zero, that is,* $\Pr(Y^* = 1|X = X^* = 1) = 0$.

Under A1 and A2' the false positive rate is

$$\text{FPR} = \Pr(Y^* = 1|X = X^*) = \frac{\Pr(X = 0)}{\Pr(X = 0) + \frac{1}{6}} \cdot \frac{\Pr(Y = 1|X = 0) - p_{1|0}}{p_{1|1} - p_{1|0}}$$

because

$$\begin{aligned}
\Pr(Y^* = 1|X = X^*) &= \Pr(X = X^* = 0|X = X^*)\Pr(Y^* = 1|X = X^* = 0) \\
&\quad + \Pr(X = X^* = 1|X = X^*)\Pr(Y^* = 1|X = X^* = 1) \\
\Pr(X = X^* = 0|X = X^*) &= \frac{\Pr(X = X^* = 0 \cap X = X^*)}{\Pr(X = X^*)} = \frac{\Pr(X = X^* = 0)}{\Pr(X = X^*)} = \frac{\Pr(X = 0)}{\Pr(X = 0) + \frac{1}{6}} \\
\Pr(Y^* = 1|X = X^* = 0) &= \frac{\Pr(Y = 1|X = 0) - p_{1|0}}{p_{1|1} - p_{1|0}} \\
\Pr(Y^* = 1|X = X^* = 1) &= 0 \\
\Pr(X = X^*) &= \Pr(X = X^* = 0) + \Pr(X = X^* = 1) = \Pr(X = 0) + \Pr(X^* = 1) \\
&= \Pr(X = 0) + \frac{1}{6}
\end{aligned}$$

Furthermore, the true positive rate is

$$\text{TPR} = \frac{\Pr(X = 1)\frac{\Pr(Y=1|X=1) - p_{1|0}}{p_{1|1} - p_{1|0}}}{\Pr(X = 1) - \frac{1}{6}}$$

which follows directly from the equations in the paper by setting $\Pr(Y^* = 1|X = X^* = 1)$ to zero.

Helper program:

```
capt prog drop rrt
program rrt, eclass
    syntax varlist [if] [in] [aw fw iw pw], over(varname) p1(varname) p0(varname) [ * ]
    marksample touse
    markout `touse' `over' `p1' `p0'
    local claim: word 1 of `varlist'
    local sq: word 2 of `varlist'
    tempvar tmp
    qui gen `tmp' = (`sq' - (`p0')) / ((`p1') - (`p0')) if `touse'
    assert (`tmp'<.) if `touse'
    qui count if `touse'
    local N = r(N)
    tempname TPR vTPR FPR vFPR P vP DPR vDPR CCR vCCR
    qui mean `tmp' if `touse' & `claim'==1 [`weight'`exp'], over(`over') `options'
    mat `TPR' = e(b)
    mat coleq `TPR' = "TPR"
    mat `vTPR' = e(V)
    mat coleq `vTPR' = "TPR"
    mat roweq `vTPR' = "TPR"
    qui mean `tmp' if `touse' & `claim'==0 [`weight'`exp'], over(`over') `options'
    mat `FPR' = e(b)
    mat coleq `FPR' = "FPR"
    mat `vFPR' = e(V)
    mat coleq `vFPR' = "FPR"
```

```
    mat roweq `vFPR' = "FPR"
    qui mean `claim' if `touse' [`weight'`exp'], over(`over') `options'
    mat `P' = e(b)
    mat coleq `P' = "P"
    mat `vP' = e(V)
    mat coleq `vP' = "P"
    mat roweq `vP' = "P"
    local N_over = colsof(`P')
    tempname b V
    mat `b' = `TPR', `FPR', `P'
    mat `V' = (`vTPR', `vFPR'*0, `vP'*0)    ///
            \ (`vFPR'*0, `vFPR', `vFPR'*0)  ///
            \ (`vP'*0, `vP'*0, `vP')
    eret post `b' `V'
    local levels: coln `P'
    local expFPR
    local expTPR
    local expP
    local expCCR
    foreach l of local levels {
        local expFPR `expFPR' ///
            (`l': ((1-[P]_b[`l'])*[FPR]_b[`l'])/((1-[P]_b[`l'])+1/6))
        local expTPR `expTPR' ///
            (`l': ([P]_b[`l']*[TPR]_b[`l'])/([P]_b[`l']-1/6))
        local expP   `expP'   ///
            (`l': [P]_b[`l']-1/6)
        local expCCR `expCCR' ///
            (`l': (([P]_b[`l']*[TPR]_b[`l'])/([P]_b[`l']-1/6))*([P]_b[`l']-1/6) ///
            + (1 - max(0, ((1-[P]_b[`l'])*[FPR]_b[`l'])/((1-[P]_b[`l'])+1/6))) * ///
              (1-([P]_b[`l']-1/6)))
    }
    foreach l in FPR TPR P CCR {
        qui nlcom `exp`l''
        mat ``l'' = r(b)
        mat coleq ``l'' = "`l'"
        mat `v`l'' = r(V)
        mat coleq `v`l'' = "`l'"
        mat roweq `v`l'' = "`l'"
    }
    mat `b' = `TPR', `FPR', `P', `CCR'
    mat `V' = (`vTPR', `vFPR'*0, `vP'*0, `vCCR'*0)        ///
            \ (`vFPR'*0, `vFPR', `vFPR'*0, `vFPR'*0)      ///
            \ (`vP'*0, `vP'*0, `vP', `vP'*0)              ///
            \ (`vCCR'*0, `vCCR'*0, `vCCR'*0, `vCCR')
    eret post `b' `V', obs(`N') esample(`touse')
    eret scalar N_over = `N_over'
    eret local cmd "rrt"
    _coef_table_header
    eret di
end
```

Estimation:

```
. rrt q6 cheat if dicegame==1, over(senstec) p1(cheat_p1) p0(cheat_p0)
                                                Number of obs     =      3,065
```

	Coef.	Std. Err.	z	P>|z|	[95% Conf. Interval]	
TPR						
DQ	.0983607	.0322171	3.05	0.002	.0352164	.161505
CM	.3506286	.0529995	6.62	0.000	.2467515	.4545057
UQ	.1468889	.04537	3.24	0.001	.0579653	.2358125
FR	.0763485	.0483013	1.58	0.114	-.0183203	.1710174
FPR						
DQ	0	(omitted)				
CM	.0827344	.0204205	4.05	0.000	.0427108	.1227579
UQ	-.0013984	.0150331	-0.09	0.926	-.0308628	.028066

```
          FR |  -.0159824   .0178286    -0.90   0.370    -.0509258     .018961
-------------+----------------------------------------------------------------
P            |
          DQ |   .2364341   .0249668     9.47   0.000        .1875    .2853682
          CM |    .266323   .0145293    18.33   0.000     .2378461    .2947999
          UQ |   .2613386   .0179953    14.52   0.000     .2260684    .2966088
          FR |   .2653016   .0180157    14.73   0.000     .2299915    .3006118
-------------+----------------------------------------------------------------
CCR          |
          DQ |   .7868217    .024708    31.84   0.000     .7383949    .8352485
          CM |   .7663571   .0227279    33.72   0.000     .7218112    .8109031
          UQ |   .7770491   .0201959    38.48   0.000     .7374658    .8166324
          FR |   .7549538   .0214189    35.25   0.000     .7129735     .796934
------------------------------------------------------------------------------

. est sto pred2
. // difference in TPR compared to direct questioning
. nlcom (dCM:_b[TPR:CM]-_b[TPR:DQ]) (dUQ:_b[TPR:UQ]-_b[TPR:DQ]) ///
>     (dFR:_b[TPR:FR ]-_b[TPR:DQ]), noheader
------------------------------------------------------------------------------
             |      Coef.   Std. Err.      z    P>|z|     [95% Conf. Interval]
-------------+----------------------------------------------------------------
         dCM |   .2522679   .0620233     4.07   0.000     .1307046    .3738313
         dUQ |   .0485282   .0556451     0.87   0.383    -.0605342    .1575906
         dFR |  -.0220121     .05806    -0.38   0.705    -.1358075    .0917833
------------------------------------------------------------------------------

. // difference in CCR compared to direct questioning
. nlcom (dCM:_b[CCR:CM]-_b[CCR:DQ]) (dUQ:_b[CCR:UQ]-_b[CCR:DQ]) ///
>     (dFR:_b[CCR:FR ]-_b[CCR:DQ]), noheader
------------------------------------------------------------------------------
             |      Coef.   Std. Err.      z    P>|z|     [95% Conf. Interval]
-------------+----------------------------------------------------------------
         dCM |  -.0204646   .0335715    -0.61   0.542    -.0862635    .0453343
         dUQ |  -.0097726   .0319118    -0.31   0.759    -.0723185    .0527733
         dFR |  -.0318679   .0326994    -0.97   0.330    -.0959577    .0322218
------------------------------------------------------------------------------
```

Graph:

```
. coefplot (pred, keep(TPR:)) (pred2, keep(TPR:)) ///
>     ||   (pred, keep(FPR:)) (pred2, keep(FPR:)) ///
>     ||   (pred, keep(CCR:)) (pred2, keep(CCR:)) ///
>     || , bylabels("True positive rate" "False positive rate"  ///
>                 "Correct classification rate" ) xlab(#10, grid) ///
>         rescale(100) byopts(xrescale cols(1) legend(pos(5))) ysize(6) xsize(4)
. addplot 2: , xline(0) norescaling
. addplot 1: , title(Prediction game) norescaling ///
>     legend(order(2 "Assuming same FPR for true winners and true losers (A2)" ///
>         4 "Assuming FPR for true winners to be zero (A2')") cols(1) size(small))
```

Interpretation: If the false positive rate of true winners is assumed to be zero (instead of equal to the false positive rate of true losers), the estimated overall false positive rate in the CM is somewhat smaller (reduction from 10.7% to 8.3%), the true positive rate is somewhat higher (increase from 28.4% to 35.1%), and, consequently, the correct classification rate is also somewhat higher (increase from 73.1% to 76.6%). For the other questioning techniques, the difference in results is negligible. That is, the alternative assumption makes the CM look a little better, but it does not change our main result that the CM is affected by a substantial amount of false positives.

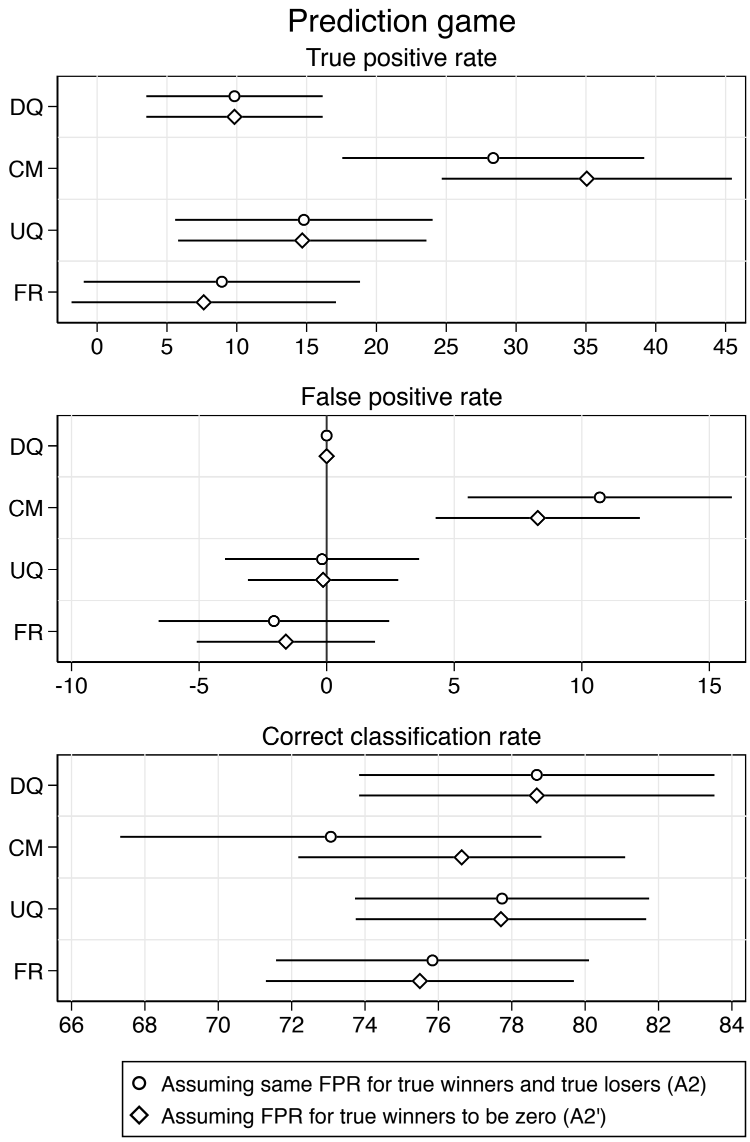

Prediction game
True positive rate
DQ
CM
UQ
FR
0 5 10 15 20 25 30 35 40 45
False positive rate
DQ
CM
UQ
FR
-10 -5 0 5 10 15
Correct classification rate
DQ
CM
UQ
FR
66 68 70 72 74 76 78 80 82 84
Assuming same FPR for true winners and true losers (A2)
Assuming FPR for true winners to be zero (A2')
