# Supplementary material for: More is not always better: An experimental individual-level validation of the randomized response technique and the crosswise model
Source: PLoS One. 2018 Aug 14;13(8):e0201770. doi: 10.1371/journal.pone.0201770 (PMC6091935; doi:10.1371/journal.pone.0201770)
Supplement: S1 Documentation — (PDF) [file pone.0201770.s005.pdf]

MTurk Survey on “Mood and Personality”

Documentation

Marc Höglinger

ETH Zurich, Sociology
CLU D2, CH-8092 Zurich
hoeglinger@soz.gess.ethz.ch

Ben Jann

University of Bern, Institute of Sociology
Fabrikstrasse 8, CH-3012 Bern
ben.jann@soz.unibe.ch

2016

ETH Zurich and University of Bern

This study was funded by the German Research Foundation (DGF) as part of the project "Asking Sensitive Questions: Possibilities and Limits of Randomized Response and Other Techniques in Different Survey Modes" (DI 292/5 to Andreas Diekmann), the Chair of Sociology of the ETH Zurich, and the Institute of Sociology at the University of Bern.

Please cite this document as follows:

Höglinger, Marc, Ben Jann. 2016. MTurk Survey on "Mood and Personality". Documentation. ETH Zurich and University of Bern.

Contents

1 Introduction **2**

1.1 Study overview 2

1.2 Pretests 4

1.3 Survey items adopted from the literature 6

2 Experimental design **6**

2.1 Factor one: dice game 7

2.1.1 Prediction game 7

2.1.2 Roll-a-six game 7

2.2 Factor two: sensitive question technique 10

2.2.1 Direct questioning 10

2.2.2 CM Question 11

2.2.3 UQ Benford 12

2.2.4 FR Number 12

2.2.5 Design parameters 14

2.3 Factor three: randomizing device test 15

2.3.1 Benford procedure 15

2.3.2 Unrelated questions for CM Question 15

2.3.3 Unrelated questions for UQ Benford 16

2.3.4 Pick-a-number device (standard) 16

2.3.5 Pick-a-number device (generic) 18

A Appendix **21**

A.1 HIT details 21

A.2 Questionnaire 22

A.3 JavaScript code 57

A.3.1 Dice 57

A.3.2 Pick-a-number fields 64

A.4 Codebook 67

1 Introduction

Social desirability and the fear of negative consequences often deter a considerable share of survey respondents from responding truthfully to sensitive questions. Self-reports of norm-breaking behavior such as shoplifting, tax evasion, non-voting or cheating for money might be subject to considerable misreporting. Thus, resulting prevalence estimates are inaccurate. Even though online surveys seem to be somewhat less prone to misreporting than less anonymous survey modes, misreporting remains at a substantial level (Kreuter et al. 2008). Indirect techniques for surveying sensitive questions such as the Randomized Response Technique (RRT, Warner 1965) are intended to mitigate misreporting by providing complete concealment of individual answers.

However, it is far from clear whether these indirect techniques actually produce more valid measurements than standard direct questioning. In order to evaluate the validity of different sensitive question techniques we carried out an online validation experiment in which respondents' self-reports of norm-breaking behavior were validated against observed actual behavior.

This document describes the design of the validation experiment and provides details on the questionnaire, the different sensitive question technique implementations, the field work, and the resulting dataset. The appendix contains a codebook of the data and facsimiles of the questionnaire pages and other survey materials.

1.1 Study overview

Our validation experiment was presented to participants as an online "Survey on mood and personality". The survey contained questions on respondents' personal background and personality and an incentivized dice game inspired by Fischbacher and Heusi (2008) and Greene and Paxton (2009), where respondents could win a $2 bonus payment. Because the game relied on self-reports about dice roll outcomes, cheating (i.e., illegitimately claiming a bonus payment) was easily possible. After playing the dice game, respondents were asked whether they played honestly, using one of several randomly assigned sensitive question techniques. Answers to this question can be validated and allow for the evaluation of the validity of the data obtained through the different sensitive question techniques. Besides the question on honest playing in the dice game, three other sensitive questions—on shoplifting, tax evasion, and voting—were asked. The answers to these questions cannot be validated against known values, but the resulting prevalence estimates can be compared across the different techniques.

Participants were recruited via the crowdsourcing platform Amazon Mechanical Turk (MTurk). MTurk has been used frequently for recruiting participants for scientific surveys and experiments (see Horton et al. 2011, Mason and Suri 2012, Paolacci et al. 2010). MTurk workers, so-called "Turkers", select job announcements posted by requesters on MTurk, the so-called "Human Intelligence Tasks" (HITs). In the announcement, Turkers see a short HIT description describing the task, the requester, and the corresponding payment for completing the task. We posted a HIT that asked for filling out a "Survey on mood and personality" for a base payment of $1 and the chance to win an additional $2 bonus payment (see Appendix A.1

Table 1: Study overview

Participants	"Turkers" (Amazon Mechanical Turk Workers) residing in the US
Number of participants	6'505
Payment	\$1 for completing the survey, possibility to win \$2 bonus
Experimental design	Online Survey with $2 \times 4 \times 5$ factorial design<br>Factor 1: Dice game (2 conditions)<br>Factor 2: Sensitive question technique (4 conditions)<br>Factor 3: Randomizing device test (5 conditions)
Field date	November 26 to December 5, 2013
Survey method	Self-recruiting online survey with personalized access links provided via the Amazon Mechanical Turk platform
Survey title	"Survey on mood and personality"
Survey software	Qualtrics (see Qualtrics Labs Inc. 2012)

for HIT details). Our HIT was posted on November 26, 2013 and remained available until December 5, 2013, when our quota of 6500 participants was reached. MTurk integration was done by Stefan Wehrli at the ETH Decision Science Laboratory (ETH DeSciL). Table 1 contains some key information about the study.

After having accepted the HIT, participants received an access link to an online questionnaire that started with some questions on the respondents' personal background. Then, respondents were assigned to one of two different dice games, the prediction game and the roll-a-six game. In these games, respondents had to roll a virtual die implemented in the questionnaire and then indicate whether they were eligible for the $2 bonus payment. Some more questions on personal background and personality followed. Then, four sensitive items on respondent's misconduct were asked: shoplifting, tax evasion, voting, and honest reporting in the dice game. The sensitive items were surveyed using various sensitive question techniques that were randomly assigned to the respondents: Direct questioning, a crosswise-model RRT variant (CM Question), an unrelated-question RRT variant (UQ Benford), and a forced response RRT variant (FR Number). After that, respondents had to evaluate the survey and the sensitive question technique employed. Lastly, some items intended for testing particular randomizing devices followed. When finishing the survey, respondents were displayed an exit code that they could enter on the MTurk webpage to receive their payment within the next 24 hours. Respondents were paid according to their claim in the dice game, irrespective of whether they played honestly or not and irrespective of their answer to the sensitive questions. We debriefed respondents by disclosing that the study's goal was to develop methods for surveying sensitive topics in areas such as epidemiology or criminology and reassured them that their answers will be handled

strictly confidentially and that payment will be according to the claim they made in the dice game.

The questionnaire contained a maximum of 23 pages, depending on the experimental conditions a respondent was assigned to (see table 2). Respondents could not navigate backwards when filling out the questionnaire (i.e., there was no back button). Median response time was 6.7 minutes; the 5th and 95th percentiles were 3.8 and 13.2 minutes, respectively.

1.2 Pretests

Prior to the main study we carried out three exploratory pretests. Each pretest was followed by alterations to the experimental design and the questionnaire. In the first pretest ($N = 53$) we checked whether there was a sufficient level of dishonest playing in the dice games. An external virtual die was used in this first pretest, that is, in order to roll the die respondents had to follow a link to an external webpage (www.random.org) that provided them with a virtual die. This design with an external die was inspired by Suri et al. (2011). The resulting cheating rates were substantial for the prediction game (56%, $N = 26$) and, although considerably lower, still significant for the roll-a-six game (20%, $N = 27$).

In the second pretest ($N = 246$) we checked whether using an internal die implemented directly within the questionnaire would still result in sufficiently high rates of dishonest playing. An internal die has the advantage that outcome randomization is under full control of the researchers: whether a participant actually rolls the die as well as the roll outcome are traceable. A disadvantage is that respondents might become suspicious and, as a consequence, be less inclined to cheat. However, resulting cheating rates were still considerable (30%, $N = 47$, for the prediction game game and 6%, $N = 45$, for the roll-a-six game). Furthermore, the roll-a-six game with external die, which was also implemented in this pretest, produced a nearly identical point estimate of cheating as the roll-a-six game with internal die (5%, $N = 50$). This convinced us that using an internal die would be feasible for our study. We also checked the effect of our treatment by comparing cheating rates in the dice games to rates in corresponding non-incentivized game versions. As expected, cheating was reduced in the non-incentivized version of the prediction game (0%, $N = 35$), indicating that it was the monetary incentive that caused cheating in the incentivized game. However, contrary to our expectations, cheating was non-negligible in both versions of the roll-a-six game (with internal die: 6%, $N = 35$; with external die: 10%, $N = 34$). These cheating rates might be explained by participants intrinsic motivation to win in the game, subjective expectations that there will be a benefit, misunderstanding, or by some other form of respondents' noncompliance. Finally, we tested whether different wording of the sensitive question on cheating in the dice game had an effect on self disclosure rates ("Did you cheat when reporting ..." versus "Did you honestly report whether ..."). Results revealed no systematic difference. For the main survey we chose the wording: "Did you honestly report whether ...".

In the third pretest ($N = 727$) we used a larger sample to estimate the rates of cheating more precisely. The cheating rate was 32% ($N = 244$) in the prediction game and 7% ($N = 237$) in the roll-a-six game. Furthermore, we assessed a new dice game variant where respondents had

Table 2: Questionnaire structure

Page	Topic	Page filter/Different page versions
Introduction		
1	Starting page	
2	Screening question	
3	Personal background I	
Dice game		*(different versions depending on game condition)*
4	Intro dice game	
5	Prediction dice game	(only prediction game)
6	Dice rolling	
Survey questions		
7	Satisfaction	(different versions depending on game condition)
8	Big Five	
9	Personal background II	
10	MTurk and other employment	
Sensitive items		*(different versions depending on technique condition)*
11	Intro sensitive questions	
12	Explanation special technique	(only RRT)
13	Benford procedure	(only UQ Benford)
14	Shoplifting	
15	Tax evasion	
16	Voting	
17	Honest dice game reporting	(different versions depending on game condition)
Survey evaluation		
18	Trust in survey confidentiality	(different page versions depending on dice game)
19	Evaluation of special technique	(only RRT)
Randomizing device test		
20	Randomizing device test	(different versions depending on device test condition)
21	Birth date knowledge	
22	Respondents' comments	
23	Final page	

Figure 1: Experimental factors and possible combinations

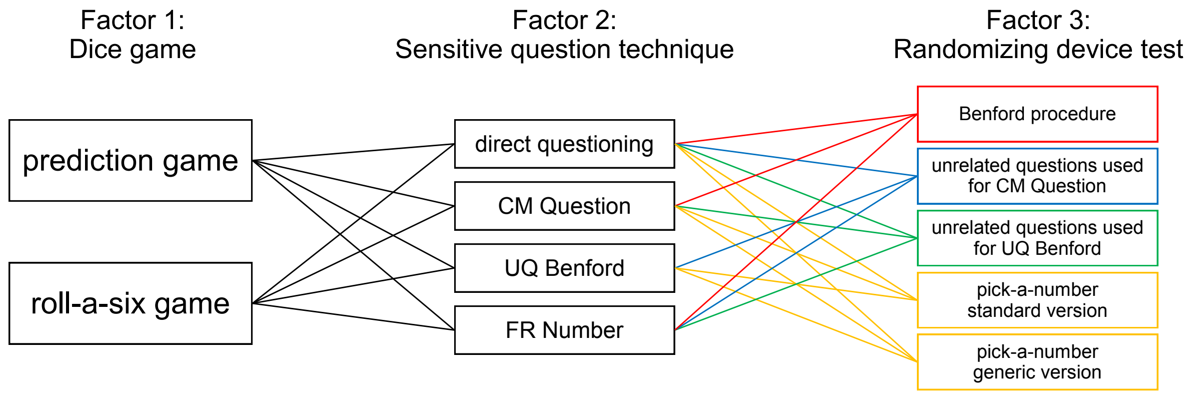

to roll a randomly assigned target number in order to win the bonus (instead of always having to roll a six). However, as this variant did not result in a higher cheating rate than the standard roll-a-six game, we did not use it in the main study. In the third pretest we also varied the base payment ($0.5 vs. $1) and the bonus payment ($1 vs. $2). The variations in payments did not have any clear effects on the cheating rates.

1.3 Survey items adopted from the literature

Several survey items have been adopted from the literature. We employed a screening question from Berinsky et al. (2012). The question about risk aversion is an item from the German Socio-Economic Panel (Dohmen et al. 2005). The "Big Five" personality dimensions are measured using the BFI-10 from Rammstedt and John (2007). The item on tax evasion is taken from John et al. (2013). Items on the evaluation of the survey confidentiality and the the sensitive question techniques are partially based on Stirnemann (2009) and Coutts and Jann (2011).

2 Experimental design

We employed a $2 \times 4 \times 5$ factorial design. Factor one was the type of the dice game that had to be played by the respondents (two variants). Factor two was the type of questioning technique used to ask the sensitive questions (four variants). Factor three assigned one of several tests to evaluate different randomizing devices to the respondents (five variants). Factors one and two were fully crossed. Factor three, however, was assigned in a way such that a different randomizing device was tested than the one employed in factor two. Figure 1 illustrates the experimental factors and the possible combinations. In the remainder we describe each factor and the corresponding experimental conditions in detail.

2.1 Factor one: dice game

Participants were randomly assigned to one of two dice games in which they could win a $2 bonus payment: the prediction game or the roll-a-six game. The games were inspired by the ones used in Fischbacher and Heusi (2008) and Greene and Paxton (2009). In both games participants used a virtual die integrated in the questionnaire, which they "rolled" by clicking on a button. Roll outcomes were random and followed a uniform distribution. The die could be rolled up to twenty times. However, it was made clear to respondents that only the first roll counts. The virtual die was implemented using JavaScript (authored by Philip Tschiemer, see Appendix A.3 for the code). Roll outcomes were predefined using stratified randomization within all combinations of dice games and sensitive question techniques. The number of rolls executed by a respondent was recorded.

2.1.1 Prediction game

In the prediction game, participants had to correctly predict the outcome of a dice roll to win the bonus payment. Participants made their prediction, memorized it, rolled the virtual die, and then indicated whether the roll outcome corresponded to their prediction or not (see figure 2). Correct predictions were awarded with a $2 bonus payment. Participants could easily cheat because they made their prediction in private and the bonus payment relied on their self-report.

Individual cheating is not detectable in the prediction game. A validation of the self-reports, however, is possible at the aggregate level. Because roll outcomes were random and followed a uniform distribution, one sixth of all predictions should turn out to be correct and five sixth should turn out to be wrong. A systematic deviation from one sixth of the participants claiming a correct prediction can be attributed to cheating.

2.1.2 Roll-a-six game

In the roll-a-six game, participants had to roll a six in order to win a $2 bonus payment. Respondents rolled a virtual die and then indicated whether their outcome was a six or not (see figure 3). Again, cheating was easily possible as the bonus payment relied on participants' self-reports. A participant could just indicate having rolled a six even if the actual outcome was different.

Roll outcomes were random and followed a uniform distribution. One sixth of all participants rolled a six. Because the roll outcome is known for every participant, validation of the answers is possible at the individual level. However, because it was quite obvious to participants that outcomes could be traced and the legitimacy of their claims could be checked, cheating was considerably lower in the roll-a-six game than in the prediction game, where cheating was completely safe. Furthermore, participants who cheated in the roll-a-six game might have been more likely to admit their cheating.

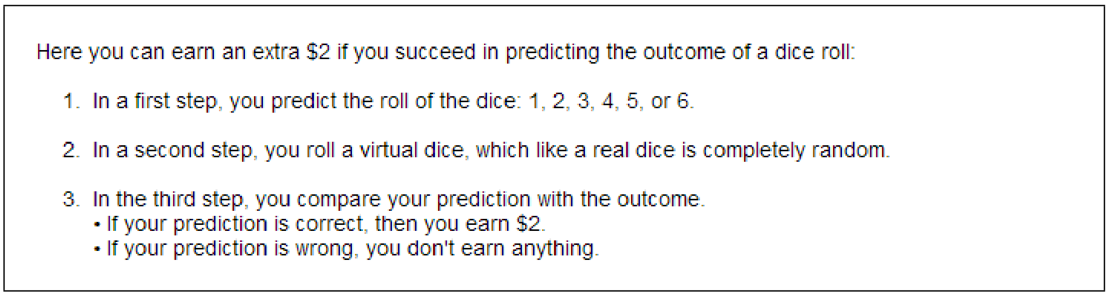

(a) Screen 1: Introduction

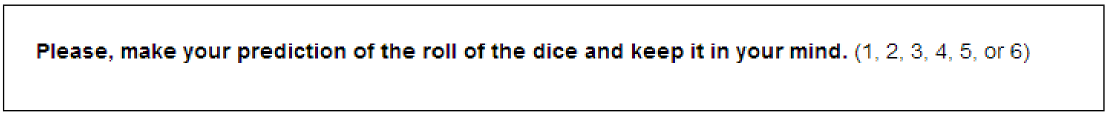

(b) Screen 2: Making the prediction

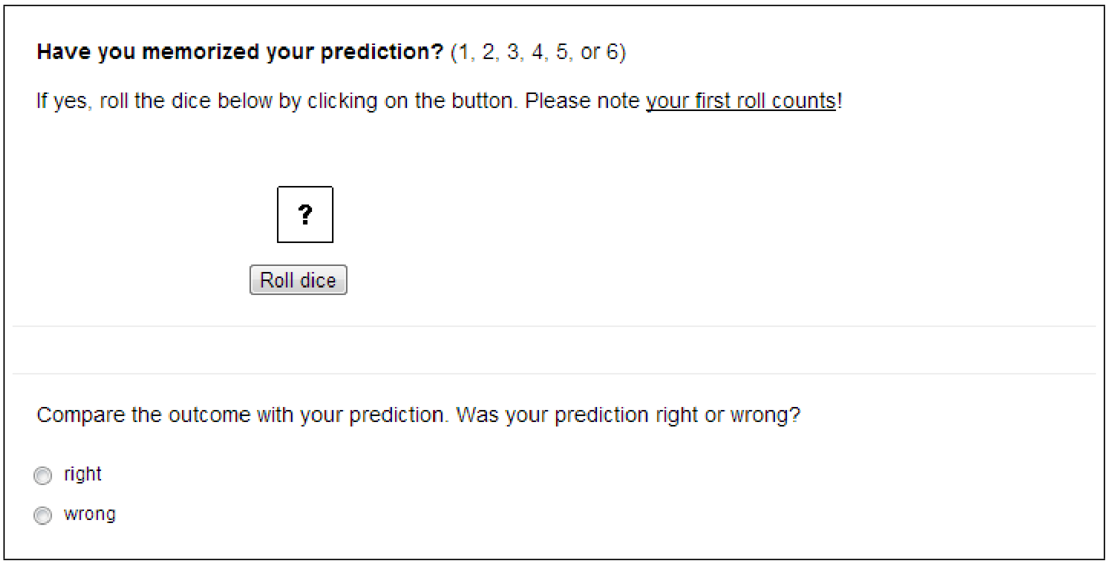

(c) Screen 3: Rolling the die and indicating the otucome

Figure 2: Prediction game

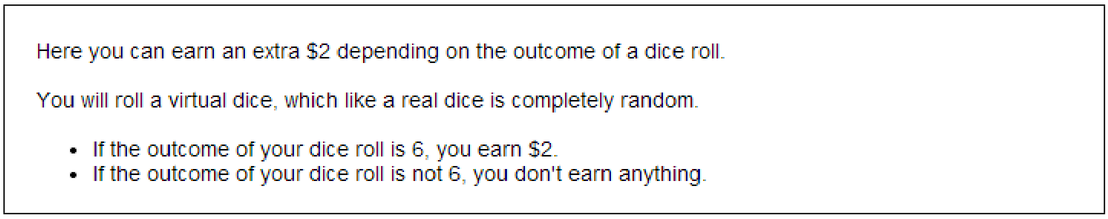

(a) Screen 1: Introduction

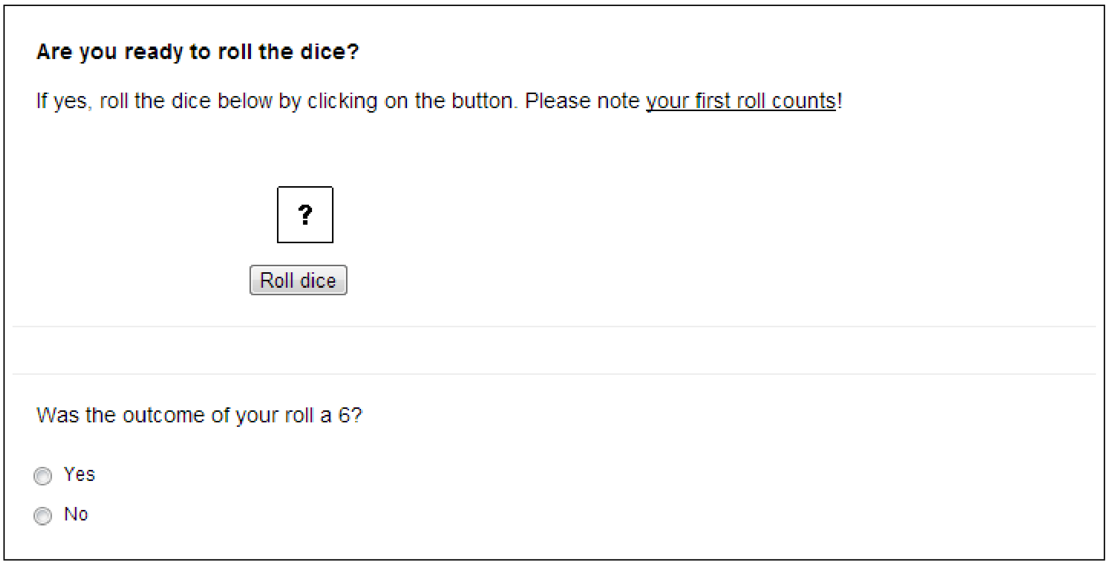

(b) Screen 2: Rolling the die and indicating the outcome

Figure 3: Roll-a-six game

Table 3: Sensitive items

Item	Wording
shoplifting	Have you ever intentionally taken something from a store without paying for it?
tax evasion	Have you ever provided misleading or incorrect information on your tax return? (adopted from John et al. 2013)
voting	Did you vote in the 2012 US presidential election?
honest dice game reporting	In the $2 dice task/game at the beginning of this survey:<br>Did you honestly report whether you actually rolled a 6? (*prediction game*)<br>Did you honestly report whether your prediction of the dice roll was right? (*roll-a-six game*)

Table 4: Sensitive question techniques

Experimental condition	Technique	Randomizing device
Direct questioning	direct questioning	
CM Question	crosswise-model RRT	unrelated question
UQ Benford	unrelated-question RRT	Benford procedure + unrelated question
FR Number	forced-response RRT	pick-a-number device

2.2 Factor two: sensitive question technique

To evaluate different sensitive question techniques, respondents were asked four sensitive questions (table 3) using one of four randomly assigned sensitive question techniques: Direct questioning, CM Question, UQ Benford, and FR Number (see table 4). Details on each of these techniques are presented in the following subsections.

2.2.1 Direct questioning

The direct questioning condition served as benchmark for the evaluation of the different sensitive question techniques. As in all other experimental conditions, the sensitive items were preceded by a screen announcing some sensitive questions, stating the importance of honest answers for the success of the study, providing some privacy assurance and telling respondents that answers to the sensitive questions would not affect their payment or the HIT approval[1].

[1] "HIT approval" means that the requester accepts a task submitted by a Turker and pays accordingly. A Turker's HIT approval rate serves as a reputation signal on MTurk and can be used as selection criterion by

Figure 4: Direct questioning

Have you ever intentionally taken something from a store without paying for it?

- Yes
- No

Figure 5: CM Question

Question A:
Is your father's birthday between the 1st and the 6th of the month (including the 1st and 6th)?
(If you do not know, please use the birth date of someone else you know.)

Question B:
Have you ever intentionally taken something from a store without paying for it?

Compare your responses to questions A & B. Are they identical or different?

- Identical *(both responses "No" or both "Yes")*
- Different *(one response "Yes", the other "No")*

Figure 4 presents a screenshot of the first sensitive item as presented in the direct questioning condition.

2.2.2 CM Question

The CM Question condition is an implementation of the unrelated-question crosswise-model RRT as used in Jann et al. (2011) or Höglinger et al. (2014). Respondents were asked two questions at the same time: A sensitive question and an unrelated non-sensitive question. Respondents then had to indicate whether their answers to the two questions were identical (both "No", or both "Yes") or different (one "Yes", the other "No") (see figure 5).

Due to the mixing with the answer to the nonsensitive question, a respondent's answer to the sensitive question remains completely private. Nonetheless, prevalence estimation for the sensitive question is possible at the aggregate level if the probability distribution of the unrelated non-sensitive question is known. As unrelated questions we used questions about the birth dates of respondents' parents. Table 5 lists them together with our estimates of the corresponding probabilities of a "yes" answer. The unrelated questions were randomly paired with the sensitive items for each respondent.

As in all sensitive questions conditions other than direct questioning, respondents were shown a screen before the first sensitive item that announced the use of a special technique in order to protect their privacy. The particular sensitive question technique procedure was

---

requesters. Hence, a HIT rejection damages a worker's reputation and might preclude him from some future HITs.

Table 5: Unrelated questions for CM Question

No.	Unrelated question	Probability
1	Is your mother's birthday in January or February?	0.159
2	Is your mother's birthday between the 1st and the 6th of the month (including the 1st and 6th)?	0.197
3	Is your father's birthday in January or February?	0.159
4	Is your father's birthday between the 1st and the 6th of the month (including the 1st and 6th)?	0.197

Note.– Probabilites calculated assuming a uniform birth distribution.

outlined and a short explanation on how the technique protects individual answers was given (see questionnaire in Appendix A.2).

2.2.3 UQ Benford

In the UQ Benford condition an unrelated-question RRT design as proposed in Diekmann (2012) was used. Respondents were asked to think of an acquaintance and use the first digit of this person's house number as their personal random number (figure 6a). If their random digit was 1, 2, 3, 4, or 5, respondents then had to answer the subsequent sensitive questions; otherwise they had to answer the subsequent unrelated non-sensitive questions (see figure 6b). Diekmann (2012) provides evidence that first digits of house numbers elicited by respondents this way follow "Benford's Law". Accordingly, the probability of 1, 2, 3, 4, or 5 (i.e., of having to answer the sensitive questions) is 0.778.

The non-sensitive unrelated questions we used are listed in table 6, together with the corresponding probabilities that the questions are answered with "yes". The unrelated questions were randomly paired with the sensitive items for each respondent.

2.2.4 FR Number

FR Number is a forced-response variant of the RRT (Boruch 1971, Greenberg et al. 1969) using a novel randomizing device called pick-a-number (Höglinger et al. 2014). The device works as follows: Respondents are presented twelve fields on the screen, numbered from one to twelve. They are told to privately choose a field and memorize their choice (without clicking on it). Then, they are told to click the "Show instruction" button to uncover the instructions hidden within the fields and follow the instruction that appears in the field of their choice (see figure 7). Possible instructions were "Answer question", "Directly tick Yes", or "Directly tick No". The distribution of the different instructions was two times "Directly tick Yes", once "Directly tick No", and nine times "Answer question" for the items on shoplifting and tax evasion. To keep

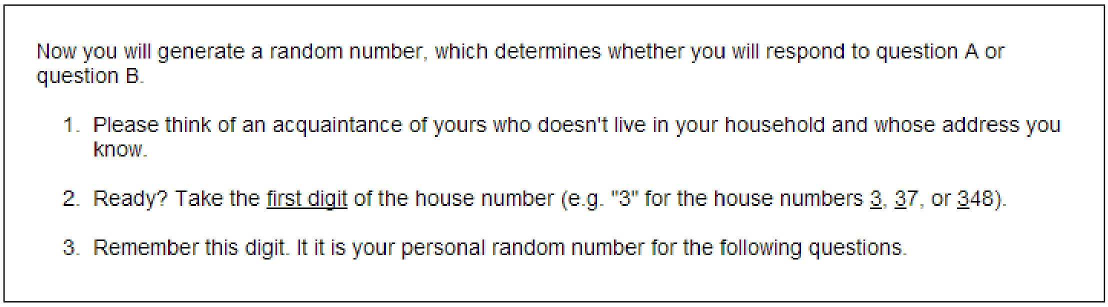

Now you will generate a random number, which determines whether you will respond to question A or question B.

1. Please think of an acquaintance of yours who doesn't live in your household and whose address you know.
2. Ready? Take the first digit of the house number (e.g. "3" for the house numbers 3, 37, or 348).
3. Remember this digit. It it is your personal random number for the following questions.

(a) Benford procedure

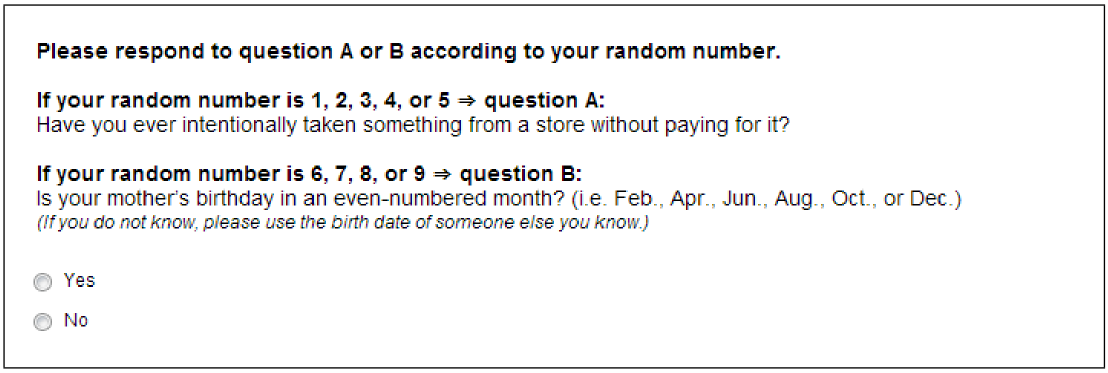

Please respond to question A or B according to your random number.

If your random number is 1, 2, 3, 4, or 5 ⇒ question A:
Have you ever intentionally taken something from a store without paying for it?

If your random number is 6, 7, 8, or 9 ⇒ question B:
Is your mother's birthday in an even-numbered month? (i.e. Feb., Apr., Jun., Aug., Oct., or Dec.)
(If you do not know, please use the birth date of someone else you know.)

Yes

No

(b) Answering sensitive or unrelated non-sensitive question

Figure 6: UQ Benford

Table 6: Unrelated questions for UQ Benford

No.	Unrelated question	Probability
1	Is your mother's birthday in the months of January through June?	0.521
2	Is your mother's birthday in an even-numbered month? (Feb., Apr., Jun., Aug., Oct., Dec.)	0.495
3	Is your mother's birthday in the first half of the month? (from 1st to 15th)	0.493
4	Is your mother's birthday on an even-numbered day? (2nd, 4th, 6th, etc. of the month)	0.490
5	Is your mother's birth year even-numbered? (Please, consider 0 as an even number.)	0.5

Note.– Probabilites calculated assuming a uniform birth distribution.

Figure 7: FR Number with uncovered random instructions

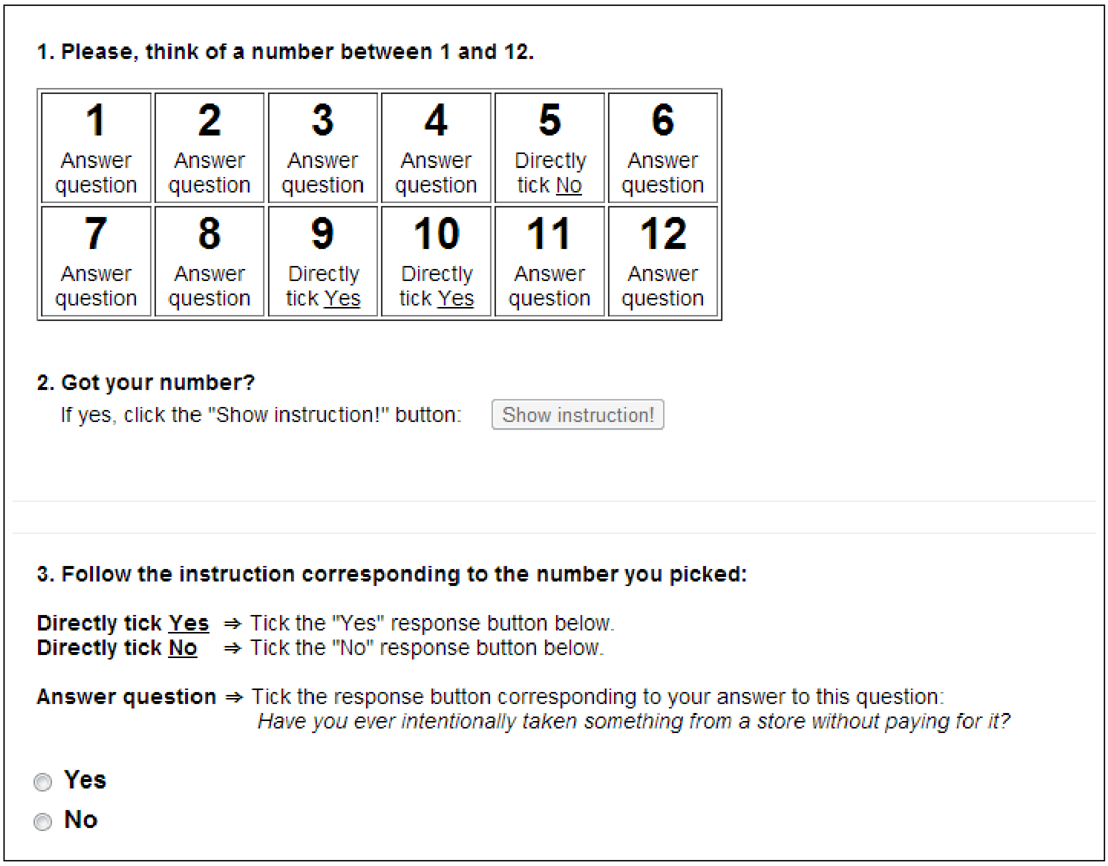

the design equal for the items on voting and honest dice-game reporting, where "No" and not "Yes" is the sensitive response, the probabilities of "Directly tick Yes" and "Directly tick No" were interchanged. The pick-a-number fields were implemented using JavaScript (authored by Philip Tschiemer, see Appendix A.3 for the code). To avoid bias due to respondents' preference for particular numbers, instructions were randomized across fields.

2.2.5 Design parameters

To analyze the data collected using the RRT, the corresponding design parameters must be known. For the forced-response or unrelated-question RRT there are two design parameters, the probability of a forced or unrelated "yes" (p^{yes}) and the probability of a forced or unrelated "no" (p^{no}). For the crosswise-model there is one design parameter, the probability of a "yes"-answer to the unrelated question (p^{cm}). For an overview of basic formulas for analyzing RRT data see, e.g., Krumpal et al. (2015). Regression estimators for RRT data are provided by Jann (2005, 2008).

The design parameters can be reconstructed from the descriptions of the RRT implementations above. For the conditions CM Question and FR Number the probabilities directly follow from the given information on the distribution of unrelated questions or random instructions, respectively. For UQ Benford p^{yes} and p^{no} can be computed by multiplying the probabilities

Figure 8: Test of Benford procedure

The following question is a little strange, however, please answer it carefully:

1. Please think of an acquaintance of yours who doesn't live in your household and whose address you know.
2. Ready? Take the first digit of the house number (e.g. "3" for the house numbers 3, 37, or 348).
3. What is this digit? Please, report it here:

of the personal random number and the unrelated questions. For the sake of convenience variables holding the design parameters for each sensitive item are included in the dataset. For direct questioning the parameters were set to $p^{yes} = p^{no} = 0$ and $p^{cm} = 1$, which is useful for joint analyses across experimental conditions.

2.3 Factor three: randomizing device test

In our study we also tested whether the randomizing devices used for the different RRT implementations actually produce the expected outcome distributions. Towards the end of the questionnaire we asked respondents to apply one such randomizing device and explicitly state the outcome. To avoid any interference of this test with the randomizing device employed when surveying the sensitive questions (if any), respondents received a different randomizing device than was used for the sensitive questions. For example, participants who had to answer unrelated questions for the sensitive items (conditions CM Question or UQ Benford) were not assigned to one of the randomizing device tests with unrelated questions. See figure 1 for the possible combinations of sensitive question techniques and randomizing device tests. The randomizing device test was introduced as a “strange” task without explanation of the purpose.

2.3.1 Benford procedure

In this test we evaluated whether the random digits generated by respondents actually follow a Benford distribution. Respondents were asked to indicate the first digit of a randomly selected acquaintance’s house number (see figure 8). The procedure was identical to the one used when surveying the sensitive items in the condition UQ Benford. The only exception was that respondents had to enter the resulting digit instead of memorizing it as a personal random number.

2.3.2 Unrelated questions for CM Question

To test whether the probabilities of a “yes”-answer to the unrelated questions used for CM Question correspond to the theoretical expectation we asked respondents in this condition to

Figure 9: Test of unrelated questions for CM Question (example)

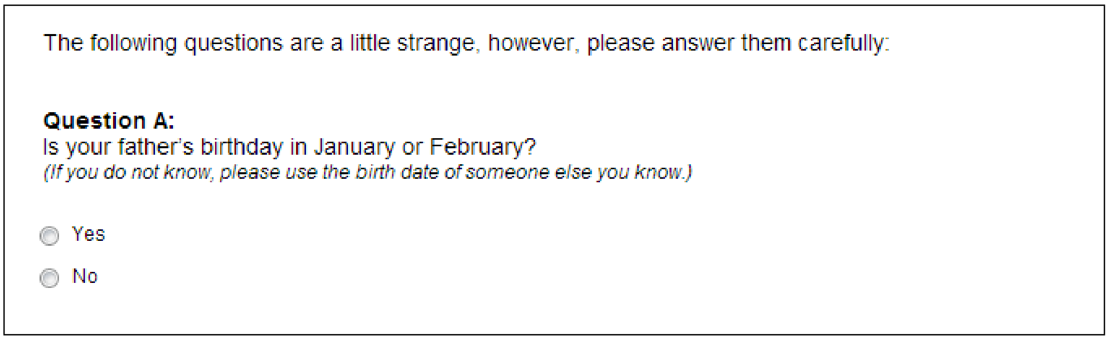
The following questions are a little strange, however, please answer them carefully:

Question A:
Is your father's birthday in January or February?
(If you do not know, please use the birth date of someone else you know.)

Yes

No

Figure 10: Test of unrelated questions for UQ Benford (example)

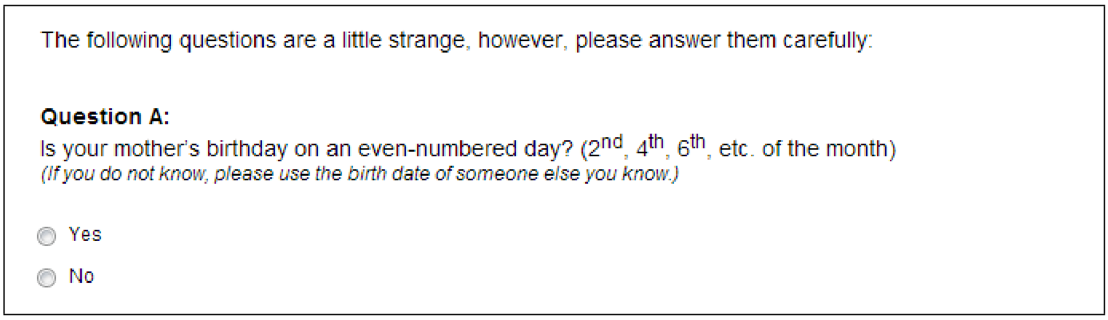
The following questions are a little strange, however, please answer them carefully:

Question A:
Is your mother's birthday on an even-numbered day? (2nd, 4th, 6th, etc. of the month)
(If you do not know, please use the birth date of someone else you know.)

Yes

No

explicitly answer these questions (see figure 9). The order of the unrelated questions was randomized for each respondent. The data set contains variables indicating the order of the unrelated questions as well as the expected probabilities of "yes"-answers.

2.3.3 Unrelated questions for UQ Benford

Respondents were asked to answer the four unrelated questions we used in UQ Benford (see figure 10). The order of the unrelated questions was randomized; the data set contains variables indicating the order of the unrelated questions as well as the expected probabilities of "yes"-answers.

2.3.4 Pick-a-number device (standard)

With this test we wanted to see whether the resulting distribution from the pick-a-number device corresponds to the theoretical expectation. The design of the randomizing device was identical to the one used in FR Number. The only exception was that instead of answering the sensitive question with a probability of of $9/12$, respondents had to tick "Other" with a probability of $9/12$ (see figure 11).

Figure 11: Test of Pick-a-number device (standard) (with uncovered fields)

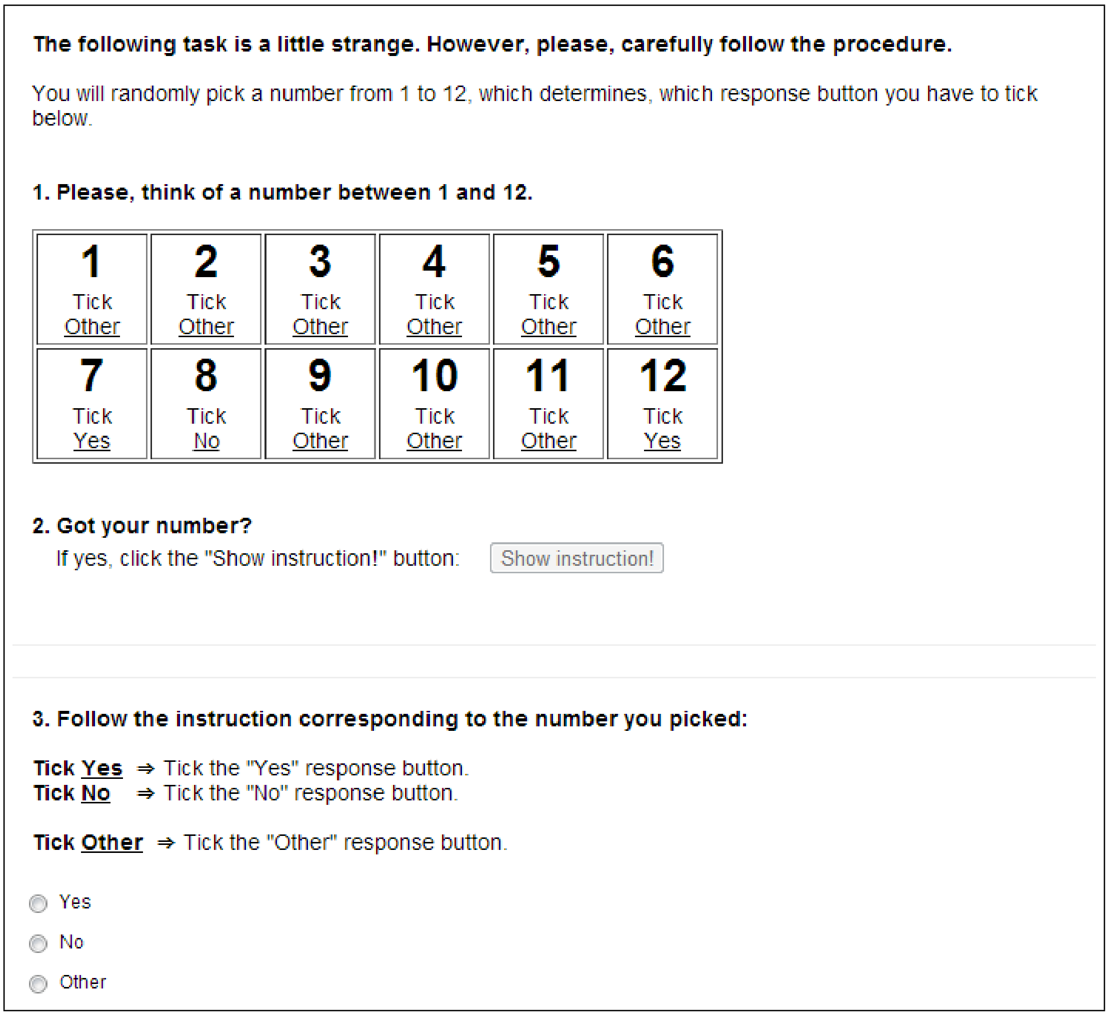

The following task is a little strange. However, please, carefully follow the procedure.

You will randomly pick a number from 1 to 12, which determines, which response button you have to tick below.

1. Please, think of a number between 1 and 12.

**1** Tick Other	**2** Tick Other	**3** Tick Other	**4** Tick Other	**5** Tick Other	**6** Tick Other
**7** Tick Yes	**8** Tick No	**9** Tick Other	**10** Tick Other	**11** Tick Other	**12** Tick Yes

2. Got your number?
If yes, click the "Show instruction!" button: Show instruction!

3. Follow the instruction corresponding to the number you picked:

Tick Yes ⇒ Tick the "Yes" response button.
Tick No ⇒ Tick the "No" response button.

Tick Other ⇒ Tick the "Other" response button.

- Yes
- No
- Other

Figure 12: Test of Pick-a-number device (generic) (with uncovered fields)

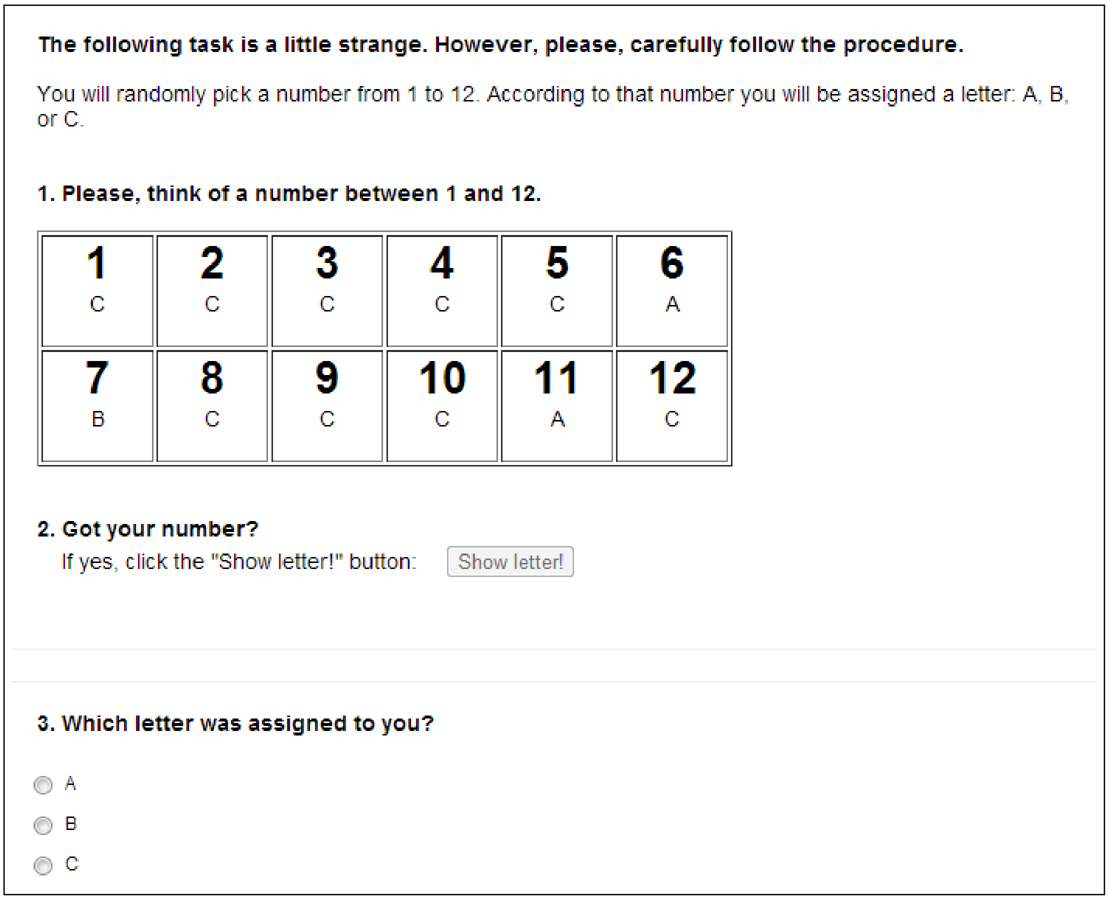

2.3.5 Pick-a-number device (generic)

This test is a slightly altered version of the pick-a-number device test. It has neutral response categories “A”, “B”, and “C” instead of “Yes”, “No”, and “Other”. Respondents followed the usual procedure and had to indicate the letter that was assigned to them (see figure 12).

References

Berinsky, Adam J., Michele Margolis, and Michael W. Sances. 2012. "Separating the Shirkers from the Workers? Making Sure Respondents Pay Attention on Internet Surveys." In *NYU CESS 5th Annual Experimental Political Science Conference*.

Boruch, Robert F. 1971. "Assuring Confidentiality of Responses in Social Research: A Note on Strategies." *The American Sociologist* 6:308–311.

Coutts, Elisabeth and Ben Jann. 2011. "Sensitive Questions in Online Surveys: Experimental Results for the Randomized Response Technique (RRT) and the Unmatched Count Technique (UCT)." *Sociological Methods & Research* 40:169–193.

Diekmann, Andreas. 2012. "Making Use of "Benford's Law" for the Randomized Response Technique." *Sociological Methods & Research* 41:325–334.

Dohmen, Thomas, Armin Falk, David Huffman, Uwe Sunde, Jürgen Schupp, and Gert G. Wagner. 2005. "Individual Risk Attitudes: New Evidence from a Large, Representative, Experimentally-Validated Survey." , DIW Berlin, German Institute for Economic Research. Discussion Papers of DIW Berlin.

Fischbacher, Urs and Franziska Heusi. 2008. "Lies in Disguise. An experimental study on cheating." , Thurgau Institute of Economics and Department of Economics at the University of Konstanz.

Greenberg, Bernard G., Abdel-Latif A. Abul-Ela, Walt R. Simmons, and Daniel G. Horvitz. 1969. "The unrelated question randomized response model: Theoretical Framework." *Journal of the American Statistical Association* 64:520–539.

Greene, Joshua D. and Joseph M. Paxton. 2009. "Patterns of neural activity associated with honest and dishonest moral decisions." *Proceedings of the National Academy of Sciences* 106:12506–12511.

Höglinger, Marc, Ben Jann, and Andreas Diekmann. 2014. "Online Survey on "Exams and Written Papers", Documentation." , ETH Zurich and University of Bern.

Horton, John, David Rand, and Richard Zeckhauser. 2011. "The online laboratory: conducting experiments inÂ aÂ realÂ laborÂ market." *Experimental Economics* 14:399–425.

Jann, Ben. 2005. "rrlogit: Stata module to estimate logistic regression for randomized response data." , Boston College Department of Economics.

Jann, Ben. 2008. "rrreg: Stata module to estimate linear probability model for randomized response data." , Boston College Department of Economics.

Jann, Ben, Julia Jerke, and Ivar Krumpal. 2011. "Asking Sensitive Questions Using the Crosswise Model: Some Experimental Results." *Public Opinion Quarterly* 75:1–18.

John, Leslie K., George Loewenstein, Alessandro Acquisti, and Joachim Vosgerau. 2013. "Paradoxical Effects of Randomized Response Techniques."

Kreuter, Frauke, Stanley Presser, and Roger Tourangeau. 2008. "Social Desirability Bias in CATI, IVR, and Web Surveys." *Public Opinion Quarterly* 72:847–865.

Krumpal, Ivar, Ben Jann, Kathrin Auspurg, and Hagen von Hermanni. 2015. "Asking Sensitive Questions: A Critical Account of the Randomized Response Technique and Related Methods." In *Improving Survey Methods: Lessons from Recent Research*, edited by Uwe Engel, Ben Jann, Peter Lynn, Annette Scherpenzeel, and Patrick Sturgis, pp. 122–136. Routledge.

Mason, Winter and Siddharth Suri. 2012. "Conducting behavioral research on Amazon's Mechanical Turk." *Behavior Research Methods* 44:1–23.

Paolacci, Gabriele, Jesse Chandler, and Panagiotis G. Ipeirotis. 2010. "Running experiments on Amazon Mechanical Turk." *Judgment and Decision Making* 5:411–419.

Qualtrics Labs Inc. 2012. "Qualtrics Survey Software. Handbook for Research Professionals."

Rammstedt, Beatrice and Oliver P. John. 2007. "Measuring personality in one minute or less: A 10-item short version of the Big Five Inventory in English and German." *Journal of Research in Personality* 41:203–212.

Stirnemann, Philipp. 2009. "Unmatched Count Technik: Zusammenhang zwischen Anonymität und statistischer Effizienz." , Seminar für Statistik der ETH Zürich und Professur für Soziologie der ETH Zürich.

Suri, Siddhartha, Daniel G. Goldstein, and Winter A. Mason. 2011. "Honesty in an Online Labor Market." *Human Computation: Papers from the 2011 AAAI Workshop (WS-11-11)* .

Warner, Stanley L. 1965. "Randomized-response: A survey technique for eliminating evasive answer bias." *Journal of the American Statistical Association* 60:63–69.

A Appendix

A.1 HIT details

Title	Survey on mood and personality ($1 + chance for $2 bonus)
Description	Fill in a survey on mood and personality (8 minutes)
Reward	1 (Dollar)
Keywords	survey, study, research, psychology, social science, experiment
Instructions	**Participation:** Please accept this HIT only if you have *** not *** already participated in a HIT called "Survey on mood and personality" (2013). This task must not be done on a smartphone or tablet/iPad. You need JavaScript enabled in your browser.<br>**What you will do:** If you decide to participate, you will complete a short scientific survey. There are no right or wrong answers but is important that you take the survey seriously and respond truthfully.<br>**Time required:** The study will take approximately 8 minutes to complete.<br>**Risks:** There are no anticipated risks associated with participating in this study and your participation will remain anonymous.<br>**Compensation:** You will receive $1 for participation and have the chance to win a $2 bonus payment. It is important that you follow the guidelines for participation and answer the survey questions carefully. We screen for random responses.

A.2 Questionnaire

Page 1: Starting page (prediction game)

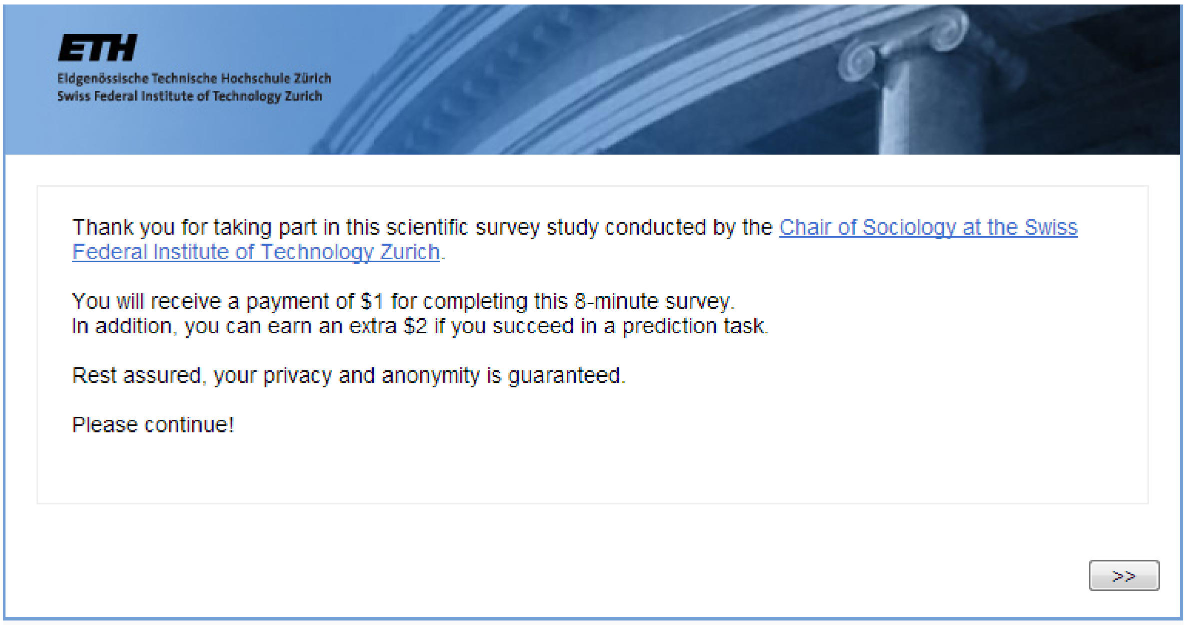

Page 1: Starting page (roll-a-six game)

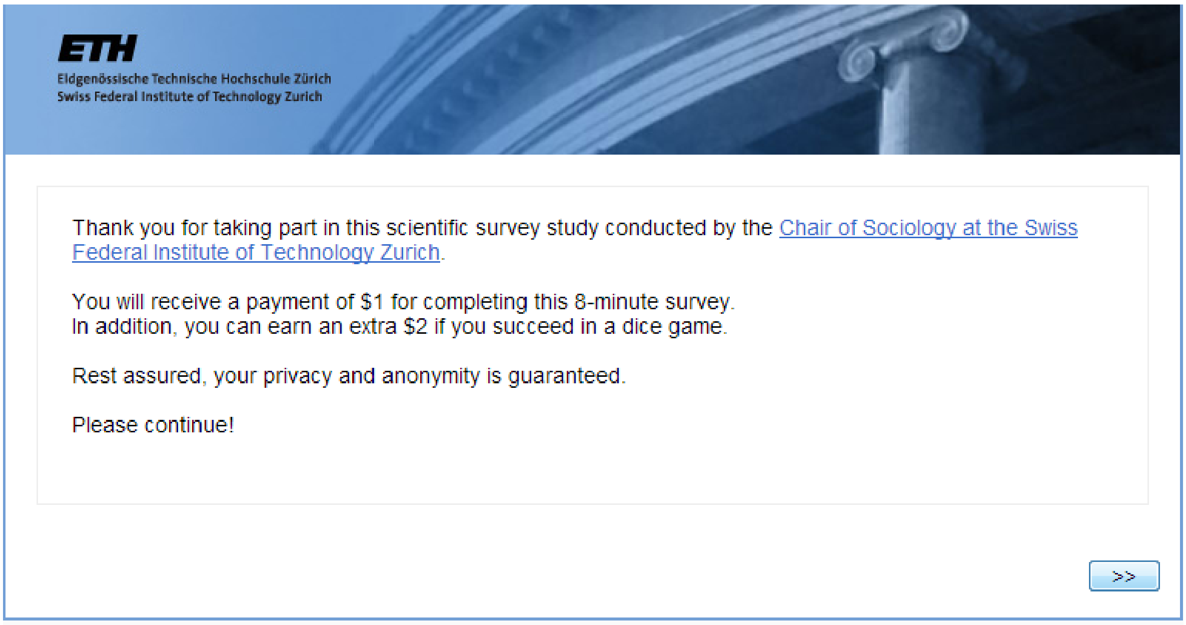

Page 2: Screening question

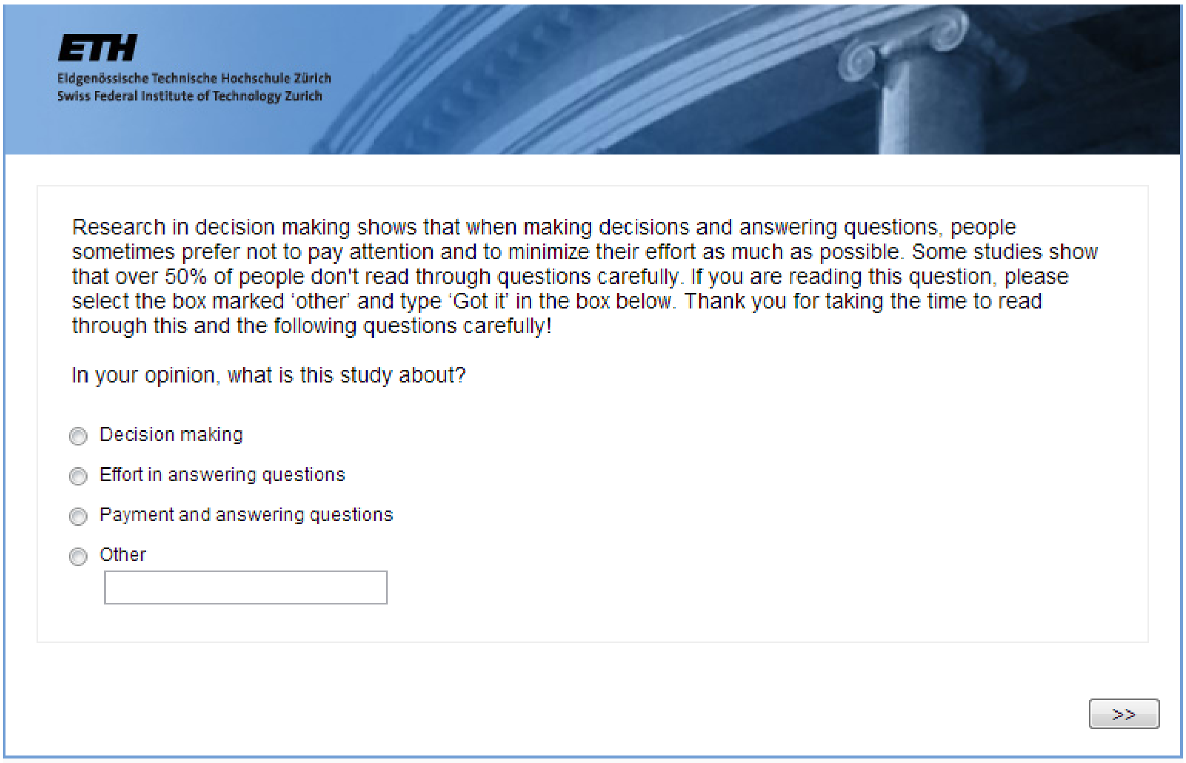

ETH
Eidgenössische Technische Hochschule Zürich
Swiss Federal Institute of Technology Zurich

Research in decision making shows that when making decisions and answering questions, people sometimes prefer not to pay attention and to minimize their effort as much as possible. Some studies show that over 50% of people don't read through questions carefully. If you are reading this question, please select the box marked ‘other’ and type ‘Got it’ in the box below. Thank you for taking the time to read through this and the following questions carefully!

In your opinion, what is this study about?

- Decision making
- Effort in answering questions
- Payment and answering questions
- Other

>>

Page 3: Personal background I

ETH
Eidgenössische Technische Hochschule Zürich
Swiss Federal Institute of Technology Zurich

How do you see yourself: Are you generally a person who is fully prepared to take risks or do you try to avoid taking risks?

unwilling to take risks 0	1	2	3	4	5	6	7	8	9	fully prepared to take risks 10
○	○	○	○	○	○	○	○	○	○	○

Is English your native language?

○ Yes

○ No

Are you a US citizen?

○ Yes

○ No

>>

Page 4: Intro dice game (prediction game)

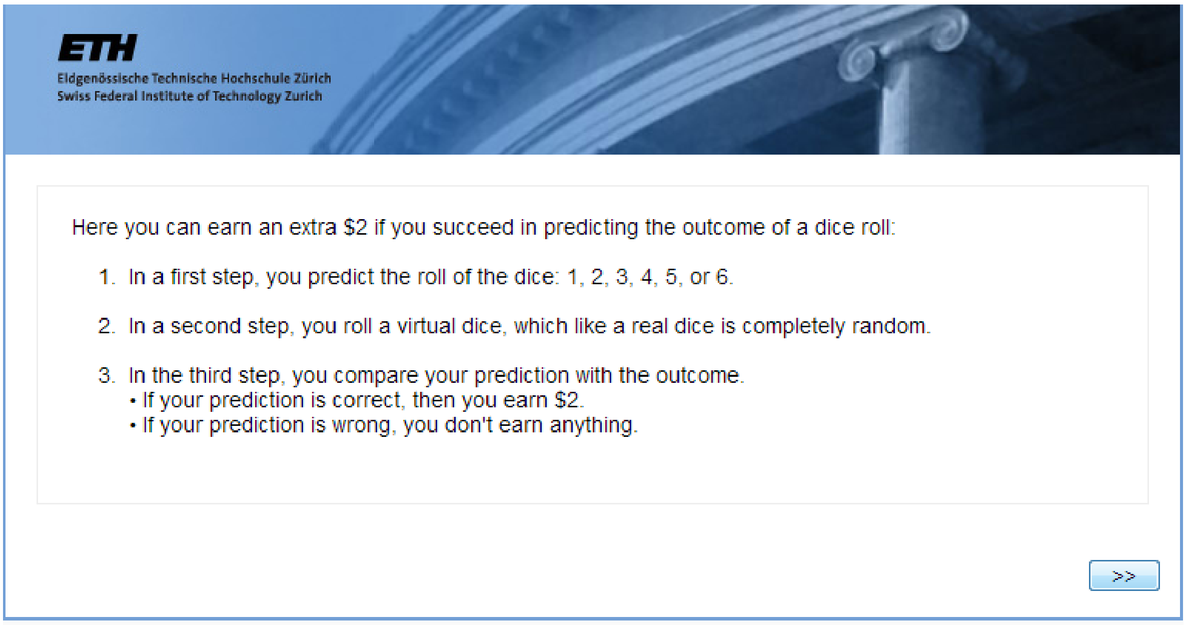

Page 4: Intro dice game (roll-a-six game)

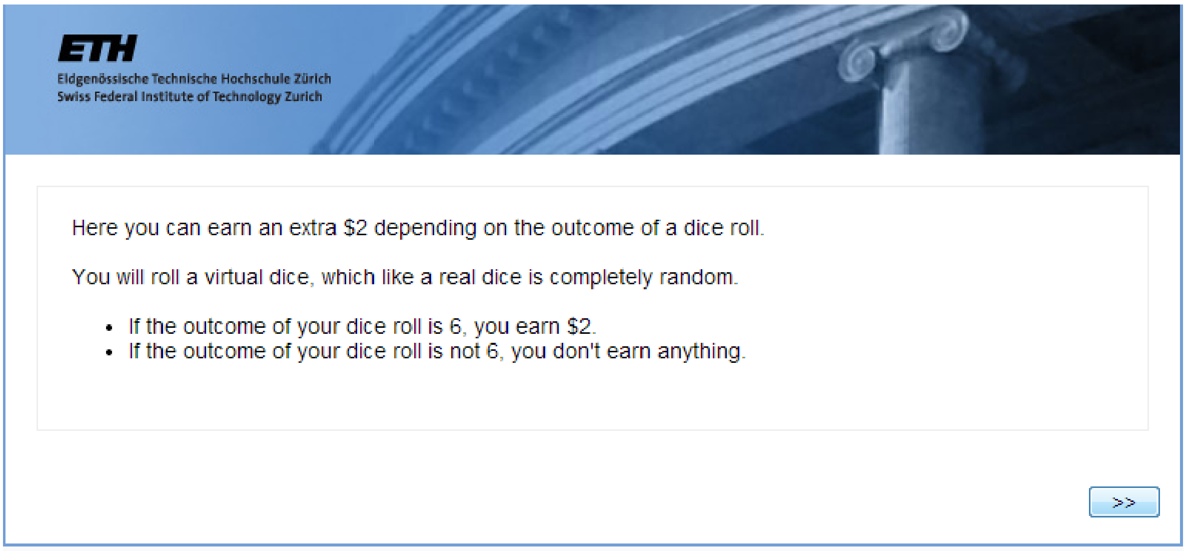

Page 5: Prediction dice game (prediction game)

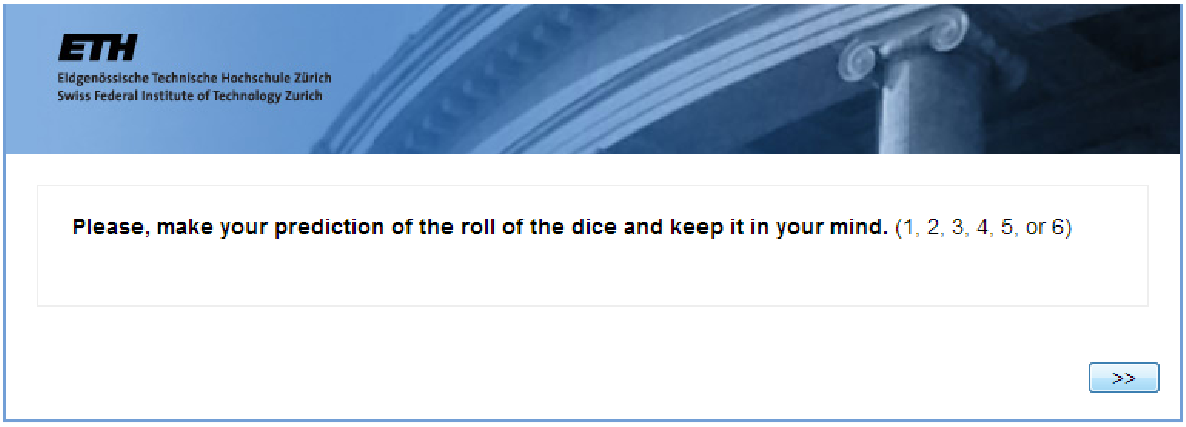

Page 6: Dice rolling (prediction game)

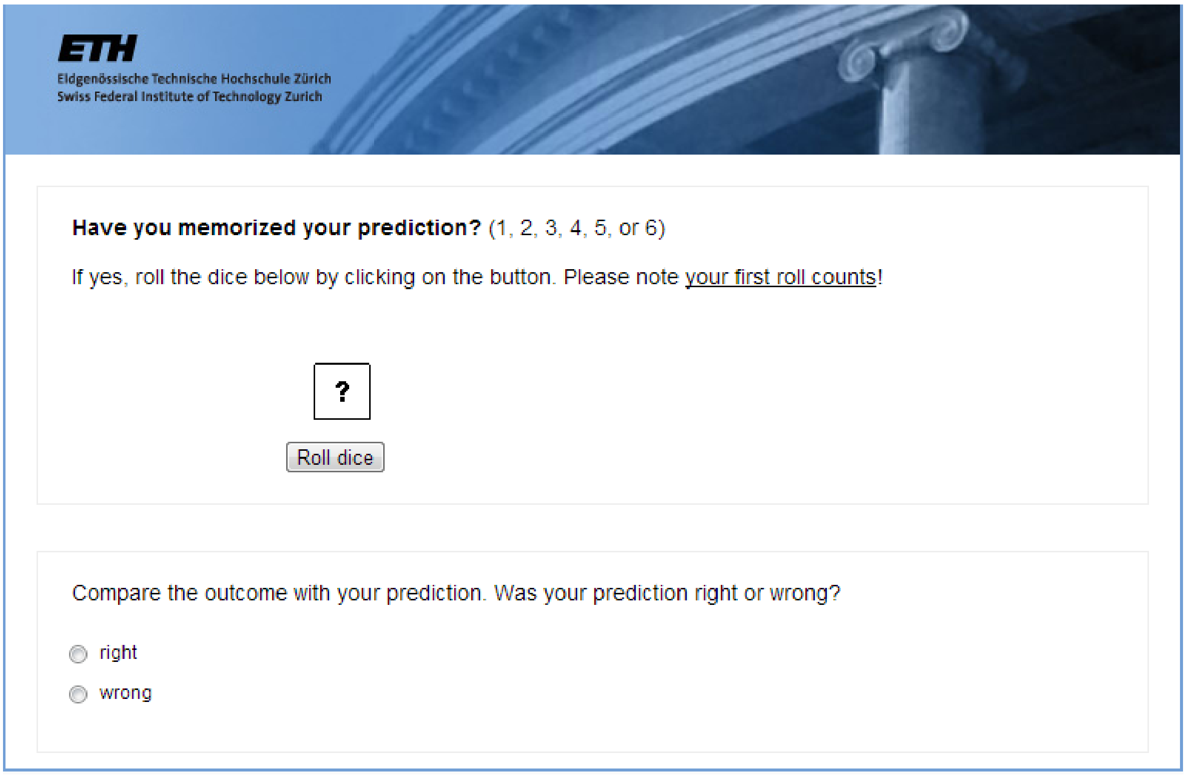

Page 6: Dice rolling (roll-a-six game)

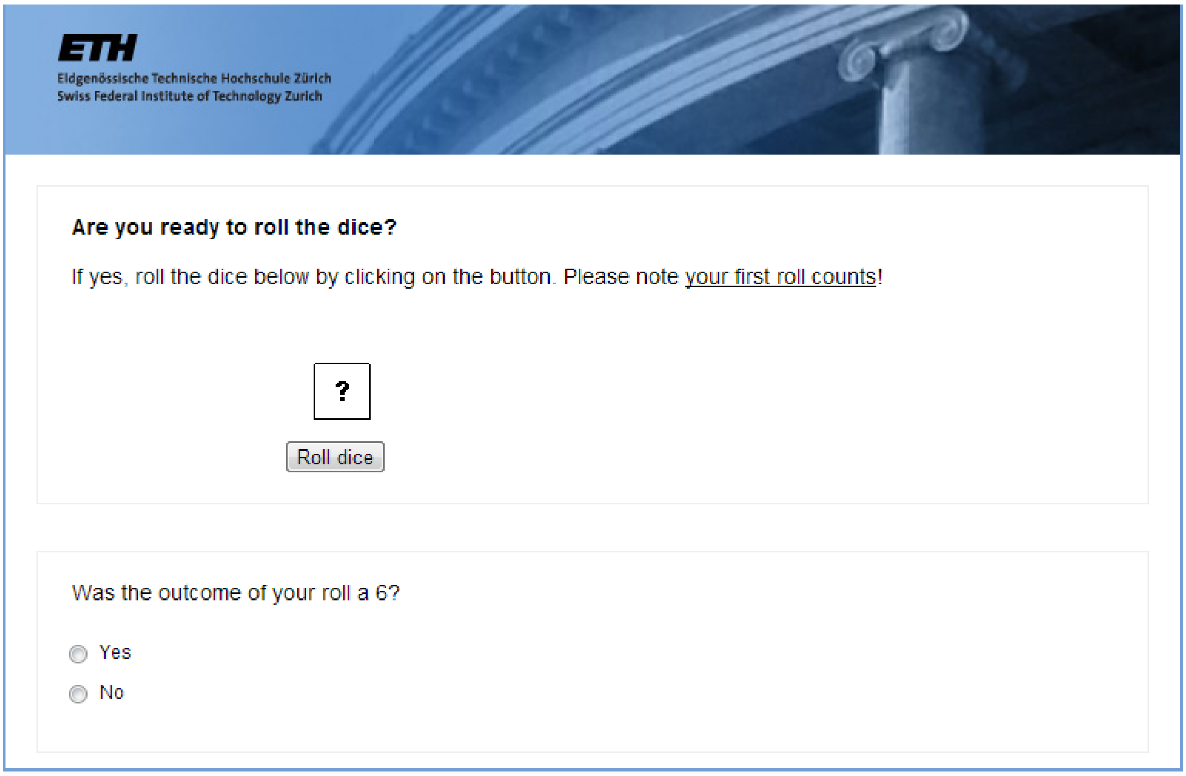

ETH
Eidgenössische Technische Hochschule Zürich
Swiss Federal Institute of Technology Zurich

Are you ready to roll the dice?

If yes, roll the dice below by clicking on the button. Please note <u>your first roll counts</u>!

?

Roll dice

Was the outcome of your roll a 6?

- Yes
- No

Page 7: Satisfaction (prediction game)

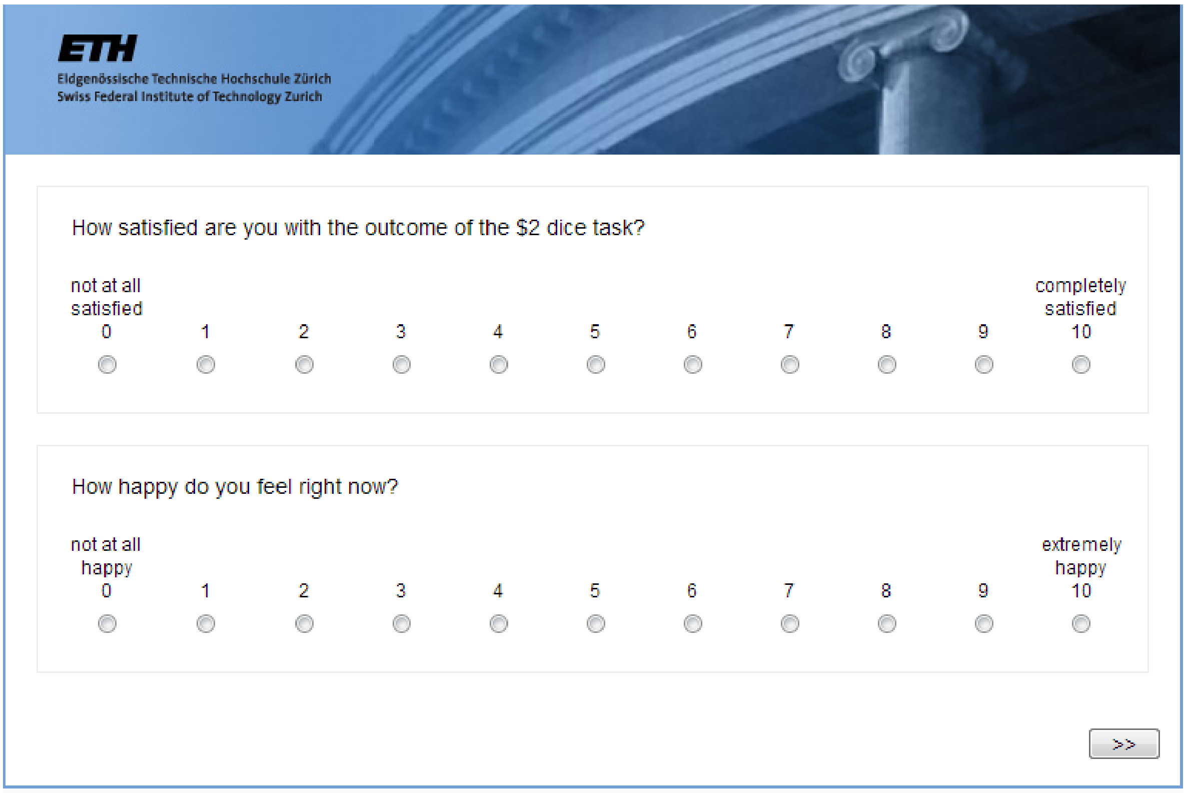

ETH
Eidgenössische Technische Hochschule Zürich
Swiss Federal Institute of Technology Zurich

How satisfied are you with the outcome of the $2 dice task?

not at all satisfied 0	1	2	3	4	5	6	7	8	9	completely satisfied 10
○	○	○	○	○	○	○	○	○	○	○

How happy do you feel right now?

not at all happy 0	1	2	3	4	5	6	7	8	9	extremely happy 10
○	○	○	○	○	○	○	○	○	○	○

>>

Page 7: Satisfaction (roll-a-six game)

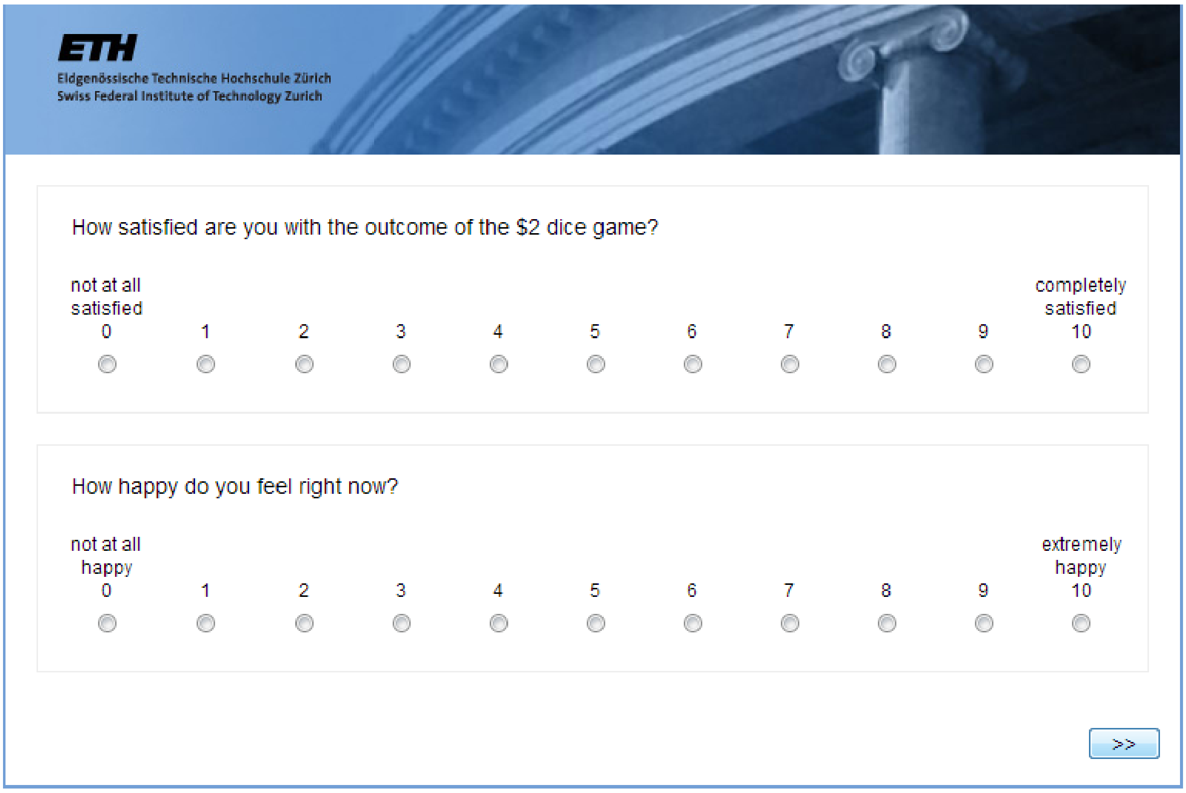
ETH
Eidgenössische Technische Hochschule Zürich
Swiss Federal Institute of Technology Zurich

How satisfied are you with the outcome of the $2 dice game?

not at all satisfied 0	1	2	3	4	5	6	7	8	9	completely satisfied 10
○	○	○	○	○	○	○	○	○	○	○

How happy do you feel right now?

not at all happy 0	1	2	3	4	5	6	7	8	9	extremely happy 10
○	○	○	○	○	○	○	○	○	○	○

>>

Page 8: Big Five

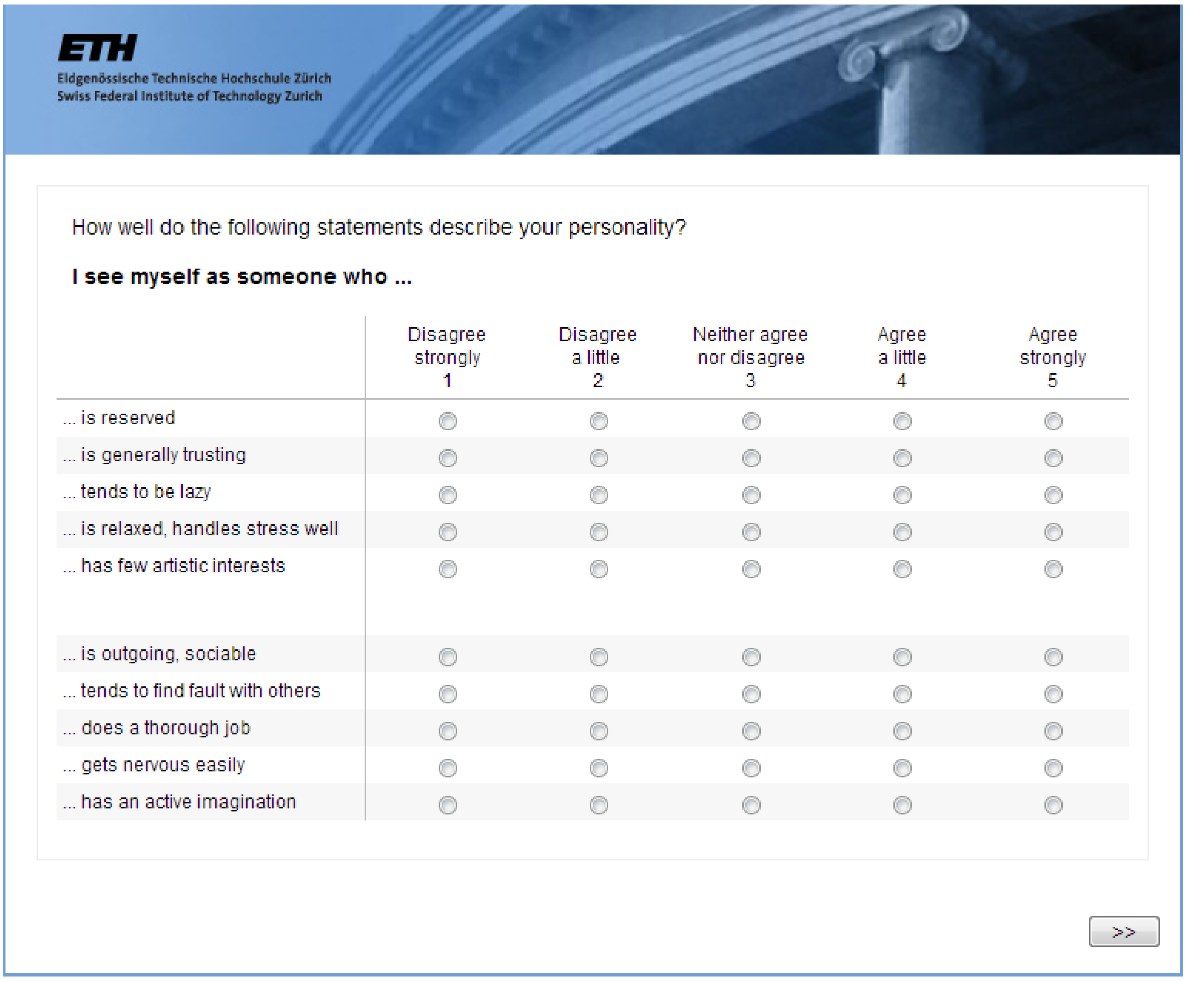
ETH
Eidgenössische Technische Hochschule Zürich
Swiss Federal Institute of Technology Zurich

How well do the following statements describe your personality?

I see myself as someone who ...

	Disagree strongly 1	Disagree a little 2	Neither agree nor disagree 3	Agree a little 4	Agree strongly 5
... is reserved	○	○	○	○	○
... is generally trusting	○	○	○	○	○
... tends to be lazy	○	○	○	○	○
... is relaxed, handles stress well	○	○	○	○	○
... has few artistic interests	○	○	○	○	○
... is outgoing, sociable	○	○	○	○	○
... tends to find fault with others	○	○	○	○	○
... does a thorough job	○	○	○	○	○
... gets nervous easily	○	○	○	○	○
... has an active imagination	○	○	○	○	○

>>

Page 9: Personal background II

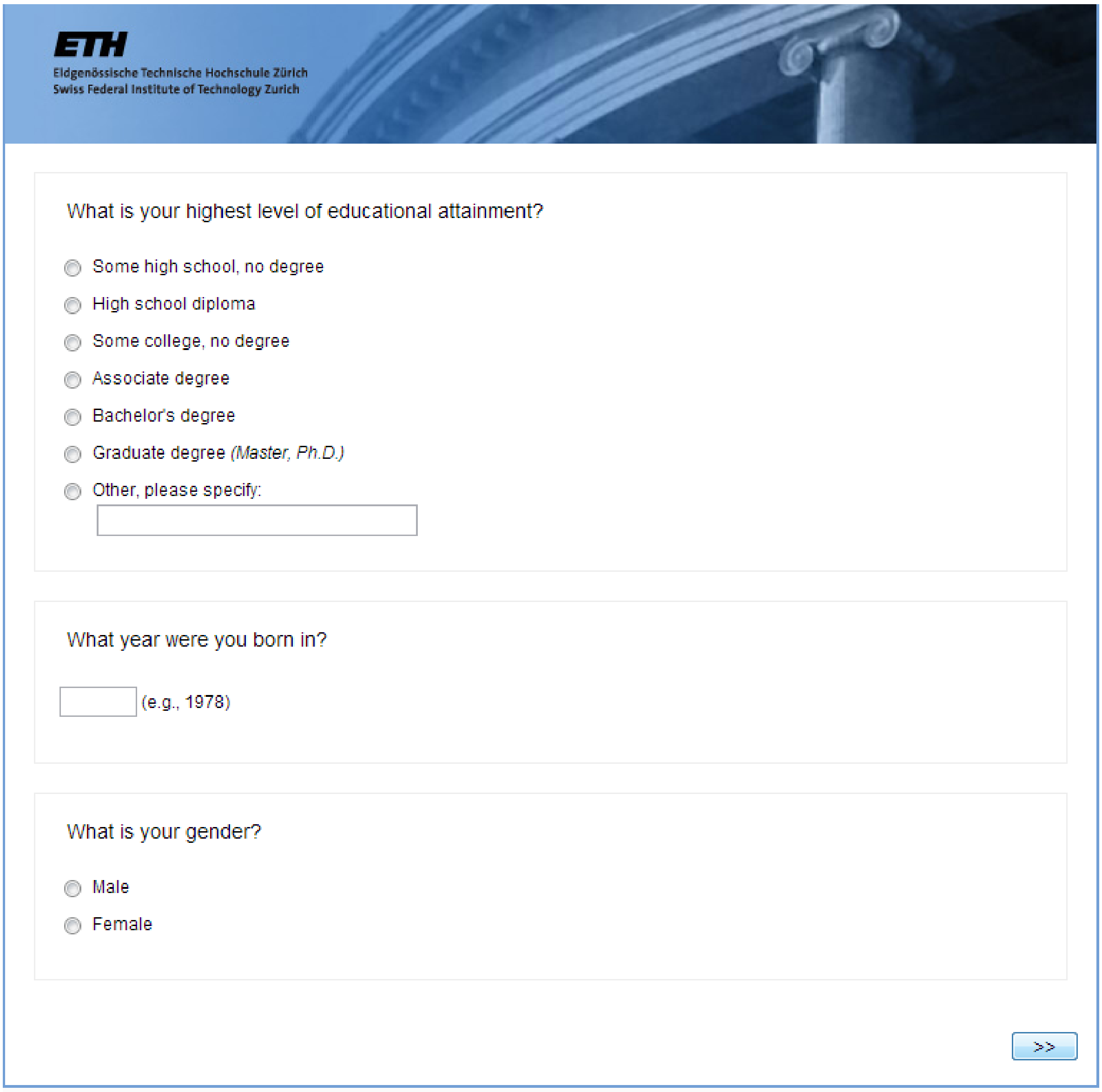

ETH
Eidgenössische Technische Hochschule Zürich
Swiss Federal Institute of Technology Zurich

What is your highest level of educational attainment?

- Some high school, no degree
- High school diploma
- Some college, no degree
- Associate degree
- Bachelor's degree
- Graduate degree *(Master, Ph.D.)*
- Other, please specify:

What year were you born in?

(e.g., 1978)

What is your gender?

- Male
- Female

>>

Page 10: MTurk and other employment

ETH
Eidgenössische Technische Hochschule Zürich
Swiss Federal Institute of Technology Zurich

How many times have you participated in scientific studies such as surveys or experiments on MTurk? *(excluding this one)*

Are you currently...

- employed
- self-employed
- out of work and looking for work
- a homemaker
- a student
- retired
- other, please specify:

Where are you right now?

- at home
- at your workplace/office
- in a cafe or restaurant
- at school/university
- travelling (car, bus, subway, train, etc.)
- other, please specify:

>>

Page 11: Intro sensitive questions

ETH
Eidgenössische Technische Hochschule Zürich
Swiss Federal Institute of Technology Zurich

On the following pages we will ask you to answer some questions about your behavior.

Honest answers are essential to our research.

Answers are kept strictly confidential and will not affect your payment or HIT approval.

>>

Page 12: Explanation special technique (CM Question)

ETH
Eidgenössische Technische Hochschule Zürich
Swiss Federal Institute of Technology Zurich

To secure your privacy and to make you feel comfortable answering the following questions, we employ a special survey technique.

It works by asking you two questions, A & B, together. Question A is a question about your parents' birth date. Question B is about a sensitive topic.

Instead of responding directly to each question, you should respond to the questions jointly.
Simply indicate whether your answers to the two questions are:

- identical *(both responses "No", or both "Yes")*
- different *(one response "Yes", the other "No")*

This way we do not know your response to the sensitive question.

Because birth dates are approximately evenly distributed over the year, we can estimate the aggregate share of "Yes" and "No" responses on the sensitive topic without knowing your individual response.

>>

Page 12: Explanation special technique (UQ Benford)

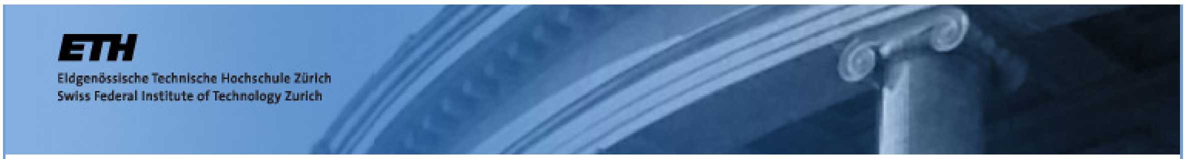

To secure your privacy and to make you feel comfortable answering the following questions, we employ a special survey technique.

It works by presenting you two questions, A & B. Question A is a question on a sensitive topic. Question B is about your mother's birth date.

A random number, generated by you, and known only to you, determines whether you have to respond to question A or question B.

This way we do not know whether you responded to the sensitive question or to the birth date question.

We can only estimate the aggregate share of "Yes" and "No" responses for all respondents.

>>

Page 12: Explanation special technique (FR Number)

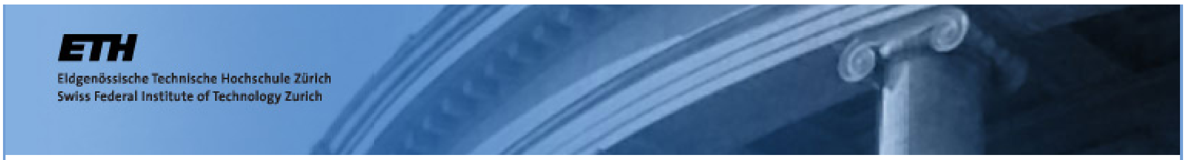

To secure your privacy and to make you feel comfortable answering the following questions, we employ a special survey technique.

It works as follows: Before responding you will randomly pick a number from 1 to 12, which determines, whether you have to:

- directly tick the "Yes" response button
- directly tick the "No" response button
- answer a sensitive question and tick the response button corresponding to your answer

We don't know whether you had to directly tick a predetermined response button or whether you answered the sensitive question, because you alone know the number you have picked and the corresponding instruction.

As such, a "Yes" response could be the result of you directly ticking the "Yes" response button or of having answered the sensitive question "Yes". By taking into account the share of respondents that had to directly tick a predetermined response, we can estimate the aggregate share of "Yes" and "No" responses to the sensitive question without knowing your individual response.

>>

Page 13: Benford procedure (UQ Benford)

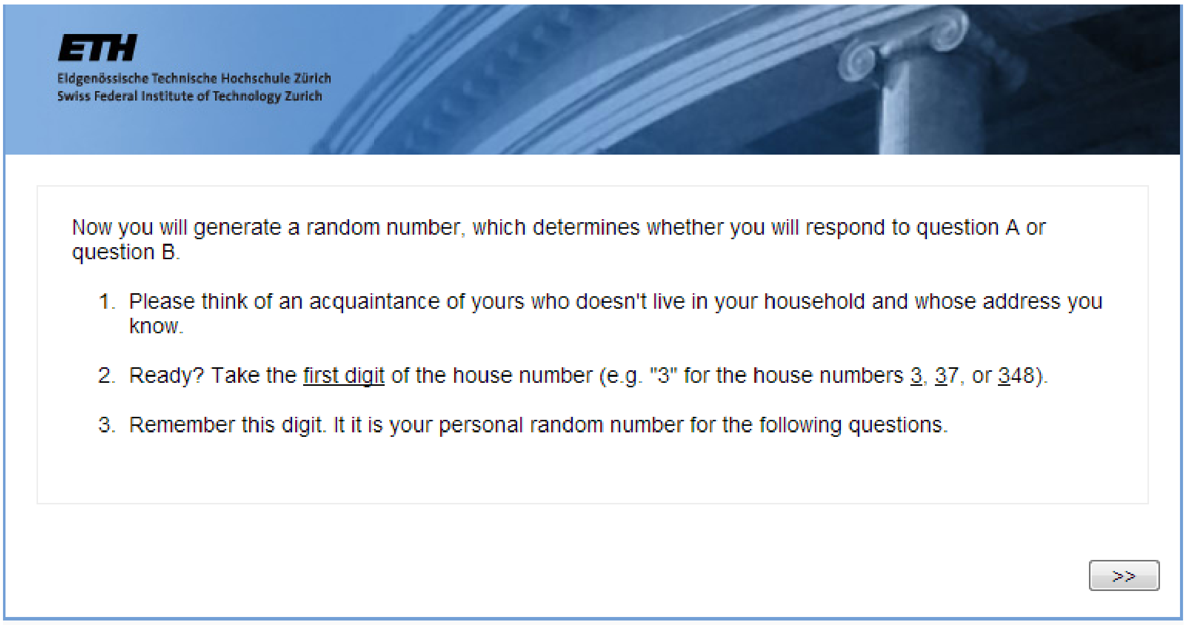

Now you will generate a random number, which determines whether you will respond to question A or question B.

1. Please think of an acquaintance of yours who doesn't live in your household and whose address you know.
2. Ready? Take the first digit of the house number (e.g. "3" for the house numbers 3, 37, or 348).
3. Remember this digit. It it is your personal random number for the following questions.

>>

Page 14: Shoplifting (Direct questioning)

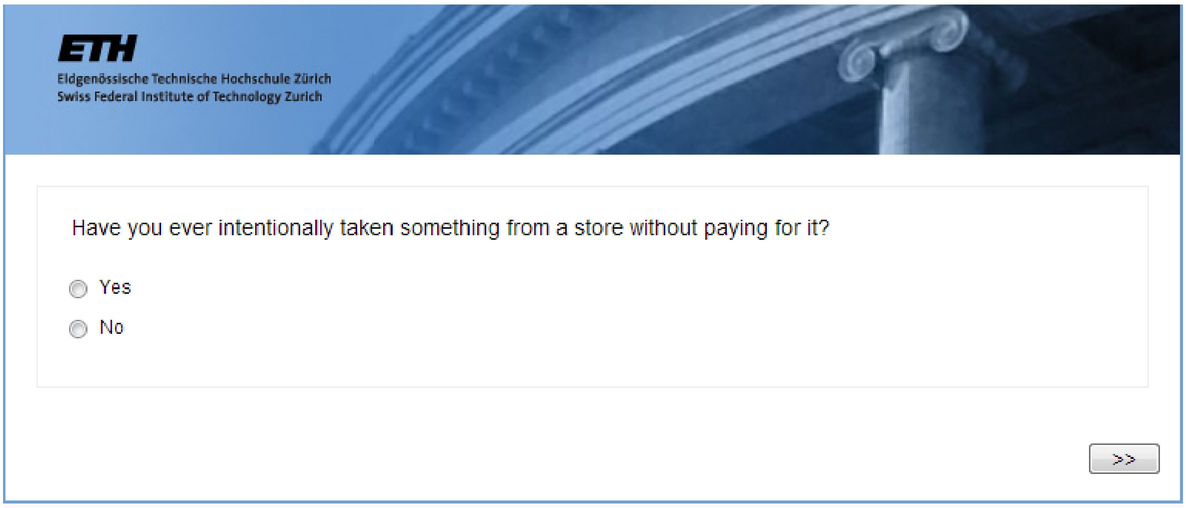

Have you ever intentionally taken something from a store without paying for it?

- Yes
- No

>>

Page 14: Shoplifting (CM Question)

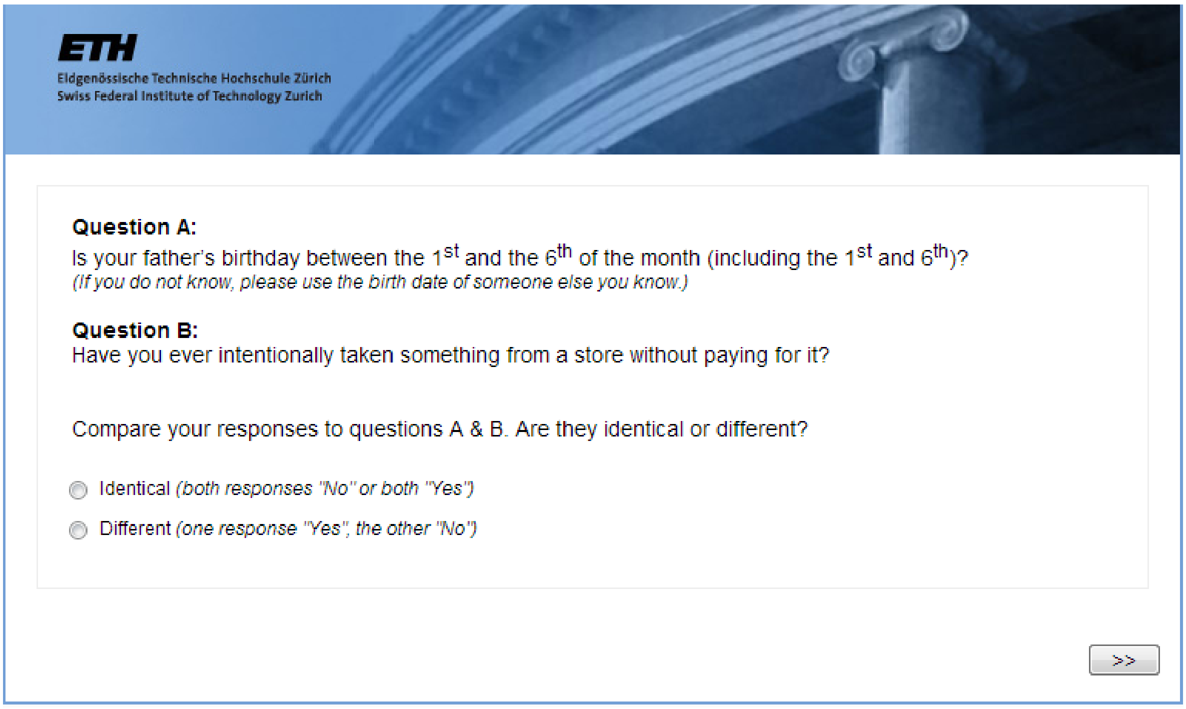

Eidgenössische Technische Hochschule Zürich
Swiss Federal Institute of Technology Zurich

Question A:
Is your father's birthday between the 1st and the 6th of the month (including the 1st and 6th)?
(If you do not know, please use the birth date of someone else you know.)

Question B:
Have you ever intentionally taken something from a store without paying for it?

Compare your responses to questions A & B. Are they identical or different?

- Identical *(both responses "No" or both "Yes")*
- Different *(one response "Yes", the other "No")*

>>

Page 14: Shoplifting (UQ Benford)

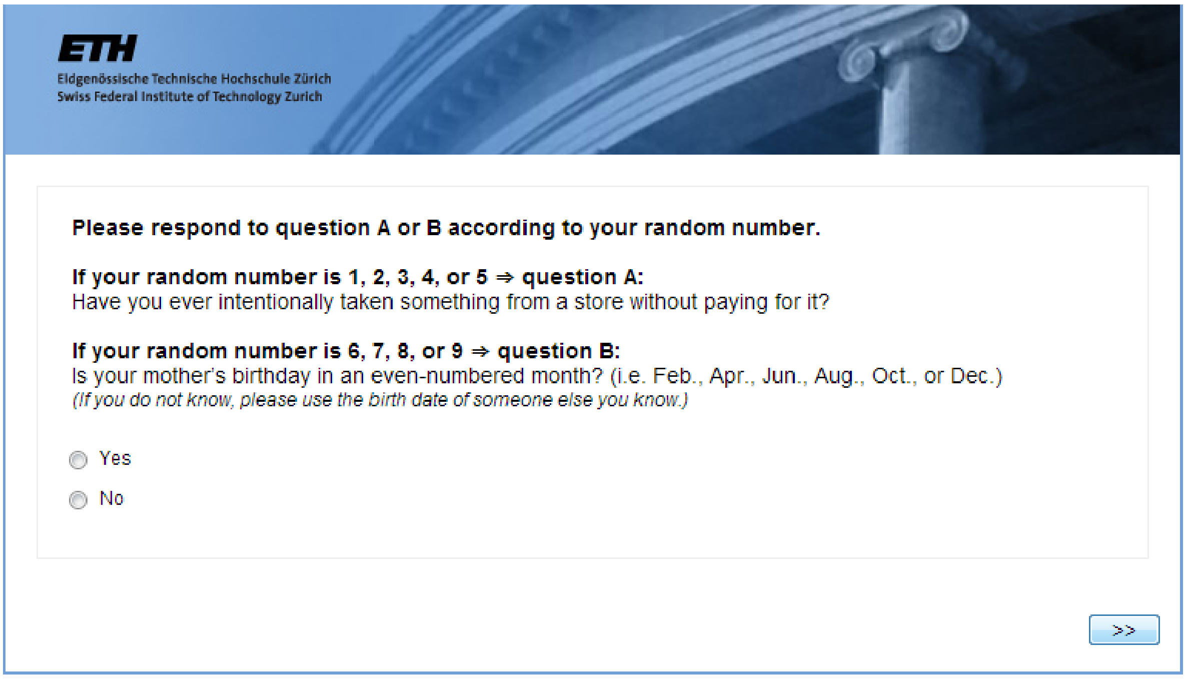

Eidgenössische Technische Hochschule Zürich
Swiss Federal Institute of Technology Zurich

Please respond to question A or B according to your random number.

If your random number is 1, 2, 3, 4, or 5 ⇒ question A:
Have you ever intentionally taken something from a store without paying for it?

If your random number is 6, 7, 8, or 9 ⇒ question B:
Is your mother's birthday in an even-numbered month? (i.e. Feb., Apr., Jun., Aug., Oct., or Dec.)
(If you do not know, please use the birth date of someone else you know.)

- Yes
- No

>>

Page 14: Shoplifting (FR Number)

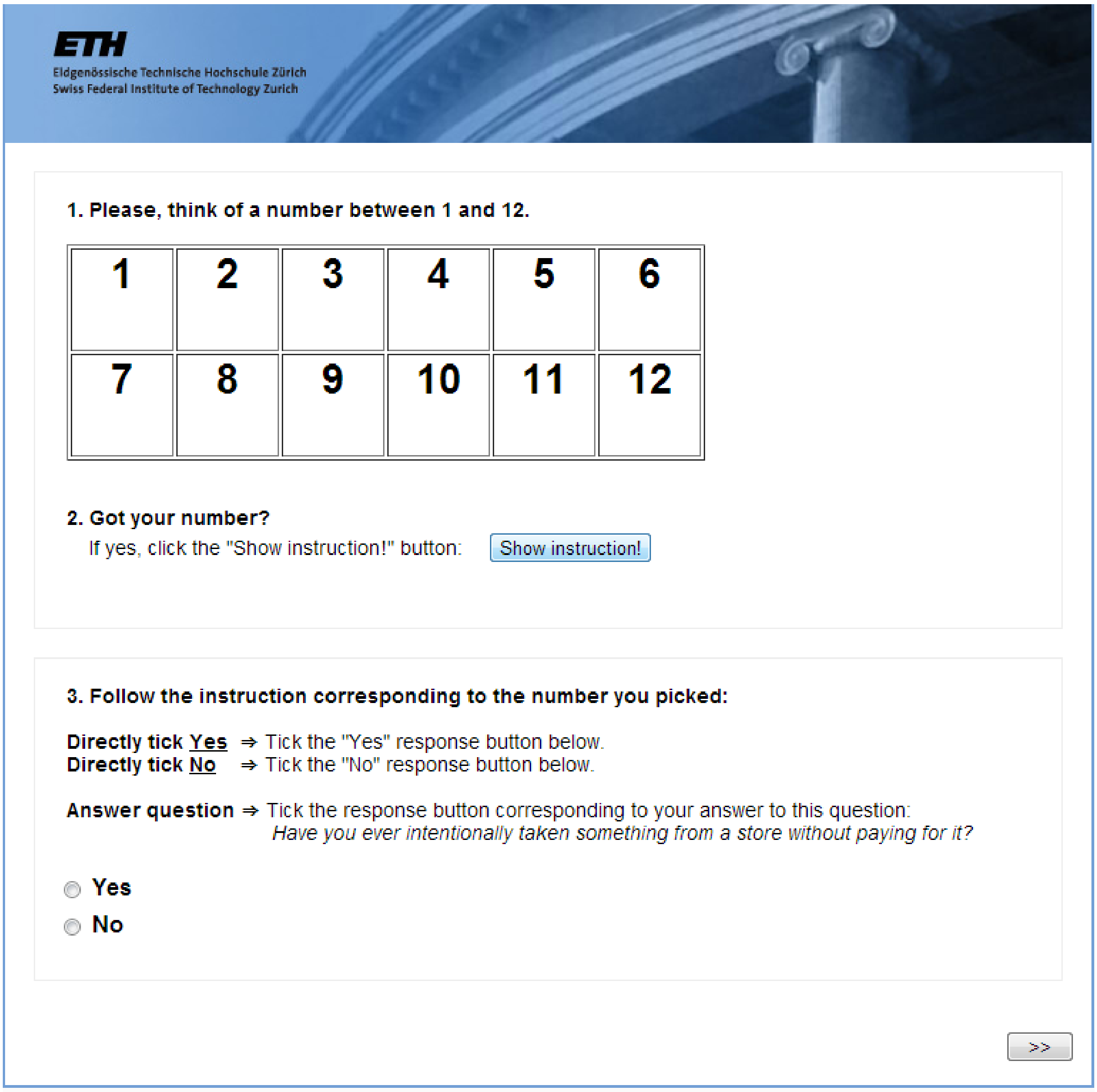

ETH
Eldgenössische Technische Hochschule Zürich
Swiss Federal Institute of Technology Zurich

1. Please, think of a number between 1 and 12.

1	2	3	4	5	6
7	8	9	10	11	12

2. Got your number?
If yes, click the "Show instruction!" button: Show instruction!

3. Follow the instruction corresponding to the number you picked:

Directly tick Yes ⇒ Tick the "Yes" response button below.
Directly tick No ⇒ Tick the "No" response button below.

Answer question ⇒ Tick the response button corresponding to your answer to this question:
Have you ever intentionally taken something from a store without paying for it?

○ Yes
○ No

>>

Page 15: Tax evasion (Direct questioning)

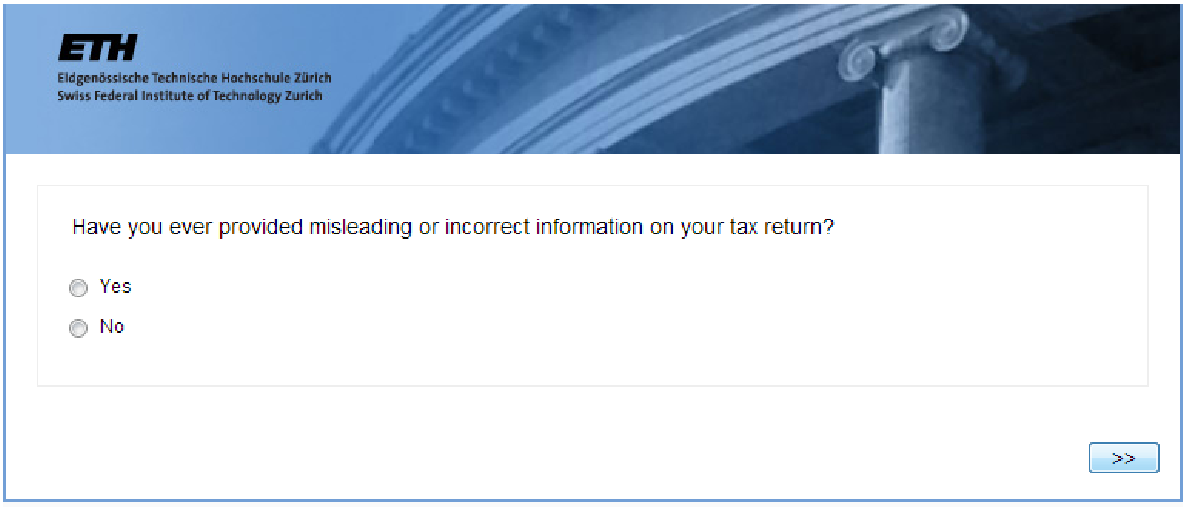

Have you ever provided misleading or incorrect information on your tax return?

- Yes
- No

>>

Page 15: Tax evasion (CM Question)

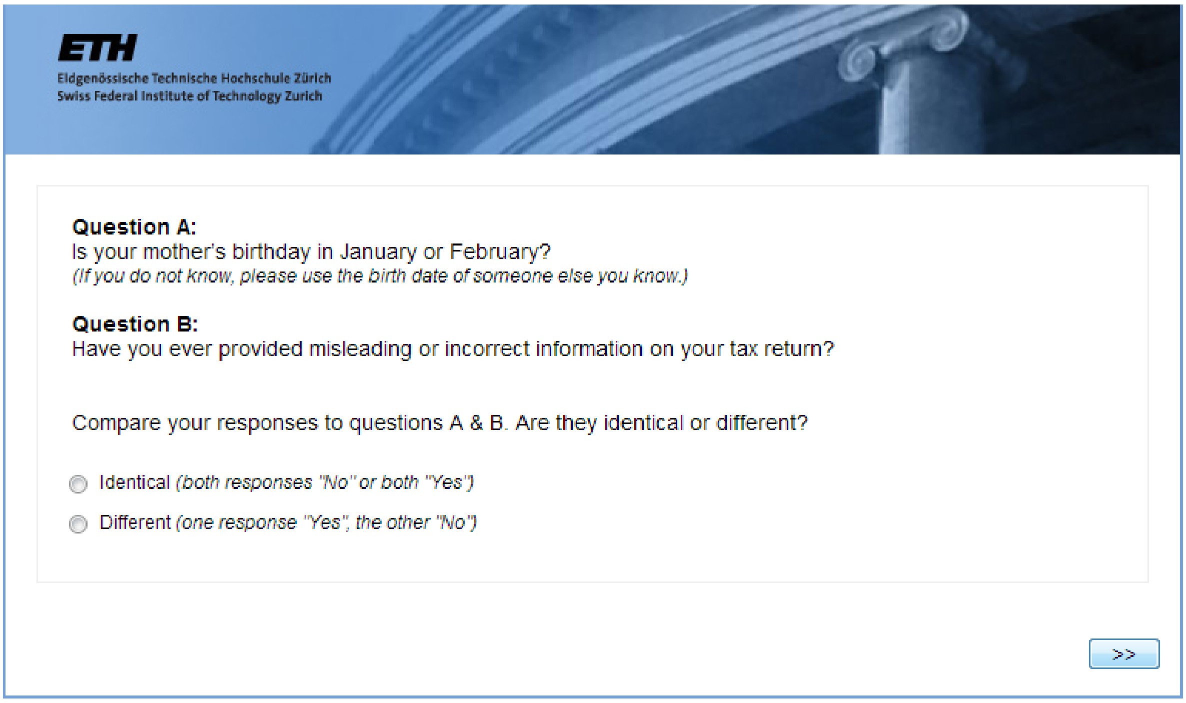

Question A:
Is your mother's birthday in January or February?
(If you do not know, please use the birth date of someone else you know.)

Question B:
Have you ever provided misleading or incorrect information on your tax return?

Compare your responses to questions A & B. Are they identical or different?

- Identical *(both responses "No" or both "Yes")*
- Different *(one response "Yes", the other "No")*

>>

Page 15: Tax evasion (UQ Benford)

ETH
Eidgenössische Technische Hochschule Zürich
Swiss Federal Institute of Technology Zurich

Please respond to question A or B according to your random number.

If your random number is 1, 2, 3, 4, or 5 ⇒ question A:
Have you ever provided misleading or incorrect information on your tax return?

If your random number is 6, 7, 8, or 9 ⇒ question B:
Is your mother's birthday between January and June (including January and June)?
(If you do not know, please use the birth date of someone else you know.)

- Yes
- No

>>

Page 15: Tax evasion (FR Number)

ETH
Eidgenössische Technische Hochschule Zürich
Swiss Federal Institute of Technology Zurich

1. Once again, think of a number between 1 and 12.

1	2	3	4	5	6
7	8	9	10	11	12

2. Got your number?

If yes, click the "Show instruction!" button: Show instruction!

3. Follow the instruction corresponding to the number you picked:

Directly tick Yes ⇒ Tick the "Yes" response button below.
Directly tick No ⇒ Tick the "No" response button below.

Answer question ⇒ Tick the response button corresponding to your answer to this question:
Have you ever provided misleading or incorrect information on your tax return?

○ **Yes**
○ **No**

>>

Page 16: Voting (Direct questioning)

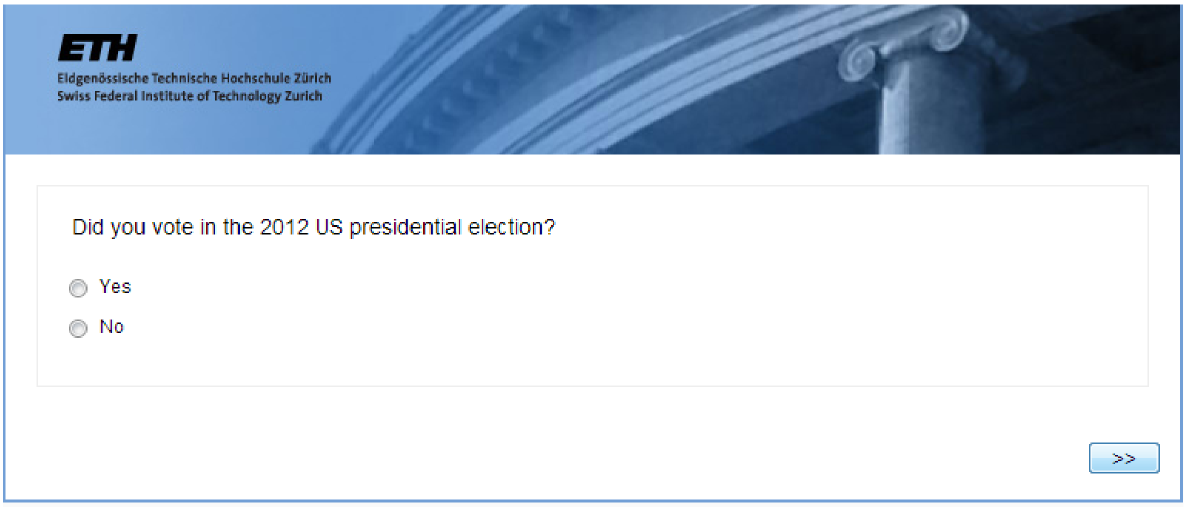

Page 16: Voting (CM Question)

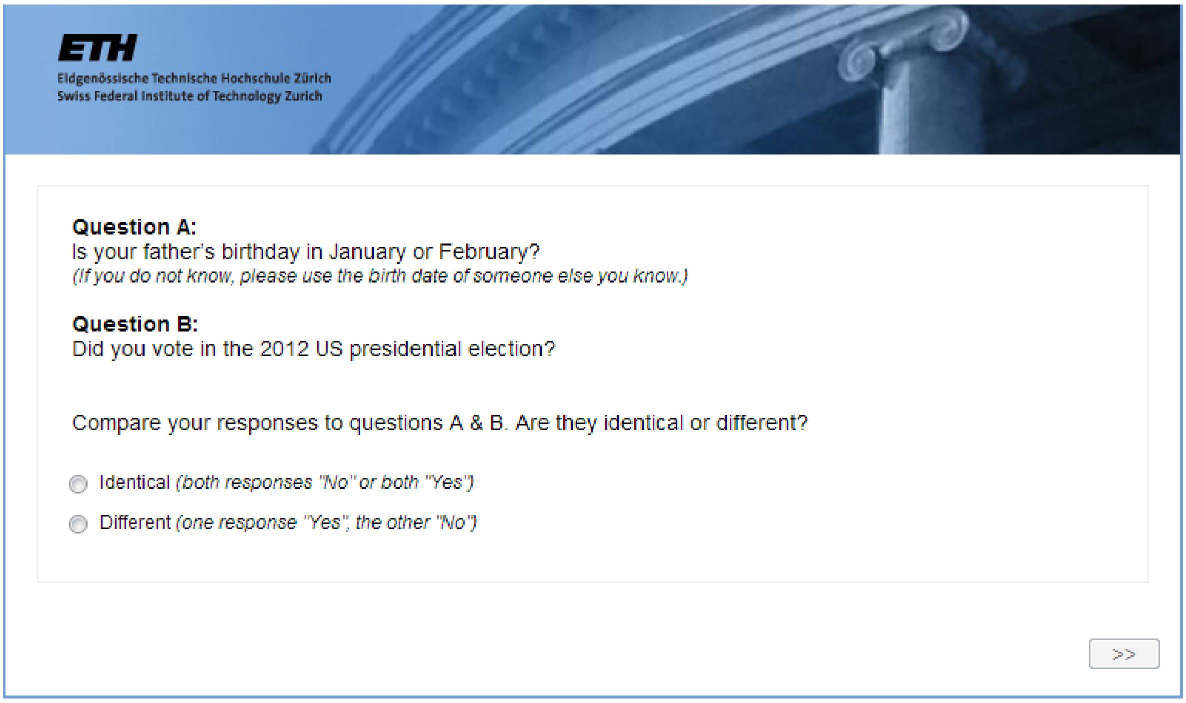

Page 16: Voting (UQ Benford)

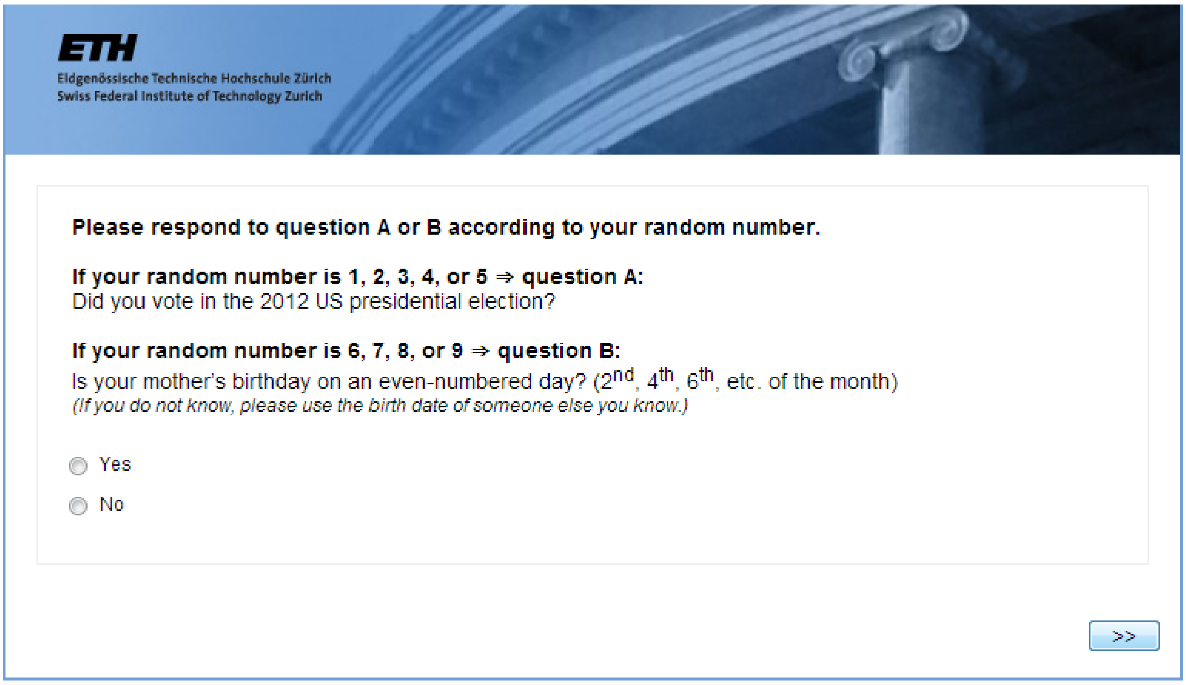
ETH
Eidgenössische Technische Hochschule Zürich
Swiss Federal Institute of Technology Zurich

Please respond to question A or B according to your random number.

If your random number is 1, 2, 3, 4, or 5 ⇒ question A:
Did you vote in the 2012 US presidential election?

If your random number is 6, 7, 8, or 9 ⇒ question B:
Is your mother's birthday on an even-numbered day? (2nd, 4th, 6th, etc. of the month)
(If you do not know, please use the birth date of someone else you know.)

- Yes
- No

>>

Page 16: Voting (FR Number)

ETH
Eidgenössische Technische Hochschule Zürich
Swiss Federal Institute of Technology Zurich

1. Once again, think of a number between 1 and 12.

1	2	3	4	5	6
7	8	9	10	11	12

2. Got your number?

If yes, click the "Show instruction!" button: [Show instruction!]

3. Follow the instruction corresponding to the number you picked:

Directly tick Yes ⇒ Tick the "Yes" response button below.
Directly tick No ⇒ Tick the "No" response button below.

Answer question ⇒ Tick the response button corresponding to your answer to this question:
Did you vote in the 2012 US presidential election?

○ Yes
○ No

[>>]

Page 17: Honest dice game reporting (Direct questioning, prediction game)

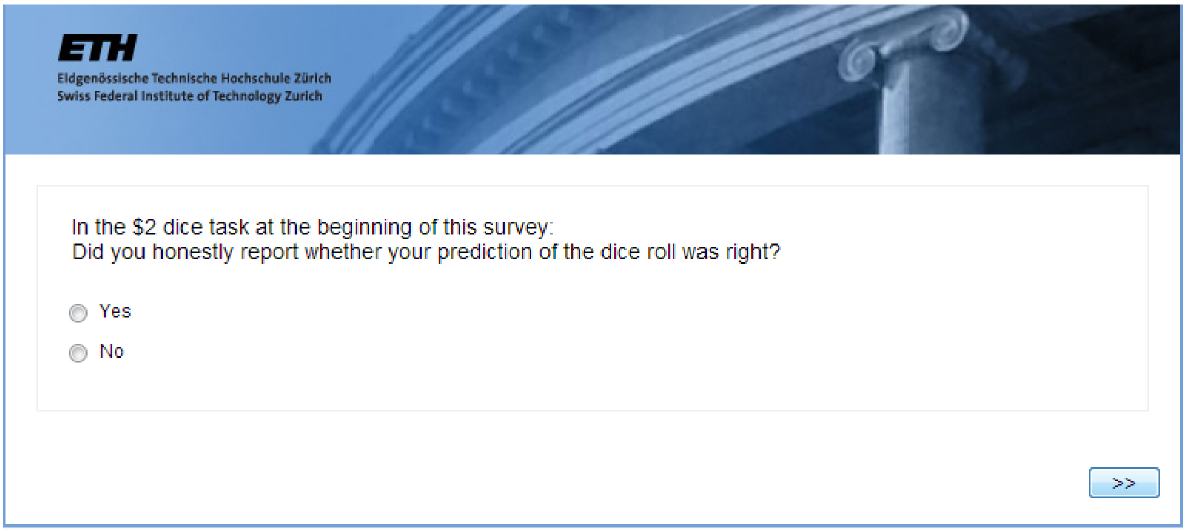
ETH
Eidgenössische Technische Hochschule Zürich
Swiss Federal Institute of Technology Zurich

In the $2 dice task at the beginning of this survey:
Did you honestly report whether your prediction of the dice roll was right?

- Yes
- No

>>

Page 17: Honest dice game reporting (Direct questioning, roll-a-six game)

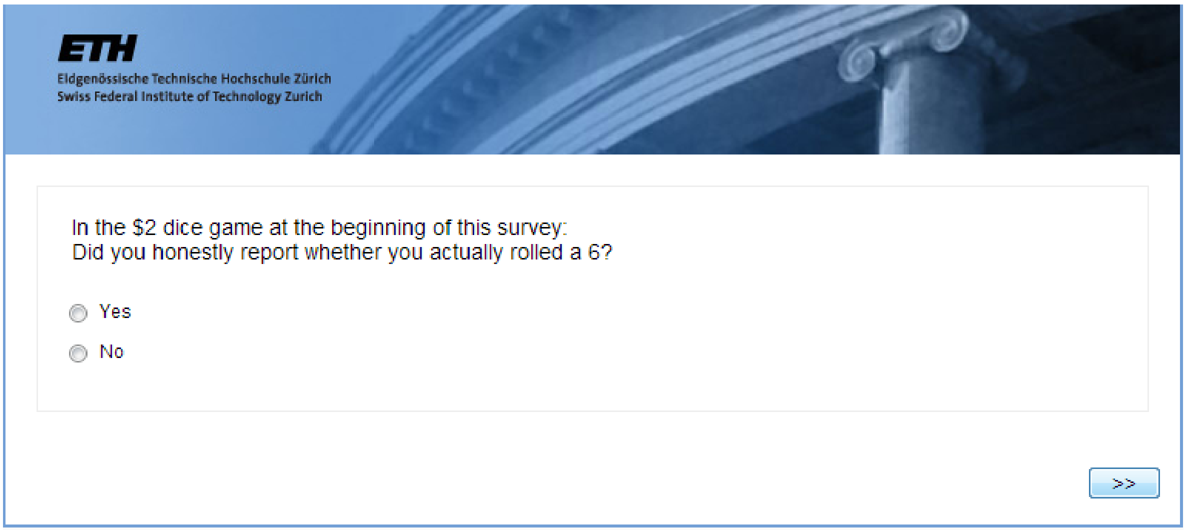
ETH
Eidgenössische Technische Hochschule Zürich
Swiss Federal Institute of Technology Zurich

In the $2 dice game at the beginning of this survey:
Did you honestly report whether you actually rolled a 6?

- Yes
- No

>>

Page 17: Honest dice game reporting (CM Question, prediction game)

ETH
Eidgenössische Technische Hochschule Zürich
Swiss Federal Institute of Technology Zurich

Question A:
Is your father's birthday in January or February?
(If you do not know, please use the birth date of someone else you know.)

Question B:
In the $2 dice task at the beginning of this survey:
Did you honestly report whether your prediction of the dice roll was right?

Compare your responses to questions A & B. Are they identical or different?

- Identical *(both responses "No" or both "Yes")*
- Different *(one response "Yes", the other "No")*

>>

Page 17: Honest dice game reporting (CM Question, roll-a-six game)

ETH
Eidgenössische Technische Hochschule Zürich
Swiss Federal Institute of Technology Zurich

Question A:
Is your mother's birthday between the 1st and the 6th of the month (including the 1st and 6th)?
(If you do not know, please use the birth date of someone else you know.)

Question B:
In the $2 dice game at the beginning of this survey:
Did you honestly report whether you actually rolled a 6?

Compare your responses to questions A & B. Are they identical or different?

- Identical *(both responses "No" or both "Yes")*
- Different *(one response "Yes", the other "No")*

>>

Page 17: Honest dice game reporting (UQ Benford, prediction game)

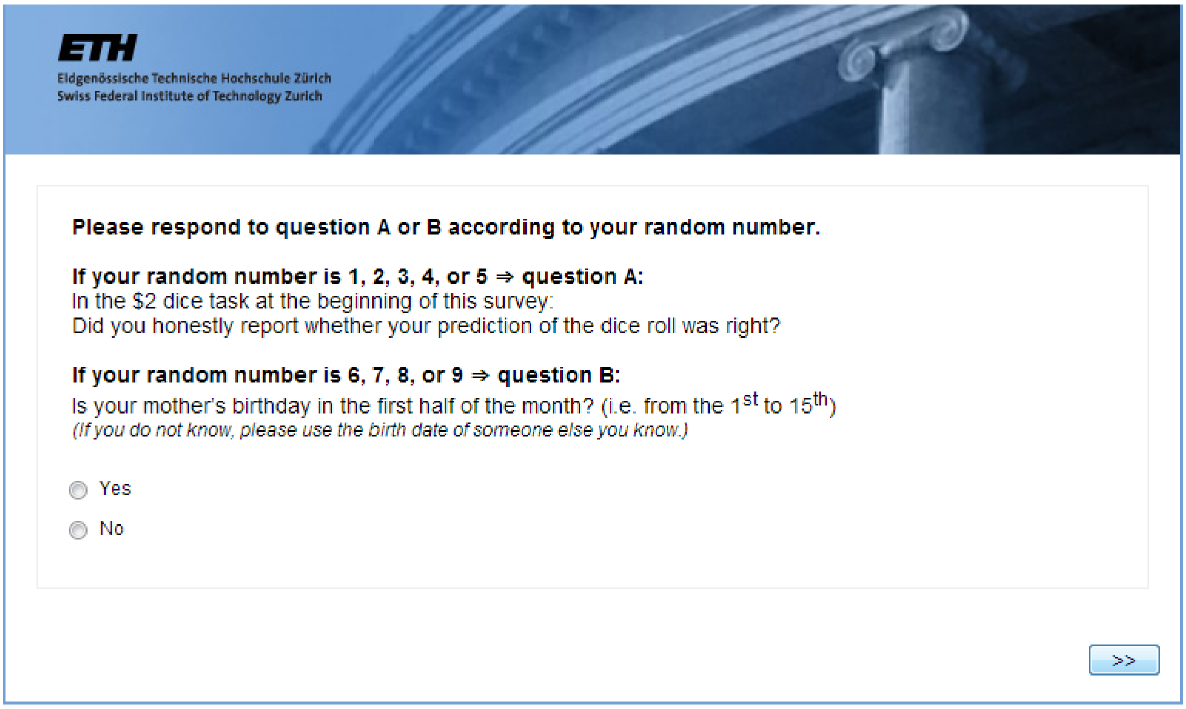

ETH
Eidgenössische Technische Hochschule Zürich
Swiss Federal Institute of Technology Zurich

Please respond to question A or B according to your random number.

If your random number is 1, 2, 3, 4, or 5 ⇒ question A:
In the $2 dice task at the beginning of this survey:
Did you honestly report whether your prediction of the dice roll was right?

If your random number is 6, 7, 8, or 9 ⇒ question B:
Is your mother's birthday in the first half of the month? (i.e. from the 1st to 15th)
(If you do not know, please use the birth date of someone else you know.)

○ Yes
○ No

>>

Page 17: Honest dice game reporting (UQ Benford, roll-a-six game)

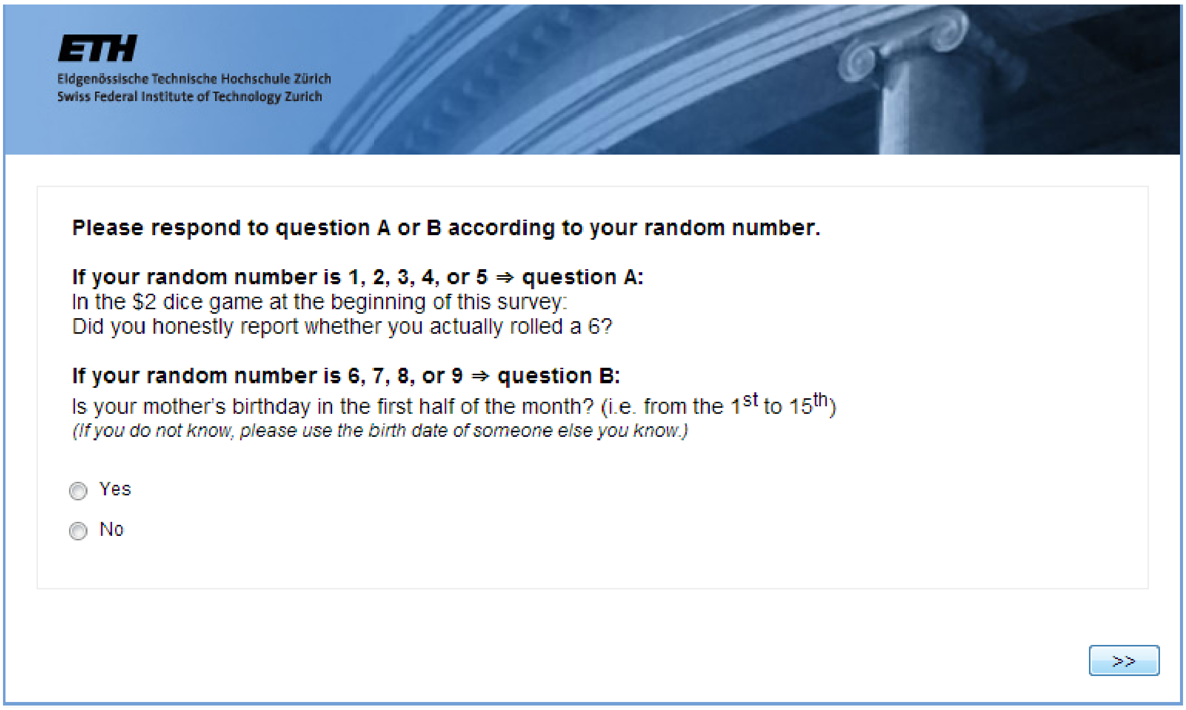

ETH
Eidgenössische Technische Hochschule Zürich
Swiss Federal Institute of Technology Zurich

Please respond to question A or B according to your random number.

If your random number is 1, 2, 3, 4, or 5 ⇒ question A:
In the $2 dice game at the beginning of this survey:
Did you honestly report whether you actually rolled a 6?

If your random number is 6, 7, 8, or 9 ⇒ question B:
Is your mother's birthday in the first half of the month? (i.e. from the 1st to 15th)
(If you do not know, please use the birth date of someone else you know.)

○ Yes
○ No

>>

Page 17: Honest dice game reporting (FR Number, prediction game)

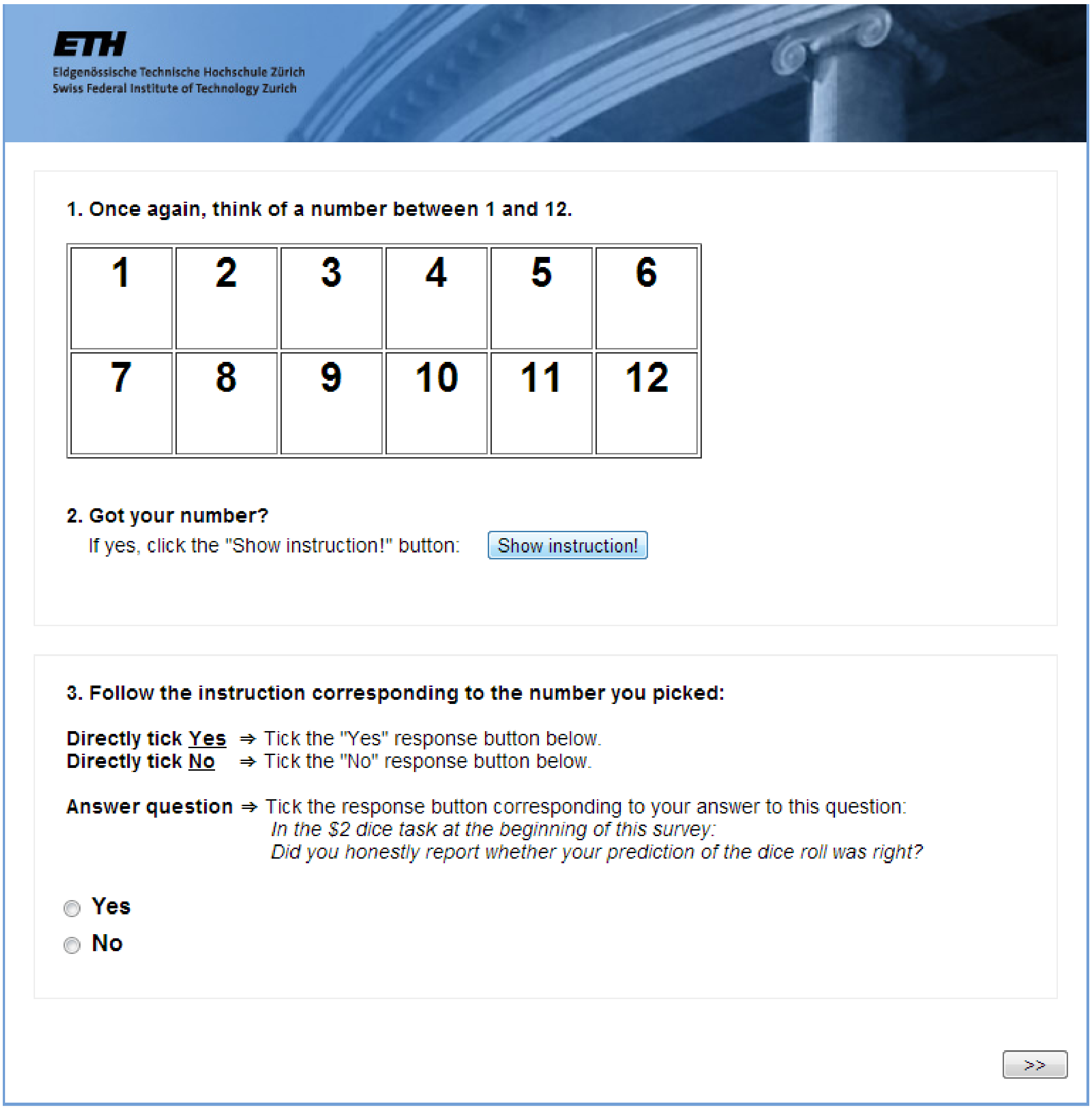

ETH
Eidgenössische Technische Hochschule Zürich
Swiss Federal Institute of Technology Zurich

1. Once again, think of a number between 1 and 12.

1	2	3	4	5	6
7	8	9	10	11	12

2. Got your number?
If yes, click the "Show instruction!" button: Show instruction!

3. Follow the instruction corresponding to the number you picked:

Directly tick Yes ⇒ Tick the "Yes" response button below.
Directly tick No ⇒ Tick the "No" response button below.

Answer question ⇒ Tick the response button corresponding to your answer to this question:
In the $2 dice task at the beginning of this survey:
Did you honestly report whether your prediction of the dice roll was right?

- Yes
- No

>>

Page 17: Honest dice game reporting (FR Number, roll-a-six game)

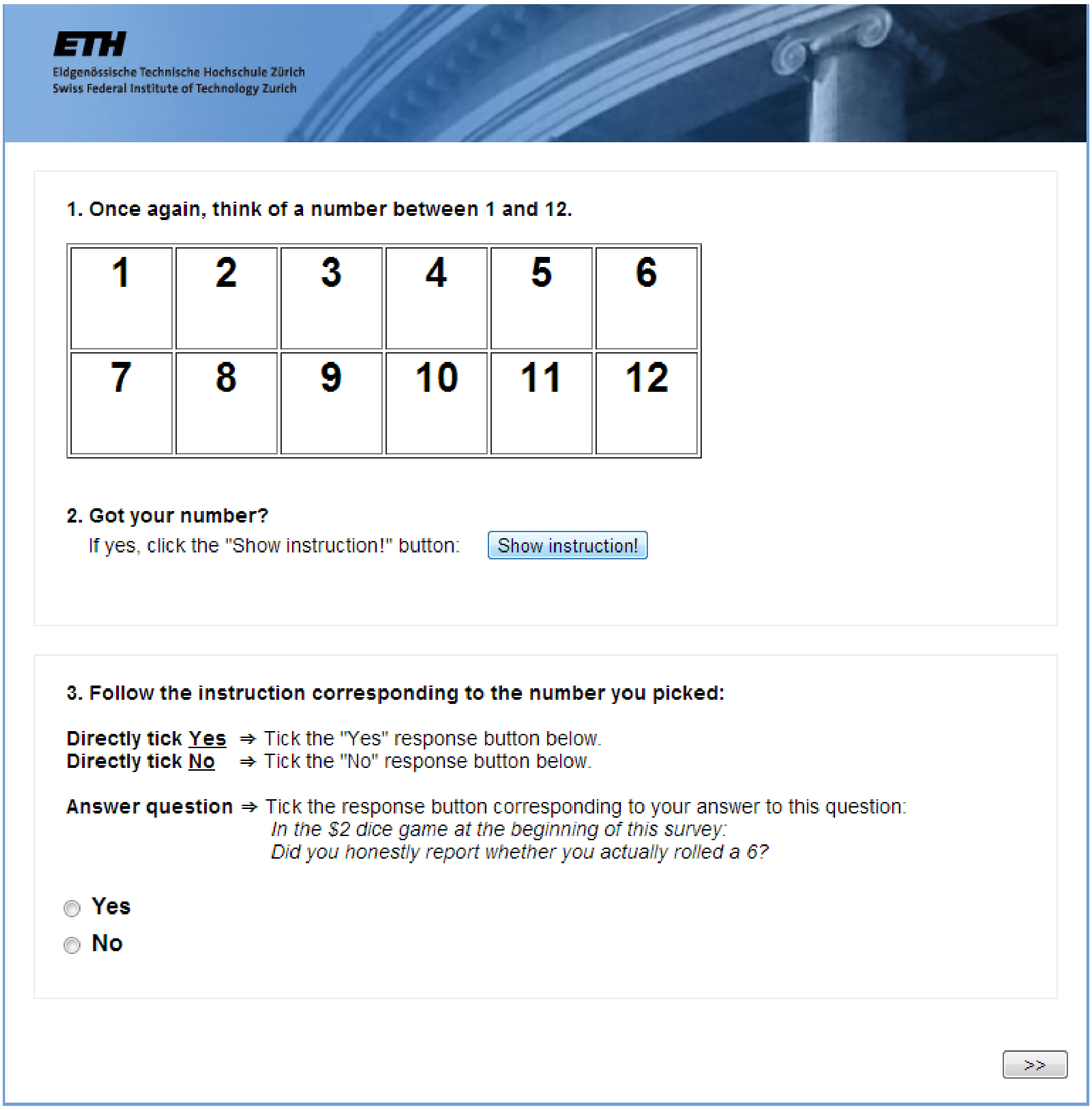

ETH
Eidgenössische Technische Hochschule Zürich
Swiss Federal Institute of Technology Zurich

1. Once again, think of a number between 1 and 12.

1	2	3	4	5	6
7	8	9	10	11	12

2. Got your number?

If yes, click the "Show instruction!" button: Show instruction!

3. Follow the instruction corresponding to the number you picked:

Directly tick Yes ⇒ Tick the "Yes" response button below.
Directly tick No ⇒ Tick the "No" response button below.

Answer question ⇒ Tick the response button corresponding to your answer to this question:
In the $2 dice game at the beginning of this survey:
Did you honestly report whether you actually rolled a 6?

○ Yes
○ No

>>

Page 18: Trust in survey confidentiality (prediction game)

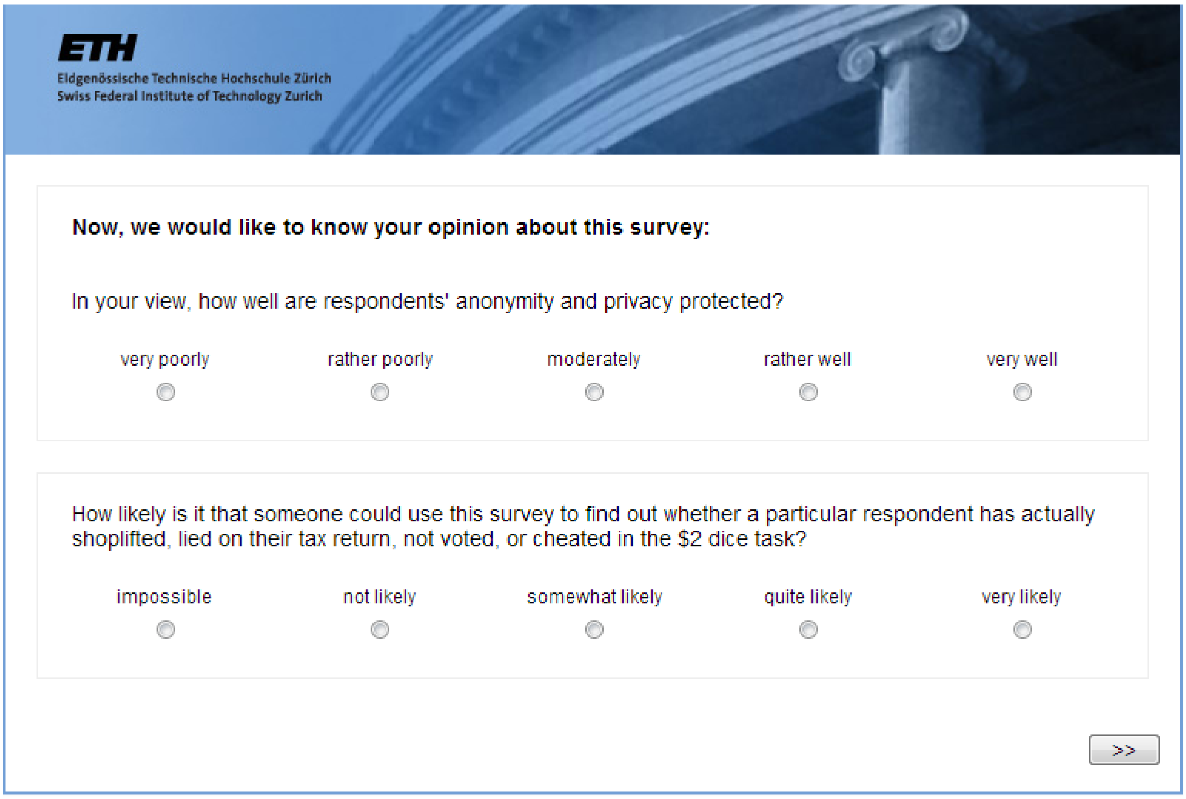
Eldgenössische Technische Hochschule Zürich
Swiss Federal Institute of Technology Zurich

Now, we would like to know your opinion about this survey:

In your view, how well are respondents' anonymity and privacy protected?

very poorly ○ rather poorly ○ moderately ○ rather well ○ very well ○

How likely is it that someone could use this survey to find out whether a particular respondent has actually shoplifted, lied on their tax return, not voted, or cheated in the $2 dice task?

impossible ○ not likely ○ somewhat likely ○ quite likely ○ very likely ○

>>

Page 18: Trust in survey confidentiality (roll-a-six game)

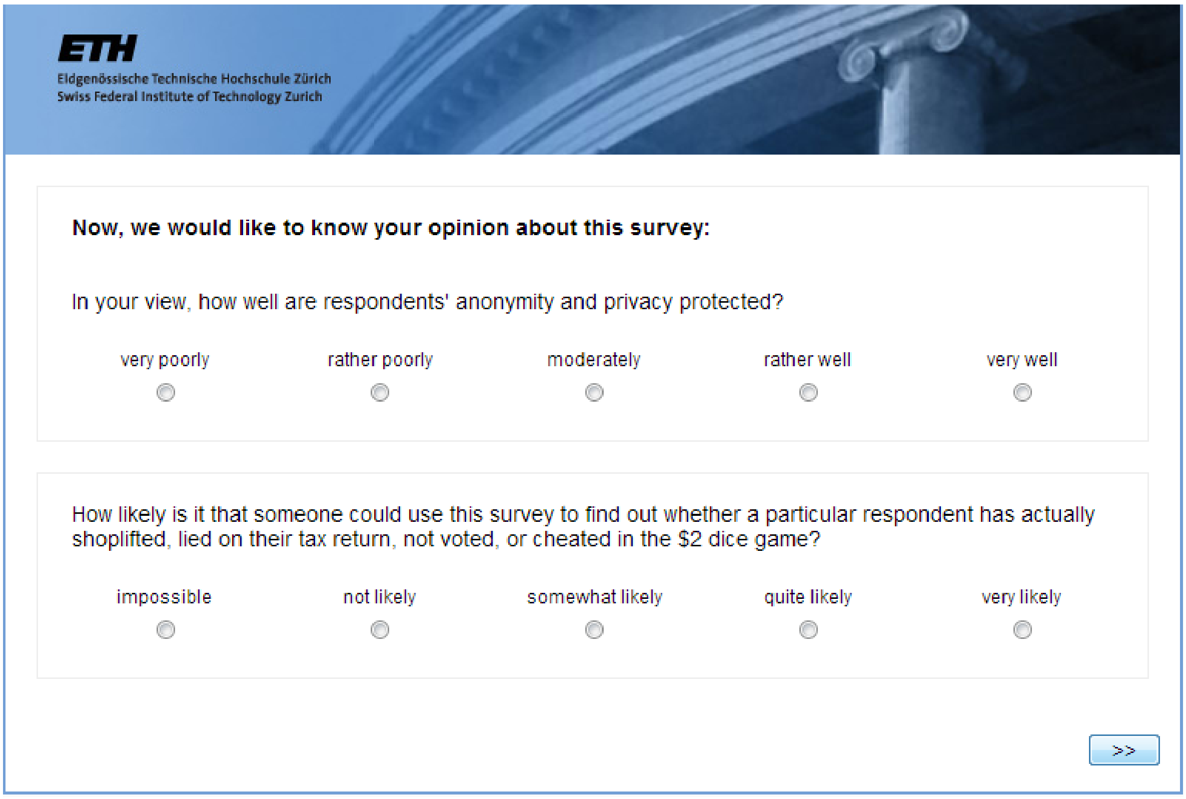
Eldgenössische Technische Hochschule Zürich
Swiss Federal Institute of Technology Zurich

Now, we would like to know your opinion about this survey:

In your view, how well are respondents' anonymity and privacy protected?

very poorly ○ rather poorly ○ moderately ○ rather well ○ very well ○

How likely is it that someone could use this survey to find out whether a particular respondent has actually shoplifted, lied on their tax return, not voted, or cheated in the $2 dice game?

impossible ○ not likely ○ somewhat likely ○ quite likely ○ very likely ○

>>

Page 19: Evaluation of special technique (RRT)

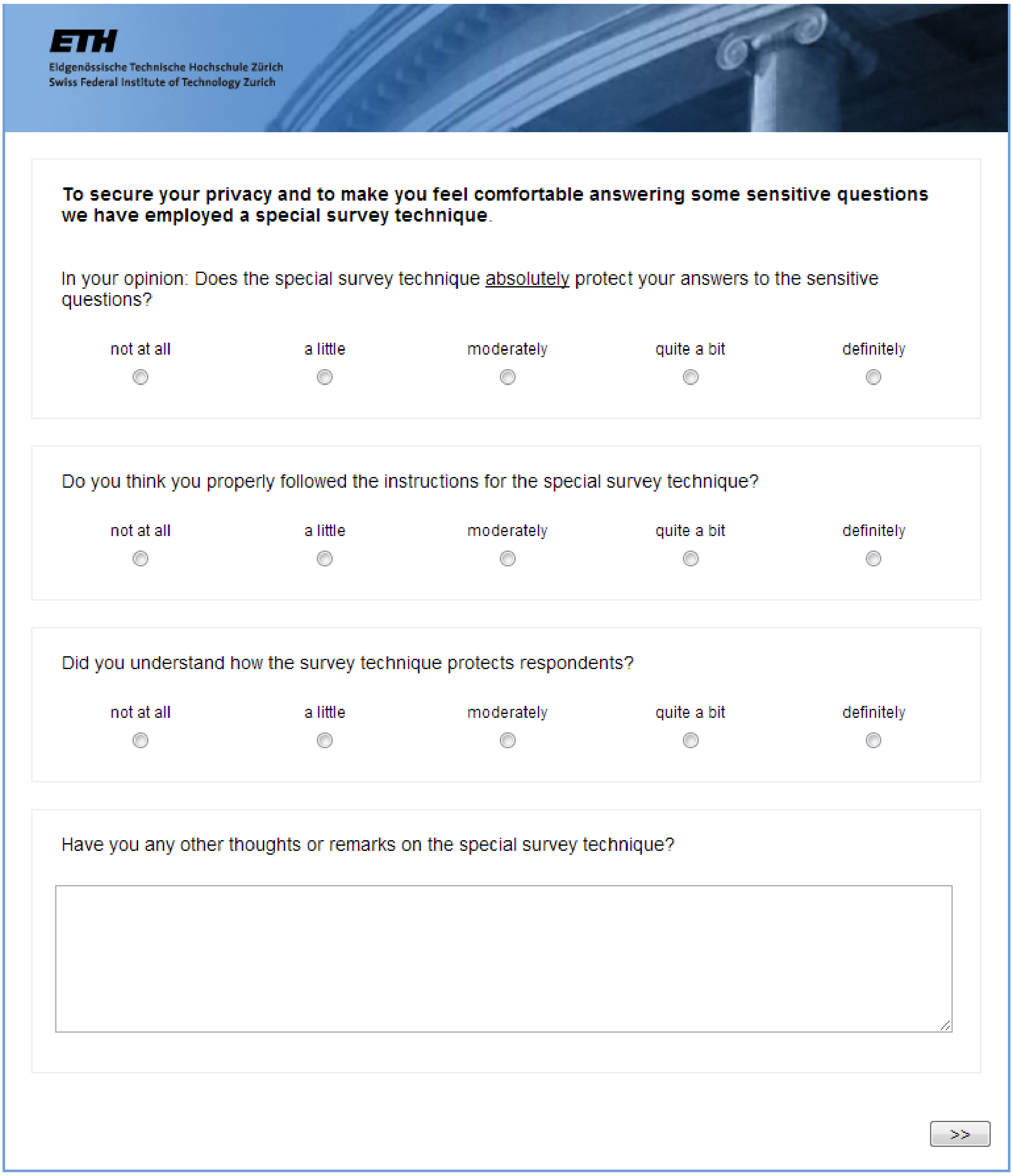

ETH
Eidgenössische Technische Hochschule Zürich
Swiss Federal Institute of Technology Zurich

To secure your privacy and to make you feel comfortable answering some sensitive questions we have employed a special survey technique.

In your opinion: Does the special survey technique absolutely protect your answers to the sensitive questions?

not at all	a little	moderately	quite a bit	definitely
○	○	○	○	○

Do you think you properly followed the instructions for the special survey technique?

not at all	a little	moderately	quite a bit	definitely
○	○	○	○	○

Did you understand how the survey technique protects respondents?

not at all	a little	moderately	quite a bit	definitely
○	○	○	○	○

Have you any other thoughts or remarks on the special survey technique?

>>

Page 20: Randomizing device test (Benford procedure)

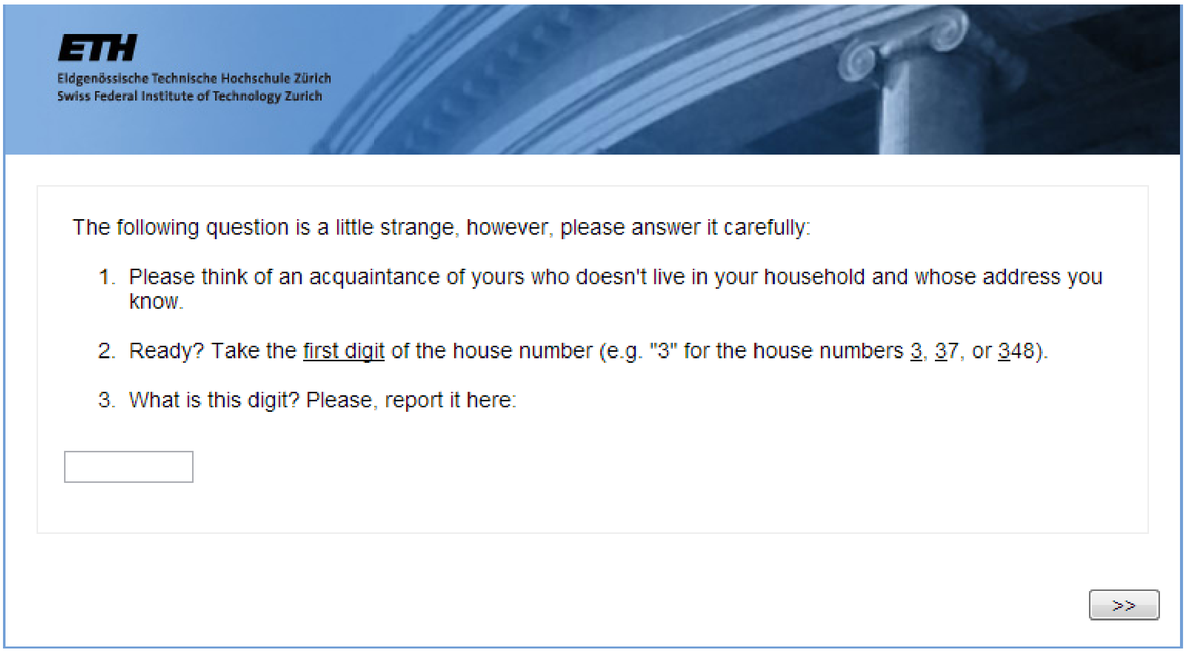

ETH
Eidgenössische Technische Hochschule Zürich
Swiss Federal Institute of Technology Zurich

The following question is a little strange, however, please answer it carefully:

1. Please think of an acquaintance of yours who doesn't live in your household and whose address you know.
2. Ready? Take the first digit of the house number (e.g. "3" for the house numbers 3, 37, or 348).
3. What is this digit? Please, report it here:

>>

Page 20: Randomizing device test (Unrelated questions for CM Question)

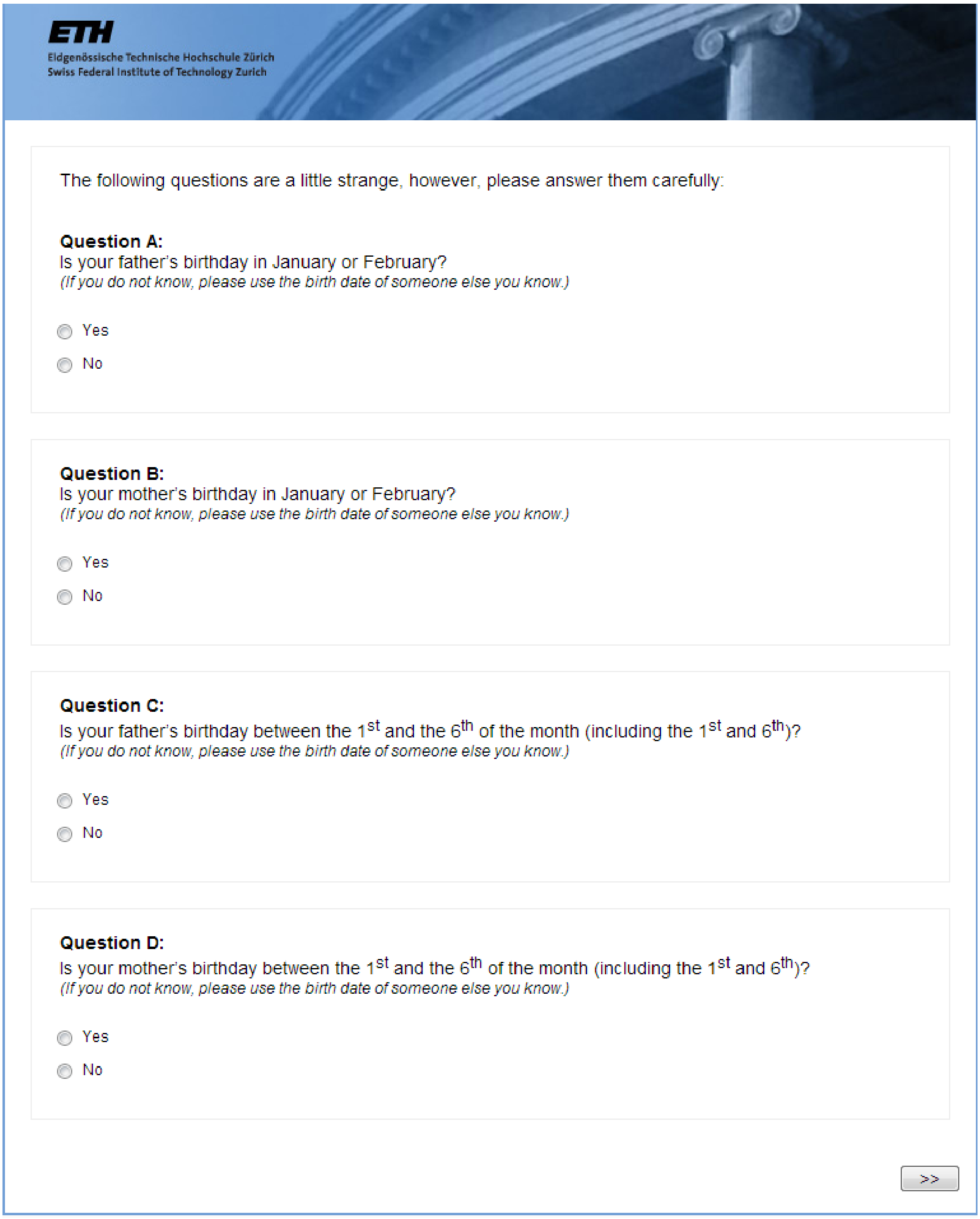

The following questions are a little strange, however, please answer them carefully:

Question A:
Is your father's birthday in January or February?
(If you do not know, please use the birth date of someone else you know.)

- Yes
- No

Question B:
Is your mother's birthday in January or February?
(If you do not know, please use the birth date of someone else you know.)

- Yes
- No

Question C:
Is your father's birthday between the 1st and the 6th of the month (including the 1st and 6th)?
(If you do not know, please use the birth date of someone else you know.)

- Yes
- No

Question D:
Is your mother's birthday between the 1st and the 6th of the month (including the 1st and 6th)?
(If you do not know, please use the birth date of someone else you know.)

- Yes
- No

>>

Page 20: Randomizing device test (Unrelated questions for UQ Benford)

ETH
Eidgenössische Technische Hochschule Zürich
Swiss Federal Institute of Technology Zurich

The following questions are a little strange, however, please answer them carefully:

Question A:
Is your mother's birthday on an even-numbered day? (2nd, 4th, 6th, etc. of the month)
(If you do not know, please use the birth date of someone else you know.)

- ○ Yes
- ○ No

Question B:
Is your mother's birthday in the first half of the month? (i.e. from the 1st to 15th)
(If you do not know, please use the birth date of someone else you know.)

- ○ Yes
- ○ No

Question C:
Is your mother's birthday between January and June (including January and June)?
(If you do not know, please use the birth date of someone else you know.)

- ○ Yes
- ○ No

Question D:
Is your mother's birthday in an even-numbered month? (i.e. Feb., Apr., Jun., Aug., Oct., or Dec.)
(If you do not know, please use the birth date of someone else you know.)

- ○ Yes
- ○ No

[>>]

Page 20: Randomizing device test (Pick-a-number device (standard))

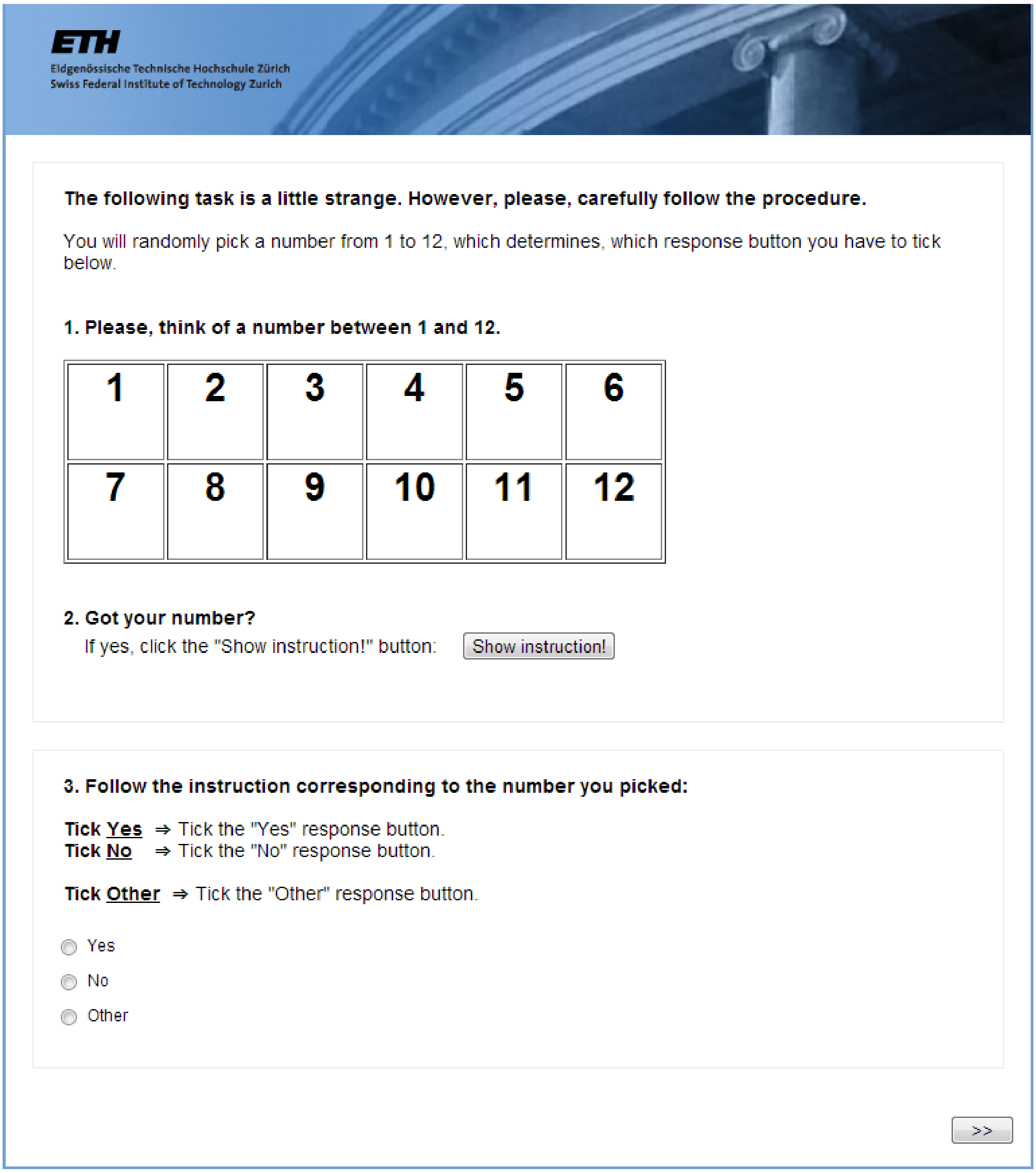

The following task is a little strange. However, please, carefully follow the procedure.

You will randomly pick a number from 1 to 12, which determines, which response button you have to tick below.

1. Please, think of a number between 1 and 12.

1	2	3	4	5	6
7	8	9	10	11	12

2. Got your number?

If yes, click the "Show instruction!" button: Show instruction!

3. Follow the instruction corresponding to the number you picked:

Tick Yes ⇒ Tick the "Yes" response button.
Tick No ⇒ Tick the "No" response button.

Tick Other ⇒ Tick the "Other" response button.

- Yes
- No
- Other

>>

Page 20: Randomizing device test (Pick-a-number device (generic))

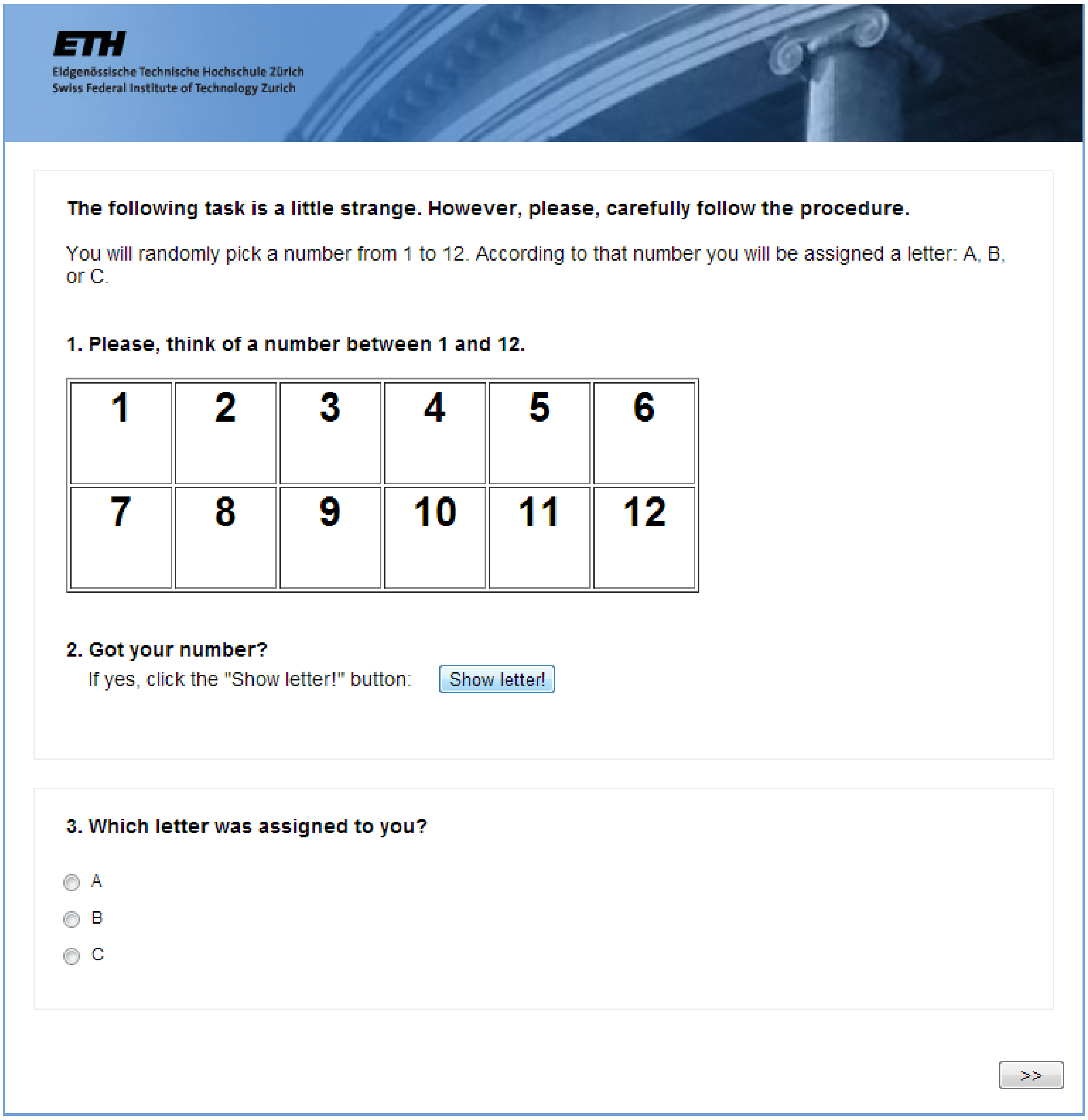

ETH
Eidgenössische Technische Hochschule Zürich
Swiss Federal Institute of Technology Zurich

The following task is a little strange. However, please, carefully follow the procedure.

You will randomly pick a number from 1 to 12. According to that number you will be assigned a letter: A, B, or C.

1. Please, think of a number between 1 and 12.

1	2	3	4	5	6
7	8	9	10	11	12

2. Got your number?

If yes, click the "Show letter!" button: Show letter!

3. Which letter was assigned to you?

- A
- B
- C

>>

Page 21: Birth date knowledge

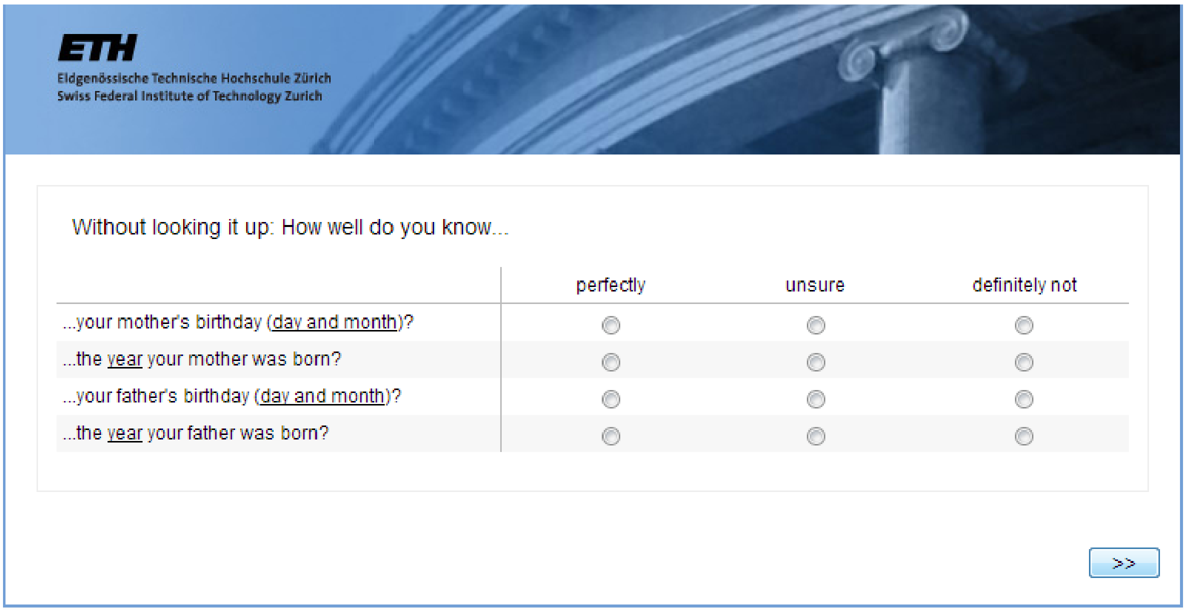

ETH
Eidgenössische Technische Hochschule Zürich
Swiss Federal Institute of Technology Zurich

Without looking it up: How well do you know...

	perfectly	unsure	definitely not
...your mother's birthday (day and month)?	○	○	○
...the year your mother was born?	○	○	○
...your father's birthday (day and month)?	○	○	○
...the year your father was born?	○	○	○

>>

Page 22: Respondents' comments

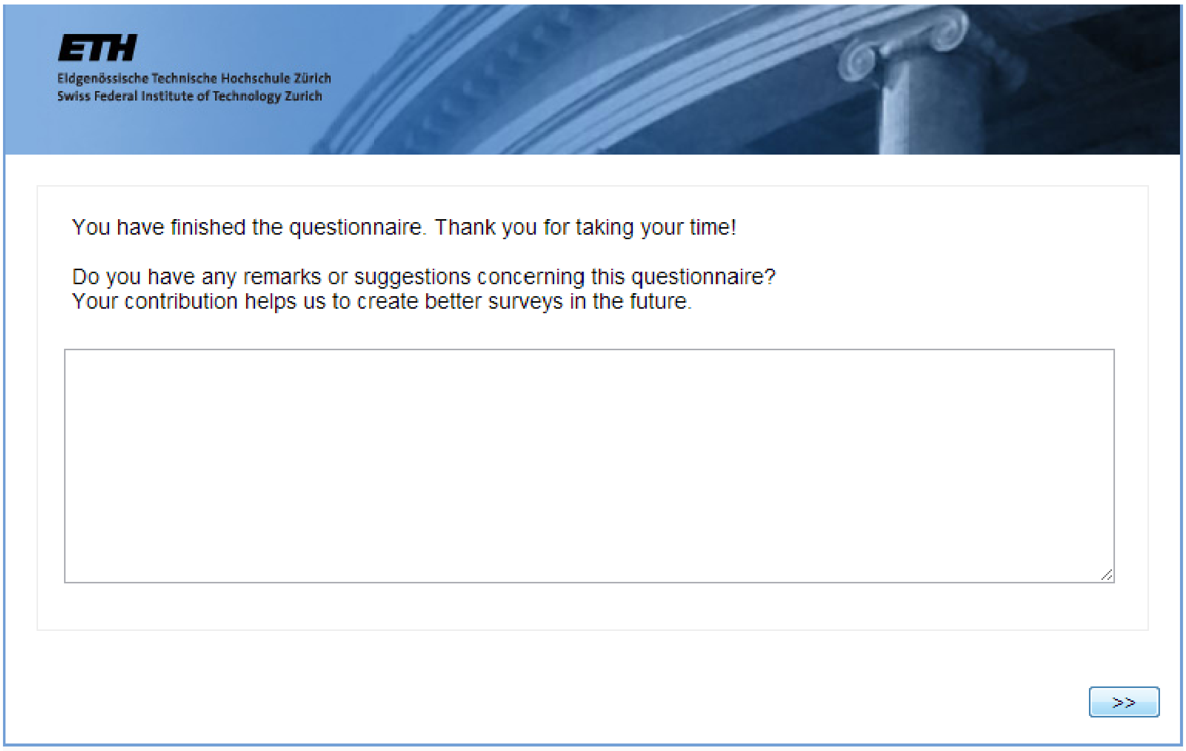

ETH
Eidgenössische Technische Hochschule Zürich
Swiss Federal Institute of Technology Zurich

You have finished the questionnaire. Thank you for taking your time!

Do you have any remarks or suggestions concerning this questionnaire?
Your contribution helps us to create better surveys in the future.

>>

Page 23: Final page

ETH
Eidgenössische Technische Hochschule Zürich
Swiss Federal Institute of Technology Zurich

In order to receive your payment you must copy and paste the following code back to AMT:

jyOjVICNKmKa

Your payment will be processed within the next 24 hours.
If you encounter problems submitting this HIT, please, search for a HIT called "ETH Descil Trouble TICKET Mood and Personality" and report your problem there.

The survey you just finished is part of a scientific study conducted at the Swiss Federal Institute of Technology Zurich. Results from the study will be used to develop new survey methods to ask sensitive issues in areas such as epidemiology, criminology or political science.

You were asked some sensitive questions on cheating behavior. All answers are kept confidential and participants' privacy is completely secured.

All participants who completed the survey will receive the indicated payments irrespective of their answers in the survey.

If you have any remarks on or questions about this study, please, do not hesitate to contact me.

Marc Hoeglinger

Chair of Sociology
Swiss Federal Institute of Technology
Clausiusstrasse 50
8092 Zurich
Switzerland
marchoe@ethz.ch
phone: +41 44 632 55 58

A.3 JavaScript code

All JavaScript code authored by Philip Tschiemer. For additional code documentation see https://github.com/tschiemer/qualtrics-gambling.

A.3.1 Dice

For the prediction game the following code was used:

```
<strong>Have you memorized your prediction?</strong> (1, 2, 3, 4, 5, or 6)<br />
<br />
If yes, roll the dice below by clicking on the button. Please note <u>your first roll counts</u>!
<br />
<br /><br /><br />
<script src="https://www.descil.ethz.ch/projects/1305-SensQuest/dice/dice.js"></script>
<div style="position:absolute; left:200px;">
<div id="dice"></div></div>
 <br /> <br /> <br />
<div style="position:absolute; left:179px;">

  <button id="throw">Roll dice</button>
</div>
 <script type="text/javascript">
// Dice parameters
var current_dice = 'dice1';
var max_throws = 20;
var throw_nr_offset = 0;

// remaining throws for this dice
var done_throws = ${e://Field/done_throws}+0;
var counting_for_dice = '${e://Field/current_dice}';
if (counting_for_dice != current_dice)
{
    Qualtrics.SurveyEngine.setEmbeddedData('current_dice', current_dice);
    Qualtrics.SurveyEngine.setEmbeddedData('done_throws', 0);
    done_throws = 0;
}
throw_nr_offset += done_throws;

// instantiate dice
var dice = new Dice("dice","throw",{
    possible_throws: max_throws - done_throws,
    value_set:[
      {key:"1",probability:1},
      {key:"2",probability:1},
      {key:"3",probability:1},
      {key:"4",probability:1},
      {key:"5",probability:1},
      {key:"6",probability:1}
    ],
result_set:
'${e://Field/rnd_dr1},${e://Field/rnd_dr2},${e://Field/rnd_dr3},${e://Field/rnd_dr4},${e://Field/rnd_dr5},${e://Field/rnd_dr6},${e://Fi
eld/rnd_dr7},${e://Field/rnd_dr8},${e://Field/rnd_dr9},${e://Field/rnd_dr10},${e://Field/rnd_dr11},${e://Field/rnd_dr12},${e://Field/rn
d_dr13},${e://Field/rnd_dr14},${e://Field/rnd_dr15},${e://Field/rnd_dr16},${e://Field/rnd_dr17},${e://Field/rnd_dr18},${e://Field/rnd_d
r19},${e://Field/rnd_dr20}',
 first_throw_nr: ${e://Field/result_throw_nr}+0,
 images: {base_url:'https://www.descil.ethz.ch/projects/1305-SensQuest/dice/img/',ext:'.png', init : 'qmark1.png'},
    animation: {images: []},
result_callback: function(throw_nr,result_key){
Qualtrics.SurveyEngine.setEmbeddedData('drr'+(throw_nr+throw_nr_offset), result_key);
Qualtrics.SurveyEngine.setEmbeddedData('result_throw_nr', throw_nr + this.first_throw_nr);
Qualtrics.SurveyEngine.setEmbeddedData('done_throws', throw_nr+throw_nr_offset);
}
});
// if no throws remain deactivate controls
if (max_throws - done_throws <= 0)
{
    dice.deactivate_controls();
}
</script></div>
```

For the roll-a-six game the following code was used:

```
<strong>Are you ready to roll the dice?</strong><br />
<br />
If yes, roll the dice below by clicking on the button. Please note <u>your first roll counts</u>!
<br />
<br /><br /><br />

<script src="https://www.descil.ethz.ch/projects/1305-SensQuest/dice/dice.js"></script>

<div style="position:absolute; left:200px;">
<div id="dice"></div></div>
 <br /> <br /> <br />
<div style="position:absolute; left:179px;">

  <button id="throw">Roll dice</button>
</div>
 <script type="text/javascript">
// Dice parameters
var current_dice = 'dice1';
var max_throws = 20;
var throw_nr_offset = 0;

// remaining throws for this dice
var done_throws = ${e://Field/done_throws}+0;
var counting_for_dice = '${e://Field/current_dice}';
if (counting_for_dice != current_dice)
{
    Qualtrics.SurveyEngine.setEmbeddedData('current_dice', current_dice);
    Qualtrics.SurveyEngine.setEmbeddedData('done_throws', 0);
    done_throws = 0;
}
throw_nr_offset += done_throws;

// instantiate dice
var dice = new Dice("dice","throw",{
    possible_throws: max_throws - done_throws,
    value_set:[
      {key:"1",probability:1},
      {key:"2",probability:1},
      {key:"3",probability:1},
      {key:"4",probability:1},
      {key:"5",probability:1},
      {key:"6",probability:1}
    ],
result_set:
'${e://Field/rnd_dr1},${e://Field/rnd_dr2},${e://Field/rnd_dr3},${e://Field/rnd_dr4},${e://Field/rnd_dr5},${e://Field/rnd_dr6},${e://Fi
eld/rnd_dr7},${e://Field/rnd_dr8},${e://Field/rnd_dr9},${e://Field/rnd_dr10},${e://Field/rnd_dr11},${e://Field/rnd_dr12},${e://Field/rn
d_dr13},${e://Field/rnd_dr14},${e://Field/rnd_dr15},${e://Field/rnd_dr16},${e://Field/rnd_dr17},${e://Field/rnd_dr18},${e://Field/rnd_d
r19},${e://Field/rnd_dr20}',
 first_throw_nr: ${e://Field/result_throw_nr}+0,
 images: {base_url:'https://www.descil.ethz.ch/projects/1305-SensQuest/dice/img/',ext:'.png', init : 'qmark1.png'},
    animation: {images: []},
result_callback: function(throw_nr,result_key){
Qualtrics.SurveyEngine.setEmbeddedData('drr'+(throw_nr+throw_nr_offset), result_key);
Qualtrics.SurveyEngine.setEmbeddedData('result_throw_nr', throw_nr + this.first_throw_nr);
Qualtrics.SurveyEngine.setEmbeddedData('done_throws', throw_nr+throw_nr_offset);
}
});
// if no throws remain deactivate controls
if (max_throws - done_throws <= 0)
{
    dice.deactivate_controls();
}
</script></div>
```

In addition, the library dice.js was called:

```
/**
 * dice.js
 *
 * For demo see dice.html
 *
 * @copyright 2013 ETH Zurich, www.socio.ethz.ch, c/o Marc Hoeglinger <hoeglinger@soz.gess.ethz.ch>
 * @author Philip Tschiemer <tschiemer@filou.se>
 * @version 2013-09-05
 */


    function Dice(dice_id, btn_throw_id, options)
    {
        if (dice_id == undefined || btn_throw_id == undefined || options == undefined)
        {
            alert('Parameters not set. Please make sure to set <dice_id>, <btn_throw_id> and <options>.');
            return;
        }

        // Dice initialization

        // Required for correct referencing in anonymous functions
        var self = this;

        // Is Dice currently being thrown?
        this.is_throwing = false;

        // Number of throws that can be made before controls are deactivated
        this.possible_throws = options.possible_throws == undefined ? 0 : options.possible_throws;

        // Number of throws currently done.
        this.done_throws     = 0;

        // Reference to dice DOM container
        this.dice       = document.getElementById(dice_id);

        // Reference to throw button DOM
        this.btn_throw  = document.getElementById(btn_throw_id);

        // Set of all possible values following the structure
        // {key: "my_key", probability: 1} where the key field will be passed to any callback
        this.value_set = options.value_set;

        // Set of all predefined result keys, either an array of key values or
        // a string with comma-separated keys, ie
        // ['key1','key2',..,'keyN']   OR   'key1,key2,..,keyN'
        this.result_set = [];
        if (typeof options.result_set != 'undefined')
        {
            this.first_throw_nr = 0;
            if (typeof options.first_throw_nr == 'string')
            {
                this.first_throw_nr = parseInt(options.first_throw_nr);
            }
            else if (typeof options.first_throw_nr == 'number')
            {
                this.first_throw_nr = Math.floor(options.first_throw_nr);
            }

            if (typeof options.result_set == 'string')
            {
               this.result_set = options.result_set.replace(/ /g,'').split(',');
            }
            else
            {
                this.result_set = options.result_set;
            }

            if (this.possible_throws == 0)
            {
                this.possible_throws = this.result_set.length - this.first_throw_nr;
//                if (this.possible_throws +  > this.result_set.length)
//                {
//                    this.possible_throws = this.result_set.length - this.first_throw_nr;
//                }
            }

            this.fallback_strategy = 'wrap-around';
            if (typeof options.fallback_strategy == 'string')
            {
                this.fallback_strategy = options.fallback_strategy;
            }
        }

        // Duration of roll/throw animation, resp duration until result is shown.
        // Can be number or function returning a number
        // Default 1
        // In Seconds.
        this.roll_duration = options.roll_duration == undefined ? 1 : options.roll_duration;

        // Callback function to receive any results
        // Signature: result_callback( throw_nr, result_key )
        //  throw_nr    : number of current throw starting with 1
        //  result_key  : key of throw result, as given through value_set
        this.result_callback = null;
        if (options.result_callback !== undefined)
        {
```

```
            this.result_callback = options.result_callback;
        }

        if (this.possible_throws > 0 && options.finished_callback !== undefined)
        {
            this.finished_callback = options.finished_callback;
        }

        // default Animation options, can be overriden
        this.animation = {
            rotate: true,           // Rotate image?
            swap_images: true,      // Randomly swap images during animation?
            nsteps: 20,             // Number of steps a rotation is divided into.
            change_image_all: 5     // Swap images each n-th step where n = this setting
        };

        // override animation settings with given options
        if (options.animation !== undefined)
        {
            for(var key in options.animation)
            {
                this.animation[key] = options.animation[key];
            }
        }

        // Store image options
        if (options.images == undefined)
        {
            alert('Image settings missing.');
            return;
        }
        this.images = options.images;


        // Sanity checks: could DOM elements be found?
        if (this.dice == null){
            alert('Dice ID not properly set, could not find an element with id '+dice_id);
            return;
        }
        if (this.btn_throw == null){
            alert('Throw button ID not properly set, could not find an element.'+btn_throw_id);
            return;
        }

//        if (options.btn_reset_id !== undefined)
//        {
//            this.btn_reset      = document.getElementById(options.btn_reset_id);
//            if (this.btn_reset == null){
//                alert('Reset button id not properly set, could not find an element with id '+options.btn_reset_id);
//                return;
//            }
//            this.reset = function()
//            {
//                if (this.callback != null)
//                {
//                    while(this.done_throws > 0)
//                    {
//                        this.callback('reset_throw',this.done_throws);
//                        this.done_throws--;
//                    }
//                }
//            }
//            this.btn_reset.onclick = function(){self.reset();};
//        }


        //// Finalize Value Sets (sanity check and normalize probablities)
        var sum = 0, count=0;
        for(var v = 0; v < this.value_set.length; v++)
        {
            sum += this.value_set[v].probability;
            count++;
        }
        if (count == 0)
        {
            alert('No possible results entered.');
            return;
        }
        // if probablity sum = 0, assume uniform probablitiy distribution
        if (sum == 0)
        {
            var p = 1 / count;
            for (var v = 0; v < this.value_set.length; v++)
            {
                this.value_set[v].probability = p;
            }
        }
        else
        {
            for(var v = 0; v < this.value_set.length; v++)
            {
                this.value_set[v].probability /= sum;
            }
        }


        //// Setup HTML / Style
```

```
        //
        this.dice.innerHTML = '';

        // create main image
        this.dice_img = document.createElement('img');
        if (typeof this.images.init != 'undefined')
        {
            this.dice_img.src = this.images.base_url + this.images.init;
        }
        else
        {
            this.dice_img.src = this.images.base_url + this.value_set[0].key + this.images.ext;
        }
        //this.dice_img.style.cursor = 'pointer';
        this.dice.appendChild(this.dice_img);

        // preload result images
        for(var v = 0; v < this.value_set.length; v++)
        {
            var img = document.createElement('img');
            img.style.display = 'none';
            img.src = this.images.base_url + this.value_set[v].key + this.images.ext;

            this.dice.appendChild(img);
        }

        // preload animation images
        if (this.animation.images !== undefined)
        {
            for(var i = 0; i < this.animation.images.length; i++ )
            {
                var img = document.createElement('img');
                img.style.display = 'none';
                img.src = this.images.base_url + this.animation.images[i];

                this.dice.appendChild(img);
            }
        }


        //// Object Methods


        this.deactivate_controls = function()
        {
            this.btn_throw.disabled = true;
//            if (this.btn_reset !== undefined)
//            {
//                this.btn_reset.disabled = true;
//            }
        }

        this.activate_controls = function()
        {
            this.btn_throw.disabled = false;
//            if (this.btn_reset !== undefined)
//            {
//                this.btn_reset.disabled = false;
//            }
        }


        // Throw dice
        this.throw_dice = function()
        {
            if (this.possible_throws > 0 && this.done_throws >= this.possible_throws)
            {
                return;
            }
            if (this.is_throwing)
            {
                return;
            }
            this.is_throwing = true;
            this.deactivate_controls();

            // get roll duration
            var roll_duration;
            if (typeof this.roll_duration == 'number')
            {
                roll_duration = this.roll_duration;
            }
            else
            {
                roll_duration = this.roll_duration();
            }
            roll_duration = Math.floor(1000*roll_duration); // is in millisec

            // Start animation and tell it how long it will likely last (required for computation of
            this.start_animation(roll_duration);


            // Determine result ..
            var result = null;

            // .. by relying on the given result
```

```
            if (this.result_set.length > 0 && (this.first_throw_nr + this.done_throws < this.result_set.length || this.
            fallback_strategy == 'wrap-around'))
            {
                var idx = (this.first_throw_nr + this.done_throws) % this.result_set.length;
                var result_key = this.result_set[idx];
                for(var i=0; i < this.value_set.length; i++)
                {
                    if (this.value_set[i].key == result_key)
                    {
                        result = this.value_set[i];
                        break;
                    }
                }
            }
            // .. or by randomly drawing one.
            else
            {

                // Determine result (Monte Carlo style, 1 sample..)
                var p = Math.random();
                var sum = 0;
                for(var v in this.value_set)
                {
                    sum += this.value_set[v].probability;
                    if (sum >= p)
                    {
                        result = this.value_set[v];
                        break;
                    }
                }
            }

            // Set timeout for stopping animatino and showing result
            var self = this;
            window.setTimeout(function(){
                self.show_result(result);
            }, roll_duration);
        }
        this.btn_throw.onclick = function(){self.throw_dice();return false;};


        // Animation internals

        this.animate_interval = 0;
        this.animate_step_counter = null;

        // Start animation lasting <roll_duration> microseconds.
        this.start_animation = function(roll_duration)
        {
            // If animation is alraedy running abort.
            if (this.animate_interval != 0)
            {
                return;
            }

            // Each rotation is shown in <nsteps> steps.
            var nsteps = this.animation.nsteps;

            // Each <chance_image_all> steps the image is swapped
            var change_image_all = this.animation.change_image_all;


            // compute step timing and according rotation to get a nice rounded off rotation

            // at least 1 rotation, number of rotations dependent on roll/animation duration
            var rotations = 1 + Math.floor(roll_duration / 2000);

            var time_per_rot = roll_duration / rotations;
            var time_per_step = time_per_rot / nsteps;

            // Degree change per step
            var rotate_step = this.animation.rotate ? 360 / nsteps : 0;

            // Setup variables for actual animation tick function
            var self = this;
            this.animation_step_counter = 0;

            // .. and start ticker.
            this.animate_interval = window.setInterval(function(){
                self.animation_tick(change_image_all, rotate_step);
            },time_per_step);
        }

        // Animation Step / Tick / Redraw function
        this.animation_tick = function(change_image_all,rotate_step)
        {
            // swap image
            if (this.animation_step_counter == 0 && this.animation.swap_images)
            {

                var img_src;

                // if animation images are set, pick from these..
                if (this.animation.images !== undefined && this.animation.images.length > 0)
                {
                    var img_i = Math.floor(this.animation.images.length * Math.random()) % this.animation.images.length;
                    img_src = this.animation.images[img_i];
```

```
            }

            // .. otherwise pick from result images.
            else
            {
                var img_i = Math.floor(this.value_set.length * Math.random()) % this.value_set.length;
                img_src = this.value_set[img_i].key + this.images.ext;
            }

            // update image
            this.dice_img.src = this.images.base_url + img_src;
        }

        this.animation_step_counter = (this.animation_step_counter + 1) % change_image_all;


        // Rotate image
        if (this.animation.rotate)
        {
            // Default rotation
            var deg = 0;

            // If image is already rotated, get current rotation degree.
            if (this.dice_img.style.transform !== undefined)
            {
                deg = +this.dice_img.style.transform.replace(/rotate\(|deg\)/g,'');

            }

            // Update rotation degree
            deg += rotate_step;

            // Set CSS rotation image
            this.dice_img.style.transform = 'rotate('+deg+'deg)';
            this.dice_img.style['-webkit-transform'] = 'rotate('+deg+'deg)';
            this.dice_img.style['-ms-transform'] = 'rotate('+deg+'deg)';
            this.dice_img.style['-moz-transform'] = 'rotate('+deg+'deg)';
            this.dice_img.style['-o-transform'] = 'rotate('+deg+'deg)';

            //var iecos = Math.cos(deg * Math.PI / 180);
            //var iesin = Math.sin(deg * Math.PI / 180);
            //this.dice_img.style['filter'] =
            'progid:DXImageTransform.Microsoft.Matrix(M11='+iecos+',M21='+iesin+',M22='+iecos+',M12='+(-iesin)+',
            sizingMethod="auto expand")';
        }

    }

    // Stop all animation
    this.stop_animation = function()
    {
        // If animation ticker is not active, abort
        if (this.animate_interval == 0)
        {
            return;
        }

        // Clear animatino ticker
        window.clearInterval(this.animate_interval);
        this.animate_interval = 0;

        // Set rotation degrees to original state (0 degrees)
        if (this.animation.rotate)
        {
            this.dice_img.style.transform = 'rotate(0deg)';
            this.dice_img.style['-webkit-transform'] = 'rotate(0deg)';
            this.dice_img.style['-ms-transform'] = 'rotate(0deg)';
            this.dice_img.style['-moz-transform'] = 'rotate(0deg)';
            this.dice_img.style['-o-transform'] = 'rotate(0deg)';

            //this.dice_img.style['filter'] = 'progid:DXImageTransform.Microsoft.Matrix(1,0,1,0, "auto expand")';
        }
    }

    // Show throw result and enabled controls again.
    this.show_result = function(result)
    {
        this.stop_animation();

        this.dice_img.src = this.images.base_url + result.key + this.images.ext;

        this.is_throwing = false;
        this.done_throws++;

        // If a callback function has been set, call it.
        if (this.result_callback != null)
        {
            this.result_callback(this.done_throws, result.key);
        }

        if (this.possible_throws == 0 || this.done_throws < this.possible_throws)
        {
            this.activate_controls();
        }
        else if (typeof this.finished_callback != 'undefined')
        {
            this.finished_callback();
```

```
            }
        }
    }

    // Alternative initialization function to be used when not
    function dice_create_here(btn_throw_label, options)
    {
        // Generate random HTML tag ids.
        var dice_id         = 'dice_' + Math.floor(1000*Math.random());
        var btn_throw_id    = 'btn_throw_' + Math.floor(1000*Math.random());
//        var btn_reset_id    = 'btn_reset_' + Math.floor(1000*Math.random());

        // Add required HTML elements to document.
        document.write('<div id="'+dice_id+'"></div>');
        document.write('<button id="'+btn_throw_id+'">'+btn_throw_label+'</button>');

//        if (btn_reset_label != null)
//        {
//            document.write('<button id="'+btn_reset_id+'">'+btn_reset_label+'</button>');
//            options.btn_reset_id = btn_reset_id;
//        }

        // Initialize dice by standard method. Use little safety delay.
        window.setTimeout(function(){new Dice(dice_id, btn_throw_id, options);},10);
    }


    // If you load this file dynamically and want to run your custom code when it has finished
    // loading, you can defined <dive_js_load_callback> as your callback function
    if (typeof dice_js_load_callback != 'undefined')
    {
        dice_js_load_callback();
    }
```

A.3.2 Pick-a-number fields

```
<script src="https://www.descil.ethz.ch/projects/1305-SensQuest/pickanumber/pickanumber.js" type="text/javascript"></script>

<strong>1. Please, think of a number between 1 and 12.</strong>
<br><br>
<table id="pan-table" border="1" cellpadding="2"></table>
<br><br>
<strong>2. Got your number?</strong><br>      If yes, click the "Show instruction!" button:   
<button id="show">Show instruction!</button>

    <script type="text/javascript">

        var my_fields = [
                        {multiplicity: 9, hidden:'<h1>{{index}}</h1>', visible: '<h1>{{index}}</h1>Answer<br>question'},
                        {multiplicity: 2, hidden:'<h1>{{index}}</h1>', visible: '<h1>{{index}}</h1>Directly<br>tick <u>Yes</u>'},
                        {multiplicity: 1, hidden:'<h1>{{index}}</h1>', visible: '<h1>{{index}}</h1>Directly<br>tick <u>No</u>'}
                    ];

        var my_options = {
            shuffle: true,
            dim: [2,6],
            field_style : {
                height: '70px',
                width:  '70px',
                'backgroundColor' : 'white',
                'verticalAlign': 'top',
                'textAlign'    : 'center',
                'color' : 'black',
                'fontWeight'  : '400'
            }
        };

        new PickANumber('pan-table','show',my_fields,my_options);
    </script>
```

In addition, the library pickanumber.js was called:

```
/**
 * pickanumber.js
 *
 * For demo see pickanumber.html
 *
 * @author Philip Tschiemer, tschiemer@filou.se
 * @version 2013-06-19
 */

function PickANumber(table_id, btn_show_id, fields, options)
{
    var self = this;

    this.table = document.getElementById(table_id);
    this.btn_show = document.getElementById(btn_show_id);

    this.options = {
        shuffle : false,
        //dim : [1,fields.length],
        field_style : {
            'vertical-align': 'top',
            'text-align'    : 'center'
        }
    };

    if (typeof options != 'undefined')
    {
        for(var key in options)
        {
            this.options[key] = options[key];
        }
    }

    // Create collection of all fields
    var has_multiplicities = false;
    this.fields = [];
    for(var f = 0; f < fields.length; f++)
    {
        var field = fields[f];
        var i = typeof field.multiplicity == 'number' ? field.multiplicity : 1;

        while(0 < i--)
        {
            var copy = {};
            for( var k in field)
            {
                copy[k] = field[k];
            }
            this.fields.push(copy);
        }
    }

    if (typeof this.options.dim == 'undefined')
    {
        this.options.dim = [1,this.fields.length];
    }
    else if (typeof this.options.dim == 'number' && this.options.dim == 2)
    {
        var r = Math.ceil(Math.sqrt(this.fields.length));
        this.options.dim = [r,r];
    }
    else if (this.options.dim[0] * this.options.dim[1] < this.fields.length)
    {
        alert('Trying to create a '+this.options.dim[0]+'x'+this.options.dim[1]+' Matrix for a total of '+this.fields.length+'
        elements. Please adjust matrix dimensions to fit fields.');
        return;
    }

    // Shuffle fields randomly if so wanted
    if (this.options.shuffle)
    {
        // assign a random real number in [0,1] to each field
        for (var s = 0; s < this.fields.length; s++)
        {
            this.fields[s].sort_index = Math.random();
        }

        // sort fields according to random number, ie pick random permutation of fields
        this.fields.sort(function(a,b){
            return a.sort_index - b.sort_index;
        });
    }

    // Set index of each field according to position
    for(var f =0; f < this.fields.length; f++)
    {
        this.fields[f].index = f+1;
    }


    var tbody = document.createElement('tbody');
    this.table.appendChild(tbody);

    for (var r = 1, f=0; r <= this.options.dim[0]; r++)
    {
        var tr = document.createElement('tr');
        tbody.appendChild(tr);
```

```
        for (var c = 1; c <= this.options.dim[1]; c++)
        {
            var td = document.createElement('td');

            for(var s in this.options.field_style)
            {
                td.style[s] = this.options.field_style[s];
            }

            if (f >= this.fields.length)
            {
                td.className = 'pan-empty pan-row-'+r+' pan-col-'+c;
            }
            else
            {

                var field = this.fields[f];

                field.td = td;

                var content = '';
                switch(field.type)
                {
                    default:
                    case 'asis':
                        content = field.hidden;
                        break;
                }
                field.td.innerHTML = content.replace(/\{\{index\}\}/g,field.index);
                field.td.className = 'pan-index-'+field.index+' pan-row-'+r+' pan-col-'+c;

            }


            tr.appendChild(td);

            f++;
        }
    }



    this.show = function()
    {
        for(var f = 0; f < this.fields.length; f++)
        {
            var field = this.fields[f];

            var content = '';
            switch (field.type)
            {
                default:
                case 'asis':
                    content = field.visible;
                    break;
            }
            field.td.innerHTML = content.replace(/\{\{index\}\}/g,field.index);
        }

        this.btn_show.disabled = true;
    }
    this.btn_show.onclick = function(){self.show();return false;}
}

function pickanumber_create_here(btn_show_label,fields,options)
{
    var table_id    = 'pan_table_'+Math.floor(1000*Math.random());
    var btn_show_id = 'pan_show_'+Math.floor(1000*Math.random());

    document.write('<table id="'+table_id+'"></table>');
    document.write('<button id="'+btn_show_id+'">'+btn_show_label+'</button>');

    new PickANumber(table_id,btn_show_id,fields,options);
}
```

A.4 Codebook

id — Access ID

		Count	Percent	Valid %	Cum. %
Valid	1	1	0.02	0.02	0.02
	2	1	0.02	0.02	0.03
	4	1	0.02	0.02	0.05
	5	1	0.02	0.02	0.06
	6	1	0.02	0.02	0.08
	⋮	⋮	⋮	⋮	⋮
	6631	1	0.02	0.02	99.94
	6632	1	0.02	0.02	99.95
	6633	1	0.02	0.02	99.97
	6634	1	0.02	0.02	99.98
	6635	1	0.02	0.02	100.00
	Total	6505	100.00	100.00	

completed — Survey completion status

			Count	Percent	Valid %	Cum. %
Valid	0	discontinued	44	0.68	0.68	0.68
	1	completed	6461	99.32	99.32	100.00
	Total		6505	100.00	100.00	

startdate — Date and time of first access (GMT +01:00)

		Count	Percent	Valid %	Cum. %
Valid	26nov2013 16:51:00	1	0.02	0.02	0.02
	26nov2013 16:51:28	1	0.02	0.02	0.03
	26nov2013 16:51:30	1	0.02	0.02	0.05
	26nov2013 16:51:31	1	0.02	0.02	0.06
	26nov2013 16:51:34	1	0.02	0.02	0.08
	⋮	⋮	⋮	⋮	⋮
	05dec2013 14:32:22	1	0.02	0.02	99.94
	05dec2013 14:37:31	1	0.02	0.02	99.95
	05dec2013 14:50:51	1	0.02	0.02	99.97
	05dec2013 14:54:34	1	0.02	0.02	99.98
	05dec2013 14:58:56	1	0.02	0.02	100.00
	Total	6505	100.00	100.00	

enddate — Date and time of last access (GMT +01:00)

		Count	Percent	Valid %	Cum. %
Valid	26nov2013 16:55:12	1	0.02	0.02	0.02
	26nov2013 16:56:02	1	0.02	0.02	0.03
	26nov2013 16:56:06	1	0.02	0.02	0.05
	26nov2013 16:56:09	1	0.02	0.02	0.06
	26nov2013 16:56:22	1	0.02	0.02	0.08
	⋮	⋮	⋮	⋮	⋮
	05dec2013 14:40:59	1	0.02	0.02	99.94
	05dec2013 14:45:45	1	0.02	0.02	99.95
	05dec2013 14:58:38	1	0.02	0.02	99.97
	05dec2013 15:02:43	1	0.02	0.02	99.98
	05dec2013 15:13:32	1	0.02	0.02	100.00
	Total	6505	100.00	100.00	

ip — IP address

		Count	Percent	Valid %	Cum. %
Valid	1.162.46.95	1	0.02	0.02	0.02
	100.1.111.195	1	0.02	0.02	0.03
	100.1.27.172	1	0.02	0.02	0.05
	100.1.32.135	1	0.02	0.02	0.06
	100.1.91.54	1	0.02	0.02	0.08
	⋮	⋮	⋮	⋮	⋮
	99.95.172.195	1	0.02	0.02	99.92
	99.95.66.180	1	0.02	0.02	99.94
	99.95.7.30	1	0.02	0.02	99.95
	99.99.201.133	2	0.03	0.03	99.98
	99.99.42.102	1	0.02	0.02	100.00
	Total	6505	100.00	100.00	

city — IP geolocation: city

		Count	Percent	Valid %	Cum. %
Valid	Aberdeen	5	0.08	0.08	0.08
	Abilene	1	0.02	0.02	0.09
	Absecon	2	0.03	0.03	0.13
	Abu Dhabi	1	0.02	0.02	0.14
	Accra	1	0.02	0.02	0.16
	⋮	⋮	⋮	⋮	⋮
	Zapopan	1	0.02	0.02	99.92
	Zebulon	1	0.02	0.02	99.94
	Zephyrhills	2	0.03	0.03	99.97
	Zion	1	0.02	0.02	99.98
	Zionsville	1	0.02	0.02	100.00
	Total	6346	97.56	100.00	
Missing		159	2.44		
Total		6505	100.00		

postalcode — IP geolocation: postalcode

		Count	Percent	Valid %	Cum. %
Valid	01003	2	0.03	0.05	0.05
	01027	1	0.02	0.02	0.07
	01040	1	0.02	0.02	0.09
	01050	1	0.02	0.02	0.12
	01056	1	0.02	0.02	0.14
	⋮	⋮	⋮	⋮	⋮
	EH19	1	0.02	0.02	99.91
	J1X	1	0.02	0.02	99.93
	KY3	1	0.02	0.02	99.95
	N1	1	0.02	0.02	99.98
	R7A	1	0.02	0.02	100.00
	Total	4317	66.36	100.00	
Missing		2188	33.64		
Total		6505	100.00		

region — IP geolocation: region

			Count	Percent	Valid %	Cum. %
Valid	00		4	0.06	0.06	0.06
	01		5	0.08	0.08	0.14
	02		10	0.15	0.16	0.30
	03		5	0.08	0.08	0.38
	04		2	0.03	0.03	0.41
	⋮		⋮	⋮	⋮	⋮
	VT		15	0.23	0.24	94.65
	WA		170	2.61	2.67	97.32
	WI		115	1.77	1.81	99.12
	WV		47	0.72	0.74	99.86
	WY		9	0.14	0.14	100.00
	Total		6369	97.91	100.00	
Missing			136	2.09		
Total			6505	100.00		

areacode — IP geolocation: areacode

			Count	Percent	Valid %	Cum. %
Valid	3		1	0.02	0.02	0.02
	201		32	0.49	0.51	0.53
	202		23	0.35	0.37	0.90
	203		30	0.46	0.48	1.38
	205		28	0.43	0.45	1.83
	⋮		⋮	⋮	⋮	⋮
	973		33	0.51	0.53	98.84
	978		25	0.38	0.40	99.24
	979		11	0.17	0.18	99.42
	985		12	0.18	0.19	99.61
	989		24	0.37	0.39	100.00
	Total		6222	95.65	100.00	
Missing	.a	not available	283	4.35		
Total			6505	100.00		

country — IP geolocation: country

		Count	Percent	Valid %	Cum. %
Valid	Anonymous Proxy	1	0.02	0.02	0.02
	Antigua and Barbuda	1	0.02	0.02	0.03
	Argentina	1	0.02	0.02	0.05
	Australia	3	0.05	0.05	0.09
	Belgium	1	0.02	0.02	0.11
	⋮	⋮	⋮	⋮	⋮
	Thailand	3	0.05	0.05	2.03
	Turkey	2	0.03	0.03	2.06
	United Arab Emirates	2	0.03	0.03	2.09
	United Kingdom	9	0.14	0.14	2.23
	United States	6360	97.77	97.77	100.00
	Total	6505	100.00	100.00	

loclat — IP geolocation: latitude

		Count	Percent	Valid %	Cum. %
Valid	-37.883301	1	0.02	0.02	0.02
	-37.833298	1	0.02	0.02	0.03
	-34.587494	1	0.02	0.02	0.05
	-29.683304	1	0.02	0.02	0.06
	-28.813293	1	0.02	0.02	0.08
	⋮	⋮	⋮	⋮	⋮
	61.218094	3	0.05	0.05	99.94
	61.223099	1	0.02	0.02	99.95
	61.523499	1	0.02	0.02	99.97
	62	1	0.02	0.02	99.98
	64.818497	1	0.02	0.02	100.00
	Total	6461	99.32	100.00	
Missing	.	44	0.68		
Total		6505	100.00		

loclong — IP geolocation: longitude

		Count	Percent	Valid %	Cum. %
Valid	-158.0862	1	0.02	0.02	0.02
	-158.0183	3	0.05	0.05	0.06
	-157.9371	1	0.02	0.02	0.08
	-157.93491	1	0.02	0.02	0.09
	-157.89819	1	0.02	0.02	0.11
	⋮	⋮	⋮	⋮	⋮
	137.3833	1	0.02	0.02	99.94
	139.45081	1	0.02	0.02	99.95
	145.03329	1	0.02	0.02	99.97
	145.1333	1	0.02	0.02	99.98
	153.27579	1	0.02	0.02	100.00
	Total	6461	99.32	100.00	
Missing	.	44	0.68		
Total		6505	100.00		

q2 — Screening question: category

			Count	Percent	Valid %	Cum. %
Valid	1	Decision making	146	2.24	2.25	2.25
	2	Effort in answering questions	33	0.51	0.51	2.76
	3	Payment and answering questions	9	0.14	0.14	2.89
	4	Other	6307	96.96	97.11	100.00
	Total		6495	99.85	100.00	
Missing	.b	break-off	10	0.15		
Total			6505	100.00		

q2txt — Screening question: other (text)

		Count	Percent	Valid %	Cum. %
Valid	"Got it"	12	0.18	0.19	0.19
	'Got It'	4	0.06	0.06	0.25
	'Got it'	68	1.05	1.08	1.34
	⋮	⋮	⋮	⋮	⋮
	paying attention	1	0.02	0.02	99.84
	‘Got it	2	0.03	0.03	99.87
	‘Got it’	8	0.12	0.13	100.00
	Total	6291	96.71	100.00	
Missing		214	3.29		
Total		6505	100.00		

q3_1 — Risk attitude (GSOEP 11-point scale)

			Count	Percent	Valid %	Cum. %
Valid	0	unwilling to take risks	76	1.17	1.17	1.17
	1		156	2.40	2.41	3.58
	2		498	7.66	7.68	11.26
	3		785	12.07	12.11	23.37
	4		592	9.10	9.13	32.50
	5		604	9.29	9.32	41.81
	6		971	14.93	14.98	56.79
	7		1279	19.66	19.73	76.51
	8		872	13.41	13.45	89.96
	9		250	3.84	3.86	93.82
	10	fully prepared to take risks	401	6.16	6.18	100.00
	Total		6484	99.68	100.00	
Missing	.a	no answer	7	0.11		
	.b	break-off	14	0.22		
	Total		21	0.32		
Total			6505	100.00		

q3_2 — Native English speaker

			Count	Percent	Valid %	Cum. %
Valid	0	no	173	2.66	2.67	2.67
	1	yes	6313	97.05	97.33	100.00
	Total		6486	99.71	100.00	
Missing	.a	no answer	5	0.08		
	.b	break-off	14	0.22		
	Total		19	0.29		
Total			6505	100.00		

q3_3 — US citizen

			Count	Percent	Valid %	Cum. %
Valid	0	no	110	1.69	1.70	1.70
	1	yes	6377	98.03	98.30	100.00
	Total		6487	99.72	100.00	
Missing	.a	no answer	4	0.06		
	.b	break-off	14	0.22		
	Total		18	0.28		
Total			6505	100.00		

dicegame — Dice game assignment

			Count	Percent	Valid %	Cum. %
Valid	1	prediction	3245	49.88	50.02	50.02
	2	roll-a-six	3243	49.85	49.98	100.00
	Total		6488	99.74	100.00	
Missing	.b	break-off	17	0.26		
Total			6505	100.00		

q6 — Dice game response

			Count	Percent	Valid %	Cum. %
Valid	0	wrong/no	4340	66.72	67.04	67.04
	1	right/yes	2134	32.81	32.96	100.00
	Total		6474	99.52	100.00	
Missing	.a	no answer	10	0.15		
	.b	break-off	21	0.32		
	Total		31	0.48		
Total			6505	100.00		

q6_rollcount — Dice roll counter

			Count	Percent	Valid %	Cum. %
Valid	0	no single roll executed/recorded	125	1.92	1.93	1.93
	1		5891	90.56	90.85	92.78
	2		187	2.87	2.88	95.67
	3		94	1.45	1.45	97.12
	4		52	0.80	0.80	97.92
	⋮		⋮	⋮	⋮	⋮
	14		5	0.08	0.08	99.78
	15		1	0.02	0.02	99.80
	16		3	0.05	0.05	99.85
	17		2	0.03	0.03	99.88
	20		8	0.12	0.12	100.00
	Total		6484	99.68	100.00	
Missing	.b	break-off	21	0.32		
Total			6505	100.00		

q6_roll1 — Predefined outcome roll 1

			Count	Percent	Valid %	Cum. %
Valid	1		1077	16.56	16.56	16.56
	2		1153	17.72	17.72	34.28
	3		1081	16.62	16.62	50.90
	4		1082	16.63	16.63	67.53
	5		1052	16.17	16.17	83.70
	6		1060	16.30	16.30	100.00
	Total		6505	100.00	100.00	

q6_roll2 — Predefined outcome roll 2

			Count	Percent	Valid %	Cum. %
Valid	1		1096	16.85	16.85	16.85
	2		1052	16.17	16.17	33.02
	3		1086	16.69	16.69	49.72
	4		1059	16.28	16.28	66.00
	5		1118	17.19	17.19	83.18
	6		1094	16.82	16.82	100.00
	Total		6505	100.00	100.00	

q6_roll3 — Predefined outcome roll 3

		Count	Percent	Valid %	Cum. %
Valid	1	1131	17.39	17.39	17.39
	2	1074	16.51	16.51	33.90
	3	1084	16.66	16.66	50.56
	4	1095	16.83	16.83	67.39
	5	1061	16.31	16.31	83.70
	6	1060	16.30	16.30	100.00
	Total	6505	100.00	100.00	

q6_roll4 — Predefined outcome roll 4

		Count	Percent	Valid %	Cum. %
Valid	1	1072	16.48	16.48	16.48
	2	1085	16.68	16.68	33.16
	3	1078	16.57	16.57	49.73
	4	1073	16.50	16.50	66.23
	5	1107	17.02	17.02	83.24
	6	1090	16.76	16.76	100.00
	Total	6505	100.00	100.00	

q6_roll5 — Predefined outcome roll 5

		Count	Percent	Valid %	Cum. %
Valid	1	1078	16.57	16.57	16.57
	2	1090	16.76	16.76	33.33
	3	1089	16.74	16.74	50.07
	4	1090	16.76	16.76	66.83
	5	1097	16.86	16.86	83.69
	6	1061	16.31	16.31	100.00
	Total	6505	100.00	100.00	

q6_roll6 — Predefined outcome roll 6

		Count	Percent	Valid %	Cum. %
Valid	1	1095	16.83	16.83	16.83
	2	1105	16.99	16.99	33.82
	3	1063	16.34	16.34	50.16
	4	1092	16.79	16.79	66.95
	5	1079	16.59	16.59	83.54
	6	1071	16.46	16.46	100.00
	Total	6505	100.00	100.00	

q6_roll7 — Predefined outcome roll 7

		Count	Percent	Valid %	Cum. %
Valid	1	1100	16.91	16.91	16.91
	2	1104	16.97	16.97	33.88
	3	1095	16.83	16.83	50.71
	4	1092	16.79	16.79	67.50
	5	1048	16.11	16.11	83.61
	6	1066	16.39	16.39	100.00
	Total	6505	100.00	100.00	

q6_roll8 — Predefined outcome roll 8

		Count	Percent	Valid %	Cum. %
Valid	1	1076	16.54	16.54	16.54
	2	1107	17.02	17.02	33.56
	3	1060	16.30	16.30	49.85
	4	1076	16.54	16.54	66.40
	5	1119	17.20	17.20	83.60
	6	1067	16.40	16.40	100.00
	Total	6505	100.00	100.00	

q6_roll9 — Predefined outcome roll 9

		Count	Percent	Valid %	Cum. %
Valid	1	1077	16.56	16.56	16.56
	2	1090	16.76	16.76	33.31
	3	1060	16.30	16.30	49.61
	4	1100	16.91	16.91	66.52
	5	1078	16.57	16.57	83.09
	6	1100	16.91	16.91	100.00
	Total	6505	100.00	100.00	

q6_roll10 — Predefined outcome roll 10

		Count	Percent	Valid %	Cum. %
Valid	1	1063	16.34	16.34	16.34
	2	1084	16.66	16.66	33.01
	3	1104	16.97	16.97	49.98
	4	1036	15.93	15.93	65.90
	5	1103	16.96	16.96	82.86
	6	1115	17.14	17.14	100.00
	Total	6505	100.00	100.00	

q6_roll11 — Predefined outcome roll 11

		Count	Percent	Valid %	Cum. %
Valid	1	1054	16.20	16.20	16.20
	2	1096	16.85	16.85	33.05
	3	1099	16.89	16.89	49.95
	4	1085	16.68	16.68	66.63
	5	1076	16.54	16.54	83.17
	6	1095	16.83	16.83	100.00
	Total	6505	100.00	100.00	

q6_roll12 — Predefined outcome roll 12

		Count	Percent	Valid %	Cum. %
Valid	1	1091	16.77	16.77	16.77
	2	1123	17.26	17.26	34.04
	3	1096	16.85	16.85	50.88
	4	1054	16.20	16.20	67.09
	5	1062	16.33	16.33	83.41
	6	1079	16.59	16.59	100.00
	Total	6505	100.00	100.00	

q6_roll13 — Predefined outcome roll 13

		Count	Percent	Valid %	Cum. %
Valid	1	1092	16.79	16.79	16.79
	2	1099	16.89	16.89	33.68
	3	1062	16.33	16.33	50.01
	4	1080	16.60	16.60	66.61
	5	1085	16.68	16.68	83.29
	6	1087	16.71	16.71	100.00
	Total	6505	100.00	100.00	

q6_roll14 — Predefined outcome roll 14

		Count	Percent	Valid %	Cum. %
Valid	1	1048	16.11	16.11	16.11
	2	1083	16.65	16.65	32.76
	3	1072	16.48	16.48	49.24
	4	1120	17.22	17.22	66.46
	5	1100	16.91	16.91	83.37
	6	1082	16.63	16.63	100.00
	Total	6505	100.00	100.00	

q6_roll15 — Predefined outcome roll 15

		Count	Percent	Valid %	Cum. %
Valid	1	1102	16.94	16.94	16.94
	2	1096	16.85	16.85	33.79
	3	1084	16.66	16.66	50.45
	4	1073	16.50	16.50	66.95
	5	1072	16.48	16.48	83.43
	6	1078	16.57	16.57	100.00
	Total	6505	100.00	100.00	

q6_roll16 — Predefined outcome roll 16

		Count	Percent	Valid %	Cum. %
Valid	1	1082	16.63	16.63	16.63
	2	1108	17.03	17.03	33.67
	3	1052	16.17	16.17	49.84
	4	1086	16.69	16.69	66.53
	5	1124	17.28	17.28	83.81
	6	1053	16.19	16.19	100.00
	Total	6505	100.00	100.00	

q6_roll17 — Predefined outcome roll 17

		Count	Percent	Valid %	Cum. %
Valid	1	1062	16.33	16.33	16.33
	2	1084	16.66	16.66	32.99
	3	1077	16.56	16.56	49.55
	4	1065	16.37	16.37	65.92
	5	1113	17.11	17.11	83.03
	6	1104	16.97	16.97	100.00
	Total	6505	100.00	100.00	

q6_roll18 — Predefined outcome roll 18

		Count	Percent	Valid %	Cum. %
Valid	1	1079	16.59	16.59	16.59
	2	1065	16.37	16.37	32.96
	3	1073	16.50	16.50	49.45
	4	1084	16.66	16.66	66.12
	5	1087	16.71	16.71	82.83
	6	1117	17.17	17.17	100.00
	Total	6505	100.00	100.00	

q6_roll19 — Predefined outcome roll 19

		Count	Percent	Valid %	Cum. %
Valid	1	1082	16.63	16.63	16.63
	2	1100	16.91	16.91	33.54
	3	1111	17.08	17.08	50.62
	4	1054	16.20	16.20	66.83
	5	1095	16.83	16.83	83.66
	6	1063	16.34	16.34	100.00
	Total	6505	100.00	100.00	

q6_roll20 — Predefined outcome roll 20

		Count	Percent	Valid %	Cum. %
Valid	1	1088	16.73	16.73	16.73
	2	1054	16.20	16.20	32.93
	3	1104	16.97	16.97	49.90
	4	1068	16.42	16.42	66.32
	5	1092	16.79	16.79	83.11
	6	1099	16.89	16.89	100.00
	Total	6505	100.00	100.00	

q7_1 — Satisfaction with dice game result

			Count	Percent	Valid %	Cum. %
Valid	0	not at all satisfied	1707	26.24	26.35	26.35
	1		453	6.96	6.99	33.34
	2		528	8.12	8.15	41.49
	3		432	6.64	6.67	48.16
	4		225	3.46	3.47	51.64
	5		625	9.61	9.65	61.28
	6		153	2.35	2.36	63.65
	7		194	2.98	2.99	66.64
	8		230	3.54	3.55	70.19
	9		218	3.35	3.37	73.56
	10	completely satisfied	1713	26.33	26.44	100.00
	Total		6478	99.58	100.00	
Missing	.a	no answer	5	0.08		
	.b	break-off	22	0.34		
	Total		27	0.42		
Total			6505	100.00		

q7_2 — Actual happiness

			Count	Percent	Valid %	Cum. %
Valid	0	not at all happy	641	9.85	9.89	9.89
	1		237	3.64	3.66	13.55
	2		407	6.26	6.28	19.83
	3		509	7.82	7.85	27.69
	4		522	8.02	8.06	35.74
	5		1069	16.43	16.50	52.24
	6		467	7.18	7.21	59.44
	7		502	7.72	7.75	67.19
	8		549	8.44	8.47	75.66
	9		350	5.38	5.40	81.06
	10	extremely happy	1227	18.86	18.94	100.00
	Total		6480	99.62	100.00	
Missing	.a	no answer	3	0.05		
	.b	break-off	22	0.34		
	Total		25	0.38		
Total			6505	100.00		

q8_1 — BFI-10: Extraversion

			Count	Percent	Valid %	Cum. %
Valid	2	minimal score	453	6.96	7.00	7.00
	3		727	11.18	11.23	18.23
	4		1059	16.28	16.36	34.59
	5		918	14.11	14.18	48.77
	6		1322	20.32	20.42	69.20
	7		687	10.56	10.61	79.81
	8		658	10.12	10.17	89.97
	9		386	5.93	5.96	95.94
	10	maximal score	263	4.04	4.06	100.00
	Total		6473	99.51	100.00	
Missing	.a	no answer	7	0.11		
	.b	break-off	25	0.38		
	Total		32	0.49		
Total			6505	100.00		

q8_2 — BFI-10: Agreeableness

			Count	Percent	Valid %	Cum. %
Valid	2	minimal score	66	1.01	1.02	1.02
	3		184	2.83	2.85	3.87
	4		456	7.01	7.06	10.93
	5		552	8.49	8.55	19.48
	6		1314	20.20	20.35	39.83
	7		1263	19.42	19.56	59.39
	8		1274	19.58	19.73	79.12
	9		869	13.36	13.46	92.58
	10	maximal score	479	7.36	7.42	100.00
	Total		6457	99.26	100.00	
Missing	.a	no answer	23	0.35		
	.b	break-off	25	0.38		
	Total		48	0.74		
Total			6505	100.00		

q8_3 — BFI-10: Conscientiousness

			Count	Percent	Valid %	Cum. %
Valid	2	minimal score	9	0.14	0.14	0.14
	3		62	0.95	0.96	1.10
	4		200	3.07	3.10	4.20
	5		507	7.79	7.86	12.06
	6		1184	18.20	18.35	30.40
	7		1280	19.68	19.84	50.24
	8		1203	18.49	18.64	68.88
	9		1045	16.06	16.19	85.08
	10	maximal score	963	14.80	14.92	100.00
	Total		6453	99.20	100.00	
Missing	.a	no answer	27	0.42		
	.b	break-off	25	0.38		
	Total		52	0.80		
Total			6505	100.00		

q8_4 — BFI-10: Neuroticism

			Count	Percent	Valid %	Cum. %
Valid	2	minimal score	468	7.19	7.25	7.25
	3		593	9.12	9.18	16.43
	4		1035	15.91	16.03	32.46
	5		900	13.84	13.94	46.40
	6		1175	18.06	18.20	64.60
	7		785	12.07	12.16	76.75
	8		781	12.01	12.10	88.85
	9		482	7.41	7.46	96.31
	10	maximal score	238	3.66	3.69	100.00
	Total		6457	99.26	100.00	
Missing	.a	no answer	23	0.35		
	.b	break-off	25	0.38		
	Total		48	0.74		
Total			6505	100.00		

q8_5 — BFI-10: Openness

			Count	Percent	Valid %	Cum. %
Valid	2	minimal score	32	0.49	0.49	0.49
	3		99	1.52	1.53	2.03
	4		230	3.54	3.56	5.58
	5		431	6.63	6.67	12.25
	6		1192	18.32	18.43	30.68
	7		994	15.28	15.37	46.06
	8		1152	17.71	17.82	63.87
	9		1081	16.62	16.72	80.59
	10	maximal score	1255	19.29	19.41	100.00
	Total		6466	99.40	100.00	
Missing	.a	no answer	14	0.22		
	.b	break-off	25	0.38		
	Total		39	0.60		
Total			6505	100.00		

q8_1_1 — I see myself as someone who is reserved

			Count	Percent	Valid %	Cum. %
Valid	1	disagree strongly	396	6.09	6.11	6.11
	2	disagree a little	1006	15.47	15.53	21.65
	3	neither agree nor disagree	755	11.61	11.66	33.30
	4	agree a little	2872	44.15	44.34	77.64
	5	agree strongly	1448	22.26	22.36	100.00
	Total		6477	99.57	100.00	
Missing	.a	no answer	3	0.05		
	.b	break-off	25	0.38		
	Total		28	0.43		
Total			6505	100.00		

q8_2_1 — I see myself as someone who is generally trusting

			Count	Percent	Valid %	Cum. %
Valid	1	disagree strongly	300	4.61	4.64	4.64
	2	disagree a little	867	13.33	13.41	18.06
	3	neither agree nor disagree	724	11.13	11.20	29.26
	4	agree a little	2748	42.24	42.52	71.78
	5	agree strongly	1824	28.04	28.22	100.00
	Total		6463	99.35	100.00	
Missing	.a	no answer	17	0.26		
	.b	break-off	25	0.38		
	Total		42	0.65		
Total			6505	100.00		

q8_3_1 — I see myself as someone who tends to be lazy

			Count	Percent	Valid %	Cum. %
Valid	1	disagree strongly	1282	19.71	19.85	19.85
	2	disagree a little	1705	26.21	26.39	46.24
	3	neither agree nor disagree	1319	20.28	20.42	66.66
	4	agree a little	1718	26.41	26.59	93.25
	5	agree strongly	436	6.70	6.75	100.00
	Total		6460	99.31	100.00	
Missing	.a	no answer	20	0.31		
	.b	break-off	25	0.38		
	Total		45	0.69		
Total			6505	100.00		

q8_4_1 — I see myself as someone who is relaxed, handles stress well

			Count	Percent	Valid %	Cum. %
Valid	1	disagree strongly	415	6.38	6.42	6.42
	2	disagree a little	1310	20.14	20.26	26.68
	3	neither agree nor disagree	1189	18.28	18.39	45.07
	4	agree a little	2307	35.47	35.68	80.76
	5	agree strongly	1244	19.12	19.24	100.00
	Total		6465	99.39	100.00	
Missing	.a	no answer	15	0.23		
	.b	break-off	25	0.38		
	Total		40	0.61		
Total			6505	100.00		

q8_5_1 — I see myself as someone who has few artistic interests

			Count	Percent	Valid %	Cum. %
Valid	1	disagree strongly	1805	27.75	27.90	27.90
	2	disagree a little	1808	27.79	27.95	55.85
	3	neither agree nor disagree	981	15.08	15.16	71.02
	4	agree a little	1282	19.71	19.82	90.83
	5	agree strongly	593	9.12	9.17	100.00
	Total		6469	99.45	100.00	
Missing	.a	no answer	11	0.17		
	.b	break-off	25	0.38		
	Total		36	0.55		
Total			6505	100.00		

q8_1_2 — I see myself as someone who is outgoing, sociable

			Count	Percent	Valid %	Cum. %
Valid	1	disagree strongly	672	10.33	10.38	10.38
	2	disagree a little	1588	24.41	24.53	34.90
	3	neither agree nor disagree	1142	17.56	17.64	52.54
	4	agree a little	2063	31.71	31.86	84.40
	5	agree strongly	1010	15.53	15.60	100.00
	Total		6475	99.54	100.00	
Missing	.a	no answer	5	0.08		
	.b	break-off	25	0.38		
	Total		30	0.46		
Total			6505	100.00		

q8_2_2 — I see myself as someone who tends to find fault with others

			Count	Percent	Valid %	Cum. %
Valid	1	disagree strongly	937	14.40	14.47	14.47
	2	disagree a little	1891	29.07	29.21	43.68
	3	neither agree nor disagree	1385	21.29	21.39	65.08
	4	agree a little	1894	29.12	29.26	94.33
	5	agree strongly	367	5.64	5.67	100.00
	Total		6474	99.52	100.00	
Missing	.a	no answer	6	0.09		
	.b	break-off	25	0.38		
	Total		31	0.48		
Total			6505	100.00		

q8_3_2 — I see myself as someone who does a thorough job

			Count	Percent	Valid %	Cum. %
Valid	1	disagree strongly	31	0.48	0.48	0.48
	2	disagree a little	216	3.32	3.34	3.82
	3	neither agree nor disagree	727	11.18	11.23	15.05
	4	agree a little	2824	43.41	43.63	58.68
	5	agree strongly	2674	41.11	41.32	100.00
	Total		6472	99.49	100.00	
Missing	.a	no answer	8	0.12		
	.b	break-off	25	0.38		
	Total		33	0.51		
Total			6505	100.00		

q8_4_2 — I see myself as someone who gets nervous easily

			Count	Percent	Valid %	Cum. %
Valid	1	disagree strongly	805	12.38	12.44	12.44
	2	disagree a little	1557	23.94	24.06	36.50
	3	neither agree nor disagree	1155	17.76	17.85	54.35
	4	agree a little	1975	30.36	30.52	84.87
	5	agree strongly	979	15.05	15.13	100.00
	Total		6471	99.48	100.00	
Missing	.a	no answer	9	0.14		
	.b	break-off	25	0.38		
	Total		34	0.52		
Total			6505	100.00		

q8_5_2 — I see myself as someone who has an active imagination

			Count	Percent	Valid %	Cum. %
Valid	1	disagree strongly	80	1.23	1.24	1.24
	2	disagree a little	411	6.32	6.35	7.58
	3	neither agree nor disagree	822	12.64	12.69	20.27
	4	agree a little	2446	37.60	37.76	58.04
	5	agree strongly	2718	41.78	41.96	100.00
	Total		6477	99.57	100.00	
Missing	.a	no answer	3	0.05		
	.b	break-off	25	0.38		
	Total		28	0.43		
Total			6505	100.00		

q9_1 — Educational attainment: categorized

			Count	Percent	Valid %	Cum. %
Valid	1	some high school, no degree	82	1.26	1.27	1.27
	2	high school diploma	676	10.39	10.44	11.71
	3	some college, no degree	2207	33.93	34.10	45.81
	4	associate degree	683	10.50	10.55	56.37
	5	bachelor's degree	2084	32.04	32.20	88.57
	6	graduate degree	666	10.24	10.29	98.86
	7	other (please specify)	74	1.14	1.14	100.00
	Total		6472	99.49	100.00	
Missing	.a	no answer	5	0.08		
	.b	break-off	28	0.43		
	Total		33	0.51		
Total			6505	100.00		

q9_1txt — Educational attainment: other (text)

		Count	Percent	Valid %	Cum. %
Valid	37 Certificates in Information Technologies	1	0.02	1.37	1.37
	AT-CTI program for Air Traffic Control	1	0.02	1.37	2.74
	BS, RCP,(Respiratory Care Practitioner)	1	0.02	1.37	4.11
	Certificate from cosmetology	1	0.02	1.37	5.48
	Certificate of Completion (trade)	1	0.02	1.37	6.85
	⋮	⋮	⋮	⋮	⋮
	tech school	1	0.02	1.37	93.15
	tech school - completed	1	0.02	1.37	94.52
	technical school	1	0.02	1.37	95.89
	trade school	2	0.03	2.74	98.63
	trade school certificate and some college	1	0.02	1.37	100.00
	Total	73	1.12	100.00	
Missing		6432	98.88		
Total		6505	100.00		

q9_2 — Year of birth

			Count	Percent	Valid %	Cum. %
Valid	21		1	0.02	0.02	0.02
	22		2	0.03	0.03	0.05
	23		1	0.02	0.02	0.06
	25		3	0.05	0.05	0.11
	28		4	0.06	0.06	0.17
	⋮		⋮	⋮	⋮	⋮
	1992		307	4.72	4.74	92.05
	1993		243	3.74	3.75	95.80
	1994		180	2.77	2.78	98.58
	1995		88	1.35	1.36	99.94
	1996		4	0.06	0.06	100.00
	Total		6476	99.55	100.00	
Missing	.a	no answer	1	0.02		
	.b	break-off	28	0.43		
	Total		29	0.45		
Total			6505	100.00		

q9_3 — Gender

			Count	Percent	Valid %	Cum. %
Valid	1	Male	3232	49.68	49.97	49.97
	2	Female	3236	49.75	50.03	100.00
	Total		6468	99.43	100.00	
Missing	.a	no answer	9	0.14		
	.b	break-off	28	0.43		
	Total		37	0.57		
Total			6505	100.00		

q10_1 — Number of MTurk studies attended

		Count	Percent	Valid %	Cum. %
Valid	+10	2	0.03	0.03	0.03
	0	461	7.09	7.14	7.17
	04	1	0.02	0.02	7.19
	1	279	4.29	4.32	11.51
	1 I think	1	0.02	0.02	11.53
	⋮	⋮	⋮	⋮	⋮
	400	1	0.02	0.02	99.91
	5	1	0.02	0.02	99.92
	50	3	0.05	0.05	99.97
	650	1	0.02	0.02	99.98
	7	1	0.02	0.02	100.00
	Total	6454	99.22	100.00	
Missing		51	0.78		
Total		6505	100.00		

q10_2_1 — Currently employed

			Count	Percent	Valid %	Cum. %
Valid	0	not selected	2966	45.60	45.91	45.91
	1	selected	3495	53.73	54.09	100.00
	Total		6461	99.32	100.00	
Missing	.a	no answer	16	0.25		
	.b	break-off	28	0.43		
	Total		44	0.68		
Total			6505	100.00		

q10_2_2 — Currently self-employed

			Count	Percent	Valid %	Cum. %
Valid	0	not selected	5582	85.81	86.40	86.40
	1	selected	879	13.51	13.60	100.00
	Total		6461	99.32	100.00	
Missing	.a	no answer	16	0.25		
	.b	break-off	28	0.43		
	Total		44	0.68		
Total			6505	100.00		

q10_2_3 — Currently out of work and looking for work

			Count	Percent	Valid %	Cum. %
Valid	0	not selected	5688	87.44	88.04	88.04
	1	selected	773	11.88	11.96	100.00
	Total		6461	99.32	100.00	
Missing	.a	no answer	16	0.25		
	.b	break-off	28	0.43		
	Total		44	0.68		
Total			6505	100.00		

q10_2_4 — Currently homemaker

			Count	Percent	Valid %	Cum. %
Valid	0	not selected	5941	91.33	91.95	91.95
	1	selected	520	7.99	8.05	100.00
	Total		6461	99.32	100.00	
Missing	.a	no answer	16	0.25		
	.b	break-off	28	0.43		
	Total		44	0.68		
Total			6505	100.00		

q10_2_5 — Currently student

			Count	Percent	Valid %	Cum. %
Valid	0	not selected	5046	77.57	78.10	78.10
	1	selected	1415	21.75	21.90	100.00
	Total		6461	99.32	100.00	
Missing	.a	no answer	16	0.25		
	.b	break-off	28	0.43		
	Total		44	0.68		
Total			6505	100.00		

q10_2_6 — Currently retired

			Count	Percent	Valid %	Cum. %
Valid	0	not selected	6348	97.59	98.25	98.25
	1	selected	113	1.74	1.75	100.00
	Total		6461	99.32	100.00	
Missing	.a	no answer	16	0.25		
	.b	break-off	28	0.43		
	Total		44	0.68		
Total			6505	100.00		

q10_2_7 — Currently other

			Count	Percent	Valid %	Cum. %
Valid	0	not selected	6351	97.63	98.30	98.30
	1	selected	110	1.69	1.70	100.00
	Total		6461	99.32	100.00	
Missing	.a	no answer	16	0.25		
	.b	break-off	28	0.43		
	Total		44	0.68		
Total			6505	100.00		

q10_2_7txt — Currently other (text)

		Count	Percent	Valid %	Cum. %
Valid	*I accidentally put male on the previous page; I'm female.	1	0.02	0.93	0.93
	A private tutor and occasional baker	1	0.02	0.93	1.87
	Awaiting disability decision	1	0.02	0.93	2.80
	Care Giver	1	0.02	0.93	3.74
	Caregiver	1	0.02	0.93	4.67
	⋮	⋮	⋮	⋮	⋮
	unemployed, about to move	1	0.02	0.93	96.26
	unemployed, not looking for work	1	0.02	0.93	97.20
	very part time	1	0.02	0.93	98.13
	volunteer	1	0.02	0.93	99.07
	working from home (loosely employed, through a developer on MTurk)	1	0.02	0.93	100.00
	Total	107	1.64	100.00	
Missing		6398	98.36		
Total		6505	100.00		

q10_3 — Actual location: categorized

			Count	Percent	Valid %	Cum. %
Valid	1	at home	5520	84.86	85.28	85.28
	2	at workplace/office	639	9.82	9.87	95.15
	3	in a cafe/restaurant	45	0.69	0.70	95.84
	4	at school/university	154	2.37	2.38	98.22
	5	travelling	13	0.20	0.20	98.42
	6	other (please specify)	102	1.57	1.58	100.00
	Total		6473	99.51	100.00	
Missing	.a	no answer	4	0.06		
	.b	break-off	28	0.43		
	Total		32	0.49		
Total			6505	100.00		

q10_3txt — Actual location: other (text)

		Count	Percent	Valid %	Cum. %
Valid	A friend's house.	1	0.02	0.99	0.99
	Apartment	1	0.02	0.99	1.98
	At Mom's house, which used to be my home	1	0.02	0.99	2.97
	At in-laws home	1	0.02	0.99	3.96
	At my brother's house	1	0.02	0.99	4.95
	⋮	⋮	⋮	⋮	⋮
	research library	1	0.02	0.99	96.04
	sister's house	1	0.02	0.99	97.03
	visiting at son's home	1	0.02	0.99	98.02
	visiting family	1	0.02	0.99	99.01
	visiting family in their house	1	0.02	0.99	100.00
	Total	101	1.55	100.00	
Missing		6404	98.45		
Total		6505	100.00		

senstec — Sensitive question technique assignment

			Count	Percent	Valid %	Cum. %
Valid	1	DQ	810	12.45	12.51	12.51
	2	CMquest	2438	37.48	37.66	50.18
	3	UQbenf	1618	24.87	25.00	75.17
	4	FRnumb	1607	24.70	24.83	100.00
	Total		6473	99.51	100.00	
Missing	.b	break-off	32	0.49		
Total			6505	100.00		

q14 — Have you ever intentionally taken something from a store without paying for it?

			Count	Percent	Valid %	Cum. %
Valid	0	no/different	3158	48.55	48.92	48.92
	1	yes/identical	3297	50.68	51.08	100.00
	Total		6455	99.23	100.00	
Missing	.a	no answer	16	0.25		
	.b	break-off	34	0.52		
	Total		50	0.77		
Total			6505	100.00		

q14_pyes — Shoplifting: probability of direct yes (FR/UQ)

		Count	Percent	Valid %	Cum. %
Valid	0	3248	49.93	50.19	50.19
	.10872482	402	6.18	6.21	56.41
	.10933222	410	6.30	6.34	62.74
	.11008692	806	12.39	12.46	75.20
	.16666667	1605	24.67	24.80	100.00
	Total	6471	99.48	100.00	
Missing	.b break-off	34	0.52		
Total		6505	100.00		

q14_pno — Shoplifting: probability of direct no (FR/UQ)

		Count	Percent	Valid %	Cum. %
Valid	0	3248	49.93	50.19	50.19
	.08333333	1605	24.67	24.80	75.00
	.11176183	806	12.39	12.46	87.45
	.11251653	410	6.30	6.34	93.79
	.11312393	402	6.18	6.21	100.00
	Total	6471	99.48	100.00	
Missing	.b break-off	34	0.52		
Total		6505	100.00		

q14_pcm — Shoplifting: probability of unrelated yes (CM)

		Count	Percent	Valid %	Cum. %
Valid	.15946255	1228	18.88	18.98	18.98
	.1971293	1210	18.60	18.70	37.68
	1	4033	62.00	62.32	100.00
	Total	6471	99.48	100.00	
Missing	.b break-off	34	0.52		
Total		6505	100.00		

q14_uq — Shoplifting: unrelated question

		Count	Percent	Valid %	Cum. %
Valid	1 mother: birthday Jan-Feb (CM Question)	613	9.42	15.11	15.11
	2 mother: birthday 1th-6th (CM Question)	610	9.38	15.04	30.15
	3 father: birthday Jan-Feb (CM Question)	615	9.45	15.16	45.32
	4 father: birthday 1th-6th (CM Question)	600	9.22	14.79	60.11
	5 mother: birthday Jan-Jun (UQ Benford)	407	6.26	10.03	70.14
	6 mother: even birthmonth (UQ Benford)	399	6.13	9.84	79.98
	7 mother: birthday 1st-15th (UQ Benford)	410	6.30	10.11	90.09
	8 mother: even birthday (UQ Benford)	402	6.18	9.91	100.00
	Total	4056	62.35	100.00	
Missing	.b break-off	34	0.52		
	.c filter: senstec not 2 or 3	2415	37.13		
	Total	2449	37.65		
Total		6505	100.00		

q15 — Have you ever provided misleading or incorrect information on your tax return?

			Count	Percent	Valid %	Cum. %
Valid	0	no/different	3855	59.26	59.73	59.73
	1	yes/identical	2599	39.95	40.27	100.00
	Total		6454	99.22	100.00	
Missing	.a	no answer	13	0.20		
	.b	break-off	38	0.58		
	Total		51	0.78		
Total			6505	100.00		

q15_pyes — Tax evasion: probability of direct yes (FR/UQ)

		Count	Percent	Valid %	Cum. %
Valid	0	3245	49.88	50.18	50.18
	.10872482	404	6.21	6.25	56.42
	.10933222	401	6.16	6.20	62.63
	.11008692	812	12.48	12.56	75.18
	.16666667	1605	24.67	24.82	100.00
	Total	6467	99.42	100.00	
Missing	.b break-off	38	0.58		
Total		6505	100.00		

q15_pno — Tax evasion: probability of direct no (FR/UQ)

		Count	Percent	Valid %	Cum. %
Valid	0	3245	49.88	50.18	50.18
	.08333333	1605	24.67	24.82	75.00
	.11176183	812	12.48	12.56	87.55
	.11251653	401	6.16	6.20	93.75
	.11312393	404	6.21	6.25	100.00
	Total	6467	99.42	100.00	
Missing	.b break-off	38	0.58		
Total		6505	100.00		

q15_pcm — Tax evasion: probability of unrelated yes (CM)

		Count	Percent	Valid %	Cum. %
Valid	.15946255	1210	18.60	18.71	18.71
	.1971293	1226	18.85	18.96	37.67
	1	4031	61.97	62.33	100.00
	Total	6467	99.42	100.00	
Missing	.b break-off	38	0.58		
Total		6505	100.00		

q15_uq — Tax evasion: unrelated question

		Count	Percent	Valid %	Cum. %
Valid	1 mother: birthday Jan-Feb (CM Question)	603	9.27	14.88	14.88
	2 mother: birthday 1th-6th (CM Question)	612	9.41	15.10	29.98
	3 father: birthday Jan-Feb (CM Question)	607	9.33	14.98	44.95
	4 father: birthday 1th-6th (CM Question)	614	9.44	15.15	60.10
	5 mother: birthday Jan-Jun (UQ Benford)	402	6.18	9.92	70.02
	6 mother: even birthmonth (UQ Benford)	410	6.30	10.12	80.14
	7 mother: birthday 1st-15th (UQ Benford)	401	6.16	9.89	90.03
	8 mother: even birthday (UQ Benford)	404	6.21	9.97	100.00
	Total	4053	62.31	100.00	
Missing	.b break-off	38	0.58		
	.c filter: senstec not 2 or 3	2414	37.11		
	Total	2452	37.69		
Total		6505	100.00		

q16 — Did you vote in the 2012 US presidential election?

			Count	Percent	Valid %	Cum. %
Valid	0	no/different	2966	45.60	45.98	45.98
	1	yes/identical	3484	53.56	54.02	100.00
	Total		6450	99.15	100.00	
Missing	.a	no answer	15	0.23		
	.b	break-off	40	0.61		
	Total		55	0.85		
Total			6505	100.00		

q16_pyes — Voting: probability of direct yes (FR/UQ)

		Count	Percent	Valid %	Cum. %
Valid	0	3243	49.85	50.16	50.16
	.08333333	1605	24.67	24.83	74.99
	.10872482	409	6.29	6.33	81.31
	.10933222	407	6.26	6.30	87.61
	.11008692	801	12.31	12.39	100.00
	Total	6465	99.39	100.00	
Missing	.b break-off	40	0.61		
Total		6505	100.00		

q16_pno — Voting: probability of direct no (FR/UQ)

		Count	Percent	Valid %	Cum. %
Valid	0	3243	49.85	50.16	50.16
	.11176183	801	12.31	12.39	62.55
	.11251653	407	6.26	6.30	68.85
	.11312393	409	6.29	6.33	75.17
	.16666667	1605	24.67	24.83	100.00
	Total	6465	99.39	100.00	
Missing	.b break-off	40	0.61		
Total		6505	100.00		

q16_pcm — Voting: probability of unrelated yes (CM)

		Count	Percent	Valid %	Cum. %
Valid	.15946255	1216	18.69	18.81	18.81
	.1971293	1218	18.72	18.84	37.65
	1	4031	61.97	62.35	100.00
	Total	6465	99.39	100.00	
Missing	.b break-off	40	0.61		
Total		6505	100.00		

q16_uq — Voting: unrelated question

		Count	Percent	Valid %	Cum. %
Valid	1 mother: birthday Jan-Feb (CM Question)	612	9.41	15.11	15.11
	2 mother: birthday 1th-6th (CM Question)	608	9.35	15.01	30.12
	3 father: birthday Jan-Feb (CM Question)	604	9.29	14.91	45.03
	4 father: birthday 1th-6th (CM Question)	610	9.38	15.06	60.08
	5 mother: birthday Jan-Jun (UQ Benford)	402	6.18	9.92	70.01
	6 mother: even birthmonth (UQ Benford)	399	6.13	9.85	79.86
	7 mother: birthday 1st-15th (UQ Benford)	407	6.26	10.05	89.90
	8 mother: even birthday (UQ Benford)	409	6.29	10.10	100.00
	Total	4051	62.28	100.00	
Missing	.b break-off	40	0.61		
	.c filter: senstec not 2 or 3	2414	37.11		
	Total	2454	37.72		
Total		6505	100.00		

q17 — Did you honestly report whether your prediction was right?/you rolled a 6?

			Count	Percent	Valid %	Cum. %
Valid	0	no/different	2295	35.28	35.56	35.56
	1	yes/identical	4159	63.94	64.44	100.00
	Total		6454	99.22	100.00	
Missing	.a	no answer	10	0.15		
	.b	break-off	41	0.63		
	Total		51	0.78		
Total			6505	100.00		

q17_pyes — Dice game reporting: probability of direct yes (FR/UQ)

		Count	Percent	Valid %	Cum. %
Valid	0	3242	49.84	50.15	50.15
	.08333333	1605	24.67	24.83	74.98
	.10872482	402	6.18	6.22	81.20
	.10933222	400	6.15	6.19	87.39
	.11008692	815	12.53	12.61	100.00
	Total	6464	99.37	100.00	
Missing	.b break-off	41	0.63		
Total		6505	100.00		

q17_pno — Dice game reporting: probability of direct no (FR/UQ)

		Count	Percent	Valid %	Cum. %
Valid	0	3242	49.84	50.15	50.15
	.11176183	815	12.53	12.61	62.76
	.11251653	400	6.15	6.19	68.95
	.11312393	402	6.18	6.22	75.17
	.16666667	1605	24.67	24.83	100.00
	Total	6464	99.37	100.00	
Missing	.b break-off	41	0.63		
Total		6505	100.00		

q17_pcm — Dice game reporting: probability of unrelated yes (CM)

		Count	Percent	Valid %	Cum. %
Valid	.15946255	1216	18.69	18.81	18.81
	.1971293	1217	18.71	18.83	37.64
	1	4031	61.97	62.36	100.00
	Total	6464	99.37	100.00	
Missing	.b break-off	41	0.63		
Total		6505	100.00		

q17_uq — Dice game reporting: unrelated question

		Count	Percent	Valid %	Cum. %
Valid	1 mother: birthday Jan-Feb (CM Question)	607	9.33	14.99	14.99
	2 mother: birthday 1th-6th (CM Question)	605	9.30	14.94	29.93
	3 father: birthday Jan-Feb (CM Question)	609	9.36	15.04	44.96
	4 father: birthday 1th-6th (CM Question)	612	9.41	15.11	60.07
	5 mother: birthday Jan-Jun (UQ Benford)	406	6.24	10.02	70.10
	6 mother: even birthmonth (UQ Benford)	409	6.29	10.10	80.20
	7 mother: birthday 1st-15th (UQ Benford)	400	6.15	9.88	90.07
	8 mother: even birthday (UQ Benford)	402	6.18	9.93	100.00
	Total	4050	62.26	100.00	
Missing	.b break-off	41	0.63		
	.c filter: senstec not 2 or 3	2414	37.11		
	Total	2455	37.74		
Total		6505	100.00		

q18_1 — How well are respondents' anonymity and privacy protected?

			Count	Percent	Valid %	Cum. %
Valid	1	very poorly	32	0.49	0.50	0.50
	2	rather poorly	183	2.81	2.84	3.34
	3	moderately	1265	19.45	19.66	23.01
	4	rather well	2639	40.57	41.02	64.03
	5	very well	2314	35.57	35.97	100.00
	Total		6433	98.89	100.00	
Missing	.a	no answer	29	0.45		
	.b	break-off	43	0.66		
	Total		72	1.11		
Total			6505	100.00		

q18_2 — How likely could respondents' sensitive behavior be disclosed by this survey?

			Count	Percent	Valid %	Cum. %
Valid	1	impossible	752	11.56	11.65	11.65
	2	not likely	2900	44.58	44.94	56.59
	3	somewhat likely	1663	25.56	25.77	82.36
	4	quite likely	799	12.28	12.38	94.75
	5	very likely	339	5.21	5.25	100.00
	Total		6453	99.20	100.00	
Missing	.a	no answer	9	0.14		
	.b	break-off	43	0.66		
	Total		52	0.80		
Total			6505	100.00		

q19_1 — Does the special technique absolutely protect your answers?

			Count	Percent	Valid %	Cum. %
Valid	1	not at all	565	8.69	10.03	10.03
	2	a little	963	14.80	17.10	27.13
	3	moderately	1415	21.75	25.12	52.25
	4	quite a bit	1912	29.39	33.95	86.20
	5	definitely	777	11.94	13.80	100.00
	Total		5632	86.58	100.00	
Missing	.a	no answer	20	0.31		
	.b	break-off	44	0.68		
	.c	filter: DQ	809	12.44		
	Total		873	13.42		
Total			6505	100.00		

q19_2 — Do you think you properly followed the instructions for the special technique?

			Count	Percent	Valid %	Cum. %
Valid	1	not at all	14	0.22	0.25	0.25
	2	a little	53	0.81	0.94	1.19
	3	moderately	196	3.01	3.48	4.67
	4	quite a bit	917	14.10	16.28	20.95
	5	definitely	4452	68.44	79.05	100.00
	Total		5632	86.58	100.00	
Missing	.a	no answer	20	0.31		
	.b	break-off	44	0.68		
	.c	filter: DQ	809	12.44		
	Total		873	13.42		
Total			6505	100.00		

q19_3 — Did you understand how the technique protects respondents?

			Count	Percent	Valid %	Cum. %
Valid	1	not at all	269	4.14	4.78	4.78
	2	a little	522	8.02	9.27	14.05
	3	moderately	1160	17.83	20.61	34.66
	4	quite a bit	1657	25.47	29.44	64.10
	5	definitely	2021	31.07	35.90	100.00
	Total		5629	86.53	100.00	
Missing	.a	no answer	23	0.35		
	.b	break-off	44	0.68		
	.c	filter: DQ	809	12.44		
	Total		876	13.47		
Total			6505	100.00		

q19_4txt — Have you any other thoughts or remarks on the special technique? (text)

		Count	Percent	Valid %	Cum. %
Valid	"special survey technique" by itself is a meaningless phrase	1	0.02	0.04	0.04
	.	1	0.02	0.04	0.07
	/ Not really	1	0.02	0.04	0.11
	⋮	⋮	⋮	⋮	⋮
	yes	1	0.02	0.04	99.93
	you cannot answer honestly if you follow instructions	1	0.02	0.04	99.96
	you cant conclude which questions had yes or no answers because my fathers bday is in january and i did answer honestly on the dice game so my answer of yes to both those questions is identical. But what if the team viewing this survey decides to think my response was no to both questions? therefore you cannot really get the correct answers for the questions with this method employed.	1	0.02	0.04	100.00
	Total	2852	43.84	100.00	
Missing		3653	56.16		
Total		6505	100.00		

rndtest — Randomizing device test assignment

			Count	Percent	Valid %	Cum. %
Valid	1	Benford procedure	1686	25.92	26.10	26.10
	2	unrelated questions for CM Question	890	13.68	13.77	39.87
	3	unrelated questions for UQ Benford	852	13.10	13.19	53.06
	4	pick-a-number device (standard)	1504	23.12	23.28	76.33
	5	pick-a-number device (generic)	1529	23.50	23.67	100.00
	Total		6461	99.32	100.00	
Missing	.b	break-off	44	0.68		
Total			6505	100.00		

q20_1 — First digit of acquaintance's house number

			Count	Percent	Valid %	Cum. %
Valid	1		522	8.02	30.96	30.96
	2		276	4.24	16.37	47.33
	3		156	2.40	9.25	56.58
	4		189	2.91	11.21	67.79
	5		145	2.23	8.60	76.39
	⋮		⋮	⋮	⋮	⋮
	1102		1	0.02	0.06	99.76
	1150		1	0.02	0.06	99.82
	2613		1	0.02	0.06	99.88
	9192		1	0.02	0.06	99.94
	24120		1	0.02	0.06	100.00
	Total		1686	25.92	100.00	
Missing	.b	break-off	44	0.68		
	.c	filter: rndtest not 1	4775	73.41		
	Total		4819	74.08		
Total			6505	100.00		

q20_2_1 — Response unrel. question A (CM Question)

			Count	Percent	Valid %	Cum. %
Valid	0	no	709	10.90	79.66	79.66
	1	yes	181	2.78	20.34	100.00
	Total		890	13.68	100.00	
Missing	.b	break-off	44	0.68		
	.c	filter: rndtest not 2	5571	85.64		
	Total		5615	86.32		
Total			6505	100.00		

q20_2_1_uq — Wording unrel. question A (CM Question)

		Count	Percent	Valid %	Cum. %
Valid	1 mother: birthday Jan-Feb (CM Question)	230	3.54	25.84	25.84
	2 mother: birthday 1st-6th (CM Question)	233	3.58	26.18	52.02
	3 father: birthday Jan-Feb (CM Question)	221	3.40	24.83	76.85
	4 father: birthday 1st-6th (CM Question)	206	3.17	23.15	100.00
	Total	890	13.68	100.00	
Missing	.b break-off	44	0.68		
	.c filter: rndtest not 2	5571	85.64		
	Total	5615	86.32		
Total		6505	100.00		

q20_2_1_pyes — Probability yes-answer unrel. question A (CM Question)

		Count	Percent	Valid %	Cum. %
Valid	.1594625488545281522	451	6.93	50.67	50.67
	.1971293045031725366	439	6.75	49.33	100.00
	Total	890	13.68	100.00	
Missing	.b break-off	44	0.68		
	.c filter: rndtest not 2	5571	85.64		
	Total	5615	86.32		
Total		6505	100.00		

q20_2_2 — Response unrel. question B (CM Question)

			Count	Percent	Valid %	Cum. %
Valid	0	no	731	11.24	82.23	82.23
	1	yes	158	2.43	17.77	100.00
	Total		889	13.67	100.00	
Missing	.a	no answer	1	0.02		
	.b	break-off	44	0.68		
	.c	filter: rndtest not 2	5571	85.64		
	Total		5616	86.33		
Total			6505	100.00		

q20_2_2_uq — Wording unrel. question B (CM Question)

		Count	Percent	Valid %	Cum. %
Valid	1 mother: birthday Jan-Feb (CM Question)	236	3.63	26.52	26.52
	2 mother: birthday 1st-6th (CM Question)	210	3.23	23.60	50.11
	3 father: birthday Jan-Feb (CM Question)	215	3.31	24.16	74.27
	4 father: birthday 1st-6th (CM Question)	229	3.52	25.73	100.00
	Total	890	13.68	100.00	
Missing	.b break-off	44	0.68		
	.c filter: rndtest not 2	5571	85.64		
	Total	5615	86.32		
Total		6505	100.00		

q20_2_2_pyes — Probability yes-answer unrel. question B (CM Question)

		Count	Percent	Valid %	Cum. %
Valid	.1594625488545281522	451	6.93	50.67	50.67
	.1971293045031725366	439	6.75	49.33	100.00
	Total	890	13.68	100.00	
Missing	.b break-off	44	0.68		
	.c filter: rndtest not 2	5571	85.64		
	Total	5615	86.32		
Total		6505	100.00		

q20_2_3 — Response unrel. question C (CM Question)

			Count	Percent	Valid %	Cum. %
Valid	0	no	722	11.10	81.31	81.31
	1	yes	166	2.55	18.69	100.00
	Total		888	13.65	100.00	
Missing	.a	no answer	2	0.03		
	.b	break-off	44	0.68		
	.c	filter: rndtest not 2	5571	85.64		
	Total		5617	86.35		
Total			6505	100.00		

q20_2_3_uq — Wording unrel. question C (CM Question)

		Count	Percent	Valid %	Cum. %
Valid	1 mother: birthday Jan-Feb (CM Question)	236	3.63	26.52	26.52
	2 mother: birthday 1st-6th (CM Question)	220	3.38	24.72	51.24
	3 father: birthday Jan-Feb (CM Question)	208	3.20	23.37	74.61
	4 father: birthday 1st-6th (CM Question)	226	3.47	25.39	100.00
	Total	890	13.68	100.00	
Missing	.b break-off	44	0.68		
	.c filter: rndtest not 2	5571	85.64		
	Total	5615	86.32		
Total		6505	100.00		

q20_2_3_pyes — Probability yes-answer unrel. question C (CM Question)

		Count	Percent	Valid %	Cum. %
Valid	.1594625488545281522	444	6.83	49.89	49.89
	.1971293045031725366	446	6.86	50.11	100.00
	Total	890	13.68	100.00	
Missing	.b break-off	44	0.68		
	.c filter: rndtest not 2	5571	85.64		
	Total	5615	86.32		
Total		6505	100.00		

q20_2_4 — Response unrel. question D (CM Question)

			Count	Percent	Valid %	Cum. %
Valid	0	no	706	10.85	79.42	79.42
	1	yes	183	2.81	20.58	100.00
	Total		889	13.67	100.00	
Missing	.a	no answer	1	0.02		
	.b	break-off	44	0.68		
	.c	filter: rndtest not 2	5571	85.64		
	Total		5616	86.33		
Total			6505	100.00		

q20_2_4_uq — Wording unrel. question D (CM Question)

		Count	Percent	Valid %	Cum. %
Valid	1 mother: birthday Jan-Feb (CM Question)	188	2.89	21.12	21.12
	2 mother: birthday 1st-6th (CM Question)	227	3.49	25.51	46.63
	3 father: birthday Jan-Feb (CM Question)	246	3.78	27.64	74.27
	4 father: birthday 1st-6th (CM Question)	229	3.52	25.73	100.00
	Total	890	13.68	100.00	
Missing	.b break-off	44	0.68		
	.c filter: rndtest not 2	5571	85.64		
	Total	5615	86.32		
Total		6505	100.00		

q20_2_4_pyes — Probability yes-answer unrel. question D (CM Question)

		Count	Percent	Valid %	Cum. %
Valid	.1594625488545281522	434	6.67	48.76	48.76
	.1971293045031725366	456	7.01	51.24	100.00
	Total	890	13.68	100.00	
Missing	.b break-off	44	0.68		
	.c filter: rndtest not 2	5571	85.64		
	Total	5615	86.32		
Total		6505	100.00		

q20_3_1 — Response unrel. question A (UQ Benford)

			Count	Percent	Valid %	Cum. %
Valid	0	no	422	6.49	49.59	49.59
	1	yes	429	6.59	50.41	100.00
	Total		851	13.08	100.00	
Missing	.a	no answer	1	0.02		
	.b	break-off	44	0.68		
	.c	filter: rndtest not 3	5609	86.23		
	Total		5654	86.92		
Total			6505	100.00		

q20_3_1_uq — Wording unrel. question A (UQ Benford)

		Count	Percent	Valid %	Cum. %
Valid	1 mother: birthday Jan-Jun (UQ Benford)	209	3.21	24.53	24.53
	2 mother: even birthmonth (UQ Benford)	214	3.29	25.12	49.65
	3 mother: birthday 1st-15th (UQ Benford)	210	3.23	24.65	74.30
	4 mother: even birthday (UQ Benford)	219	3.37	25.70	100.00
	Total	852	13.10	100.00	
Missing	.b break-off	44	0.68		
	.c filter: rndtest not 3	5609	86.23		
	Total	5653	86.90		
Total		6505	100.00		

q20_3_1_pyes — Probability yes-answer unrel. question A (UQ Benford)

		Count	Percent	Valid %	Cum. %
Valid	.4900853542509428507	219	3.37	25.70	25.70
	.4928232612579313554	210	3.23	24.65	50.35
	.4962251107141146256	423	6.50	49.65	100.00
	Total	852	13.10	100.00	
Missing	.b break-off	44	0.68		
	.c filter: rndtest not 3	5609	86.23		
	Total	5653	86.90		
Total		6505	100.00		

q20_3_2 — Response unrel. question B (UQ Benford)

			Count	Percent	Valid %	Cum. %
Valid	0	no	445	6.84	52.29	52.29
	1	yes	406	6.24	47.71	100.00
	Total		851	13.08	100.00	
Missing	.a	no answer	1	0.02		
	.b	break-off	44	0.68		
	.c	filter: rndtest not 3	5609	86.23		
	Total		5654	86.92		
Total			6505	100.00		

q20_3_2_uq — Wording unrel. question B (UQ Benford)

		Count	Percent	Valid %	Cum. %
Valid	1 mother: birthday Jan-Jun (UQ Benford)	201	3.09	23.59	23.59
	2 mother: even birthmonth (UQ Benford)	221	3.40	25.94	49.53
	3 mother: birthday 1st-15th (UQ Benford)	218	3.35	25.59	75.12
	4 mother: even birthday (UQ Benford)	212	3.26	24.88	100.00
	Total	852	13.10	100.00	
Missing	.b break-off	44	0.68		
	.c filter: rndtest not 3	5609	86.23		
	Total	5653	86.90		
Total		6505	100.00		

q20_3_2_pyes — Probability yes-answer unrel. question B (UQ Benford)

		Count	Percent	Valid %	Cum. %
Valid	.4900853542509428507	212	3.26	24.88	24.88
	.4928232612579313554	218	3.35	25.59	50.47
	.4962251107141146256	422	6.49	49.53	100.00
	Total	852	13.10	100.00	
Missing	.b break-off	44	0.68		
	.c filter: rndtest not 3	5609	86.23		
	Total	5653	86.90		
Total		6505	100.00		

q20_3_3 — Response unrel. question C (UQ Benford)

			Count	Percent	Valid %	Cum. %
Valid	0	no	409	6.29	48.06	48.06
	1	yes	442	6.79	51.94	100.00
	Total		851	13.08	100.00	
Missing	.a	no answer	1	0.02		
	.b	break-off	44	0.68		
	.c	filter: rndtest not 3	5609	86.23		
	Total		5654	86.92		
Total			6505	100.00		

q20_3_3_uq — Wording unrel. question C (UQ Benford)

		Count	Percent	Valid %	Cum. %
Valid	1 mother: birthday Jan-Jun (UQ Benford)	218	3.35	25.59	25.59
	2 mother: even birthmonth (UQ Benford)	206	3.17	24.18	49.77
	3 mother: birthday 1st-15th (UQ Benford)	218	3.35	25.59	75.35
	4 mother: even birthday (UQ Benford)	210	3.23	24.65	100.00
	Total	852	13.10	100.00	
Missing	.b break-off	44	0.68		
	.c filter: rndtest not 3	5609	86.23		
	Total	5653	86.90		
Total		6505	100.00		

q20_3_3_pyes — Probability yes-answer unrel. question C (UQ Benford)

		Count	Percent	Valid %	Cum. %
Valid	.4900853542509428507	210	3.23	24.65	24.65
	.4928232612579313554	218	3.35	25.59	50.23
	.4962251107141146256	424	6.52	49.77	100.00
	Total	852	13.10	100.00	
Missing	.b break-off	44	0.68		
	.c filter: rndtest not 3	5609	86.23		
	Total	5653	86.90		
Total		6505	100.00		

q20_3_4 — Response unrel. question D (UQ Benford)

			Count	Percent	Valid %	Cum. %
Valid	0	no	443	6.81	52.00	52.00
	1	yes	409	6.29	48.00	100.00
	Total		852	13.10	100.00	
Missing	.b	break-off	44	0.68		
	.c	filter: rndtest not 3	5609	86.23		
	Total		5653	86.90		
Total			6505	100.00		

q20_3_4_uq — Wording unrel. question D (UQ Benford)

		Count	Percent	Valid %	Cum. %
Valid	1 mother: birthday Jan-Jun (UQ Benford)	224	3.44	26.29	26.29
	2 mother: even birthmonth (UQ Benford)	211	3.24	24.77	51.06
	3 mother: birthday 1st-15th (UQ Benford)	206	3.17	24.18	75.23
	4 mother: even birthday (UQ Benford)	211	3.24	24.77	100.00
	Total	852	13.10	100.00	
Missing	.b break-off	44	0.68		
	.c filter: rndtest not 3	5609	86.23		
	Total	5653	86.90		
Total		6505	100.00		

q20_3_4_pyes — Probability yes-answer unrel. question D (UQ Benford)

		Count	Percent	Valid %	Cum. %
Valid	.4900853542509428507	211	3.24	24.77	24.77
	.4928232612579313554	206	3.17	24.18	48.94
	.4962251107141146256	435	6.69	51.06	100.00
	Total	852	13.10	100.00	
Missing	.b break-off	44	0.68		
	.c filter: rndtest not 3	5609	86.23		
	Total	5653	86.90		
Total		6505	100.00		

q20_4 — Response pick-a-number device (standard)

			Count	Percent	Valid %	Cum. %
Valid	1	yes	293	4.50	19.51	19.51
	2	no	126	1.94	8.39	27.90
	3	other	1083	16.65	72.10	100.00
	Total		1502	23.09	100.00	
Missing	.a	no answer	2	0.03		
	.b	break-off	44	0.68		
	.c	filter: rndtest not 4	4957	76.20		
	Total		5003	76.91		
Total			6505	100.00		

q20_5 — Response pick-a-number device (generic)

			Count	Percent	Valid %	Cum. %
Valid	1	a	264	4.06	17.31	17.31
	2	b	129	1.98	8.46	25.77
	3	c	1132	17.40	74.23	100.00
	Total		1525	23.44	100.00	
Missing	.a	no answer	4	0.06		
	.b	break-off	44	0.68		
	.c	filter: rndtest not 5	4932	75.82		
	Total		4980	76.56		
Total			6505	100.00		

q21_1 — How well do you know your mother's birthday (day and month)

			Count	Percent	Valid %	Cum. %
Valid	1	perfectly	5733	88.13	88.73	88.73
	2	unsure	592	9.10	9.16	97.90
	3	definitely not	136	2.09	2.10	100.00
	Total		6461	99.32	100.00	
Missing	.b	break-off	44	0.68		
Total			6505	100.00		

q21_2 — How well do you know the year your mother was born?

			Count	Percent	Valid %	Cum. %
Valid	1	perfectly	4534	69.70	70.23	70.23
	2	unsure	1473	22.64	22.82	93.05
	3	definitely not	449	6.90	6.95	100.00
	Total		6456	99.25	100.00	
Missing	.a	no answer	5	0.08		
	.b	break-off	44	0.68		
	Total		49	0.75		
Total			6505	100.00		

q21_3 — How well do you know your father's birthday (day and month)

			Count	Percent	Valid %	Cum. %
Valid	1	perfectly	5079	78.08	78.65	78.65
	2	unsure	906	13.93	14.03	92.68
	3	definitely not	473	7.27	7.32	100.00
	Total		6458	99.28	100.00	
Missing	.a	no answer	3	0.05		
	.b	break-off	44	0.68		
	Total		47	0.72		
Total			6505	100.00		

q21_4 — How well do you know the year your father was born?

			Count	Percent	Valid %	Cum. %
Valid	1	perfectly	3986	61.28	61.78	61.78
	2	unsure	1674	25.73	25.95	87.72
	3	definitely not	792	12.18	12.28	100.00
	Total		6452	99.19	100.00	
Missing	.a	no answer	9	0.14		
	.b	break-off	44	0.68		
	Total		53	0.81		
Total			6505	100.00		

q22txt — Do you have any remarks or suggestions concerning this questionnaire? (text)

		Count	Percent	Valid %	Cum. %
Valid	"Dice" is plural. You're talking about the roll of a single die.	1	0.02	0.04	0.04
	$2 for something people can easily lie about and can not be proven wrong seems to be rewarding unethical behavior. That is my main comment.	1	0.02	0.04	0.08
	.	1	0.02	0.04	0.11
	⋮	⋮	⋮	⋮	⋮
	yes it is nice ,it is a better survey	1	0.02	0.04	99.92
	you're right..some questions were a bit weird	1	0.02	0.04	99.96
	your survey's are the best. hope to get more survey like this in the future.	1	0.02	0.04	100.00
	Total	2642	40.61	100.00	
Missing		3863	59.39		
Total		6505	100.00		

browser1 — Browser name p1

			Count	Percent	Valid %	Cum. %
Valid	1	Chrome	3539	54.40	55.00	55.00
	2	Chrome iPad	3	0.05	0.05	55.05
	3	Chrome iPhone	3	0.05	0.05	55.10
	4	Firefox	1824	28.04	28.35	83.45
	5	MSIE	576	8.85	8.95	92.40
	6	Mozilla	1	0.02	0.02	92.42
	7	Opera	15	0.23	0.23	92.65
	8	Safari	369	5.67	5.74	98.38
	9	Safari iPad	76	1.17	1.18	99.56
	10	Safari iPhone	28	0.43	0.44	100.00
	Total		6434	98.91	100.00	
Missing	.a	unknown	71	1.09		
Total			6505	100.00		

version1 — Browser version p1

		Count	Percent	Valid %	Cum. %
Valid	10.0	386	5.93	6.00	6.00
	10.0.12	1	0.02	0.02	6.01
	10.0.2	2	0.03	0.03	6.05
	11.0	1	0.02	0.02	6.06
	11.0.696.34	2	0.03	0.03	6.09
	⋮	⋮	⋮	⋮	⋮
	8.0	81	1.25	1.26	98.06
	8.0.1	1	0.02	0.02	98.07
	8536.25	1	0.02	0.02	98.09
	9.0	108	1.66	1.68	99.77
	9.80	15	0.23	0.23	100.00
	Total	6434	98.91	100.00	
Missing		71	1.09		
Total		6505	100.00		

system1 — Operating system p1

		Count	Percent	Valid %	Cum. %
Valid	AOL 9.7	1	0.02	0.02	0.02
	Android	3	0.05	0.05	0.06
	Android 2.1-update1	1	0.02	0.02	0.08
	Android 2.2.1	1	0.02	0.02	0.09
	Android 2.3.4	3	0.05	0.05	0.14
	⋮	⋮	⋮	⋮	⋮
	en-us	3	0.05	0.05	98.26
	iPad	78	1.20	1.21	99.47
	iPhone	31	0.48	0.48	99.95
	iPod touch	1	0.02	0.02	99.97
	masking-agent	2	0.03	0.03	100.00
	Total	6434	98.91	100.00	
Missing		71	1.09		
Total		6505	100.00		

screen1 — Screen resolution p1

		Count	Percent	Valid %	Cum. %
Valid	1012x569	2	0.03	0.03	0.03
	1024x1280	1	0.02	0.02	0.05
	1024x576	4	0.06	0.06	0.11
	1024x600	58	0.89	0.90	1.01
	1024x614	5	0.08	0.08	1.09
	⋮	⋮	⋮	⋮	⋮
	922x691	1	0.02	0.02	99.92
	960x540	1	0.02	0.02	99.94
	960x768	2	0.03	0.03	99.97
	989x618	1	0.02	0.02	99.98
	990x742	1	0.02	0.02	100.00
	Total	6434	98.91	100.00	
Missing		71	1.09		
Total		6505	100.00		

flash1 — Adobe Flash version (-1 not installed) p1

		Count	Percent	Valid %	Cum. %
Valid	-1	205	3.15	3.19	3.19
	10.0.22	1	0.02	0.02	3.20
	10.0.32	1	0.02	0.02	3.22
	10.0.45	1	0.02	0.02	3.23
	10.0.45.2	1	0.02	0.02	3.25
	⋮	⋮	⋮	⋮	⋮
	11.9.900.152	227	3.49	3.53	99.86
	12.0.0	6	0.09	0.09	99.95
	6.0.21.0	1	0.02	0.02	99.97
	9.0.124.0	1	0.02	0.02	99.98
	9.1.122	1	0.02	0.02	100.00
	Total	6434	98.91	100.00	
Missing		71	1.09		
Total		6505	100.00		

java1 — Java support p1

			Count	Percent	Valid %	Cum. %
Valid	0	not installed	678	10.42	10.54	10.54
	1	installed	5756	88.49	89.46	100.00
	Total		6434	98.91	100.00	
Missing	.a	unknown	71	1.09		
Total			6505	100.00		

user1 — User agent string p1

		Count	Percent	Valid %	Cum. %
Valid	Mozilla/4.0 (compatible; MSIE 7.0; Windows NT 5.1; .NET CLR 1.1.4322; .NET CLR 2.0.50727; .NET CLR 3.0.4506.2152; .NET CLR 3.5.30729)	1	0.02	0.02	0.02
	Mozilla/4.0 (compatible; MSIE 7.0; Windows NT 6.0; GTB7.4; SLCC1; .NET CLR 2.0.50727; Media Center PC 5.0; .NET CLR 3.5.30729; .NET CLR 3.0.30618)	1	0.02	0.02	0.03
	Mozilla/4.0 (compatible; MSIE 7.0; Windows NT 6.0; GTB7.5; SLCC1; .NET CLR 2.0.50727; Media Center PC 5.0; .NET CLR 1.1.4322; .NET CLR 3.0.30618; .NET CLR 3.5.30729; OfficeLiveConnector.1.3; OfficeLivePatch.0.0; .NET4.0C)	1	0.02	0.02	0.05
	⋮	⋮	⋮	⋮	⋮
	Opera/9.80 (Windows NT 6.1; WOW64) Presto/2.12.388 Version/12.16	5	0.08	0.08	99.97
	Opera/9.80 (Windows NT 6.1; Win64; x64) Presto/2.12.388 Version/12.16	1	0.02	0.02	99.98
	Opera/9.80 (Windows NT 6.2; WOW64) Presto/2.12.388 Version/12.16	1	0.02	0.02	100.00
	Total	6434	98.91	100.00	
Missing		71	1.09		
Total		6505	100.00		

browser21 — Browser name p21

			Count	Percent	Valid %	Cum. %
Valid	1	Chrome	3558	54.70	55.17	55.17
	2	Chrome iPad	3	0.05	0.05	55.22
	3	Chrome iPhone	3	0.05	0.05	55.26
	4	Firefox	1819	27.96	28.21	83.47
	5	MSIE	577	8.87	8.95	92.42
	6	Mozilla	1	0.02	0.02	92.43
	7	Opera	15	0.23	0.23	92.67
	8	Safari	369	5.67	5.72	98.39
	9	Safari iPad	78	1.20	1.21	99.60
	10	Safari iPhone	26	0.40	0.40	100.00
	Total		6449	99.14	100.00	
Missing	.a	unknown	12	0.18		
	.b	break-off	44	0.68		
	Total		56	0.86		
Total			6505	100.00		

version21 — Browser version p21

		Count	Percent	Valid %	Cum. %
Valid	10.0	384	5.90	5.95	5.95
	10.0.12	1	0.02	0.02	5.97
	10.0.2	2	0.03	0.03	6.00
	11.0	1	0.02	0.02	6.02
	11.0.696.34	2	0.03	0.03	6.05
	⋮	⋮	⋮	⋮	⋮
	8.0	82	1.26	1.27	98.03
	8.0.1	1	0.02	0.02	98.05
	8536.25	1	0.02	0.02	98.06
	9.0	110	1.69	1.71	99.77
	9.80	15	0.23	0.23	100.00
	Total	6449	99.14	100.00	
Missing		56	0.86		
Total		6505	100.00		

system21 — Operating system p21

		Count	Percent	Valid %	Cum. %
Valid	AOL 9.7	1	0.02	0.02	0.02
	Android	3	0.05	0.05	0.06
	Android 2.2.1	1	0.02	0.02	0.08
	Android 2.3.4	3	0.05	0.05	0.12
	Android 2.3.6	3	0.05	0.05	0.17
	⋮	⋮	⋮	⋮	⋮
	en-us	3	0.05	0.05	98.26
	iPad	80	1.23	1.24	99.50
	iPhone	29	0.45	0.45	99.95
	iPod touch	1	0.02	0.02	99.97
	masking-agent	2	0.03	0.03	100.00
	Total	6449	99.14	100.00	
Missing		56	0.86		
Total		6505	100.00		

screen21 — Screen resolution p21

		Count	Percent	Valid %	Cum. %
Valid	1012x569	1	0.02	0.02	0.02
	1024x1280	1	0.02	0.02	0.03
	1024x576	4	0.06	0.06	0.09
	1024x600	59	0.91	0.91	1.01
	1024x614	5	0.08	0.08	1.09
	⋮	⋮	⋮	⋮	⋮
	960x540	2	0.03	0.03	99.92
	960x768	2	0.03	0.03	99.95
	976x549	1	0.02	0.02	99.97
	989x618	1	0.02	0.02	99.98
	990x742	1	0.02	0.02	100.00
	Total	6449	99.14	100.00	
Missing		56	0.86		
Total		6505	100.00		

flash21 — Adobe Flash version (-1 not installed) p21

		Count	Percent	Valid %	Cum. %
Valid	-1	202	3.11	3.13	3.13
	10.0.22	1	0.02	0.02	3.15
	10.0.32	1	0.02	0.02	3.16
	10.0.45	1	0.02	0.02	3.18
	10.0.45.2	1	0.02	0.02	3.19
	⋮	⋮	⋮	⋮	⋮
	11.9.900.117	218	3.35	3.38	96.34
	11.9.900.152	228	3.50	3.54	99.88
	12.0.0	6	0.09	0.09	99.97
	6.0.21.0	1	0.02	0.02	99.98
	9.0.124.0	1	0.02	0.02	100.00
	Total	6449	99.14	100.00	
Missing		56	0.86		
Total		6505	100.00		

java21 — Java support p21

			Count	Percent	Valid %	Cum. %
Valid	0	not installed	673	10.35	10.44	10.44
	1	installed	5776	88.79	89.56	100.00
	Total		6449	99.14	100.00	
Missing	.a	unknown	12	0.18		
	.b	break-off	44	0.68		
	Total		56	0.86		
Total			6505	100.00		

user21 — User agent string p21

		Count	Percent	Valid %	Cum. %
Valid	Mozilla/4.0 (compatible; MSIE 7.0; Windows NT 5.1; .NET CLR 1.1.4322; .NET CLR 2.0.50727; .NET CLR 3.0.4506.2152; .NET CLR 3.5.30729)	1	0.02	0.02	0.02
	Mozilla/4.0 (compatible; MSIE 7.0; Windows NT 6.0; GTB7.4; SLCC1; .NET CLR 2.0.50727; Media Center PC 5.0; .NET CLR 3.5.30729; .NET CLR 3.0.30618)	1	0.02	0.02	0.03
	Mozilla/4.0 (compatible; MSIE 7.0; Windows NT 6.0; GTB7.5; SLCC1; .NET CLR 2.0.50727; Media Center PC 5.0; .NET CLR 1.1.4322; .NET CLR 3.0.30618; .NET CLR 3.5.30729; OfficeLiveConnector.1.3; OfficeLivePatch.0.0; .NET4.0C)	1	0.02	0.02	0.05
	⋮	⋮	⋮	⋮	⋮
	Opera/9.80 (Windows NT 6.1; WOW64) Presto/2.12.388 Version/12.16	5	0.08	0.08	99.97
	Opera/9.80 (Windows NT 6.1; Win64; x64) Presto/2.12.388 Version/12.16	1	0.02	0.02	99.98
	Opera/9.80 (Windows NT 6.2; WOW64) Presto/2.12.388 Version/12.16	1	0.02	0.02	100.00
	Total	6449	99.14	100.00	
Missing		56	0.86		
Total		6505	100.00		

firstcl1 — Secs pageload to first click p1 (Starting page)

			Count	Percent	Valid %	Cum. %
Valid	0		6051	93.02	94.18	94.18
	.273		1	0.02	0.02	94.19
	.285		1	0.02	0.02	94.21
	.526		1	0.02	0.02	94.23
	.527		1	0.02	0.02	94.24
	⋮		⋮	⋮	⋮	⋮
	290.501		1	0.02	0.02	99.94
	308.622		1	0.02	0.02	99.95
	336.516		1	0.02	0.02	99.97
	766.692		1	0.02	0.02	99.98
	1320.071		1	0.02	0.02	100.00
	Total		6425	98.77	100.00	
Missing	.a	not recorded	74	1.14		
	.b	break-off	6	0.09		
	Total		80	1.23		
Total			6505	100.00		

lastcl1 — Secs pageload to last click p1 (excl. submit) (Starting page)

			Count	Percent	Valid %	Cum. %
Valid	0		6051	93.02	94.18	94.18
	.285		1	0.02	0.02	94.19
	.527		1	0.02	0.02	94.21
	.58		1	0.02	0.02	94.23
	.703		1	0.02	0.02	94.24
	⋮		⋮	⋮	⋮	⋮
	290.811		1	0.02	0.02	99.94
	308.622		1	0.02	0.02	99.95
	336.516		1	0.02	0.02	99.97
	771.076		1	0.02	0.02	99.98
	1325		1	0.02	0.02	100.00
	Total		6425	98.77	100.00	
Missing	.a	not recorded	74	1.14		
	.b	break-off	6	0.09		
	Total		80	1.23		
Total			6505	100.00		

submit1 — Secs pageload to submit p1 (Starting page)

			Count	Percent	Valid %	Cum. %
Valid	.094		1	0.02	0.02	0.02
	.375		1	0.02	0.02	0.03
	.39		1	0.02	0.02	0.05
	.412		1	0.02	0.02	0.06
	.441		1	0.02	0.02	0.08
	⋮		⋮	⋮	⋮	⋮
	1201.677		1	0.02	0.02	99.94
	1267.684		1	0.02	0.02	99.95
	1330.936		1	0.02	0.02	99.97
	1731.161		1	0.02	0.02	99.98
	1991.915		1	0.02	0.02	100.00
	Total		6425	98.77	100.00	
Missing	.a	not recorded	74	1.14		
	.b	break-off	6	0.09		
	Total		80	1.23		
Total			6505	100.00		

clcount1 — Click count p1 (excl. submit) (Starting page)

			Count	Percent	Valid %	Cum. %
Valid	0		6061	93.17	94.19	94.19
	1		259	3.98	4.02	98.21
	2		62	0.95	0.96	99.18
	3		16	0.25	0.25	99.43
	4		9	0.14	0.14	99.56
	⋮		⋮	⋮	⋮	⋮
	11		1	0.02	0.02	99.91
	12		1	0.02	0.02	99.92
	13		2	0.03	0.03	99.95
	14		2	0.03	0.03	99.98
	15		1	0.02	0.02	100.00
	Total		6435	98.92	100.00	
Missing	.a	not recorded	64	0.98		
	.b	break-off	6	0.09		
	Total		70	1.08		
Total			6505	100.00		

firstcl2 — Secs pageload to first click p2 (Screening question)

			Count	Percent	Valid %	Cum. %
Valid	0		7	0.11	0.11	0.11
	.062		1	0.02	0.02	0.12
	.089		1	0.02	0.02	0.14
	.106		1	0.02	0.02	0.15
	.107		1	0.02	0.02	0.17
	⋮		⋮	⋮	⋮	⋮
	688.919		1	0.02	0.02	99.94
	794.266		1	0.02	0.02	99.95
	930.682		1	0.02	0.02	99.97
	1056.972		1	0.02	0.02	99.98
	1074.391		1	0.02	0.02	100.00
	Total		6464	99.37	100.00	
Missing	.a	not recorded	31	0.48		
	.b	break-off	10	0.15		
	Total		41	0.63		
Total			6505	100.00		

lastcl2 — Secs pageload to last click p2 (excl. submit) (Screening question)

			Count	Percent	Valid %	Cum. %
Valid	0		7	0.11	0.11	0.11
	.835		1	0.02	0.02	0.12
	1.134		1	0.02	0.02	0.14
	1.266		1	0.02	0.02	0.15
	1.287		1	0.02	0.02	0.17
	⋮		⋮	⋮	⋮	⋮
	719.214		1	0.02	0.02	99.94
	795.966		1	0.02	0.02	99.95
	932.667		1	0.02	0.02	99.97
	1062.37		1	0.02	0.02	99.98
	1075.903		1	0.02	0.02	100.00
	Total		6464	99.37	100.00	
Missing	.a	not recorded	31	0.48		
	.b	break-off	10	0.15		
	Total		41	0.63		
Total			6505	100.00		

submit2 — Secs pageload to submit p2 (Screening question)

			Count	Percent	Valid %	Cum. %
Valid	.702		1	0.02	0.02	0.02
	2.17		1	0.02	0.02	0.03
	2.19		1	0.02	0.02	0.05
	2.257		1	0.02	0.02	0.06
	2.543		1	0.02	0.02	0.08
	⋮		⋮	⋮	⋮	⋮
	731.46		1	0.02	0.02	99.94
	802.55		1	0.02	0.02	99.95
	936.071		1	0.02	0.02	99.97
	1067.299		1	0.02	0.02	99.98
	1080.593		1	0.02	0.02	100.00
	Total		6464	99.37	100.00	
Missing	.a	not recorded	31	0.48		
	.b	break-off	10	0.15		
	Total		41	0.63		
Total			6505	100.00		

clcount2 — Click count p2 (excl. submit) (Screening question)

			Count	Percent	Valid %	Cum. %
Valid	0		15	0.23	0.23	0.23
	1		635	9.76	9.81	10.04
	2		4297	66.06	66.39	76.44
	3		844	12.97	13.04	89.48
	4		317	4.87	4.90	94.38
	⋮		⋮	⋮	⋮	⋮
	18		5	0.08	0.08	99.94
	20		1	0.02	0.02	99.95
	23		1	0.02	0.02	99.97
	33		1	0.02	0.02	99.98
	64		1	0.02	0.02	100.00
	Total		6472	99.49	100.00	
Missing	.a	not recorded	23	0.35		
	.b	break-off	10	0.15		
	Total		33	0.51		
Total			6505	100.00		

firstcl3 — Secs pageload to first click p3 (Personal background I)

			Count	Percent	Valid %	Cum. %
Valid	0		13	0.20	0.20	0.20
	.083		1	0.02	0.02	0.22
	.652		1	0.02	0.02	0.23
	.775		1	0.02	0.02	0.25
	.807		1	0.02	0.02	0.26
	⋮		⋮	⋮	⋮	⋮
	482.961		1	0.02	0.02	99.94
	616.986		1	0.02	0.02	99.95
	728.537		1	0.02	0.02	99.97
	735.076		1	0.02	0.02	99.98
	907.44		1	0.02	0.02	100.00
	Total		6463	99.35	100.00	
Missing	.a	not recorded	28	0.43		
	.b	break-off	14	0.22		
	Total		42	0.65		
Total			6505	100.00		

lastcl3 — Secs pageload to last click p3 (excl. submit) (Personal background I)

			Count	Percent	Valid %	Cum. %
Valid	0		13	0.20	0.20	0.20
	1.843		1	0.02	0.02	0.22
	2.79		1	0.02	0.02	0.23
	2.791		1	0.02	0.02	0.25
	2.808		1	0.02	0.02	0.26
	⋮		⋮	⋮	⋮	⋮
	484.115		1	0.02	0.02	99.94
	619.22		1	0.02	0.02	99.95
	732.351		1	0.02	0.02	99.97
	738.683		1	0.02	0.02	99.98
	911.449		1	0.02	0.02	100.00
	Total		6463	99.35	100.00	
Missing	.a	not recorded	28	0.43		
	.b	break-off	14	0.22		
	Total		42	0.65		
Total			6505	100.00		

submit3 — Secs pageload to submit p3 (Personal background I)

			Count	Percent	Valid %	Cum. %
Valid	3.125		1	0.02	0.02	0.02
	3.619		1	0.02	0.02	0.03
	3.916		1	0.02	0.02	0.05
	3.921		1	0.02	0.02	0.06
	3.984		1	0.02	0.02	0.08
	⋮		⋮	⋮	⋮	⋮
	484.989		1	0.02	0.02	99.94
	622.156		1	0.02	0.02	99.95
	736.942		1	0.02	0.02	99.97
	751.6		1	0.02	0.02	99.98
	912.011		1	0.02	0.02	100.00
	Total		6463	99.35	100.00	
Missing	.a	not recorded	28	0.43		
	.b	break-off	14	0.22		
	Total		42	0.65		
Total			6505	100.00		

clcount3 — Click count p3 (excl. submit) (Personal background I)

			Count	Percent	Valid %	Cum. %
Valid	0		21	0.32	0.32	0.32
	1		6	0.09	0.09	0.42
	2		13	0.20	0.20	0.62
	3		4575	70.33	70.70	71.32
	4		1269	19.51	19.61	90.93
	⋮		⋮	⋮	⋮	⋮
	16		1	0.02	0.02	99.94
	17		1	0.02	0.02	99.95
	19		1	0.02	0.02	99.97
	24		1	0.02	0.02	99.98
	35		1	0.02	0.02	100.00
	Total		6471	99.48	100.00	
Missing	.a	not recorded	20	0.31		
	.b	break-off	14	0.22		
	Total		34	0.52		
Total			6505	100.00		

firstcl4 — Secs pageload to first click p4 (Intro dice game)

			Count	Percent	Valid %	Cum. %
Valid	0		6015	92.47	93.08	93.08
	.398		1	0.02	0.02	93.10
	.788		1	0.02	0.02	93.11
	.874		1	0.02	0.02	93.13
	.905		2	0.03	0.03	93.16
	⋮		⋮	⋮	⋮	⋮
	107.243		1	0.02	0.02	99.94
	123.055		1	0.02	0.02	99.95
	162.937		1	0.02	0.02	99.97
	172.72		1	0.02	0.02	99.98
	271.293		1	0.02	0.02	100.00
	Total		6462	99.34	100.00	
Missing	.a	not recorded	26	0.40		
	.b	break-off	17	0.26		
	Total		43	0.66		
Total			6505	100.00		

lastcl4 — Secs pageload to last click p4 (excl. submit) (Intro dice game)

			Count	Percent	Valid %	Cum. %
Valid	0		6015	92.47	93.08	93.08
	.398		1	0.02	0.02	93.10
	.788		1	0.02	0.02	93.11
	.905		1	0.02	0.02	93.13
	1.211		1	0.02	0.02	93.14
	⋮		⋮	⋮	⋮	⋮
	107.243		1	0.02	0.02	99.94
	123.055		1	0.02	0.02	99.95
	162.937		1	0.02	0.02	99.97
	172.72		1	0.02	0.02	99.98
	271.293		1	0.02	0.02	100.00
	Total		6462	99.34	100.00	
Missing	.a	not recorded	26	0.40		
	.b	break-off	17	0.26		
	Total		43	0.66		
Total			6505	100.00		

submit4 — Secs pageload to submit p4 (Intro dice game)

			Count	Percent	Valid %	Cum. %
Valid	1.23		1	0.02	0.02	0.02
	1.34		1	0.02	0.02	0.03
	1.345		1	0.02	0.02	0.05
	1.437		1	0.02	0.02	0.06
	1.485		1	0.02	0.02	0.08
	⋮		⋮	⋮	⋮	⋮
	391.358		1	0.02	0.02	99.94
	462.254		1	0.02	0.02	99.95
	498.54		1	0.02	0.02	99.97
	504.914		1	0.02	0.02	99.98
	1845.574		1	0.02	0.02	100.00
	Total		6462	99.34	100.00	
Missing	.a	not recorded	26	0.40		
	.b	break-off	17	0.26		
	Total		43	0.66		
Total			6505	100.00		

clcount4 — Click count p4 (excl. submit) (Intro dice game)

			Count	Percent	Valid %	Cum. %
Valid	0		6023	92.59	93.09	93.09
	1		185	2.84	2.86	95.95
	2		95	1.46	1.47	97.42
	3		44	0.68	0.68	98.10
	4		38	0.58	0.59	98.69
	⋮		⋮	⋮	⋮	⋮
	28		1	0.02	0.02	99.94
	31		1	0.02	0.02	99.95
	34		1	0.02	0.02	99.97
	37		1	0.02	0.02	99.98
	67		1	0.02	0.02	100.00
	Total		6470	99.46	100.00	
Missing	.a	not recorded	18	0.28		
	.b	break-off	17	0.26		
	Total		35	0.54		
Total			6505	100.00		

firstcl5 — Secs pageload to first click p5 (Prediction dice game)

			Count	Percent	Valid %	Cum. %
Valid	0		2713	41.71	83.92	83.92
	.801		1	0.02	0.03	83.95
	1.201		1	0.02	0.03	83.98
	1.26		1	0.02	0.03	84.01
	1.263		1	0.02	0.03	84.04
	⋮		⋮	⋮	⋮	⋮
	30.069		1	0.02	0.03	99.88
	38.055		1	0.02	0.03	99.91
	42.713		1	0.02	0.03	99.94
	68.183		1	0.02	0.03	99.97
	125.92		1	0.02	0.03	100.00
	Total		3233	49.70	100.00	
Missing	.a	not recorded	12	0.18		
	.b	break-off	17	0.26		
	.c	filter: dicegame roll-a-six	3243	49.85		
	Total		3272	50.30		
Total			6505	100.00		

lastcl5 — Secs pageload to last click p5 (excl. submit) (Prediction dice game)

			Count	Percent	Valid %	Cum. %
Valid	0		2713	41.71	83.92	83.92
	1.201		1	0.02	0.03	83.95
	1.26		1	0.02	0.03	83.98
	1.263		1	0.02	0.03	84.01
	1.31		1	0.02	0.03	84.04
	⋮		⋮	⋮	⋮	⋮
	35.381		1	0.02	0.03	99.88
	38.055		1	0.02	0.03	99.91
	42.713		1	0.02	0.03	99.94
	68.183		1	0.02	0.03	99.97
	125.92		1	0.02	0.03	100.00
	Total		3233	49.70	100.00	
Missing	.a	not recorded	12	0.18		
	.b	break-off	17	0.26		
	.c	filter: dicegame roll-a-six	3243	49.85		
	Total		3272	50.30		
Total			6505	100.00		

submit5 — Secs pageload to submit p5 (Prediction dice game)

			Count	Percent	Valid %	Cum. %
Valid	.948		1	0.02	0.03	0.03
	1.002		1	0.02	0.03	0.06
	1.085		1	0.02	0.03	0.09
	1.092		1	0.02	0.03	0.12
	1.103		1	0.02	0.03	0.15
	⋮		⋮	⋮	⋮	⋮
	115.438		1	0.02	0.03	99.88
	126.331		1	0.02	0.03	99.91
	145.167		1	0.02	0.03	99.94
	167.128		1	0.02	0.03	99.97
	248.03		1	0.02	0.03	100.00
	Total		3233	49.70	100.00	
Missing	.a	not recorded	12	0.18		
	.b	break-off	17	0.26		
	.c	filter: dicegame roll-a-six	3243	49.85		
	Total		3272	50.30		
Total			6505	100.00		

clcount5 — Click count p5 (excl. submit) (Prediction dice game)

			Count	Percent	Valid %	Cum. %
Valid	0		2716	41.75	83.93	83.93
	1		288	4.43	8.90	92.83
	2		117	1.80	3.62	96.45
	3		47	0.72	1.45	97.90
	4		26	0.40	0.80	98.70
	⋮		⋮	⋮	⋮	⋮
	8		2	0.03	0.06	99.81
	9		1	0.02	0.03	99.85
	11		3	0.05	0.09	99.94
	14		1	0.02	0.03	99.97
	15		1	0.02	0.03	100.00
	Total		3236	49.75	100.00	
Missing	.a	not recorded	9	0.14		
	.b	break-off	17	0.26		
	.c	filter: dicegame roll-a-six	3243	49.85		
	Total		3269	50.25		
Total			6505	100.00		

firstcl6 — Secs pageload to first click p6 (Dice rolling)

			Count	Percent	Valid %	Cum. %
Valid	0		15	0.23	0.23	0.23
	.047		1	0.02	0.02	0.25
	.055		1	0.02	0.02	0.26
	.063		1	0.02	0.02	0.28
	.073		1	0.02	0.02	0.29
	⋮		⋮	⋮	⋮	⋮
	124.652		1	0.02	0.02	99.94
	130.517		1	0.02	0.02	99.95
	191.954		1	0.02	0.02	99.97
	194.927		1	0.02	0.02	99.98
	365.725		1	0.02	0.02	100.00
	Total		6451	99.17	100.00	
Missing	.a	not recorded	33	0.51		
	.b	break-off	21	0.32		
	Total		54	0.83		
Total			6505	100.00		

lastcl6 — Secs pageload to last click p6 (excl. submit) (Dice rolling)

			Count	Percent	Valid %	Cum. %
Valid	0		15	0.23	0.23	0.23
	.944		1	0.02	0.02	0.25
	1.444		1	0.02	0.02	0.26
	1.545		1	0.02	0.02	0.28
	1.576		1	0.02	0.02	0.29
	⋮		⋮	⋮	⋮	⋮
	194.872		1	0.02	0.02	99.94
	200.924		1	0.02	0.02	99.95
	238.492		1	0.02	0.02	99.97
	301.564		1	0.02	0.02	99.98
	426.091		1	0.02	0.02	100.00
	Total		6451	99.17	100.00	
Missing	.a	not recorded	33	0.51		
	.b	break-off	21	0.32		
	Total		54	0.83		
Total			6505	100.00		

submit6 — Secs pageload to submit p6 (Dice rolling)

			Count	Percent	Valid %	Cum. %
Valid	1.043		1	0.02	0.02	0.02
	1.123		1	0.02	0.02	0.03
	1.25		1	0.02	0.02	0.05
	1.42		1	0.02	0.02	0.06
	1.592		1	0.02	0.02	0.08
	⋮		⋮	⋮	⋮	⋮
	180.275		1	0.02	0.02	99.94
	195.901		1	0.02	0.02	99.95
	201.63		1	0.02	0.02	99.97
	256.136		1	0.02	0.02	99.98
	304.59		1	0.02	0.02	100.00
	Total		6451	99.17	100.00	
Missing	.a	not recorded	33	0.51		
	.b	break-off	21	0.32		
	Total		54	0.83		
Total			6505	100.00		

clcount6 — Click count p6 (excl. submit) (Dice rolling)

			Count	Percent	Valid %	Cum. %
Valid	0		23	0.35	0.36	0.36
	1		28	0.43	0.43	0.79
	2		5503	84.60	85.20	85.99
	3		494	7.59	7.65	93.64
	4		151	2.32	2.34	95.97
	⋮		⋮	⋮	⋮	⋮
	19		2	0.03	0.03	99.88
	21		5	0.08	0.08	99.95
	22		1	0.02	0.02	99.97
	23		1	0.02	0.02	99.98
	26		1	0.02	0.02	100.00
	Total		6459	99.29	100.00	
Missing	.a	not recorded	25	0.38		
	.b	break-off	21	0.32		
	Total		46	0.71		
Total			6505	100.00		

firstcl7 — Secs pageload to first click p7 (Satisfaction)

			Count	Percent	Valid %	Cum. %
Valid	0		9	0.14	0.14	0.14
	.031		1	0.02	0.02	0.15
	.268		1	0.02	0.02	0.17
	.281		1	0.02	0.02	0.19
	.39		1	0.02	0.02	0.20
	⋮		⋮	⋮	⋮	⋮
	102.328		1	0.02	0.02	99.94
	104.963		1	0.02	0.02	99.95
	126.392		1	0.02	0.02	99.97
	218.071		1	0.02	0.02	99.98
	279.795		1	0.02	0.02	100.00
	Total		6462	99.34	100.00	
Missing	.a	not recorded	21	0.32		
	.b	break-off	22	0.34		
	Total		43	0.66		
Total			6505	100.00		

lastcl7 — Secs pageload to last click p7 (excl. submit) (Satisfaction)

			Count	Percent	Valid %	Cum. %
Valid	0		9	0.14	0.14	0.14
	1.482		1	0.02	0.02	0.15
	1.62		1	0.02	0.02	0.17
	1.813		1	0.02	0.02	0.19
	1.828		1	0.02	0.02	0.20
	⋮		⋮	⋮	⋮	⋮
	106.195		1	0.02	0.02	99.94
	127.999		1	0.02	0.02	99.95
	218.961		1	0.02	0.02	99.97
	282.806		1	0.02	0.02	99.98
	794.136		1	0.02	0.02	100.00
	Total		6462	99.34	100.00	
Missing	.a	not recorded	21	0.32		
	.b	break-off	22	0.34		
	Total		43	0.66		
Total			6505	100.00		

submit7 — Secs pageload to submit p7 (Satisfaction)

			Count	Percent	Valid %	Cum. %
Valid	2.06		1	0.02	0.02	0.02
	2.424		1	0.02	0.02	0.03
	2.578		1	0.02	0.02	0.05
	2.581		1	0.02	0.02	0.06
	2.591		1	0.02	0.02	0.08
	⋮		⋮	⋮	⋮	⋮
	106.726		1	0.02	0.02	99.94
	128.904		1	0.02	0.02	99.95
	219.772		1	0.02	0.02	99.97
	283.758		1	0.02	0.02	99.98
	795.525		1	0.02	0.02	100.00
	Total		6462	99.34	100.00	
Missing	.a	not recorded	21	0.32		
	.b	break-off	22	0.34		
	Total		43	0.66		
Total			6505	100.00		

clcount7 — Click count p7 (excl. submit) (Satisfaction)

			Count	Percent	Valid %	Cum. %
Valid	0		16	0.25	0.25	0.25
	1		1	0.02	0.02	0.26
	2		4668	71.76	72.16	72.42
	3		1175	18.06	18.16	90.59
	4		370	5.69	5.72	96.31
	⋮		⋮	⋮	⋮	⋮
	16		1	0.02	0.02	99.94
	18		1	0.02	0.02	99.95
	20		1	0.02	0.02	99.97
	22		1	0.02	0.02	99.98
	42		1	0.02	0.02	100.00
	Total		6469	99.45	100.00	
Missing	.a	not recorded	14	0.22		
	.b	break-off	22	0.34		
	Total		36	0.55		
Total			6505	100.00		

firstcl8 — Secs pageload to first click p8 (Big Five)

			Count	Percent	Valid %	Cum. %
Valid	0		7	0.11	0.11	0.11
	.062		1	0.02	0.02	0.12
	.078		1	0.02	0.02	0.14
	.089		1	0.02	0.02	0.15
	.655		1	0.02	0.02	0.17
	⋮		⋮	⋮	⋮	⋮
	229.506		1	0.02	0.02	99.94
	300.671		1	0.02	0.02	99.95
	384.916		1	0.02	0.02	99.97
	483.839		1	0.02	0.02	99.98
	498.549		1	0.02	0.02	100.00
	Total		6461	99.32	100.00	
Missing	.a	not recorded	19	0.29		
	.b	break-off	25	0.38		
	Total		44	0.68		
Total			6505	100.00		

lastcl8 — Secs pageload to last click p8 (excl. submit) (Big Five)

			Count	Percent	Valid %	Cum. %
Valid	0		7	0.11	0.11	0.11
	4.294		1	0.02	0.02	0.12
	4.321		1	0.02	0.02	0.14
	4.353		1	0.02	0.02	0.15
	4.495		1	0.02	0.02	0.17
	⋮		⋮	⋮	⋮	⋮
	447.285		1	0.02	0.02	99.94
	504.837		1	0.02	0.02	99.95
	526.841		1	0.02	0.02	99.97
	607.567		1	0.02	0.02	99.98
	1010.794		1	0.02	0.02	100.00
	Total		6461	99.32	100.00	
Missing	.a	not recorded	19	0.29		
	.b	break-off	25	0.38		
	Total		44	0.68		
Total			6505	100.00		

submit8 — Secs pageload to submit p8 (Big Five)

			Count	Percent	Valid %	Cum. %
Valid	4.898		1	0.02	0.02	0.02
	5.089		1	0.02	0.02	0.03
	5.192		1	0.02	0.02	0.05
	5.502		1	0.02	0.02	0.06
	5.662		1	0.02	0.02	0.08
	⋮		⋮	⋮	⋮	⋮
	447.701		1	0.02	0.02	99.94
	505.445		1	0.02	0.02	99.95
	527.544		1	0.02	0.02	99.97
	608.044		1	0.02	0.02	99.98
	1018.407		1	0.02	0.02	100.00
	Total		6461	99.32	100.00	
Missing	.a	not recorded	19	0.29		
	.b	break-off	25	0.38		
	Total		44	0.68		
Total			6505	100.00		

clcount8 — Click count p8 (excl. submit) (Big Five)

			Count	Percent	Valid %	Cum. %
Valid	0		14	0.22	0.22	0.22
	3		1	0.02	0.02	0.23
	4		1	0.02	0.02	0.25
	7		1	0.02	0.02	0.26
	8		3	0.05	0.05	0.31
	⋮		⋮	⋮	⋮	⋮
	28		1	0.02	0.02	99.92
	29		2	0.03	0.03	99.95
	36		1	0.02	0.02	99.97
	45		1	0.02	0.02	99.98
	46		1	0.02	0.02	100.00
	Total		6468	99.43	100.00	
Missing	.a	not recorded	12	0.18		
	.b	break-off	25	0.38		
	Total		37	0.57		
Total			6505	100.00		

firstcl9 — Secs pageload to first click p9 (Personal background II)

			Count	Percent	Valid %	Cum. %
Valid	0		11	0.17	0.17	0.17
	.11		1	0.02	0.02	0.19
	.116		1	0.02	0.02	0.20
	.172		1	0.02	0.02	0.22
	.438		1	0.02	0.02	0.23
	⋮		⋮	⋮	⋮	⋮
	208.63		1	0.02	0.02	99.94
	290.937		1	0.02	0.02	99.95
	326.459		1	0.02	0.02	99.97
	350.586		1	0.02	0.02	99.98
	583.048		1	0.02	0.02	100.00
	Total		6458	99.28	100.00	
Missing	.a	not recorded	19	0.29		
	.b	break-off	28	0.43		
	Total		47	0.72		
Total			6505	100.00		

lastcl9 — Secs pageload to last click p9 (excl. submit) (Personal background II)

			Count	Percent	Valid %	Cum. %
Valid	0		11	0.17	0.17	0.17
	.172		1	0.02	0.02	0.19
	1.652		1	0.02	0.02	0.20
	1.747		1	0.02	0.02	0.22
	1.921		1	0.02	0.02	0.23
	⋮		⋮	⋮	⋮	⋮
	331.184		1	0.02	0.02	99.94
	354.486		1	0.02	0.02	99.95
	444.003		1	0.02	0.02	99.97
	482.849		1	0.02	0.02	99.98
	588.83		1	0.02	0.02	100.00
	Total		6458	99.28	100.00	
Missing	.a	not recorded	19	0.29		
	.b	break-off	28	0.43		
	Total		47	0.72		
Total			6505	100.00		

submit9 — Secs pageload to submit p9 (Personal background II)

			Count	Percent	Valid %	Cum. %
Valid	2.531		1	0.02	0.02	0.02
	3.662		1	0.02	0.02	0.03
	3.854		1	0.02	0.02	0.05
	3.978		1	0.02	0.02	0.06
	4.1		1	0.02	0.02	0.08
	⋮		⋮	⋮	⋮	⋮
	355.406		1	0.02	0.02	99.94
	445.844		1	0.02	0.02	99.95
	483.73		1	0.02	0.02	99.97
	505.727		1	0.02	0.02	99.98
	589.393		1	0.02	0.02	100.00
	Total		6458	99.28	100.00	
Missing	.a	not recorded	19	0.29		
	.b	break-off	28	0.43		
	Total		47	0.72		
Total			6505	100.00		

clcount9 — Click count p9 (excl. submit) (Personal background II)

			Count	Percent	Valid %	Cum. %
Valid	0		18	0.28	0.28	0.28
	1		4	0.06	0.06	0.34
	2		78	1.20	1.21	1.55
	3		4738	72.84	73.29	74.83
	4		1153	17.72	17.83	92.67
	⋮		⋮	⋮	⋮	⋮
	11		1	0.02	0.02	99.94
	12		1	0.02	0.02	99.95
	13		1	0.02	0.02	99.97
	14		1	0.02	0.02	99.98
	17		1	0.02	0.02	100.00
	Total		6465	99.39	100.00	
Missing	.a	not recorded	12	0.18		
	.b	break-off	28	0.43		
	Total		40	0.61		
Total			6505	100.00		

firstcl10 — Secs pageload to first click p10 (MTurk and employment)

			Count	Percent	Valid %	Cum. %
Valid	0		10	0.15	0.15	0.15
	.125		1	0.02	0.02	0.17
	.219		1	0.02	0.02	0.19
	.343		1	0.02	0.02	0.20
	.453		1	0.02	0.02	0.22
	⋮		⋮	⋮	⋮	⋮
	245.738		1	0.02	0.02	99.94
	250.157		1	0.02	0.02	99.95
	254.808		1	0.02	0.02	99.97
	316.756		1	0.02	0.02	99.98
	376.448		1	0.02	0.02	100.00
	Total		6458	99.28	100.00	
Missing	.a	not recorded	19	0.29		
	.b	break-off	28	0.43		
	Total		47	0.72		
Total			6505	100.00		

lastcl10 — Secs pageload to last click p10 (excl. submit) (MTurk and employment)

			Count	Percent	Valid %	Cum. %
Valid	0		10	0.15	0.15	0.15
	.624		1	0.02	0.02	0.17
	2.028		1	0.02	0.02	0.19
	2.062		1	0.02	0.02	0.20
	2.605		1	0.02	0.02	0.22
	⋮		⋮	⋮	⋮	⋮
	322.101		1	0.02	0.02	99.94
	367.833		1	0.02	0.02	99.95
	390.712		1	0.02	0.02	99.97
	471.727		1	0.02	0.02	99.98
	507.838		1	0.02	0.02	100.00
	Total		6458	99.28	100.00	
Missing	.a	not recorded	19	0.29		
	.b	break-off	28	0.43		
	Total		47	0.72		
Total			6505	100.00		

submit10 — Secs pageload to submit p10 (MTurk and employment)

			Count	Percent	Valid %	Cum. %
Valid	1.388		1	0.02	0.02	0.02
	5.226		1	0.02	0.02	0.03
	5.772		1	0.02	0.02	0.05
	6.069		1	0.02	0.02	0.06
	6.085		1	0.02	0.02	0.08
	⋮		⋮	⋮	⋮	⋮
	324.546		1	0.02	0.02	99.94
	374.906		1	0.02	0.02	99.95
	392.868		1	0.02	0.02	99.97
	475.024		1	0.02	0.02	99.98
	517.685		1	0.02	0.02	100.00
	Total		6458	99.28	100.00	
Missing	.a	not recorded	19	0.29		
	.b	break-off	28	0.43		
	Total		47	0.72		
Total			6505	100.00		

clcount10 — Click count p10 (excl. submit) (MTurk and employment)

			Count	Percent	Valid %	Cum. %
Valid	0		17	0.26	0.26	0.26
	1		9	0.14	0.14	0.40
	2		32	0.49	0.49	0.90
	3		3804	58.48	58.84	59.74
	4		1422	21.86	22.00	81.73
	⋮		⋮	⋮	⋮	⋮
	15		3	0.05	0.05	99.94
	16		1	0.02	0.02	99.95
	17		1	0.02	0.02	99.97
	21		1	0.02	0.02	99.98
	22		1	0.02	0.02	100.00
	Total		6465	99.39	100.00	
Missing	.a	not recorded	12	0.18		
	.b	break-off	28	0.43		
	Total		40	0.61		
Total			6505	100.00		

firstcl11 — Secs pageload to first click p11 (Intro sensitive questions)

			Count	Percent	Valid %	Cum. %
Valid	0		6157	94.65	95.35	95.35
	.078		1	0.02	0.02	95.37
	.529		1	0.02	0.02	95.38
	.546		1	0.02	0.02	95.40
	.636		1	0.02	0.02	95.42
	⋮		⋮	⋮	⋮	⋮
	34.963		1	0.02	0.02	99.94
	40.154		1	0.02	0.02	99.95
	68.024		1	0.02	0.02	99.97
	92.781		1	0.02	0.02	99.98
	242.626		1	0.02	0.02	100.00
	Total		6457	99.26	100.00	
Missing	.a	not recorded	20	0.31		
	.b	break-off	28	0.43		
	Total		48	0.74		
Total			6505	100.00		

lastcl11 — Secs pageload to last click p11 (excl. submit) (Intro sensitive questions)

			Count	Percent	Valid %	Cum. %
Valid	0		6157	94.65	95.35	95.35
	.078		1	0.02	0.02	95.37
	.529		1	0.02	0.02	95.38
	.546		1	0.02	0.02	95.40
	.636		1	0.02	0.02	95.42
	⋮		⋮	⋮	⋮	⋮
	40.154		1	0.02	0.02	99.94
	68.757		1	0.02	0.02	99.95
	68.855		1	0.02	0.02	99.97
	94.597		1	0.02	0.02	99.98
	242.626		1	0.02	0.02	100.00
	Total		6457	99.26	100.00	
Missing	.a	not recorded	20	0.31		
	.b	break-off	28	0.43		
	Total		48	0.74		
Total			6505	100.00		

submit11 — Secs pageload to submit p11 (Intro sensitive questions)

			Count	Percent	Valid %	Cum. %
Valid	.613		1	0.02	0.02	0.02
	1.087		1	0.02	0.02	0.03
	1.154		1	0.02	0.02	0.05
	1.281		1	0.02	0.02	0.06
	1.342		1	0.02	0.02	0.08
	⋮		⋮	⋮	⋮	⋮
	299.039		1	0.02	0.02	99.94
	306.839		1	0.02	0.02	99.95
	418.376		1	0.02	0.02	99.97
	455.895		1	0.02	0.02	99.98
	479.993		1	0.02	0.02	100.00
	Total		6457	99.26	100.00	
Missing	.a	not recorded	20	0.31		
	.b	break-off	28	0.43		
	Total		48	0.74		
Total			6505	100.00		

clcount11 — Click count p11 (excl. submit) (Intro sensitive questions)

			Count	Percent	Valid %	Cum. %
Valid	0		6164	94.76	95.36	95.36
	1		195	3.00	3.02	98.38
	2		54	0.83	0.84	99.21
	3		9	0.14	0.14	99.35
	4		16	0.25	0.25	99.60
	⋮		⋮	⋮	⋮	⋮
	9		1	0.02	0.02	99.91
	10		1	0.02	0.02	99.92
	11		1	0.02	0.02	99.94
	12		3	0.05	0.05	99.98
	17		1	0.02	0.02	100.00
	Total		6464	99.37	100.00	
Missing	.a	not recorded	13	0.20		
	.b	break-off	28	0.43		
	Total		41	0.63		
Total			6505	100.00		

firstcl12 — Secs pageload to first click p12 (Explanation special technique)

			Count	Percent	Valid %	Cum. %
Valid	0		5044	77.54	89.32	89.32
	.453		1	0.02	0.02	89.34
	.531		1	0.02	0.02	89.36
	.796		1	0.02	0.02	89.37
	.82		1	0.02	0.02	89.39
	⋮		⋮	⋮	⋮	⋮
	160.474		1	0.02	0.02	99.93
	212.581		1	0.02	0.02	99.95
	247.213		1	0.02	0.02	99.96
	255.417		1	0.02	0.02	99.98
	408.523		1	0.02	0.02	100.00
	Total		5647	86.81	100.00	
Missing	.a	not recorded	16	0.25		
	.b	break-off	32	0.49		
	.c	filter: DQ	810	12.45		
	Total		858	13.19		
Total			6505	100.00		

lastcl12 — Secs pageload to last click p12 (excl. submit) (Explanation special technique)

			Count	Percent	Valid %	Cum. %
Valid	0		5044	77.54	89.32	89.32
	.531		1	0.02	0.02	89.34
	.838		1	0.02	0.02	89.36
	.889		1	0.02	0.02	89.37
	1.161		1	0.02	0.02	89.39
	⋮		⋮	⋮	⋮	⋮
	265.954		1	0.02	0.02	99.93
	286.932		1	0.02	0.02	99.95
	399.904		1	0.02	0.02	99.96
	418.575		1	0.02	0.02	99.98
	1804.963		1	0.02	0.02	100.00
	Total		5647	86.81	100.00	
Missing	.a	not recorded	16	0.25		
	.b	break-off	32	0.49		
	.c	filter: DQ	810	12.45		
	Total		858	13.19		
Total			6505	100.00		

submit12 — Secs pageload to submit p12 (Explanation special technique)

			Count	Percent	Valid %	Cum. %
Valid	.125		1	0.02	0.02	0.02
	1.043		1	0.02	0.02	0.04
	1.057		1	0.02	0.02	0.05
	1.148		1	0.02	0.02	0.07
	1.288		1	0.02	0.02	0.09
	⋮		⋮	⋮	⋮	⋮
	915.008		1	0.02	0.02	99.93
	950.538		1	0.02	0.02	99.95
	1059.781		1	0.02	0.02	99.96
	1170.272		1	0.02	0.02	99.98
	1874.881		1	0.02	0.02	100.00
	Total		5647	86.81	100.00	
Missing	.a	not recorded	16	0.25		
	.b	break-off	32	0.49		
	.c	filter: DQ	810	12.45		
	Total		858	13.19		
Total			6505	100.00		

clcount12 — Click count p12 (excl. submit) (Explanation special technique)

			Count	Percent	Valid %	Cum. %
Valid	0		5050	77.63	89.33	89.33
	1		225	3.46	3.98	93.31
	2		127	1.95	2.25	95.56
	3		69	1.06	1.22	96.78
	4		33	0.51	0.58	97.36
	⋮		⋮	⋮	⋮	⋮
	46		2	0.03	0.04	99.93
	48		1	0.02	0.02	99.95
	52		1	0.02	0.02	99.96
	74		1	0.02	0.02	99.98
	149		1	0.02	0.02	100.00
	Total		5653	86.90	100.00	
Missing	.a	not recorded	10	0.15		
	.b	break-off	32	0.49		
	.c	filter: DQ	810	12.45		
	Total		852	13.10		
Total			6505	100.00		

firstcl13 — Secs pageload to first click p13 (Benford procedure)

			Count	Percent	Valid %	Cum. %
Valid	0		1485	22.83	91.95	91.95
	.058		1	0.02	0.06	92.01
	1.036		1	0.02	0.06	92.07
	1.268		1	0.02	0.06	92.14
	1.364		1	0.02	0.06	92.20
	⋮		⋮	⋮	⋮	⋮
	47.435		1	0.02	0.06	99.75
	48.297		1	0.02	0.06	99.81
	51.453		1	0.02	0.06	99.88
	78.676		1	0.02	0.06	99.94
	78.948		1	0.02	0.06	100.00
	Total		1615	24.83	100.00	
Missing	.a	not recorded	3	0.05		
	.b	break-off	32	0.49		
	.c	filter: senstec not 3	4855	74.63		
	Total		4890	75.17		
Total			6505	100.00		

lastcl13 — Secs pageload to last click p13 (excl. submit) (Benford procedure)

			Count	Percent	Valid %	Cum. %
Valid	0		1485	22.83	91.95	91.95
	.058		1	0.02	0.06	92.01
	1.036		1	0.02	0.06	92.07
	1.364		1	0.02	0.06	92.14
	1.955		1	0.02	0.06	92.20
	⋮		⋮	⋮	⋮	⋮
	48.297		1	0.02	0.06	99.75
	52.312		1	0.02	0.06	99.81
	78.676		1	0.02	0.06	99.88
	78.948		1	0.02	0.06	99.94
	81.652		1	0.02	0.06	100.00
	Total		1615	24.83	100.00	
Missing	.a	not recorded	3	0.05		
	.b	break-off	32	0.49		
	.c	filter: senstec not 3	4855	74.63		
	Total		4890	75.17		
Total			6505	100.00		

submit13 — Secs pageload to submit p13 (Benford procedure)

			Count	Percent	Valid %	Cum. %
Valid	.946		1	0.02	0.06	0.06
	.996		1	0.02	0.06	0.12
	1.466		1	0.02	0.06	0.19
	1.699		1	0.02	0.06	0.25
	1.881		1	0.02	0.06	0.31
	⋮		⋮	⋮	⋮	⋮
	195.364		1	0.02	0.06	99.75
	203.95		1	0.02	0.06	99.81
	228.234		1	0.02	0.06	99.88
	382.941		1	0.02	0.06	99.94
	579.468		1	0.02	0.06	100.00
	Total		1615	24.83	100.00	
Missing	.a	not recorded	3	0.05		
	.b	break-off	32	0.49		
	.c	filter: senstec not 3	4855	74.63		
	Total		4890	75.17		
Total			6505	100.00		

clcount13 — Click count p13 (excl. submit) (Benford procedure)

			Count	Percent	Valid %	Cum. %
Valid	0		1486	22.84	91.96	91.96
	1		61	0.94	3.77	95.73
	2		27	0.42	1.67	97.40
	3		13	0.20	0.80	98.21
	4		6	0.09	0.37	98.58
	⋮		⋮	⋮	⋮	⋮
	20		1	0.02	0.06	99.69
	21		1	0.02	0.06	99.75
	25		1	0.02	0.06	99.81
	26		2	0.03	0.12	99.94
	27		1	0.02	0.06	100.00
	Total		1616	24.84	100.00	
Missing	.a	not recorded	2	0.03		
	.b	break-off	32	0.49		
	.c	filter: senstec not 3	4855	74.63		
	Total		4889	75.16		
Total			6505	100.00		

firstcl14 — Secs pageload to first click p14 (Shoplifting)

			Count	Percent	Valid %	Cum. %
Valid	0		17	0.26	0.26	0.26
	.103		1	0.02	0.02	0.28
	.205		1	0.02	0.02	0.29
	.309		1	0.02	0.02	0.31
	.452		1	0.02	0.02	0.33
	⋮		⋮	⋮	⋮	⋮
	161.789		1	0.02	0.02	99.94
	202.121		1	0.02	0.02	99.95
	212.395		1	0.02	0.02	99.97
	213.122		1	0.02	0.02	99.98
	271.936		1	0.02	0.02	100.00
	Total		6448	99.12	100.00	
Missing	.a	not recorded	23	0.35		
	.b	break-off	34	0.52		
	Total		57	0.88		
Total			6505	100.00		

lastcl14 — Secs pageload to last click p14 (excl. submit) (Shoplifting)

			Count	Percent	Valid %	Cum. %
Valid	0		17	0.26	0.26	0.26
	.588		1	0.02	0.02	0.28
	.889		1	0.02	0.02	0.29
	.905		1	0.02	0.02	0.31
	1.076		1	0.02	0.02	0.33
	⋮		⋮	⋮	⋮	⋮
	202.121		1	0.02	0.02	99.94
	212.395		1	0.02	0.02	99.95
	216.987		1	0.02	0.02	99.97
	227.592		1	0.02	0.02	99.98
	271.936		1	0.02	0.02	100.00
	Total		6448	99.12	100.00	
Missing	.a	not recorded	23	0.35		
	.b	break-off	34	0.52		
	Total		57	0.88		
Total			6505	100.00		

submit14 — Secs pageload to submit p14 (Shoplifting)

			Count	Percent	Valid %	Cum. %
Valid	1.014		1	0.02	0.02	0.02
	1.606		1	0.02	0.02	0.03
	1.732		1	0.02	0.02	0.05
	2.111		1	0.02	0.02	0.06
	2.125		1	0.02	0.02	0.08
	⋮		⋮	⋮	⋮	⋮
	218.692		1	0.02	0.02	99.94
	218.867		1	0.02	0.02	99.95
	230.852		1	0.02	0.02	99.97
	273.48		1	0.02	0.02	99.98
	548.748		1	0.02	0.02	100.00
	Total		6448	99.12	100.00	
Missing	.a	not recorded	23	0.35		
	.b	break-off	34	0.52		
	Total		57	0.88		
Total			6505	100.00		

clcount14 — Click count p14 (excl. submit) (Shoplifting)

			Count	Percent	Valid %	Cum. %
Valid	0		23	0.35	0.36	0.36
	1		4185	64.34	64.84	65.20
	2		1500	23.06	23.24	88.44
	3		381	5.86	5.90	94.34
	4		144	2.21	2.23	96.58
	⋮		⋮	⋮	⋮	⋮
	21		3	0.05	0.05	99.89
	22		1	0.02	0.02	99.91
	24		4	0.06	0.06	99.97
	31		1	0.02	0.02	99.98
	35		1	0.02	0.02	100.00
	Total		6454	99.22	100.00	
Missing	.a	not recorded	17	0.26		
	.b	break-off	34	0.52		
	Total		51	0.78		
Total			6505	100.00		

firstcl15 — Secs pageload to first click p15 (Tax evasion)

			Count	Percent	Valid %	Cum. %
Valid	-70.773		1	0.02	0.02	0.02
	0		22	0.34	0.34	0.36
	.297		1	0.02	0.02	0.37
	.476		1	0.02	0.02	0.39
	.5		1	0.02	0.02	0.40
	⋮		⋮	⋮	⋮	⋮
	109.509		1	0.02	0.02	99.94
	130.9		1	0.02	0.02	99.95
	173.497		1	0.02	0.02	99.97
	183.098		1	0.02	0.02	99.98
	335.669		1	0.02	0.02	100.00
	Total		6447	99.11	100.00	
Missing	.a	not recorded	20	0.31		
	.b	break-off	38	0.58		
	Total		58	0.89		
Total			6505	100.00		

lastcl15 — Secs pageload to last click p15 (excl. submit) (Tax evasion)

			Count	Percent	Valid %	Cum. %
Valid	-70.773		1	0.02	0.02	0.02
	0		22	0.34	0.34	0.36
	.476		1	0.02	0.02	0.37
	.561		1	0.02	0.02	0.39
	.713		1	0.02	0.02	0.40
	⋮		⋮	⋮	⋮	⋮
	136.708		1	0.02	0.02	99.94
	141.534		1	0.02	0.02	99.95
	178.262		1	0.02	0.02	99.97
	183.098		1	0.02	0.02	99.98
	335.669		1	0.02	0.02	100.00
	Total		6447	99.11	100.00	
Missing	.a	not recorded	20	0.31		
	.b	break-off	38	0.58		
	Total		58	0.89		
Total			6505	100.00		

submit15 — Secs pageload to submit p15 (Tax evasion)

			Count	Percent	Valid %	Cum. %
Valid	-69.679		1	0.02	0.02	0.02
	.328		1	0.02	0.02	0.03
	.656		1	0.02	0.02	0.05
	.895		1	0.02	0.02	0.06
	1.232		1	0.02	0.02	0.08
	⋮		⋮	⋮	⋮	⋮
	138.415		1	0.02	0.02	99.94
	146.113		1	0.02	0.02	99.95
	179.106		1	0.02	0.02	99.97
	185.125		1	0.02	0.02	99.98
	339.928		1	0.02	0.02	100.00
	Total		6447	99.11	100.00	
Missing	.a	not recorded	20	0.31		
	.b	break-off	38	0.58		
	Total		58	0.89		
Total			6505	100.00		

clcount15 — Click count p15 (excl. submit) (Tax evasion)

			Count	Percent	Valid %	Cum. %
Valid	0		29	0.45	0.45	0.45
	1		4241	65.20	65.71	66.16
	2		1778	27.33	27.55	93.71
	3		292	4.49	4.52	98.23
	4		62	0.95	0.96	99.19
	⋮		⋮	⋮	⋮	⋮
	11		2	0.03	0.03	99.91
	12		3	0.05	0.05	99.95
	14		1	0.02	0.02	99.97
	21		1	0.02	0.02	99.98
	65		1	0.02	0.02	100.00
	Total		6454	99.22	100.00	
Missing	.a	not recorded	13	0.20		
	.b	break-off	38	0.58		
	Total		51	0.78		
Total			6505	100.00		

firstcl16 — Secs pageload to first click p16 (Voting)

			Count	Percent	Valid %	Cum. %
Valid	0		22	0.34	0.34	0.34
	.109		1	0.02	0.02	0.36
	.421		1	0.02	0.02	0.37
	.436		1	0.02	0.02	0.39
	.447		1	0.02	0.02	0.40
	⋮		⋮	⋮	⋮	⋮
	149.307		1	0.02	0.02	99.94
	160.709		1	0.02	0.02	99.95
	169.105		1	0.02	0.02	99.97
	231.075		1	0.02	0.02	99.98
	291.815		1	0.02	0.02	100.00
	Total		6442	99.03	100.00	
Missing	.a	not recorded	23	0.35		
	.b	break-off	40	0.61		
	Total		63	0.97		
Total			6505	100.00		

lastcl16 — Secs pageload to last click p16 (excl. submit) (Voting)

			Count	Percent	Valid %	Cum. %
Valid	0		22	0.34	0.34	0.34
	.421		1	0.02	0.02	0.36
	.436		1	0.02	0.02	0.37
	.447		1	0.02	0.02	0.39
	.47		1	0.02	0.02	0.40
	⋮		⋮	⋮	⋮	⋮
	149.307		1	0.02	0.02	99.94
	160.709		1	0.02	0.02	99.95
	169.701		1	0.02	0.02	99.97
	238.862		1	0.02	0.02	99.98
	291.815		1	0.02	0.02	100.00
	Total		6442	99.03	100.00	
Missing	.a	not recorded	23	0.35		
	.b	break-off	40	0.61		
	Total		63	0.97		
Total			6505	100.00		

submit16 — Secs pageload to submit p16 (Voting)

			Count	Percent	Valid %	Cum. %
Valid	1.154		1	0.02	0.02	0.02
	1.31		1	0.02	0.02	0.03
	1.345		1	0.02	0.02	0.05
	1.373		1	0.02	0.02	0.06
	1.378		1	0.02	0.02	0.08
	⋮		⋮	⋮	⋮	⋮
	150.538		1	0.02	0.02	99.94
	162.118		1	0.02	0.02	99.95
	170.935		1	0.02	0.02	99.97
	243.119		1	0.02	0.02	99.98
	292.703		1	0.02	0.02	100.00
	Total		6442	99.03	100.00	
Missing	.a	not recorded	23	0.35		
	.b	break-off	40	0.61		
	Total		63	0.97		
Total			6505	100.00		

clcount16 — Click count p16 (excl. submit) (Voting)

			Count	Percent	Valid %	Cum. %
Valid	0		28	0.43	0.43	0.43
	1		4414	67.86	68.46	68.89
	2		1723	26.49	26.72	95.61
	3		209	3.21	3.24	98.85
	4		43	0.66	0.67	99.52
	⋮		⋮	⋮	⋮	⋮
	9		1	0.02	0.02	99.94
	10		1	0.02	0.02	99.95
	13		1	0.02	0.02	99.97
	14		1	0.02	0.02	99.98
	15		1	0.02	0.02	100.00
	Total		6448	99.12	100.00	
Missing	.a	not recorded	17	0.26		
	.b	break-off	40	0.61		
	Total		57	0.88		
Total			6505	100.00		

firstcl17 — Secs pageload to first click p17 (Dice game reporting)

			Count	Percent	Valid %	Cum. %
Valid	0		15	0.23	0.23	0.23
	.005		1	0.02	0.02	0.25
	.148		1	0.02	0.02	0.26
	.234		1	0.02	0.02	0.28
	.248		1	0.02	0.02	0.29
	⋮		⋮	⋮	⋮	⋮
	118.144		1	0.02	0.02	99.94
	121.491		1	0.02	0.02	99.95
	147.937		1	0.02	0.02	99.97
	236.06		1	0.02	0.02	99.98
	1158.932		1	0.02	0.02	100.00
	Total		6444	99.06	100.00	
Missing	.a	not recorded	20	0.31		
	.b	break-off	41	0.63		
	Total		61	0.94		
Total			6505	100.00		

lastcl17 — Secs pageload to last click p17 (excl. submit) (Dice game reporting)

			Count	Percent	Valid %	Cum. %
Valid	0		15	0.23	0.23	0.23
	.498		1	0.02	0.02	0.25
	.612		1	0.02	0.02	0.26
	.656		1	0.02	0.02	0.28
	.68		1	0.02	0.02	0.29
	⋮		⋮	⋮	⋮	⋮
	118.144		1	0.02	0.02	99.94
	121.491		1	0.02	0.02	99.95
	147.937		1	0.02	0.02	99.97
	466.237		1	0.02	0.02	99.98
	1158.932		1	0.02	0.02	100.00
	Total		6444	99.06	100.00	
Missing	.a	not recorded	20	0.31		
	.b	break-off	41	0.63		
	Total		61	0.94		
Total			6505	100.00		

submit17 — Secs pageload to submit p17 (Dice game reporting)

			Count	Percent	Valid %	Cum. %
Valid	.078		1	0.02	0.02	0.02
	1.199		1	0.02	0.02	0.03
	1.342		1	0.02	0.02	0.05
	1.435		1	0.02	0.02	0.06
	1.562		1	0.02	0.02	0.08
	⋮		⋮	⋮	⋮	⋮
	146.657		1	0.02	0.02	99.94
	149.419		1	0.02	0.02	99.95
	216.814		1	0.02	0.02	99.97
	468.218		1	0.02	0.02	99.98
	1161.702		1	0.02	0.02	100.00
	Total		6444	99.06	100.00	
Missing	.a	not recorded	20	0.31		
	.b	break-off	41	0.63		
	Total		61	0.94		
Total			6505	100.00		

clcount17 — Click count p17 (excl. submit) (Dice game reporting)

			Count	Percent	Valid %	Cum. %
Valid	0		22	0.34	0.34	0.34
	1		4332	66.59	67.15	67.49
	2		1755	26.98	27.21	94.70
	3		246	3.78	3.81	98.51
	4		44	0.68	0.68	99.19
	⋮		⋮	⋮	⋮	⋮
	16		1	0.02	0.02	99.94
	18		1	0.02	0.02	99.95
	20		1	0.02	0.02	99.97
	21		1	0.02	0.02	99.98
	22		1	0.02	0.02	100.00
	Total		6451	99.17	100.00	
Missing	.a	not recorded	13	0.20		
	.b	break-off	41	0.63		
	Total		54	0.83		
Total			6505	100.00		

firstcl18 — Secs pageload to first click p18 (Trust in survey confidentiality)

			Count	Percent	Valid %	Cum. %
Valid	0		12	0.18	0.19	0.19
	.012		1	0.02	0.02	0.20
	.049		1	0.02	0.02	0.22
	.125		1	0.02	0.02	0.23
	.429		1	0.02	0.02	0.25
	⋮		⋮	⋮	⋮	⋮
	198.203		1	0.02	0.02	99.94
	334.892		1	0.02	0.02	99.95
	341.955		1	0.02	0.02	99.97
	417.013		1	0.02	0.02	99.98
	540.594		1	0.02	0.02	100.00
	Total		6442	99.03	100.00	
Missing	.a	not recorded	20	0.31		
	.b	break-off	43	0.66		
	Total		63	0.97		
Total			6505	100.00		

lastcl18 — Secs pageload to last click p18 (excl. submit) (Trust in survey confidentiality)

			Count	Percent	Valid %	Cum. %
Valid	0		12	0.18	0.19	0.19
	.951		1	0.02	0.02	0.20
	1.558		1	0.02	0.02	0.22
	1.919		1	0.02	0.02	0.23
	1.931		1	0.02	0.02	0.25
	⋮		⋮	⋮	⋮	⋮
	355.027		1	0.02	0.02	99.94
	420.889		1	0.02	0.02	99.95
	435.55		1	0.02	0.02	99.97
	544.187		1	0.02	0.02	99.98
	1000.995		1	0.02	0.02	100.00
	Total		6442	99.03	100.00	
Missing	.a	not recorded	20	0.31		
	.b	break-off	43	0.66		
	Total		63	0.97		
Total			6505	100.00		

submit18 — Secs pageload to submit p18 (Trust in survey confidentiality)

			Count	Percent	Valid %	Cum. %
Valid	.438		1	0.02	0.02	0.02
	1.808		1	0.02	0.02	0.03
	2.117		1	0.02	0.02	0.05
	2.683		1	0.02	0.02	0.06
	2.823		1	0.02	0.02	0.08
	⋮		⋮	⋮	⋮	⋮
	356.541		1	0.02	0.02	99.94
	421.467		1	0.02	0.02	99.95
	437.179		1	0.02	0.02	99.97
	544.969		1	0.02	0.02	99.98
	1002.056		1	0.02	0.02	100.00
	Total		6442	99.03	100.00	
Missing	.a	not recorded	20	0.31		
	.b	break-off	43	0.66		
	Total		63	0.97		
Total			6505	100.00		

clcount18 — Click count p18 (excl. submit) (Trust in survey confidentiality)

			Count	Percent	Valid %	Cum. %
Valid	0		19	0.29	0.29	0.29
	1		6	0.09	0.09	0.39
	2		4117	63.29	63.84	64.23
	3		1382	21.25	21.43	85.66
	4		487	7.49	7.55	93.21
	⋮		⋮	⋮	⋮	⋮
	27		1	0.02	0.02	99.94
	28		1	0.02	0.02	99.95
	30		1	0.02	0.02	99.97
	32		1	0.02	0.02	99.98
	45		1	0.02	0.02	100.00
	Total		6449	99.14	100.00	
Missing	.a	not recorded	13	0.20		
	.b	break-off	43	0.66		
	Total		56	0.86		
Total			6505	100.00		

firstcl19 — Secs pageload to first click p19 (Evaluation of special technique)

			Count	Percent	Valid %	Cum. %
Valid	0		11	0.17	0.20	0.20
	.035		1	0.02	0.02	0.21
	.116		1	0.02	0.02	0.23
	.24		1	0.02	0.02	0.25
	.348		1	0.02	0.02	0.27
	⋮		⋮	⋮	⋮	⋮
	350.29		1	0.02	0.02	99.93
	357.359		1	0.02	0.02	99.95
	561.136		1	0.02	0.02	99.96
	574.817		1	0.02	0.02	99.98
	2289.993		1	0.02	0.02	100.00
	Total		5636	86.64	100.00	
Missing	.a	not recorded	16	0.25		
	.b	break-off	44	0.68		
	.c	filter: DQ	809	12.44		
	Total		869	13.36		
Total			6505	100.00		

lastcl19 — Secs pageload to last click p19 (excl. submit) (Evaluation of special technique)

			Count	Percent	Valid %	Cum. %
Valid	0		11	0.17	0.20	0.20
	1.937		1	0.02	0.02	0.21
	2.511		1	0.02	0.02	0.23
	2.637		1	0.02	0.02	0.25
	2.919		1	0.02	0.02	0.27
	⋮		⋮	⋮	⋮	⋮
	703.24		1	0.02	0.02	99.93
	760.463		1	0.02	0.02	99.95
	931.401		1	0.02	0.02	99.96
	1573.704		1	0.02	0.02	99.98
	2305.64		1	0.02	0.02	100.00
	Total		5636	86.64	100.00	
Missing	.a	not recorded	16	0.25		
	.b	break-off	44	0.68		
	.c	filter: DQ	809	12.44		
	Total		869	13.36		
Total			6505	100.00		

submit19 — Secs pageload to submit p19 (Evaluation of special technique)

			Count	Percent	Valid %	Cum. %
Valid	4.617		1	0.02	0.02	0.02
	4.938		1	0.02	0.02	0.04
	4.954		1	0.02	0.02	0.05
	5.148		1	0.02	0.02	0.07
	5.164		1	0.02	0.02	0.09
	⋮		⋮	⋮	⋮	⋮
	706.482		1	0.02	0.02	99.93
	765.244		1	0.02	0.02	99.95
	963.409		1	0.02	0.02	99.96
	1575.198		1	0.02	0.02	99.98
	2314.688		1	0.02	0.02	100.00
	Total		5636	86.64	100.00	
Missing	.a	not recorded	16	0.25		
	.b	break-off	44	0.68		
	.c	filter: DQ	809	12.44		
	Total		869	13.36		
Total			6505	100.00		

clcount19 — Click count p19 (excl. submit) (Evaluation of special technique)

			Count	Percent	Valid %	Cum. %
Valid	0		17	0.26	0.30	0.30
	1		3	0.05	0.05	0.35
	2		1	0.02	0.02	0.37
	3		1408	21.64	24.96	25.33
	4		2146	32.99	38.04	63.36
	⋮		⋮	⋮	⋮	⋮
	44		1	0.02	0.02	99.93
	45		1	0.02	0.02	99.95
	50		1	0.02	0.02	99.96
	104		1	0.02	0.02	99.98
	182		1	0.02	0.02	100.00
	Total		5642	86.73	100.00	
Missing	.a	not recorded	10	0.15		
	.b	break-off	44	0.68		
	.c	filter: DQ	809	12.44		
	Total		863	13.27		
Total			6505	100.00		

firstcl20 — Secs pageload to first click p20 (Randomizing device test)

			Count	Percent	Valid %	Cum. %
Valid	0		8	0.12	0.12	0.12
	.005		1	0.02	0.02	0.14
	.015		1	0.02	0.02	0.16
	.194		1	0.02	0.02	0.17
	.268		1	0.02	0.02	0.19
	⋮		⋮	⋮	⋮	⋮
	418.675		1	0.02	0.02	99.94
	538.922		1	0.02	0.02	99.95
	722.182		1	0.02	0.02	99.97
	762.37		1	0.02	0.02	99.98
	1080.16		1	0.02	0.02	100.00
	Total		6441	99.02	100.00	
Missing	.a	not recorded	20	0.31		
	.b	break-off	44	0.68		
	Total		64	0.98		
Total			6505	100.00		

lastcl20 — Secs pageload to last click p20 (excl. submit) (Randomizing device test)

			Count	Percent	Valid %	Cum. %
Valid	0		8	0.12	0.12	0.12
	1.125		1	0.02	0.02	0.14
	1.254		1	0.02	0.02	0.16
	1.31		1	0.02	0.02	0.17
	1.326		1	0.02	0.02	0.19
	⋮		⋮	⋮	⋮	⋮
	476.875		1	0.02	0.02	99.94
	557.985		1	0.02	0.02	99.95
	737.181		1	0.02	0.02	99.97
	781.14		1	0.02	0.02	99.98
	1446.677		1	0.02	0.02	100.00
	Total		6441	99.02	100.00	
Missing	.a	not recorded	20	0.31		
	.b	break-off	44	0.68		
	Total		64	0.98		
Total			6505	100.00		

submit20 — Secs pageload to submit p20 (Randomizing device test)

			Count	Percent	Valid %	Cum. %
Valid	4.399		1	0.02	0.02	0.02
	5		1	0.02	0.02	0.03
	5.89		1	0.02	0.02	0.05
	6.376		1	0.02	0.02	0.06
	6.404		1	0.02	0.02	0.08
	⋮		⋮	⋮	⋮	⋮
	480.167		1	0.02	0.02	99.94
	559.077		1	0.02	0.02	99.95
	738.263		1	0.02	0.02	99.97
	782.517		1	0.02	0.02	99.98
	1447.645		1	0.02	0.02	100.00
	Total		6441	99.02	100.00	
Missing	.a	not recorded	20	0.31		
	.b	break-off	44	0.68		
	Total		64	0.98		
Total			6505	100.00		

clcount20 — Click count p20 (excl. submit) (Randomizing device test)

			Count	Percent	Valid %	Cum. %
Valid	0		15	0.23	0.23	0.23
	1		1391	21.38	21.57	21.81
	2		2395	36.82	37.14	58.95
	3		419	6.44	6.50	65.45
	4		1448	22.26	22.46	87.90
	⋮		⋮	⋮	⋮	⋮
	42		2	0.03	0.03	99.94
	43		1	0.02	0.02	99.95
	56		1	0.02	0.02	99.97
	69		1	0.02	0.02	99.98
	106		1	0.02	0.02	100.00
	Total		6448	99.12	100.00	
Missing	.a	not recorded	13	0.20		
	.b	break-off	44	0.68		
	Total		57	0.88		
Total			6505	100.00		

firstcl21 — Secs pageload to first click p21 (Birth date knowledge)

			Count	Percent	Valid %	Cum. %
Valid	0		6	0.09	0.09	0.09
	.169		1	0.02	0.02	0.11
	.255		1	0.02	0.02	0.12
	.318		1	0.02	0.02	0.14
	.546		1	0.02	0.02	0.16
	⋮		⋮	⋮	⋮	⋮
	205.355		1	0.02	0.02	99.94
	215.483		1	0.02	0.02	99.95
	234.862		1	0.02	0.02	99.97
	619.154		1	0.02	0.02	99.98
	700.614		1	0.02	0.02	100.00
	Total		6441	99.02	100.00	
Missing	.a	not recorded	20	0.31		
	.b	break-off	44	0.68		
	Total		64	0.98		
Total			6505	100.00		

lastcl21 — Secs pageload to last click p21 (excl. submit) (Birth date knowledge)

			Count	Percent	Valid %	Cum. %
Valid	0		6	0.09	0.09	0.09
	1.961		1	0.02	0.02	0.11
	2.732		1	0.02	0.02	0.12
	2.839		1	0.02	0.02	0.14
	2.895		1	0.02	0.02	0.16
	⋮		⋮	⋮	⋮	⋮
	210.419		1	0.02	0.02	99.94
	220.979		1	0.02	0.02	99.95
	241.383		1	0.02	0.02	99.97
	628.329		1	0.02	0.02	99.98
	704.176		1	0.02	0.02	100.00
	Total		6441	99.02	100.00	
Missing	.a	not recorded	20	0.31		
	.b	break-off	44	0.68		
	Total		64	0.98		
Total			6505	100.00		

submit21 — Secs pageload to submit p21 (Birth date knowledge)

			Count	Percent	Valid %	Cum. %
Valid	1.301		1	0.02	0.02	0.02
	2.789		1	0.02	0.02	0.03
	3.292		1	0.02	0.02	0.05
	3.674		1	0.02	0.02	0.06
	3.765		1	0.02	0.02	0.08
	⋮		⋮	⋮	⋮	⋮
	211.374		1	0.02	0.02	99.94
	222.004		1	0.02	0.02	99.95
	243.239		1	0.02	0.02	99.97
	629.754		1	0.02	0.02	99.98
	705.681		1	0.02	0.02	100.00
	Total		6441	99.02	100.00	
Missing	.a	not recorded	20	0.31		
	.b	break-off	44	0.68		
	Total		64	0.98		
Total			6505	100.00		

clcount21 — Click count p21 (excl. submit) (Birth date knowledge)

			Count	Percent	Valid %	Cum. %
Valid	0		13	0.20	0.20	0.20
	1		1	0.02	0.02	0.22
	2		1	0.02	0.02	0.23
	3		5	0.08	0.08	0.31
	4		5165	79.40	80.10	80.41
	⋮		⋮	⋮	⋮	⋮
	9		10	0.15	0.16	99.63
	10		14	0.22	0.22	99.84
	11		5	0.08	0.08	99.92
	12		4	0.06	0.06	99.98
	13		1	0.02	0.02	100.00
	Total		6448	99.12	100.00	
Missing	.a	not recorded	13	0.20		
	.b	break-off	44	0.68		
	Total		57	0.88		
Total			6505	100.00		

firstcl22 — Secs pageload to first click p22 (Respondents' comments)

			Count	Percent	Valid %	Cum. %
Valid	0		3087	47.46	47.92	47.92
	.193		1	0.02	0.02	47.94
	.273		1	0.02	0.02	47.95
	.281		1	0.02	0.02	47.97
	.343		1	0.02	0.02	47.98
	⋮		⋮	⋮	⋮	⋮
	91.338		1	0.02	0.02	99.94
	92.675		1	0.02	0.02	99.95
	99.077		1	0.02	0.02	99.97
	147.021		1	0.02	0.02	99.98
	301.018		1	0.02	0.02	100.00
	Total		6442	99.03	100.00	
Missing	.a	not recorded	19	0.29		
	.b	break-off	44	0.68		
	Total		63	0.97		
Total			6505	100.00		

lastcl22 — Secs pageload to last click p22 (excl. submit) (Respondents' comments)

			Count	Percent	Valid %	Cum. %
Valid	0		3087	47.46	47.92	47.92
	.193		1	0.02	0.02	47.94
	.281		1	0.02	0.02	47.95
	.343		1	0.02	0.02	47.97
	.691		1	0.02	0.02	47.98
	⋮		⋮	⋮	⋮	⋮
	572.095		1	0.02	0.02	99.94
	650.932		1	0.02	0.02	99.95
	847.21		1	0.02	0.02	99.97
	929.984		1	0.02	0.02	99.98
	978.615		1	0.02	0.02	100.00
	Total		6442	99.03	100.00	
Missing	.a	not recorded	19	0.29		
	.b	break-off	44	0.68		
	Total		63	0.97		
Total			6505	100.00		

submit22 — Secs pageload to submit p22 (Respondents' comments)

			Count	Percent	Valid %	Cum. %
Valid	1.135		1	0.02	0.02	0.02
	1.174		1	0.02	0.02	0.03
	1.31		1	0.02	0.02	0.05
	1.359		1	0.02	0.02	0.06
	1.39		1	0.02	0.02	0.08
	⋮		⋮	⋮	⋮	⋮
	666.854		1	0.02	0.02	99.94
	712.076		1	0.02	0.02	99.95
	865.234		1	0.02	0.02	99.97
	938.002		1	0.02	0.02	99.98
	1100.615		1	0.02	0.02	100.00
	Total		6442	99.03	100.00	
Missing	.a	not recorded	19	0.29		
	.b	break-off	44	0.68		
	Total		63	0.97		
Total			6505	100.00		

clcount22 — Click count p22 (excl. submit) (Respondents' comments)

			Count	Percent	Valid %	Cum. %
Valid	0		3094	47.56	47.98	47.98
	1		2760	42.43	42.80	90.77
	2		347	5.33	5.38	96.15
	3		113	1.74	1.75	97.91
	4		58	0.89	0.90	98.81
	⋮		⋮	⋮	⋮	⋮
	17		1	0.02	0.02	99.92
	18		2	0.03	0.03	99.95
	21		1	0.02	0.02	99.97
	22		1	0.02	0.02	99.98
	37		1	0.02	0.02	100.00
	Total		6449	99.14	100.00	
Missing	.a	not recorded	12	0.18		
	.b	break-off	44	0.68		
	Total		56	0.86		
Total			6505	100.00		
